# Gut microbial alterations in neonatal jaundice pre- and post-treatment

**DOI:** 10.1042/BSR20210362

**Published:** 2021-04-30

**Authors:** Juan Ding, Xiao Ma, Liping Han, Xianlan Zhao, Ang Li, Qi Xin, Weining Lian, Zhen Li, Hongyan Ren, Zhigang Ren

**Affiliations:** 1 Department of Quality Control, The First Affiliated Hospital of Zhengzhou University, Zhengzhou 450052, China; 2 Department of Gynecology, The First Affiliated Hospital of Zhengzhou University, Zhengzhou 450052, China; 3 Department of Obstetrics, The First Affiliated Hospital of Zhengzhou University, Zhengzhou 450052, China; 4 Department of Infectious Diseases, The First Affiliated Hospital of Zhengzhou University, Zhengzhou 450052, China; 5 Gene Hospital of Henan Province, Precision Medicine Center, The First Affiliated Hospital of Zhengzhou University, Zhengzhou 450052, China; 6 School of Basic Medical Sciences, Zhengzhou University, Zhengzhou 450052, China; 7 Department of Interventional Radiology, The First Affiliated Hospital of Zhengzhou University, Zhengzhou 450052, China; 8 Shanghai Mobio Biomedical Technology Co., Ltd

**Keywords:** Gut microbiota, MiSeq sequencing, Neonatal jaundice, Treatment

## Abstract

Neonatal jaundice is a common disease that affects up to 60% of newborns. Herein, we performed a comparative analysis of the gut microbiome in neonatal jaundice and non-neonatal jaundice infants (NJIs) and identified gut microbial alterations in neonatal jaundice pre- and post-treatment. We prospectively collected 232 fecal samples from 51 infants at five time points (0, 1, 3, 6, and 12 months). Finally, 114 samples from 6 NJIs and 19 non-NJI completed MiSeq sequencing and analysis. We characterized the gut microbiome and identified microbial differences and gene functions. Meconium microbial diversity from NJI was decreased compared with that from non-NJI. The genus *Gemella* was decreased in NJI versus non-NJI. Eleven predicted microbial functions, including fructose 1,6-bisphosphatase III and pyruvate carboxylase subunit B, decreased, while three functions, including acetyl-CoA acyltransferase, increased in NJI. After treatments, the microbial community presented significant alteration-based β diversity. The phyla *Firmicutes* and *Actinobacteria* were increased, while *Proteobacteria* and *Fusobacteria* were decreased. Microbial alterations were also analyzed between 6 recovered NJI and 19 non-NJI. The gut microbiota was unique in the meconium microbiome from NJI, implying that early gut microbiome intervention could be promising for the management of neonatal jaundice. Alterations of gut microbiota from NJI can be of great value to bolster evidence-based prevention against ‘bacterial dysbiosis’.

## Introduction

Neonatal jaundice is the most common condition after birth and often occurs during the first week of life. Approximately 60% of term infants and 80% of preterm infants develop jaundice in the first week of life [1]. There are studies on the association between neonatal jaundice and adverse long-term health outcomes, such as childhood asthma, type 1 diabetes, and impaired visual function [2–4]. Neonatal jaundice is characterized by the presence of high total serum bilirubin levels [5]. Recent progress in understanding the microbiota reveals the role of bacteria in bilirubin metabolism. A study in mouse models of germ-free multidrug resistance 2 knockouts showed increased serum bilirubin levels [6]. There are studies on the possible association between the increase in direct bilirubin and bacteria such as *Bifidobacterium* [7,8]. Thus, it is hypothesized that the gut microbiota is associated with neonatal jaundice, but the gut microbial characteristics in neonatal jaundice remain limited. It is essential to understand the relationship between the gut microbiome and neonatal jaundice and to search for gut microbial alterations in neonatal jaundice pre- and post-treatment.

In the present study, a total of 51 infants were enrolled, and fecal samples were separately collected at 0 month (meconium) and 1, 3, 6, and 12 months postpartum. After confirmation, two premature infants, five infants with incomplete information, and eight infants receiving antibiotic therapy were excluded. After DNA extraction, 16S rRNA gene sequencing and data quality control, 11 neonatal jaundice infant (NJI) meconium samples with insufficient quantities were further discarded. Finally, 6 NJI with treatment and 19 non-NJI were included in the final analysis.

We characterized the meconium microbiome between NJI and non-NJI and identified microbial differences. Furthermore, we assessed gut microbial alterations in neonatal jaundice pre- and post-treatment and reported differences in the gut microbiota of recovered NJI versus non-NJI. The study of NJI gut microbiota development can be of great value to bolster evidence-based prevention against ‘bacterial dysbiosis’, especially in such a populous territory as China.

## Materials and methods

### Participants’ information

The study complied with the ethical guidelines of the Helsinki Declaration and Rules of Good Clinical Practice. The Institutional Review Board of the First Affiliated Hospital of Zhengzhou University approved the studies (2017-KY-12). Informed consent from all participants was obtained before data and stool samples were collected. Mode of delivery and infant medication use were obtained from hospital electronic medical records. Mothers were asked to complete a questionnaire at each time point, including infant diet (exclusive, partial or no breastfeeding), height and weight of the infant, antibiotic use, and Yinzhihuang oral liquid use. Yinzhihuang oral liquid, a well-known Chinese herbal formula, is officially listed in the Chinese Pharmacopoeia, and is a clinical drug for the treatment of neonatal jaundice [9].

The diagnostic criteria for neonatal jaundice followed international guidelines [10,11]. In our study, all NJI received the same brand and manufacturer of Yinzhihuang oral liquid treatment. All NJI received Yinzhihuang oral liquid combined with phototherapy (blue light) treatment (wavelength range: 425–475 nm); and did not receive other medical interventions. Throughout the study, the manufacturer of the light was consistent. All infants included in the study were born by vaginal delivery. The inclusion criteria were as follows: exclusively breastfed, birth weight 2500–4000 g, gestational age 37–42 weeks, and fifth-minute Apgar score 8–10. The exclusion criteria were as follows: hemolysis of newborns, hereditary metabolic diseases, and infectious diseases such as neonatal pneumonia.

### Sample collection and DNA extraction

The study population was recruited before mothers’ parturition. Fecal samples were collected for the first time before infants exhibited jaundice in the hospital. The other fresh fecal samples were collected at their homes, and samples were immediately delivered to the laboratory on dry ice using foam containers. In the laboratory, the sample was stored at −80°C until DNA was extracted. Any samples that stayed at room temperature for more than 2 h were discarded. DNA was extracted from fecal samples according to the manufacturer’s instructions in the E.Z.N.A.® Stool DNA Kit (Omega Bio-tek, Inc., GA). The DNA concentration was determined by a NanoDrop 2000 spectrophotometer (Thermo Scientific), and its molecular size was estimated using agarose gel electrophoresis (0.8%). To assess DNA purity, absorbance ratios were determined at wavelengths of 260 nm relative to 280 nm and 260 nm relative to 230 nm.

### PCR amplification and MiSeq sequencing

The extracted DNA was used as the template to amplify the V3 to V4 regions of the 16S rRNA gene. The forward primer (341F) was 5′-CCTACGGGNGGCWGCAG-3′, and the reverse primer (805R) was 5′-GACTACHVGGGTATCTAATCC-3′. PCR amplification was performed in an EasyCycler 96 PCR system (Analytik Jena Corp., AG) using the following program: 1 cycle at 95°C for 3 min; 21 cycles at 94°C for 30 s, 58°C for 30 s, and 72°C for 30 s; and 1 cycle at 72°C for 5 min. Finally, PCR products were held at 4°C to prevent degradation following the PCR cycle. The products from different samples were mixed at equal ratios for sequencing according to the manufacturer’s instructions, and sequencing was performed on the Illumina MiSeq platform at Shanghai Mobio Biomedical Technology Co. Ltd. Raw Illumina read data for all samples were deposited in the European Bioinformatics Institute European Nucleotide Archive database under accession numbers PRJNA680178 and PRJNA665920.

### Operational taxonomic units and taxonomy annotation

Equal numbers of reads were randomly chosen from all samples, and then operational taxonomic units (OTUs) were binned using the UPARSE pipeline [12]. Sequences with 97% similarity level were clustered into OTUs. RDP classifier version 2.6 software [13] was used to assign sequences to the new bacterial taxonomy.

### Bacterial diversity and taxonomic analysis

Bacterial diversity was assessed by sampling-based analysis of OTUs and presented by the ACE index, which was calculated using the R program package ‘vegan’. Principal coordinates analysis (PCoA) and non-metric multidimensional scaling (NMDS) based on OTU abundance and distribution were conducted by the vegan R package (http://www.R-project.org/) to analyze microbial communities [14]. The weighted and unweighted UniFrac distances were calculated with the phyloseq package [15]. A heatmap of the identified key variables was generated by Heatmap Builder.

Bacterial differences were compared at the phylum and genus levels. Fecal microbial characteristics were analysed by the linear discriminant analysis (LDA) effect size (LEfSe) method (http://huttenhower.sph.harvard.edu/lefse/) [16]. Using a normalized relative abundance matrix, LEfSe performs the Kruskal–Wallis rank sum test to determine characteristics with significantly different abundances between assigned bacteria and uses LDA to assess the effect size of each characteristic.

### Functional annotation of the gut microbial 16S rRNA gene

To predict the functional profiles of microbial communities based on 16S rRNA gene sequences, we utilized the phylogenetic investigation of communities by reconstruction of unobserved states (PICRUSt) version 1.0.0 pipeline [17] and human version 0.99 [18] to establish KEGG orthology (KO) and KEGG pathway/module profiles.

### Statistical analysis

The Wilcoxon rank sum test was used to compare continuous variables between both groups. Fisher’s exact test was used to compare categorical variables. The infant weight-for-length z-score was calculated according to World Health Organization standards [19]. The analyses were performed using SPSS version 21.0. The statistical significance was set at *P*<0.05.

## Results

### Characteristics of the participants

**Figure 1:**
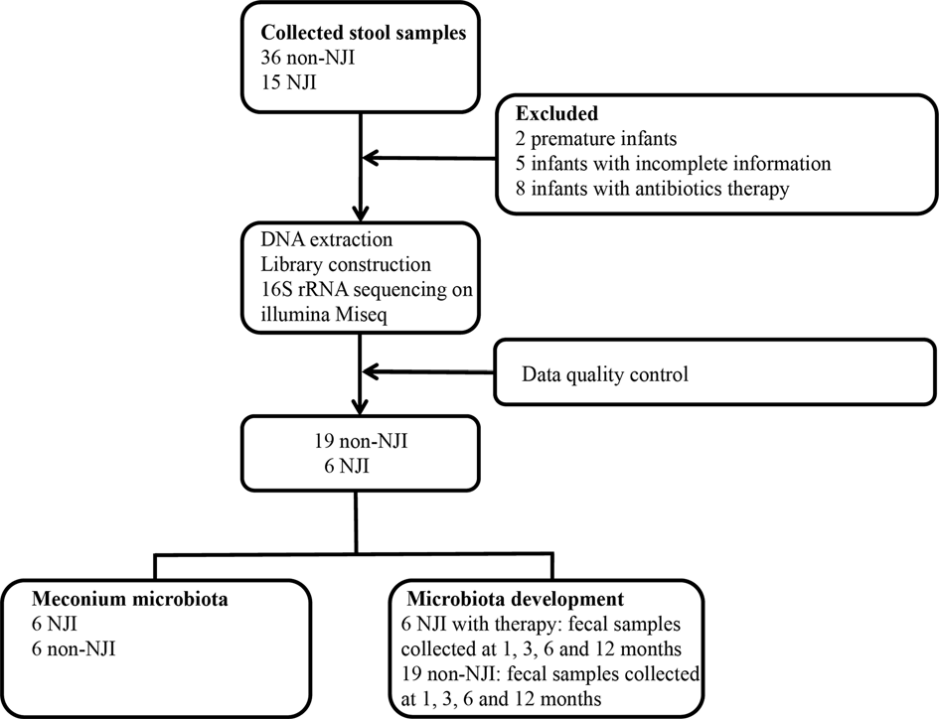
Study design and flow diagram A total of 232 fecal samples from 15 NJI and 36 non-NJI were collected. After a strict pathologic diagnosis and exclusion process, the remaining samples were used for DNA extraction, 16S rRNA sequencing and data quality control. Finally, 6 NJI with treatment and 19 non-NJI were utilized for bioinformatics analysis.

A total of 51 infants (232 fecal samples) were collected from Zhengzhou, Central China. After a strict pathological diagnosis and exclusion process, a total of 114 fecal samples from 6 NJI with treatment and 19 non-NJI were included (Figure 1). In the meconium cohort, all individuals included were required to be infants resulting from full-term pregnancies and vaginal deliveries and to be exclusively breastfed.

The demographic, clinical, and anthropometric characteristics of the 25 neonates are presented in Table 1. Among all participants, there were no significant differences in age, sex, birth weight, or birth length between NJI and non-NJI. The agarose gel electrophoresis results are shown in Supplementary Figure S1. DNA was extracted from 438 fecal samples. And exclusively breastfed infants, combined feeding infants, infants born by cesarean section, and infants born by vaginal delivery were included in Supplementary Figure S1.

**Table 1:** Characteristics of the study population

Variables	Cases (*n*=6)	Controls (*n*=19)	*P*-value
Maternal age (years)	29.67 ± 3.01	32.26 ± 4.32	0.19
Prenatal BMI (kg/m^2^)	25.72 ± 3.73	28.68 ± 3.55	0.09
Pregnancy weight gain (kg)	13.78 ± 4.52	15.88 ± 3.17	0.21
Newborn sex			
Male	4 (0.67)	12 (0.63)	0.64
Female	2 (0.33)	7 (0.37)	
Birth weight (g)	3241.67 ± 363.89	3489.47 ± 233.68	0.06
Birth length (cm)	51.67 ± 1.03	51.53 ± 1.22	0.80
Delivery mode			
Vaginal delivery	5 (0.83)	12 (0.63)	0.35
Cesarean section delivery	1 (0.17)	7 (0.37)	
Feeding patterns			
Exclusive breastfeeding	4 (0.67)	15 (0.79)	1.00
Non-exclusive breastfeeding	2 (0.33)	4 (0.21)	
Apgar score			
1 min	9.33 ± 0.82	9.37 ± 0.76	0.92
5 min	9.50 ± 0.55	9.63 ± 0.50	0.59
Gestational age (days)	282.17 ± 6.65	280.11 ± 8.03	0.58

Continuous variables were compared using the Wilcoxon rank sum test. Fisher’s exact test was used to compare categorical variables.

### Gut microbiomes are unique in NJI at 0 month

**Figure 2:**
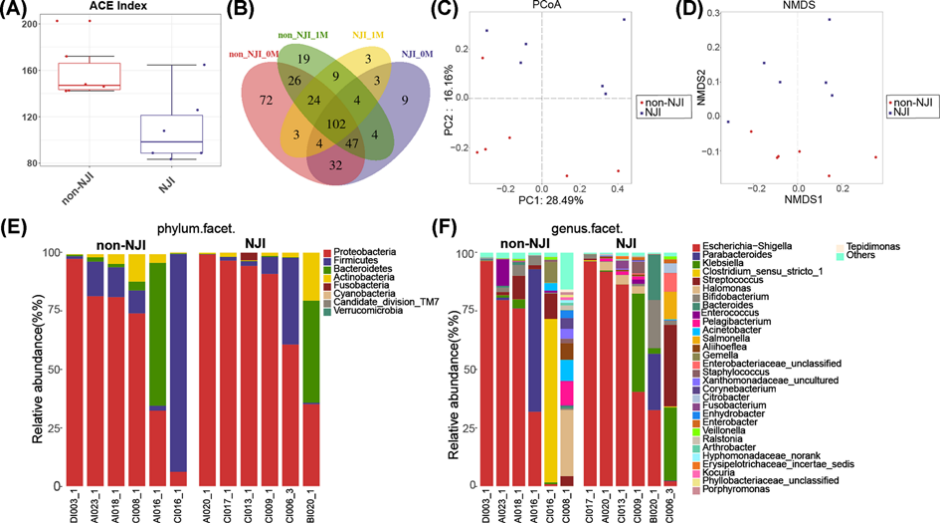
Altered meconium microbiota composition in NJI (**A**) Microbial α diversity decreased in NJI, as shown by the ACE estimator. (**B**) Shared and unique genera among NJI-0M, non-NJI-0M, NJI-1M, and non-NJI-1M. Overall diversity was calculated using unweighted UniFrac by PCoA (**C**) and NMDS (**D**), indicating a separation of samples between NJI and non-NJI. Fecal microbiota composition at the phylum level (**E**) and genus level (**F**) between NJI and non-NJI.

We collected meconium from NJI and matched non-NJI at 0 month. Compared with the non-NJI group, fecal microbial diversity, as estimated by the ACE estimator, was markedly decreased in NJI (*P*<0.05, Figure 2A, Supplementary Table S1). The Venn analysis showed that 185 of 330 OTUs were shared between NJI-0M and non-NJI-0M (Figure 2B). To assess similarity among microbial communities, we performed PCoA and NMDS analysis based on unweighted UniFrac distance (Figure 2C,D, Supplementary Tables S2 and S3).

A heatmap of the identified key variables demonstrated that a total of 27 key OTUs were significantly different between NJI and non-NJI (Supplementary Figure S2 and Table S4). We further analyzed the infant gut microbiota composition and alterations at the phylum and genus levels between the two groups. The fecal bacterial composition in each sample at the phylum and genus levels is shown in Figure 2E,F (Supplementary Tables S5 and S6). The average composition of the microbial community at the phylum and genus levels between the two groups is shown in Supplementary Figure S3A,B and Tables S7 and S8. The bacterial phyla *Proteobacteria, Firmicutes*, and *Bacteroidetes*, together accounting for up to 90% of sequences on average, were three dominant populations in the two groups (Figure 2E). Bacterial genera of *Escherichia-Shigella, Parabacteroides, Klebsiella*, and *Clostridium sensu stricto 1*, together accounting for up to 70% of sequences on average, were the four dominant populations in the two groups (Figure 2F). The average amount of *Gemella* was markedly decreased in NJI versus non-NJI (*P*<0.05, Supplementary Figure S3C and Table S9). Although there were no significant differences in the average relative abundances of *Klebsiella* and *Clostridium* between NJI and non-NJI (both *P*>0.05, Supplementary Table S9), changes in average relative abundances of *Klebsiella* and *Clostridium* were still noted in NJI (Supplementary Figure S3B).

**Figure 3:**
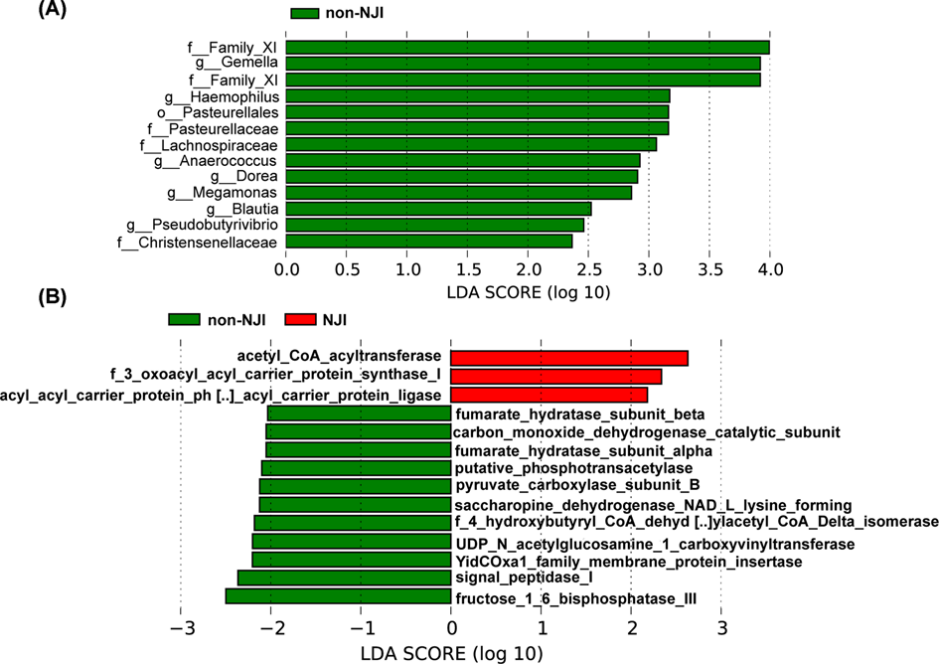
Identification of specific bacterial taxa and microbial functions associated with neonatal jaundice (**A**) The greatest differences in taxa between NJI and non-NJI are presented according to the LDA scores (log10). (**B**) Differences in gut microbial functions between NJI and non-NJI based on the LDA scores (log10).

We used LDA effective size (LEfSe) to determine the specific bacterial taxa related to neonatal jaundice. A cladogram representative of fecal microbial structure and their predominant bacteria displayed the greatest differences in taxa between NJI and non-NJI (all *P*<0.05, Supplementary Figure S4 and Table S10). Meanwhile, the cladogram of fecal microbial structure between NJI and non-NJI also showed the greatest differences in taxa (all *P*<0.05, LDA > 2, Figure 3A, Supplementary Table S10), which suggested gut microbial alterations in NJI.

Microbial metabolic function predictions using the PICRUSt pipeline [17] assessed the potential microbial functions associated with neonatal jaundice and again showed significant differences between the two groups. Based on LDA selection, 3 predicted microbial functions, mainly acetyl-CoA acyltransferase and 3 oxoacyl acyl carrier protein synthase I, were enriched, while 11 functions, mainly fructose 1,6-bisphosphatase III, signal peptidase I and YidCOxa1 family membrane protein insertase, were reduced in NJI versus non-NJI (all *P*<0.05, LDA > 2, Figure 3B, Supplementary Table S11).

### Gut microbial alterations before and after treatment

**Figure 4:**
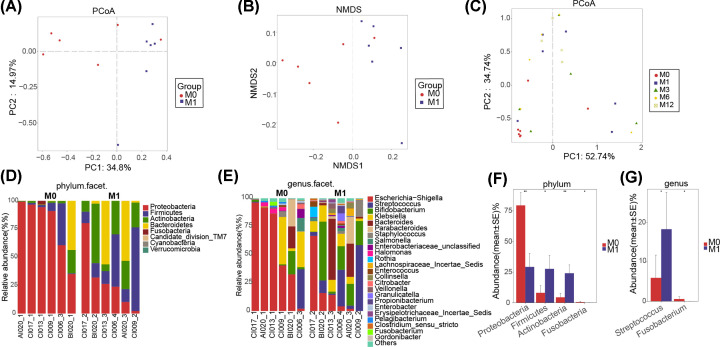
Gut microbial differences of infants between pre-treatment (NJI-0M) and post-treatment (NJI-1M) Overall diversity was calculated using unweighted UniFrac by PCoA (**A**) and NMDS (**B**), indicating a separation of samples between pre-treatment and post-treatment. (**C**) PCoA based on the weighted UniFrac distance in NJI with treatment to assess the microbial distribution among 0, 1, 3, 6, and 12 months, indicating that samples tended to be uniform at 0 and 12 months, and samples are most heterogeneous at 1–6 months. Fecal microbiota composition at the phylum level (**D**) and genus level (**E**) between pre-treatment and post-treatment. (**F**) Compared with pre-treatment, two phyla were significantly increased, while two phyla were significantly decreased post-treatment (all *P*<0.05). (**G**) One genus was increased whereas one genus was decreased in pre-treatment versus post-treatment (all *P*<0.05). Abbreviations: M0, 0 month; M1, 1 month; M3, 3 months; M6, 6 months; M12, 12 months.

The Venn analysis showed that 113 of 244 OTUs were shared between pre-treatment (NJI-0M) and post-treatment (NJI-1M) (Figure 2B). To display microbiome space between pre-treatment and post-treatment, we performed PCoA and NMDS analysis based on unweighted UniFrac distance (*P*<0.05, Figure 4A,B, Supplementary Tables S12 and S13). Moreover, PCoA was conducted based on weighted UniFrac distances to assess the microbial distribution at 0, 1, 3, 6, and 12 months (Figure 4C, Supplementary Table S14). PCoA indicated that the samples tended to be uniform at 0 and 12 months, and no obvious separation was observed in NJI with treatment. Notably, samples were most heterogeneous at the age of 1–6 months.

A heatmap of the identified key variables demonstrated that a total of 26 key OTUs were significantly different between the two groups (Supplementary Figure S5 and Table S15). Fecal bacterial composition and differences at the phylum and genus levels between the two groups are shown in Figure 4D–G, respectively (all *P*<0.05, Supplementary Tables S16–S19).

**Figure 5:**
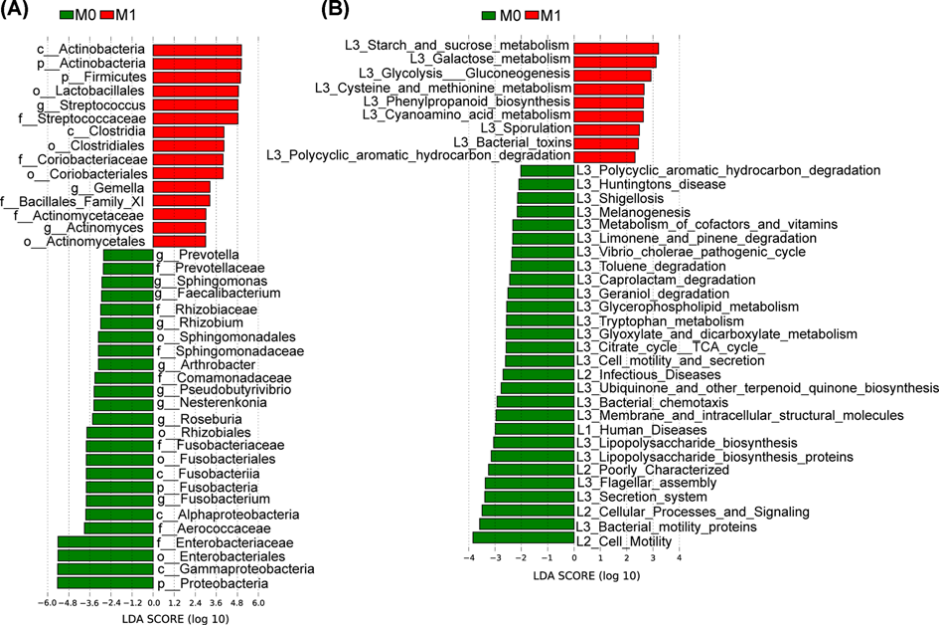
Identification of specific bacterial taxa and microbial functions between pre-treatment (0 month) and post-treatment (1 month) (**A**) The greatest differences in taxa between pre-treatment and post-treatment are presented according to the LDA scores (log10). (**B**) Differences in gut microbial functions between pre-treatment and post-treatment based on the LDA scores (log10). Abbreviations: M0, 0 month; M1, 1 month.

We detected the greatest differences in taxa between pre-treatment and post-treatment using the LEfSe method and LDA scores, as shown in Figure 5A and Supplementary Figure S6A (all *P*<0.05, LDA > 2.4, Supplementary Table S20).

The predominant fecal microbial functions between pre-treatment and post-treatment are shown by a cladogram and LDA (all *P*<0.05, LDA > 2, Figure 5B and Supplementary Figure S6B and Table S21). These data revealed significant differences between the two groups.

### Gut microbiota alterations in recovered NJI and non-NJI

**Figure 6:**
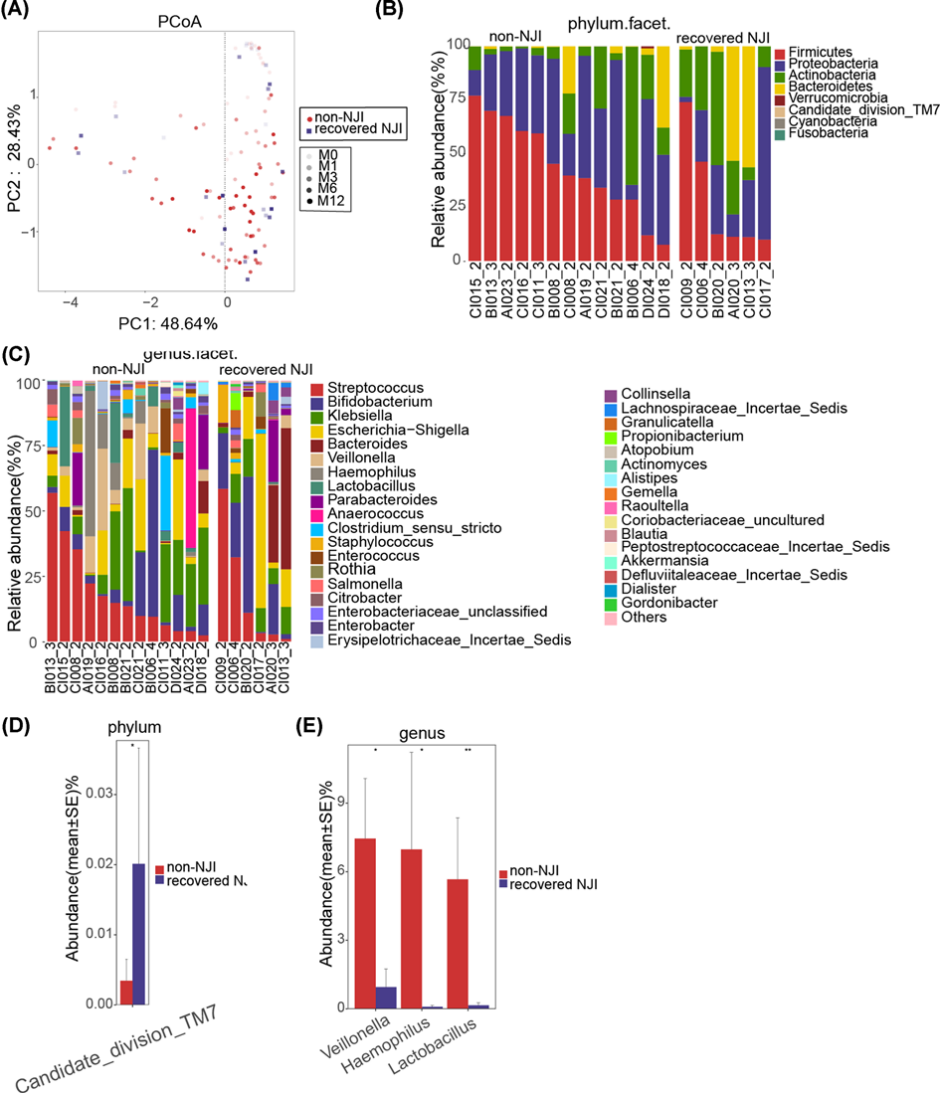
Gut microbial alterations of infants between recovered NJI and non-NJI (**A**) PCoA based on the weighted UniFrac distance between recovered NJI and non-NJI at 0, 1, 3, 6, and 12 months, indicating that the microbial communities became more uniform over time. Fecal microbiota composition at the phylum level (**B**) and genus level (**C**) between recovered NJI and non-NJI. (**D**) Compared with recovered NJI, one phylum was significantly decreased in non-NJI (*P*<0.05). (**E**) Three genera were increased in non-NJI versus recovered NJI (all *P*<0.05). Abbreviations: M0, 0 month; M1, 1 month; M3, 3 months; M6, 6 months; M12, 12 months.

The Venn analysis showed that 139 of 248 OTUs were shared between recovered NJI (NJI-1M) and non-NJI (non-NJI-1M) at 1 month (Figure 2B). To display the microbiome space between recovered NJI and non-NJI at 0, 1, 3, 6, and 12 months, we performed PCoA based on weighted UniFrac distances (Figure 6A, Supplementary Table S22). These data revealed that distinctly separate bacterial communities were present between recovered NJI and non-NJI at an early age, while the microbial communities became more uniform over time.

A heatmap of the identified key variables revealed that a total of 13 key OTUs were significantly different between recovered NJI and non-NJI at 1 month (Supplementary Figure S7 and Table S23). Fecal bacterial composition and differences at the phylum and genus levels between the two groups at 1 month are shown in Figure 6B–E (all *P*<0.05, Supplementary Table S24–S27).

**Figure 7:**
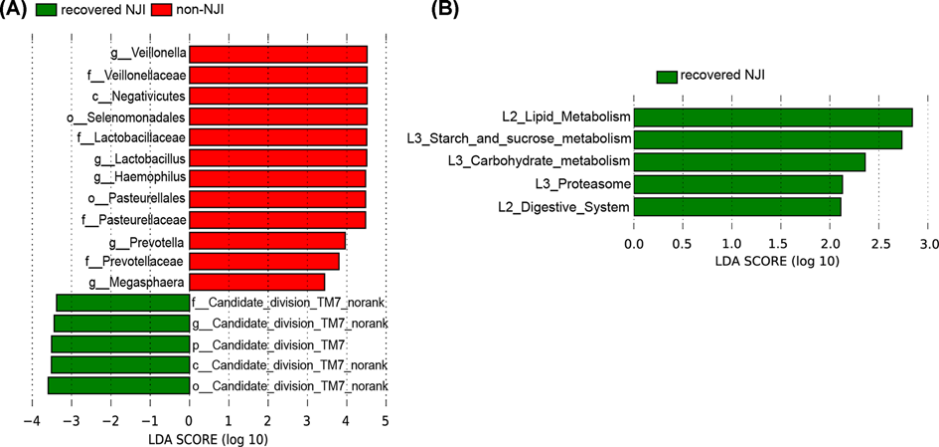
Identification of specific bacterial taxa and microbial functions between recovered NJI and non-NJI (**A**) The greatest differences in taxa between recovered NJI and non-NJI are presented according to the LDA scores (log10). (**B**) Differences in gut microbial functions between recovered NJI and non-NJI based on the LDA scores (log10).

We detected the greatest differences in taxa between recovered NJI and non-NJI at 1 month using the LEfSe method and LDA scores, as shown in Figure 7A and Supplementary Figure S8A (all *P*<0.05, LDA > 3, Supplementary Table S28).

The predominant fecal microbial functions between recovered NJI and non-NJI patients at 1 month are shown by a cladogram and LDA (all *P*<0.05, LDA > 2, Figure 7B and Supplementary Figure S8B and Table S29).

### Effect of medication on infant growth

These observations prompted us to explore the potential relationship between NJI treatment and infant growth. Thus, we examined whether the weight-for-length z-score at 12 months differed between recovered NJI and non-NJI. A *t* test for independent samples was used to compare the development and growth of recovered NJI and non-NJI at 12 months of age. The infant weight-for-length z-score was calculated according to World Health Organization standards [19]. The infant weight-for-length z-score at 12 months did not differ significantly between recovered NJI and non-NJI (*P*<0.05). Our study demonstrated that growth at 12 months of age is independent of NJI treatment in early life.

## Discussion

We illustrated that neonatal jaundice was associated with the altered composition and function of gut microbiota, as well as a decrease in α-diversity. Dong et al. [20] characterized meconium microbiome of NJIs and found that a-diversity were lower in the NJIs compared with controls, which is consistent with our study. Recent studies have reported that a high level of α-diversity was associated with a low risk of necrotizing enterocolitis, atopic eczema, and neonatal sepsis [21–23]. Our study suggested that neonatal jaundice can cause gut microbiota dysbiosis. And we observed a higher α-diversity of gut microbes at birth. Moreover, the bacterial community of neonates at risk of jaundice was separated from that of non-NJIs.

The gut microbiota is indispensable to the health of the host. Healthy infants may share some key microbiota structural features, whereas neonates at risk of jaundice may have aberrant patterns and lack some key bacteria, leading to a ‘dysbiosis’ state. We found that although the relative abundance of *Bifidobacterium* was lower in the non-NJI compared with NJI at 0 and 1 month, but *P*-values were not significant (*P*>0.05). *Bifidobacterium* was identified to exert antimicrobial activity against pathogens in infant gut, which could benefit the colonization of healthy bacteria [24,25]. Tuzun et al. [8] indicated that down-regulation of members of the *Bifidobacterium* genus, such as *B. longum, B. bifidum*, and *B. adolescentis*, was associated with an increase in serum bilirubin level. Our study showed no significant difference in the relative abundance of *Bifidobacterium* between two groups. We speculated that this is because all infants included were exclusively breastfed, and breast milk promoted the colonization of *Bifidobacterium* [26].

We observed that there was only one feature more abundant in the non-NJI (*Gemella*), and this bacterial genera can produce short-chain fatty acids (SCFAs) [27]. SCFAs (particularly propionate and butyrate) initiate several complementary mechanisms within the intestinal mucosa that activate intestinal gluconeogenesis [28]. Notably, butyrate plays an important role in bacterial energy metabolism and intestinal mucosa health in humans, is the major energy source of the intestinal mucosa, and is an important regulator of gene expression, inflammation, differentiation, and apoptosis in host cells [29–32]. LEfSe showed that some butyrate-producing bacteria, such as *Blautia* and *Pseudobutyrivibrio*, were increased in non-NJI versus NJI. A study indicated that *Pseudobutyrivibrio* are producers of butyrate, lactic acid, and formic acid [33]. In addition, members of the genus *Blautia* produce acetate, ethanol, hydrogen, lactate, or succinate, which can provide energy for the host [34]. Moreover, non-NJI harbor more beneficial populations, such as *Lachnospiraceae*, one of the major taxonomic groups of the gut microbiota, which degrade complex polysaccharides to SCFAs, including acetate, butyrate, and propionate, which can be used by the host as sources of energy [35]. These results indicated that gut microbial community alteration might play a key role during neonatal jaundice initiation.

Different microbial functions and metabolites are determined by different microbial communities, thereby contributing to the pathogenesis and development of different diseases [7,36,37]. Gut microbial functions involving fructose 1,6-bisphosphatase III and pyruvate carboxylase subunit B were enriched in non-NJI according to the LDA scores (log10). Fructose 1,6-bisphosphatase III, a key enzyme in intestinal gluconeogenesis, exerts important physiological functions in the regulation of energy metabolism and glucose homeostasis [38–40]. Previous studies have shown that an increase in lactate was found in cholestasis and are consistent with our results [41,42]. Lactate is a substrate of intestinal gluconeogenesis. Low levels of expression of the fructose 1,6-bisphosphatase III and pyruvate carboxylase subunit B enzymes involved in gluconeogenesis decreased the clearance of lactate. In addition, in infancy, the body has a great demand for energy, and the body may produce more ATP through glycolysis to maintain energy metabolism, resulting in increased lactate. Thus, neonates with limited capacity to metabolize lactate via intestinal gluconeogenesis may be associated with neonatal jaundice.

Our study analyzed the development of NJI with treatment at 0, 1, 3, 6, and 12 months. We found that gut microbiota differences within the NJI and treatment groups were completely decreased over time, suggesting that treatment can only temporarily perturb the NJI gut microbiota. Moreover, we conducted a longitudinal study of recovered NJI and non-NJI, and PCoA revealed that the microbial community of recovered NJI was clustered with that of non-NJI over time, suggesting that the microbiota of recovered NJI tends to recover to similar levels as non-NJI gradually and that the effect of treatment on gut microbiota is temporary. Importantly, infants’ growth and development at 12 months did not differ significantly between recovered NJI and non-NJI. Taken together, these results demonstrated that the gut microbiota composition was influenced by treatment at early stages, that these differences were absent over time and that the gut microbiota gradually recovered. Thus, we propose that treatment may have little long-term effect on infant health.

In summary, the present study comprehensively characterized the meconium microbiome between NJI and non-NJI and identified microbial differences. The combination of early gut microbiome intervention and currently used treatment methods may further benefit NJI. Moreover, we illustrated the gut microbial alterations and development in NJI with treatment, which may provide a solid foundation for future health outcomes through microbiota intervention.

## Supplementary Material

Supplementary Figures S1-S8 and Tables S1-S29

## Data Availability

The raw Illumina read data for all samples were deposited in the European Bioinformatics Institute European Nucleotide Archive database under the accession numbers PRJNA680178 and PRJNA6659201.

## Abbreviations

ACE: abundance-based coverage estimator
KEGG: Kyoto Encyclopedia of Genes and Genomes
LDA: linear discriminant analysis
LEfSe: LDA effect size
NJI: neonatal jaundice infant
NMDS: non-metric multidimensional scaling
OTU: operational taxonomic unit
PCoA: principal coordinates analysis
PICRUSt: phylogenetic investigation of communities by reconstruction of unobserved states
SCFA: short-chain fatty acid
