# Supplementary material for: Gut microbial alterations in neonatal jaundice pre- and post-treatment
Source: Biosci Rep. 2021 Apr 30;41(4):BSR20210362. doi: 10.1042/BSR20210362 (PMC8150162; doi:10.1042/BSR20210362)
Supplement: Supplementary Figures S1-S8 and Tables S1-S29 [file BSR-2021-0362_supp.pdf]

Supplementary Figure 1

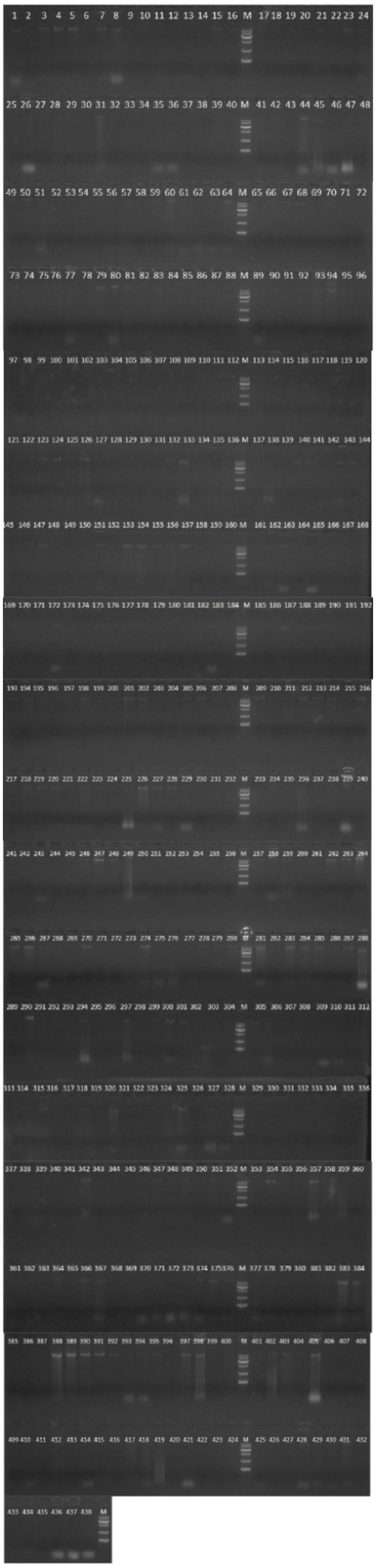

Figure S1: Agarose gel electrophoresis (0.8%) of the genomic DNA. M is a molecular weight marker.

Supplementary Figure 2

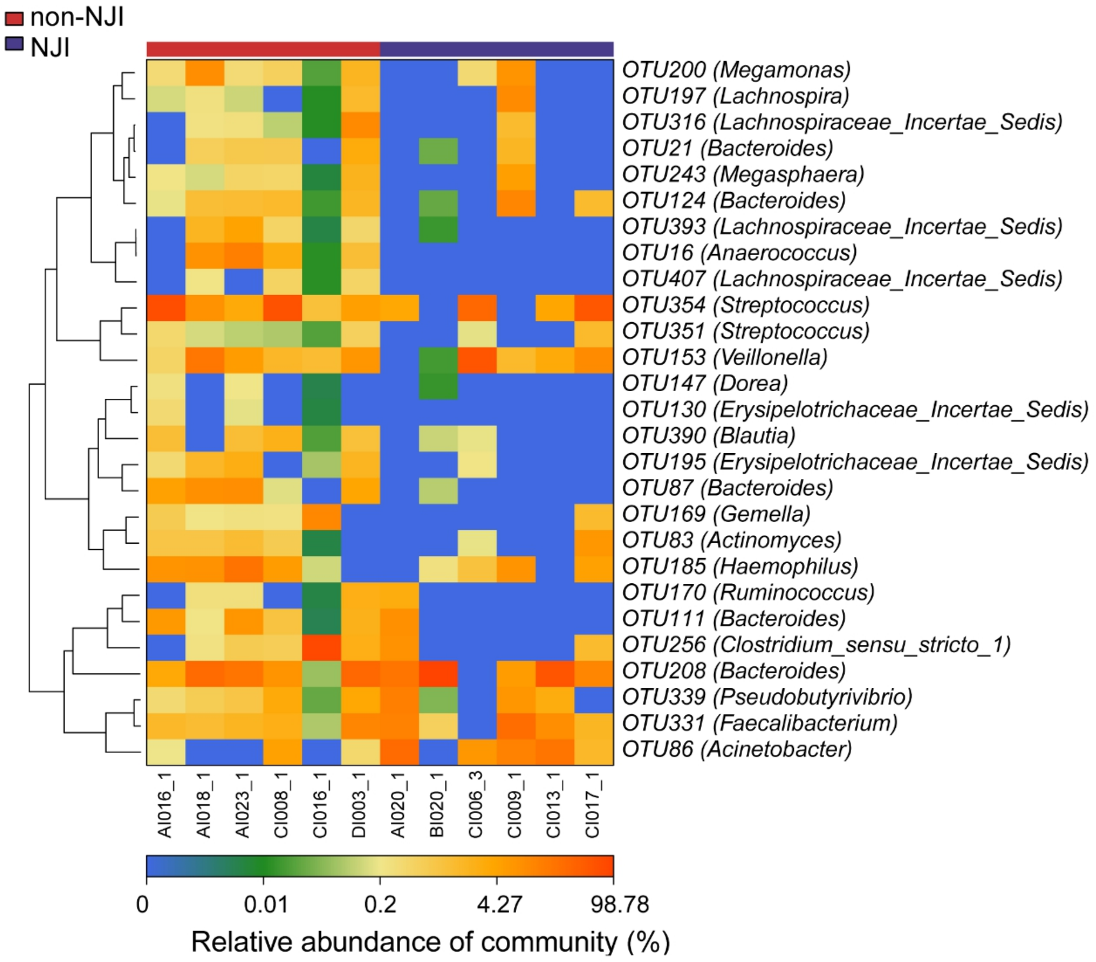

Figure S2: The heatmap showing the relative abundance of the discriminatory OTUs that drive the differences between NJI and non-NJI. Each vertical lane corresponds to one sample. Abundance values range from blue (low abundance) to red (high abundance). NJI: neonatal jaundice infants; OTUs: Operational Taxonomy Units.

Supplementary Figure 3

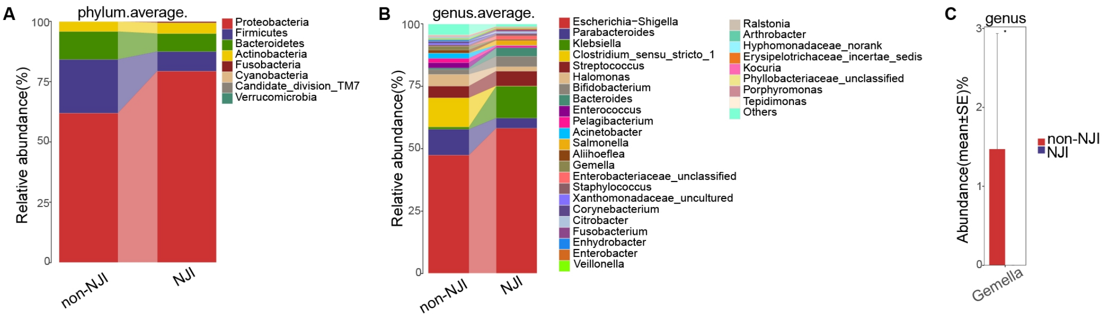

Figure S3: Faecal bacterial composition and differences at the phylum and genus levels between NJI and non-NJI. Faecal microbiota composition at the phylum level **(A)** and genus level **(B)** between NJI and non-NJI. **(C)** Compared with non-NJI, 1 genus was significantly decreased in NJI ($P < 0.05$). NJI: neonatal jaundice infants.

Supplementary Figure 4

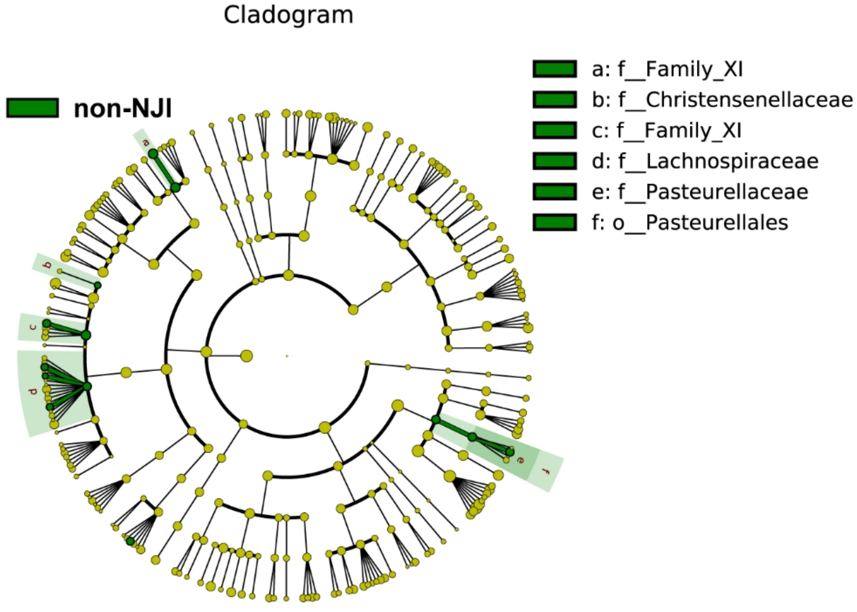

Figure S4: The phylogenetic profiles of the specific bacterial taxa and predominant bacteria between NJI and non-NJI using the LEfSe method. NJI: neonatal jaundice infants; LEfSe: linear discriminant analysis effect size.

Supplementary Figure 5

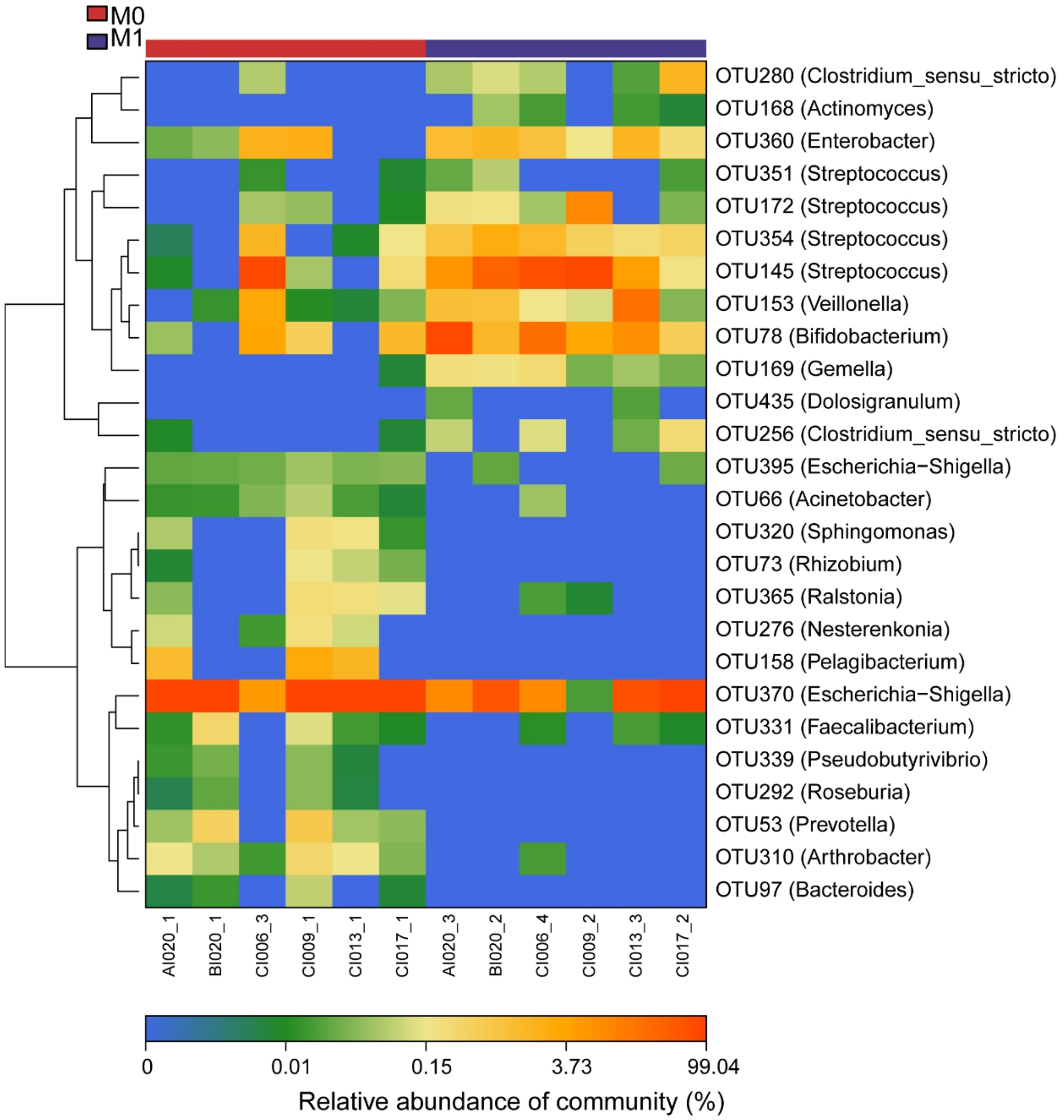

Figure S5: The heatmap showing the relative abundance of the discriminatory OTUs that drive the differences between pre-treatment (0 month) and post-treatment (1 month). Each vertical lane corresponds to one sample. Abundance values range from blue (low abundance) to red (high abundance). OTUs: Operational Taxonomy Units; M0: 0 month; M1: 1 month.

Supplementary Figure 6

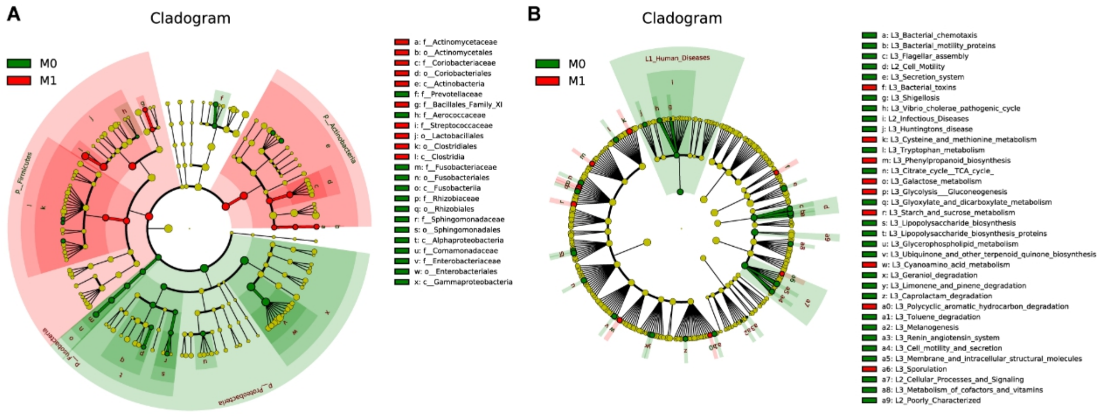

Figure S6: Identification of specific bacterial taxa and microbial functions between pre-treatment (0 month) and post-treatment (1 month). (A) The phylogenetic profiles of the specific bacterial taxa and predominant bacteria between pre-treatment and post-treatment using the LEfSe method. **(B)** A cladogram showed the gut microbial community function profiles between pre-treatment and post-treatment and their predominant microbial functions. LEfSe: linear discriminant analysis effect size; M0: 0 month; M1: 1 month.

Supplementary Figure 7

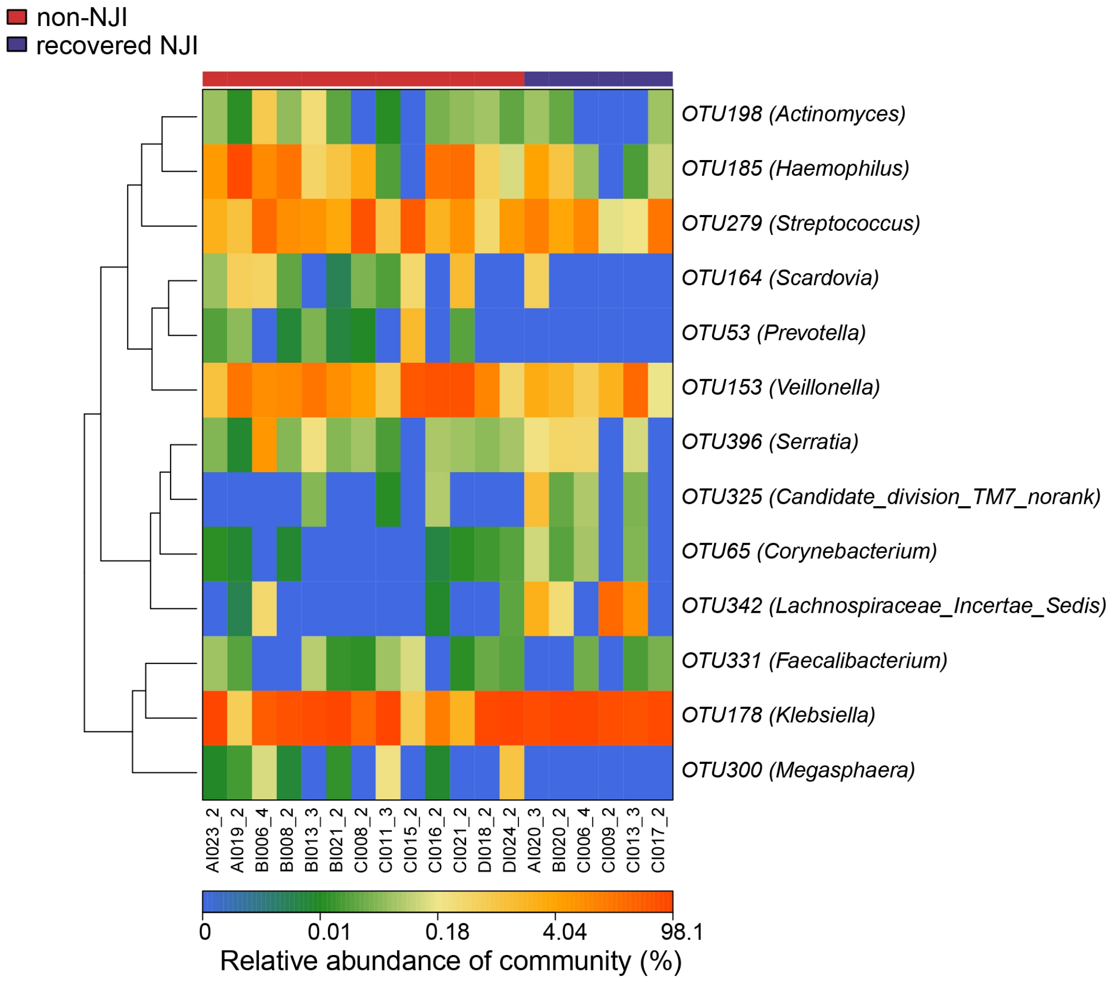

Figure S7: The heatmap showing the relative abundance of the discriminatory OTUs that drive the differences between recovered NJI and non-NJI. Each vertical lane corresponds to one sample. Abundance values range from blue (low abundance) to red (high abundance). NJI: neonatal jaundice infants; OTUs: Operational Taxonomy Units.

Supplementary Figure 8

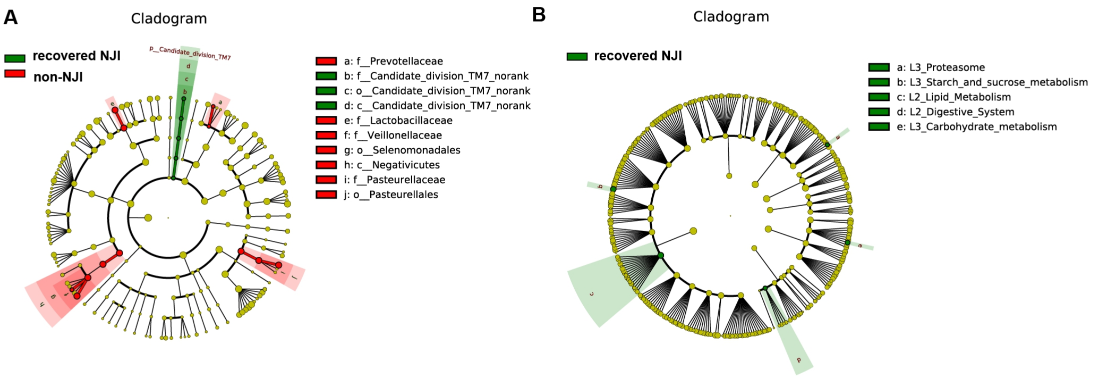

Figure S8: Identification of specific bacterial taxa and microbial functions between recovered NJI and non-NJI. (A) The phylogenetic profiles of the specific bacterial taxa and predominant bacteria between recovered NJI and non-NJI using the LEfSe method. **(B)** A cladogram showed the gut microbial community function profiles between recovered NJI and non-NJI and their predominant microbial functions. NJI: neonatal jaundice infants; LEfSe: linear discriminant analysis effect size.

Table S1. The faecal microbial diversity between neonatal jaundice infants (NJI) and non-NJI at 0 months

Alpha_diversity	non-NJI-mean	non-NJI-se	NJI-mean	NJI-se	p-value	Sig_mark
ace	158.9252042	9.841059533	109.8237025	12.71495375	0.025974026	*

Table S2. Unweighted UniFrac distance of each sample between neonatal jaundice infants (NJI) and non-NJI at 0 months

sample_ID	Standard deviation	Proportion of Variance
PC1	0.304432741	0.2849
PC2	0.229262906	0.16157
PC3	0.207736574	0.13266
PC4	0.165664528	0.08436
PC5	0.15623387	0.07503
PC6	0.146965778	0.06639
PC7	0.131810752	0.05341
PC8	0.121436486	0.04533
PC9	0.116760733	0.04191
PC10	0.105492524	0.03421
PC11	0.081113636	0.02023
PC12	1.17E-18	0

Table S3. NMDS Unweighted UniFrac of each sample between neonatal jaundice infants (NJI) and non-NJI at 0 months

sample_ID	MDS1	MDS2
AI016_1	-0.211437187621236	-0.0450003754968305
AI018_1	0.00810643389785243	-0.102836361655339
AI023_1	-0.0882327307308082	-0.116436532868024
CI008_1	0.354885397794292	-0.119795810440494
CI016_1	-0.0937108550559234	-0.120979708517486
DI003_1	0.142267680660846	-0.173825952330022
AI020_1	0.129590706217887	0.0979363994416613
BI020_1	-0.151403952371367	0.155248608180162
CI006_3	-0.314425567494251	-0.0159777127808886
CI009_1	0.157636191387458	0.0607706848783304
CI013_1	0.145560497038072	0.281601722526563
CI017_1	-0.0788366137228223	0.0992950390623682

Table S4. The identified key OTUs of a heatmap between neonatal jaundice infants (NJI) and non-NJI at 0 months

OTU	AI016_1	AI018_1	AI023_1	CI008_1	CI016_1	DI003_1	AI020_1	BI020_1	CI006_3	CI009_1	CI013_1	CI017_1
OTU86 (Acinetobacter)	0.00203206	0	0	0.05068319	0	0.00399855	0.28706625	0	0.06923514	0.13538	0.20106289	0.01746575
OTU331 (Faecalibacterium)	0.01768646	0.01437316	0.02049804	0.02656201	0.0007907	0.11959288	0.12933754	0.00589125	0	0.259688	0.0894597	0.01986301
OTU339 (Pseudobutyrivibrio)	0.00368782	0.00677979	0.00956938	0.03541602	0.00026228	0.03816794	0.14195584	0.00040701	0	0.0719678	0.03100089	0
OTU208 (Bacteroides)	0.03567397	0.27320626	0.20139191	0.07695805	0.00057494	0.32079244	0.20662461	0.98783657	0	0.0558631	0.60141718	0.11541096
OTU256 (Clostridium_sensu_stricto_1)	0	0.00255695	0.00739452	0.00653282	0.85415926	0.02671756	0.08201893	0	0	0	0	0.01541096
OTU111 (Bacteroides)	0.0629939	0.00228576	0.07193345	0.01062481	4.39E-05	0.02326427	0.0851735	0	0	0	0	0
OTU170 (Ruminococcus)	0	0.00286688	0.00288169	0	5.43E-05	0.02689931	0.02996845	0	0	0	0	0
OTU185 (Haemophilus)	0.0786483	0.08120254	0.21569161	0.05539735	0.00133851	0	0	0.002823	0.0115155	0.0764972	0	0.05034247
OTU83 (Actinomyces)	0.00955821	0.00945297	0.01413658	0.00720285	5.43E-05	0	0	0	0.00194294	0	0	0.06815068
OTU169 (Gemella)	0.00737563	0.00224702	0.00282732	0.00258441	0.1135705	0	0	0	0	0	0	0.01541096
OTU87 (Bacteroides)	0.0483179	0.08790485	0.09460635	0.00167508	0	0.04053072	0	0.00088186	0	0	0	0
OTU195 (Erysipelotrichaceae_Incertae_Sedis)	0.00376308	0.01913839	0.02832753	0	0.00072998	0.02090149	0	0	0.00222728	0	0	0
OTU390 (Blautia)	0.01354708	0	0.01386472	0.02596377	0.00019251	0.01181389	0	0.00113755	0.00194294	0	0	0
OTU130 (Erysipelotrichaceae_Incertae_Sedis)	0.00383834	0	0.00179426	0	5.68E-05	0	0	0	0	0	0	0
OTU147 (Dorea)	0.00278468	0	0.00217486	0	4.91E-05	0	0	0.00013567	0	0	0	0
OTU153 (Veillonella)	0.00466622	0.18956299	0.05632884	0.01835411	0.01463705	0.07233733	0	0.00016176	0.58923325	0.0161047	0.03542958	0.10034247
OTU351 (Streptococcus)	0.0039136	0.0013947	0.00097869	0.00078968	0.00020543	0.00563431	0	0	0.00175339	0	0	0.01609589
OTU354 (Streptococcus)	0.69240611	0.08062142	0.03371031	0.60508746	0.01236571	0.05179935	0.03785489	0	0.31831106	0	0.04162976	0.56609589
OTU407 (Lachnospiraceae_Incertae_Sedis)	0	0.0023245	0	0.00519275	0.00010982	0.00490731	0	0	0	0	0	0
OTU16 (Anaerococcus)	0	0.08282969	0.14016964	0.03084544	0.00010982	0.0132679	0	0	0	0	0	0
OTU393 (Lachnospiraceae_Incertae_Sedis)	0	0.02053309	0.04605263	0.00473809	5.43E-05	0.00436205	0	0.00014611	0	0	0	0
OTU124 (Bacteroides)	0.0019568	0.01363707	0.01489778	0.01636794	0.00015633	0.01962923	0	0.00027134	0	0.1207851	0	0.01541096
OTU243 (Megasphaera)	0.00225785	0.00143344	0.00473032	0.00457058	6.20E-05	0.02290076	0	0	0	0.05385	0	0
OTU21 (Bacteroides)	0	0.00612118	0.00826446	0.00760966	0	0.03144311	0	0.00030787	0	0.0201309	0	0
OTU316 (Lachnospiraceae_Incertae_Sedis)	0	0.00263443	0.00293606	0.00098112	0.00010465	0.10359869	0	0	0	0.0156014	0	0
OTU197 (Lachnospira)	0.00135471	0.00267318	0.00125054	0	0.00010594	0.01617594	0	0	0	0.0976346	0	0
OTU200 (Megamonas)	0.00353729	0.09421974	0.00358852	0.00586279	0.00021189	0.02126499	0	0	0.0038385	0.0764972	0	0

Table S5. Faecal bacterial composition in each sample at the phylum level at 0 months

OTUID	AI016_1	AI018_1	AI023_1	CI008_1	CI016_1	DI003_1	AI020_1	BI020_1	CI006_3	CI009_1	CI013_1	CI017_1
Actinobacteria	0.044965	0.049845	0.0211	0.122505	0.003919	0.010114	0.006418	0.206243	0.021101	0.015217	0.002826	0.018123
Bacteroidetes	0.610094	0.014118	0.018594	0.037683	0.003233	0.00648	0.001152	0.4361	0.002248	0.00453	0.001271	0.000922
Candidate_division_TM7	6.70E-05	3.40E-05	0	0.000264	0	0	0	0	4.60E-05	0	0	5.30E-05
Cyanobacteria	0	0	0	0.000756	0.000153	5.90E-05	4.50E-05	0	0	0	0	0.000773
Firmicutes	0.020765	0.126879	0.147423	0.097746	0.929858	0.011279	0.001347	0.006115	0.369938	0.072339	0.019696	0.015382
Fusobacteria	2.00E-05	0	0	0.001533	0	0	0.000181	0	0	3.30E-05	0.034168	4.10E-05
Proteobacteria	0.324089	0.809124	0.812883	0.739513	0.062837	0.972068	0.990857	0.351542	0.606667	0.907779	0.942039	0.964706
Verrucomicrobia	0	0	0	0	0	0	0	0	0	0.000102	0	0

Table S6. Faecal bacterial composition in each sample at the genus level at 0 months

OTUID	AI016_1	AI018_1	AI023_1	CI008_1	CI016_1	DI003_1	AI020_1	BI020_1	CI006_3	CI009_1	CI013_1	CI017_1
Abiotrophia	0	0	0	0.00069	0	4.40E-05	0	0	0	0	0	4.50E-05
Acinetobacter	2.70E-05	9.30E-05	2.80E-05	0.090275	0.033354	0.001364	0.00043	3.30E-05	0.004688	0.000596	0.000366	0.000143
Actinobacillus	0	0	0	0	0	0	0	0	0.000409	0	0	0
Actinomyces	0.000326	0.000485	0.000376	0.000917	7.40E-05	0	0	0	0.000215	3.90E-05	0	0.000199
Aerococcus	0	0	0	0.001101	3.90E-05	0.000312	7.10E-05	0	0	4.00E-05	3.70E-05	0
Agaricicola	0	0	0	0.000209	0	0	0	0	0	0	0	0
Akkermansia	0	0	0	0	0	0	0	0	0	0.000102	0	0
Alcaligenaceae_unclassified	0	0	0	0.00044	0	0	0	0	0	0	0	0
Aliihoeflea	0	0	0	0.070545	0	0	0.00797	0	0	0.007537	0.006354	0
Alistipes	0	0	0.000186	0.000209	0	0.000106	0	0	0	0	0	0
Allobaculum	0	0	0	0	0.000119	0	0	0	0	0	0	0
Alloprevotella	0	0	0	0	0	0	0	0	0	0.000264	0	0
Anaerococcus	0	0.00216	0.002578	0.005297	8.50E-05	0.000159	2.40E-05	0	0	0.000319	0	0
Anaerosporobacter	0	0	0	0	0	0	0	0	9.30E-05	0	0	0
Anaerostipes	3.40E-05	0	2.20E-05	0	3.90E-05	0	0	0.00018	0	0	0	0
Anaerotruncus	0	0	2.60E-05	0	0	0	0	0	0	0	0	0
Anoxybacillus	0	0.000101	0	0.00025	0	0.00263	6.80E-05	0	0	0	0	0
Aquabacterium	0	0	0	0.009409	0	0	0.00089	0	0	0.001005	0.001175	0
Arthrobacter	0	0.000395	1.50E-05	0.011189	0	0.003406	0.00144	0.00019	4.10E-05	0.001491	0.001406	0.000288
Atopobium	0.000114	0.000516	0.00039	0	0.000309	0	0	0	0.000348	0	0	9.70E-05
Atopostipes	0	0	0	0.000508	0	0	0	0	0	0	0	0
Bacteroides	0.002006	0.012174	0.00773	0.011398	0.000858	0.004027	0.00061	0.19398	7.80E-05	0.000833	0.000725	0.000482
Bifidobacterium	0.038811	0.04558	0.018632	0.006605	0.003024	0.003327	0.00215	0.2059	0.018535	0.009316	3.00E-05	0.016004
Bilophila	0	6.50E-05	0	0	0	0	0	0	0	0	0	0
Blautia	0.000287	0.000122	0.000374	0.001165	0.000345	0.00035	2.70E-05	0.00028	4.10E-05	0	9.40E-05	0
Brachybacterium	0	0	0	0.000899	0	0	0	0	0	0	0	0
Brevibacillus	0	0	0	0	0	0.000734	0	0	0	0	0	0
Brevibacterium	0	0	0	0.008388	0	0.000203	5.20E-05	0	0	0	3.70E-05	0
Brevundimonas	0	0.002546	0	0.001541	0	0.000114	8.80E-05	0	5.00E-05	0	3.50E-05	0
Butyricicoccus	6.20E-05	0	0	0	0	0	0	0	0	0	0	0
Candidate_division_TM7_norank	6.70E-05	3.40E-05	0	0.000264	0	0	0	0	4.60E-05	0	0	5.30E-05

Carnobacterium	0	0	0	0.000159	0	0	0	0	0	0	0	0
Caulobacter	0	0	0	0.00019	0	0	3.30E-05	0	0	3.40E-05	4.50E-05	0
Christensenellaceae_uncultured	0	0	3.60E-05	0.000734	3.50E-05	1.50E-05	0	0	0	0	0	0
Chryseobacterium	0	0	0	0.003224	0	0	0	0	0	4.00E-05	0	4.30E-05
Citrobacter	0.00074	0.000587	0.000581	0.00013	0.001131	0.000826	0.00029	3.60E-05	0.040719	0.0006	0	0.000236
Clostridium_sensu_stricto_1	4.10E-05	0.000729	0.00055	0.000349	0.69768	0.000147	8.20E-05	0	0.003968	3.80E-05	0	0.004383
Collinsella	0.000239	0.001585	0.001407	0	0.000115	0.000132	0.00034	3.00E-05	0	4.30E-05	4.10E-05	0
Comamonadaceae_unclassified	1.70E-05	0	0	0.004763	0	2.90E-05	0.00082	0	0	0.000759	0.001114	0
Comamonas	0	0	0	0.000302	0	0	0	0	0	0	0	0
Coprobacillus	0	0	0	0	0	0	0	0	0	0	0.003668	0
Coprococcus	8.80E-05	6.20E-05	0	4.30E-05	0	0	0	9.10E-05	0	0	3.30E-05	0
Coriobacteriaceae_uncultured	0.003449	0	0	0	0	0	0	0	0	6.40E-05	0	0
Corynebacteriaceae_uncultured	0	2.70E-05	0	2.90E-05	0	0	0	0	0	0	0	0
Corynebacterium	0	5.60E-05	0	0.043791	0	0.000574	0	9.40E-05	7.00E-05	0.002874	0.0002	0
Curvibacter	3.40E-05	0.000316	0	0	0	0	0	0	0	0	0	4.90E-05
Cyanobacteria_norank	0	0	0	0.000756	0.000153	5.90E-05	4.50E-05	0	0	0	0	0.000773
Defluviitaleaceae_incertae_sedis	6.50E-05	0	0	0	0.000262	0	0	0	0	0	0	0
Delftia	2.20E-05	0	0	0.000165	0	2.60E-05	0	0	0	0	0	0
Dermabacter	0	0	0	0.000622	0	0	0	3.30E-05	0	0	0	0
Desemzia	0	0	0	0	0	0.000307	0	0	0	0	0	0
Dialister	0	3.60E-05	1.60E-05	0	3.60E-05	0.000183	0	0	0	0.000113	0	0
Dietzia	0	0	0	0.005399	0	0	0	0	0	0	0	0
Dolosigranulum	3.30E-05	0	0	0.001503	0	0	0	0	0	0	0	0
Dorea	3.70E-05	0.000212	0.000117	0	3.80E-05	0	0	2.60E-05	0	0	0	0
Dysgonomonas	0	3.40E-05	0	0	0	0	0	0	0	0	0	0
Eggerthella	0.000989	5.90E-05	2.00E-05	0	0	0.000113	5.70E-05	0	0	0	0	0
Enhydrobacter	0	3.40E-05	0	0.034123	0	0.000336	0	0	0	0	6.80E-05	0
Enterobacter	5.10E-05	0.001976	0.000835	0	0.000346	2.90E-05	0.00024	0.00028	0.017141	0.012437	0	0
Enterobacteriaceae_unclassified	7.90E-05	0.001439	0.000327	4.20E-05	0.00031	0	0.00014	0.00049	0.079234	0.004818	0	5.20E-05
Enterococcus	0.000332	0.002145	0.112452	0.002632	0.00853	0	0	0	0.000328	0.018139	4.00E-05	0.000525
Erysipelotrichaceae_incertae_sedis	0.000127	0.000494	0.000554	0	0.000609	0.000219	0	0	4.70E-05	0	0.013087	0
Erysipelotrichaceae_uncultured	0	2.70E-05	4.50E-05	0	0.000159	2.30E-05	2.20E-05	3.50E-05	0	0	0	0
Escherichia-Shigella	0.319434	0.760052	0.796282	0.000637	0.009926	0.962178	0.91747	0.3264	0.022755	0.404309	0.862634	0.960977

Faecalibacterium	0.000235	0.000371	0.000377	0.00111	0.000612	0.000658	8.20E-05	0.00113	0	0.000516	0.000101	5.80E-05
Finegoldia	0	0	1.80E-05	0.004863	7.30E-05	0	0	0	0	0	0	0
Flavonifractor	0	2.90E-05	2.00E-05	0	0	0.000113	0	0	2.80E-05	0	0.000257	6.10E-05
Fusobacterium	2.00E-05	0	0	0.001533	0	0	0.00018	0	0	3.30E-05	0.034168	4.10E-05
Gemella	9.80E-05	5.80E-05	5.20E-05	0.000108	0.087903	0	0	0	0	0	0	4.50E-05
Geobacillus	0	0	0	0	0	0.000263	0	0	0	0	0	0
Gordonibacter	0	0	0	0	0	0	2.70E-05	0	4.50E-05	0	0	0
Granulicatella	0.000866	0	0	0.001867	0	0	0	0	0.000267	0	0	0
Haemophilus	0.001045	0.002096	0.003967	0.002883	0.00808	6.60E-05	0	0.00057	0.000286	0.000152	0	0.000147
Halomonas	0	7.40E-05	0	0.282815	0	0	0.03603	0	0	0.031203	0.040311	0
Herbaspirillum	0	0	0	0.000652	0	0	0.00012	0	0	7.80E-05	0.0004	0
Hyphomonadaceae_norank	0	0	0	0.011626	0	0	0.00179	0	0	0.00117	0.00233	0
Janibacter	0	0	0	0.001384	0	0	0	0	0	0	0	0
Klebsiella	0.000412	0.037417	0.009891	0.000455	0.008036	0.000329	0.00536	0.0236	0.313272	0.419447	0.000317	0.001237
Kocuria	0	0	0	0.011641	0	0.000623	0.0011	0	0	0.000117	0.00011	0
Kytococcus	0	0	0	0.006481	0	0	2.90E-05	0	0	0	0	0
Lachnoanaerobaculum	0	0	2.30E-05	0	0	0	0	0	0	0	4.00E-05	0
Lachnospira	1.80E-05	9.90E-05	4.80E-05	0.001072	8.20E-05	8.90E-05	0	5.90E-05	0	0.000459	0	0
Lachnospiraceae_incertae_sedis	0.000215	0.00243	0.002374	0.002735	0.001284	0.001112	0.00023	0.00024	0.000192	0.000412	0.001651	0.00024
Lachnospiraceae_unclassified	0	4.20E-05	8.50E-05	0.000321	0.000112	3.30E-05	0	0.0001	0	7.90E-05	0	0
Lachnospiraceae_uncultured	0	7.20E-05	0	0	4.90E-05	7.20E-05	0	0	0	0	0	0
Lactobacillales_unclassified	2.50E-05	3.30E-05	0	0	0	0	0	0	0.000198	0	0	0
Lactobacillus	0.000741	0.002903	0.001689	0.000519	0.001373	9.10E-05	0.00014	0.00133	0.002694	0.000832	0	0.001864
Lactococcus	0	6.90E-05	0	0	0.001089	0	0.00013	0	0	0	0.000179	0
Lautropia	0	0	0	0.00139	0	0	0	0	0	0	0	0
Leuconostoc	0	0	0	0.000194	0	0	0	0	0	0	0	0
Macrococcus	0	0	0	0.001918	0	2.40E-05	0	0	0	0	0	0
Megamonas	4.70E-05	0.006387	0.000151	0.000284	0.000164	0.000117	0	0	8.10E-05	0.000199	0	0
Megasphaera	3.00E-05	0.00026	8.70E-05	0.000191	0.000196	0.000126	3.20E-05	0	0	0.000107	0	0.000156
Methylobacterium	0	0	0	0	0	7.50E-05	0	0	0	0	0	0
Methyloversatilis	0	3.00E-05	0	0.000148	0	0.000159	0	0	0	3.50E-05	3.50E-05	0
Microbacteriaceae_unclassified	0	0	0	0.00083	0	0	0.00016	0	0	0.000165	0	0
Microbacterium	0	0	0	0	0	3.40E-05	0	0	0	0	0	0

Micrococcus	0	0	0	0.004184	0	0.001171	0	0	0	0	8.60E-05	0
Moraxella	0	0	0	8.20E-05	0	0	0	0	0	0	0	0
Morganella	0	0.000198	7.20E-05	0	0	0	0	0	0	0	0	0
Neisseria	0.001929	0	0	0.002465	0	2.10E-05	0	3.20E-05	0	0	0	0
Nesterenkonia	0	0	0	0.007156	0	0	0.00091	0	4.10E-05	0.001002	0.000844	0
Nocardioidaceae_uncultured	0	0	0	0.00206	0	0	0	0	0	0	0	0
Novosphingobium	0	0	0	0.003928	0	0	0.00044	0	0	0.000409	0.000519	0
Oceanobacillus	0	0	0	0	0	0	0	0	0	0	7.30E-05	0
Oribacterium	0	0	0	7.40E-05	0	0	0	0	0	0	0	0
Parabacteroides	0.608088	0.001177	0.010318	0.000882	0.000124	0.000326	7.70E-05	0.24069	0.002125	3.80E-05	6.20E-05	5.00E-05
Paracoccus	0	0	0	0.003668	0	0.001388	0	0	0	0	0	0
Paraprevotella	0	0	2.20E-05	0	3.30E-05	2.50E-05	2.40E-05	0	0	0	0	0
Parasutterella	0	0	0	0.000982	0	5.40E-05	0	0	0	0	0.000179	0
Parvimonas	0	0	0	0.000283	0	0	0	0	0	0	0	0
Pediococcus	0	0	0	0	0	0	0	0	0	0.000486	0	0
Pelagibacterium	0	0	0	0.103192	0	0	0.01172	0	0	0.013228	0.014396	0
Pelomonas	0	0	0	0.007716	0	0	0.0009	0	0	0.00105	0.000901	0
Peptoniphilus	0	0	0	0.002933	0	0	0	0	0	0	0	0
Peptostreptococcaceae_incertae_sedis	5.10E-05	0.000486	0.007981	0.000188	0.000186	0	0	5.70E-05	0.00013	0	0	0.000132
Phascolarctobacterium	0	0	2.10E-05	0.000183	0	9.80E-05	0	0	0	0	0	0
Phyllobacteriaceae_unclassified	0	0	0	0.010177	0	0	0.00092	0	0	0.001202	0.0012	0
Porphyromonas	0	0	0	0.011812	0	0	0	0	4.50E-05	0	0	0
Prevotella	0	0.000733	0.000338	0.007163	0.002218	0.001878	0.00041	0.00144	0	0.003355	0.000484	0.000347
Prevotellaceae_uncultured	0	0	0	0	0	3.00E-05	0	0	0	0	0	0
Propionibacterium	0	2.50E-05	0	0.007642	0	0.000312	0	0	0.000115	0	0	0
Proteus	0	3.30E-05	2.80E-05	0	4.10E-05	0	0	0	0	0	0	0
Pseudobutyrivibrio	4.90E-05	0.000175	0.000176	0.00148	0.000203	0.00021	9.00E-05	7.80E-05	0	0.000143	3.50E-05	0
Pseudochrobactrum	0	0	0	0.000357	0	0	3.10E-05	0	0	3.90E-05	3.50E-05	0
Pseudomonadales_unclassified	0	0	0	0	0	0.000207	0	0	0	0	0	0
Pseudomonas	0	0	0	0.003099	0	0.000763	0	0	0	0	0	0
Psychrobacter	0	0	0	0.001541	0	0	0	0	0	0	0.000222	0
Ralstonia	5.30E-05	0.000995	3.70E-05	0.019543	8.20E-05	0	0.00032	0	0	0.00117	0.001946	0.001342
Raoultella	0	0.000152	0.000208	0	0.000305	8.30E-05	2.90E-05	0	0.00833	0.000109	0	5.80E-05

Rhizobium	2.70E-05	0.000424	0	0.00358	0	0.000117	4.60E-05	0	0	0.000677	0.000729	0.000239
Rhodobacter	0	0	0	0.000328	0	6.30E-05	0.00028	0	0	0	0	0
Rhodococcus	0	0	0	0.000154	0	0.000185	0	0	0	0	0	0
Roseburia	0	3.60E-05	5.00E-05	0	4.20E-05	8.60E-05	2.90E-05	5.90E-05	0	0.000147	3.20E-05	0
Rothia	0.001037	0.001045	0.000199	0.000736	0.000397	3.40E-05	0	0	0.001569	0	0	0.001535
Rubrobacter	0	0	0	0.001387	0	0	0	0	0	0	0	0
Ruminococcaceae_incertae_sedis	0	6.00E-05	0	0.000151	0	8.20E-05	0	0	0	0	0	0
Ruminococcaceae_uncultured	2.10E-05	0.000125	0.000186	8.80E-05	7.80E-05	0.000647	2.40E-05	0.00019	0	0	0	4.80E-05
Ruminococcus	8.30E-05	0.000105	8.20E-05	0	0.000283	0.000148	7.10E-05	0.00011	0	0	0	0
S24-7_norank	0	0	0	0.000796	0	0	2.40E-05	0	0	0	0	0
Saccharopolyspora	0	0	0	0.001011	0	0	0.00015	0	0	0.000106	7.20E-05	0
Salmonella	0.000199	0.000405	0.000136	0.00022	0.001154	6.10E-05	0.0002	0	0.118132	0.000537	0	4.20E-05
Scardovia	0	7.20E-05	6.10E-05	0	0	0	0	0	0.000122	0	0	0
Serratia	2.00E-05	0.000131	0.000491	0.00109	7.20E-05	0.000378	7.00E-05	3.00E-05	0.001651	0.00022	0.000186	9.20E-05
Solobacterium	0	0	0	0.000275	0	0	0	0	0	0	0	0
Sphingobacterium	0	0	0	0.002199	0	0	0	0	0	0	0	0
Sphingobium	0	0	0	0	0	0.000428	0	0	0	0	0	0
Sphingomonas	0	3.00E-05	0	0.008423	0	0.002165	0.00054	0	0	0.001079	0.001609	9.20E-05
Sphingopyxis	0	0	0	0	0	0.000738	0	6.10E-05	0	0	0	0
Staphylococcus	0.000289	0.000539	0.002031	0.019359	0.008447	0.001345	5.50E-05	0.00188	0.00085	0.049693	0.000282	0.000498
Stenotrophomonas	0	0	0	0.000888	0	0	2.20E-05	0	0	0	0	0
Streptococcus	0.016777	0.100952	0.01367	0.04202	0.108139	0.000444	0.00012	0	0.348415	0.000395	4.70E-05	0.006927
Subdoligranulum	5.20E-05	5.40E-05	0.000161	0.000332	0.000238	0	5.60E-05	0.00024	0	8.00E-05	0	0
Sutterella	0	3.10E-05	0	3.30E-05	0	7.10E-05	0	2.30E-05	0	6.90E-05	0	0
Tepidimonas	0	0	0	0.011289	0	0	0	0	0	0	0	0
Veillonella	6.20E-05	0.005506	0.001371	0.000767	0.011329	0.000398	0	3.10E-05	0.012606	3.20E-05	4.00E-05	0.0004
Weissella	0	0	0	0	0	0	0	0	0	0.00011	0	0
Xanthomonadaceae_uncultured	0	0	0	0.0422	0	0	0.0036	0	0	0.003702	0.004857	0
Zoogloea	0	0	0	0.001301	0	0	6.30E-05	0	0	0.000108	7.60E-05	0
env.OPS_17_norank	0	0	0	0	0	8.80E-05	0	0	0	0	0	0
mitochondria_norank	0	0	0	0.000671	0	0	0	0	0	0	0	0

Table S7. Faecal bacterial composition at the phylum level between neonatal jaundice infants (NJI) and non-NJI at 0 months

ID	non-NJI	NJI
Proteobacteria	0.620085667	0.793931667
Firmicutes	0.222325	0.080802833
Bacteroidetes	0.115033667	0.0743705
Actinobacteria	0.042074667	0.044988
Fusobacteria	2.59E-04	0.005737167
Cyanobacteria	1.61E-04	1.36E-04
Candidate_division_TM7	6.08E-05	1.65E-05
Verrucomicrobia	0	1.70E-05

ì8. Faecal bacterial composition at the genus level between neonatal jaundice infants (NJI) and non-NJI at 0

ID	non-NJI	NJI
Escherichia-Shigella	0.4747515	0.582423667
Parabacteroides	0.103485833	0.040506333
Klebsiella	0.009423333	0.127204333
Clostridium_sensu_stricto_1	0.116582667	0.001411833
Streptococcus	0.047000333	0.0593175
Halomonas	0.047148167	0.017924
Bifidobacterium	0.019329833	0.041989167
Bacteroides	0.0063655	0.0327845
Enterococcus	0.021015167	0.003172
Pelagibacterium	0.017198667	0.006557333
Acinetobacter	0.020856833	0.001042833
Salmonella	3.63E-04	0.019819167
Aliihoeflea	0.0117575	0.003643667
Gemella	0.014703167	7.50E-06
Enterobacteriaceae_unclassified	3.66E-04	0.014121833
Staphylococcus	0.005335	0.0088765
Xanthomonadaceae_uncultured	0.007033333	0.002027
Corynebacterium	0.0074035	5.40E-04
Citrobacter	6.66E-04	0.0069805
Fusobacterium	2.59E-04	0.005737167
Enhydrobacter	0.005748833	1.13E-05
Enterobacter	5.39E-04	0.005015167
Veillonella	0.003238833	0.002184833
Ralstonia	0.003451667	7.96E-04
Arthrobacter	0.002500833	8.08E-04
Hyphomonadaceae_norank	0.001937667	8.82E-04
Erysipelotrichaceae_incertae_sedis	3.34E-04	0.002189
Kocuria	0.002044	2.22E-04
Phyllobacteriaceae_unclassified	0.001696167	5.54E-04
Porphyromonas	0.001968667	7.50E-06
Tepidimonas	0.0018815	0

Others	0.043614833	0.011243667

Table S9. Difference of fecal microbial communities at the genus level between neonatal jaundice infants (NJI) and non-NJI at 0 months

ID	non-NJI-mean	non-NJI-se	NJI-mean	NJI-se	p-value	Sig_mark	q-value
Escherichia-Shigella	0.4747515	0.171981943	0.582423667	0.157541655	0.484848485		0.75151515
Parabacteroides	0.103485833	0.100932957	0.040506333	0.040037358	0.17965368		0.75151515
Klebsiella	0.009423333	0.005859837	0.127204333	0.076936478	0.484848485		0.75151515
Clostridium_sensu_stricto_1	0.116582667	0.116219512	0.001411833	8.76E-04	0.297106984		0.75151515
Streptococcus	0.047000333	0.019031006	0.0593175	0.057830146	0.093073593		0.75151515
Halomonas	0.047148167	0.047133368	0.017924	0.008101741	0.65530639		0.86692641
Bifidobacterium	0.019329833	0.007644096	0.041989167	0.032917341	0.699134199		0.86692641
Bacteroides	0.0063655	0.00196412	0.0327845	0.032238277	0.064935065		0.75151515
Enterococcus	0.021015167	0.018330329	0.003172	0.002994654	0.22649054		0.75151515
Pelagibacterium	0.017198667	0.017198667	0.006557333	0.002952917	0.445976175		0.75151515
Acinetobacter	0.020856833	0.014892829	0.001042833	7.34E-04	1		1
Salmonella	3.63E-04	1.65E-04	0.019819167	0.019662744	0.57450381		0.8095281
Aliihoeflea	0.0117575	0.0117575	0.003643667	0.001643763	0.445976175		0.75151515
Gemella	0.014703167	0.014639975	7.50E-06	7.50E-06	0.016711445	*	0.51805478
Enterobacteriaceae_unclassified	3.66E-04	2.22E-04	0.014121833	0.013044723	0.521109983		0.76925759
Staphylococcus	0.005335	0.003064079	0.0088765	0.00816748	0.393939394		0.75151515
Xanthomonadaceae_uncultured	0.007033333	0.007033333	0.002027	9.24E-04	0.445976175		0.75151515
Corynebacterium	0.0074035	0.00727808	5.40E-04	4.68E-04	0.803315625		0.92869756
Citrobacter	6.66E-04	1.35E-04	0.0069805	0.006748271	0.30952381		0.75151515
Fusobacterium	2.59E-04	2.55E-04	0.005737167	0.005686233	0.266553869		0.75151515
Enhydrobacter	0.005748833	0.005675088	1.13E-05	1.13E-05	0.252951123		0.75151515
Enterobacter	5.39E-04	3.15E-04	0.005015167	0.003150206	0.935736281		1
Veillonella	0.003238833	0.001810637	0.002184833	0.002085137	0.132034632		0.75151515
Ralstonia	0.003451667	0.003222037	7.96E-04	3.29E-04	0.808865613		0.92869756
Arthrobacter	0.002500833	0.001820493	8.08E-04	2.86E-04	0.688403863		0.86692641
Hyphomonadaceae_norank	0.001937667	0.001937667	8.82E-04	4.22E-04	0.445976175		0.75151515
Erysipelotrichaceae_incertae_sedis	3.34E-04	1.03E-04	0.002189	0.002179614	0.158147096		0.75151515
Kocuria	0.002044	0.001922094	2.22E-04	1.78E-04	0.928858961		1
Phyllobacteriaceae_unclassified	0.001696167	0.001696167	5.54E-04	2.51E-04	0.445976175		0.75151515
Porphyromonas	0.001968667	0.001968667	7.50E-06	7.50E-06	1		1
Tepidimonas	0.0018815	0.0018815	0	0	0.404656762		0.75151515

Table S10. Detailed data for linear discriminant analysis (LDA) of the faecal microbial OTUs between neonatal jaundice infants (NJI) and non-NJI at 0 months

Biomaker_names	Logarithm value	Groups	LDA_value	P_value
d__Bacteria.p__Firmicutes.c__Negativicutes.o__Selenomonadales.f__Acidaminococcaceae	1.701855693			-
d__Bacteria.p__Proteobacteria.c__Betaproteobacteria.o__Burkholderiales.f__Comamonadaceae.g__Delftia	1.550228353			-
d__Bacteria.p__Proteobacteria.c__Deltaproteobacteria	1.034762106			-
d__Bacteria.p__Firmicutes.c__Clostridia.o__Clostridiales.f__Lachnospiraceae.g__Blautia	2.643945913	non-NJI	2.522877	0.00639
d__Bacteria.p__Proteobacteria.c__Gammaproteobacteria.o__Enterobacteriales	5.879302651			-
d__Bacteria.p__Firmicutes.c__Bacilli.o__Bacillales	4.322732562			-
d__Bacteria.p__Proteobacteria.c__Gammaproteobacteria.o__Oceanospirillales.f__Halomonadaceae.g__Halomonas	4.67346481			-
d__Bacteria.p__Bacteroidetes	5.060824963			-
d__Bacteria.p__Proteobacteria.c__Alphaproteobacteria.o__Rhizobiales.f__Rhizobiaceae.g__Rhizobium	2.839687497			-
d__Bacteria.p__Proteobacteria.c__Alphaproteobacteria.o__Sphingomonadales.f__Sphingomonadaceae	3.41808022			-
d__Bacteria.p__Bacteroidetes.c__Bacteroidia.o__Bacteroidales.f__Rikenellaceae.g__RC9_gut_group	0			-
d__Bacteria.p__Bacteroidetes.c__Bacteroidia.o__Bacteroidales.f__Bacteroidaceae	4.515668565			-
d__Bacteria.p__Actinobacteria.c__Actinobacteria.o__Actinomycetales.f__Actinomycetaceae	2.559906625			-
d__Bacteria.p__Firmicutes.c__Clostridia.o__Clostridiales.f__Lachnospiraceae.g__Coprococcus	1.507406059			-
d__Bacteria.p__Proteobacteria.c__Gammaproteobacteria.o__Xanthomonadales.f__Xanthomonadaceae.g__Stenotrophomonas	2.170261715			-
d__Bacteria.p__Proteobacteria.c__Gammaproteobacteria.o__Oceanospirillales.f__Halomonadaceae	4.67346481			-
d__Bacteria.p__Proteobacteria.c__Betaproteobacteria.o__Neisseriales.f__Neisseriaceae.g__Kingella	0			-
d__Bacteria.p__Firmicutes.c__Clostridia.o__Clostridiales.f__Lachnospiraceae.g__Stomatobaculum	0			-
d__Bacteria.p__Proteobacteria.c__Betaproteobacteria.o__Rhodocyclales.f__Rhodocyclaceae	2.436162647			-
d__Bacteria.p__Proteobacteria.c__Epsilonproteobacteria.o__Campylobacterales	0			-
d__Bacteria.p__Firmicutes.c__Clostridia.o__Clostridiales.f__Eubacteriaceae	0			-
d__Bacteria.p__Firmicutes.c__Erysipelotrichia	3.44868056			-
d__Bacteria.p__Actinobacteria.c__Actinobacteria.o__Micrococcales.f__Micrococcaceae.g__Rothia	2.759416007			-
d__Bacteria.p__Verrucomicrobia.c__Verrucomicrobiae	1.230448921			-
d__Bacteria.p__Proteobacteria.c__Alphaproteobacteria.o__Caulobacterales.f__Caulobacteraceae	2.864412187			-
d__Bacteria.p__Proteobacteria.c__Alphaproteobacteria.o__Rhizobiales.f__Hyphomicrobiaceae.g__Pelagibacterium	4.235494779			-
d__Bacteria.p__Firmicutes.c__Clostridia.o__Clostridiales.f__Lachnospiraceae	3.483896209	non-NJI	3.062388	0.01631
d__Bacteria.p__Firmicutes.c__Erysipelotrichia.o__Erysipelotrichales	3.44868056			-
d__Bacteria.p__Firmicutes.c__Negativicutes.o__Selenomonadales	3.669719679			-
d__Bacteria.p__Proteobacteria.c__Betaproteobacteria.o__Burkholderiales.f__Comamonadaceae.g__Curvibacter	1.765916794			-

Table S10. Detailed data for linear discriminant analysis (LDA) of the faecal microbial OTUs between neonatal jaundice infants (NJI) and non-NJI at 0 months (continued)

Biomaker_names	Logarithm value	Groups	LDA_value	P_value
d__Bacteria.p__Proteobacteria.c__Betaproteobacteria.o__Burkholderiales.f__Burkholderiaceae.g__Ralstonia	3.538028849			-
d__Bacteria.p__Actinobacteria.c__Actinobacteria.o__Micrococcales.f__Cellulomonadaceae	0			-
d__Bacteria.p__Proteobacteria.c__Gammaproteobacteria.o__Pasteurellales.f__Pasteurellaceae	3.480414203	non-NJI	3.161679	0.02472
d__Bacteria.p__Proteobacteria.c__Gammaproteobacteria.o__Pasteurellales	3.480414203	non-NJI	3.161679	0.02472
d__Bacteria.p__Proteobacteria.c__Alphaproteobacteria.o__Rhodobacterales	2.958006125			-
d__Bacteria.p__Firmicutes.c__Clostridia.o__Clostridiales.f__Lachnospiraceae.g__Dorea	1.828230115	non-NJI	2.906617	0.04951
d__Bacteria.p__Bacteroidetes.c__Bacteroidia.o__Bacteroidales.f__Porphyromonadaceae.g__Coprobacter	0			-
d__Bacteria.p__Firmicutes.c__Clostridia.o__Clostridiales.f__Ruminococcaceae.g__Faecalibacterium	2.748575617			-
d__Bacteria.p__Proteobacteria.c__Gammaproteobacteria.o__Enterobacteriales.f__Enterobacteriaceae.g__Raoultella	3.152594078			-
d__Bacteria.p__Verrucomicrobia.c__Verrucomicrobiae.o__Verrucomicrobiales.f__Verrucomicrobiaceae.g__Akkermansia	1.230448921			-
d__Bacteria.p__Actinobacteria.c__Actinobacteria.o__Micrococcales.f__Micrococcaceae.g__Micrococcus	2.950608225			-
d__Bacteria.p__Proteobacteria.c__Betaproteobacteria.o__Burkholderiales.f__Comamonadaceae.g__Comamonas	1.701855693			-
d__Bacteria.p__Proteobacteria.c__Gammaproteobacteria.o__Pasteurellales.f__Pasteurellaceae.g__Haemophilus	3.480414203	non-NJI	3.17163	0.02472
d__Bacteria.p__Proteobacteria.c__Alphaproteobacteria.o__Rhizobiales.f__Brucellaceae	1.774516966			-
d__Bacteria.p__Proteobacteria.c__Gammaproteobacteria.o__Pseudomonadales.f__Pseudomonadaceae	2.808661019			-
d__Bacteria.p__Fusobacteria.c__Fusobacteriia.o__Fusobacteriales	3.758697466			-
d__Bacteria.p__Proteobacteria.c__Alphaproteobacteria.o__Sphingomonadales.f__Sphingomonadaceae.g__Novosphingobium	2.816020229			-
d__Bacteria.p__Firmicutes.c__Bacilli.o__Bacillales.f__Staphylococcaceae	3.948241757			-
d__Bacteria.p__Firmicutes.c__Bacilli.o__Lactobacillales.f__Streptococcaceae.g__Streptococcus	4.773182839			-
d__Bacteria.p__Firmicutes.c__Clostridia.o__Clostridiales	5.098560666			-
d__Bacteria.p__Proteobacteria.c__Betaproteobacteria.o__Burkholderiales.f__Comamonadaceae.g__Aquabacterium	3.195392218			-
d__Bacteria.p__Actinobacteria.c__Actinobacteria.o__Bifidobacteriales.f__Bifidobacteriaceae.g__Bifidobacterium	4.623137256			-
d__Bacteria.p__Proteobacteria.c__Deltaproteobacteria.o__Desulfovibrionales.f__Desulfovibrionaceae.g__Bilophila	1.034762106			-
d__Bacteria.p__Proteobacteria.c__Gammaproteobacteria.o__Xanthomonadales.f__Xanthomonadaceae	3.856205086			-
d__Bacteria.p__Firmicutes.c__Bacilli	4.962524808			-
d__Bacteria.p__Firmicutes.c__Bacilli.o__Lactobacillales.f__Aerococcaceae	2.561498907			-
d__Bacteria.p__Firmicutes.c__Bacilli.o__Lactobacillales.f__Leuconostocaceae.g__Weissella	1.263241435			-
d__Bacteria.p__Fusobacteria	3.758697466			-
d__Bacteria.p__Actinobacteria.c__Actinobacteria.o__Micrococcales.f__Micrococcaceae	3.857613892			-
d__Bacteria.p__Firmicutes.c__Clostridia.o__Clostridiales.f__Family_XIII	0			-

Table S10. Detailed data for linear discriminant analysis (LDA) of the faecal microbial OTUs between neonatal jaundice infants (NJI) and non-NJI at 0 months (continued)

Biomaker_names	Logarithm value	Groups	LDA_value	P_value
d__Bacteria.p__Actinobacteria.c__Actinobacteria.o__Micrococcales.f__Brevibacteriaceae	3.155892469			-
d__Bacteria.p__Actinobacteria.c__Actinobacteria	4.653096686			-
d__Bacteria.p__Proteobacteria.c__Gammaproteobacteria.o__Enterobacteriales.f__Enterobacteriaceae.g__Proteus	1.230448921			-
d__Bacteria.p__Firmicutes.c__Erysipelotrichia.o__Erysipelotrichales.f__Erysipelotrichaceae.g__Turicibacter	0			-
d__Bacteria.p__Proteobacteria.c__Betaproteobacteria.o__Burkholderiales.f__Alcaligenaceae.g__Sutterella	1.352182518			-
d__Bacteria.p__Firmicutes.c__Negativicutes	3.669719679			-
d__Bacteria.p__Actinobacteria.c__Actinobacteria.o__Micrococcales.f__Dermabacteraceae.g__Dermabacter	2.015639134			-
d__Bacteria.p__Firmicutes.c__Bacilli.o__Lactobacillales.f__Streptococcaceae.g__Lactococcus	2.285557309			-
d__Bacteria.p__Proteobacteria.c__Betaproteobacteria.o__Burkholderiales.f__Comamonadaceae	3.754450271			-
d__Bacteria.p__Firmicutes.c__Bacilli.o__Lactobacillales.f__Leuconostocaceae	1.50965048			-
d__Bacteria.p__Firmicutes.c__Clostridia.o__Clostridiales.f__vadinBB60	0			-
d__Bacteria.p__Firmicutes.c__Bacilli.o__Bacillales.f__Bacillaceae.g__Geobacillus	1.641804498			-
d__Bacteria.p__Cyanobacteria.c__Cyanobacteria.o__norank.f__norank	2.346678899			-
d__Bacteria.p__Proteobacteria.c__Alphaproteobacteria.o__Sphingomonadales.f__Sphingomonadaceae.g__Sphingomonas	3.247891471			-
d__Bacteria.p__Proteobacteria.c__Alphaproteobacteria.o__Rhizobiales.f__Hyphomicrobiaceae	4.235494779			-
d__Bacteria.p__Bacteroidetes.c__Sphingobacteriia.o__Sphingobacteriales	2.581114914			-
d__Bacteria.p__Proteobacteria.c__Alphaproteobacteria.o__Caulobacterales.f__Hyphomonadaceae	3.287279068			-
d__Bacteria.p__Actinobacteria.c__Actinobacteria.o__Corynebacteriales.f__Dietziaceae.g__Dietzia	2.954162077			-
d__Bacteria.p__Proteobacteria.c__Gammaproteobacteria.o__Enterobacteriales.f__Enterobacteriaceae.g__Salmonella	4.29708539			-
d__Bacteria.p__Firmicutes.c__Clostridia.o__Clostridiales.f__Peptostreptococcaceae.g__Peptostreptococcus	0			-
d__Bacteria.p__Bacteroidetes.c__Bacteroidia.o__Bacteroidales.f__Prevotellaceae.g__Alloprevotella	1.643452676			-
d__Bacteria.p__Fusobacteria.c__Fusobacteriia.o__Fusobacteriales.f__Fusobacteriaceae.g__Fusobacterium	3.758697466			-
d__Bacteria.p__Bacteroidetes.c__Bacteroidia.o__Bacteroidales.f__Prevotellaceae.g__Paraprevotella	1.124938737			-
d__Bacteria.p__Firmicutes.c__Clostridia.o__Clostridiales.f__Defluviitaleaceae	1.736396502			-
d__Bacteria.p__Proteobacteria.c__Deltaproteobacteria.o__Desulfovibrionales	1.034762106			-
d__Bacteria.p__Firmicutes.c__Clostridia.o__Clostridiales.f__Lachnospiraceae.g__Pseudobutyrivibrio	2.582252804	non-NJI	2.461767	0.01612
d__Bacteria.p__Firmicutes.c__Negativicutes.o__Selenomonadales.f__Veillonellaceae.g__Megasphaera	2.171238756			-
d__Bacteria.p__Firmicutes.c__Bacilli.o__Lactobacillales.f__Carnobacteriaceae.g__Dolosigranulum	2.408239965			-
d__Bacteria.p__Cyanobacteria	2.207724107			-
d__Bacteria.p__Firmicutes.c__Clostridia.o__Clostridiales.f__Ruminococcaceae.g__Anaerotruncus	0.636822098			-

Table S10. Detailed data for linear discriminant analysis (LDA) of the faecal microbial OTUs between neonatal jaundice infants (NJI) and non-NJI at 0 months (continued)

Biomaker_names	Logarithm value	Groups	LDA_value	P_value
d__Bacteria.p__Proteobacteria.c__Alphaproteobacteria.o__Rhizobiales.f__Rhizobiaceae	2.839687497			-
d__Bacteria.p__Firmicutes.c__Clostridia.o__Clostridiales.f__Ruminococcaceae.g__Subdoligranulum	2.144574208			-
d__Bacteria.p__Firmicutes.c__Clostridia.o__Clostridiales.f__Christensenellaceae	2.135662602	non-NJI	2.36557	0.02223
d__Bacteria.p__Actinobacteria.c__Actinobacteria.o__Actinomycetales.f__Actinomycetaceae.g__Varibaculum	0			-
d__Bacteria.p__Proteobacteria.c__Gammaproteobacteria.o__Pasteurellales.f__Pasteurellaceae.g__Pasteurella	0			-
d__Bacteria.p__Proteobacteria.c__Gammaproteobacteria.o__Enterobacteriales.f__Enterobacteriaceae.g__Klebsiella	5.104501906			-
d__Bacteria.p__Firmicutes.c__Negativicutes.o__Selenomonadales.f__Veillonellaceae.g__Anaeroglobus	0			-
d__Bacteria.p__Proteobacteria.c__Alphaproteobacteria	4.577012816			-
d__Bacteria.p__Proteobacteria	5.899783125			-
d__Bacteria.p__Proteobacteria.c__Gammaproteobacteria.o__Pseudomonadales.f__Moraxellaceae.g__Psychrobacter	2.409651388			-
d__Bacteria.p__Proteobacteria.c__Betaproteobacteria.o__Burkholderiales.f__Alcaligenaceae.g__Parasutterella	2.237208505			-
d__Bacteria.p__Firmicutes.c__Bacilli.o__Bacillales.f__Bacillaceae.g__Anoxybacillus	2.696210726			-
d__Bacteria.p__Actinobacteria.c__Actinobacteria.o__Corynebacteriales.f__Nocardiaceae	1.752048448			-
d__Bacteria.p__Actinobacteria.c__Actinobacteria.o__Micrococcales.f__Micrococcaceae.g__Nesterenkonia	3.076519082			-
d__Bacteria.p__Actinobacteria.c__Actinobacteria.o__Bifidobacteriales.f__Bifidobacteriaceae.g__Gardnerella	0			-
d__Bacteria.p__Firmicutes.c__Clostridia.o__Clostridiales.f__Ruminococcaceae.g__Intestinimonas	0			-
d__Bacteria.p__Bacteroidetes.c__Flavobacteriia.o__Flavobacteriales.f__Flavobacteriaceae	2.730243783			-
d__Bacteria.p__Cyanobacteria.c__Cyanobacteria	2.207724107			-
d__Bacteria.p__Firmicutes.c__Clostridia.o__Clostridiales.f__Family_XI	4.249882902	non-NJI	3.995101	0.00988
d__Bacteria.p__Firmicutes.c__Negativicutes.o__Selenomonadales.f__Veillonellaceae.g__Negativicoccus	0			-
d__Bacteria.p__Proteobacteria.c__Alphaproteobacteria.o__Rhizobiales	4.497627554			-
d__Bacteria.p__Firmicutes.c__Bacilli.o__Bacillales.f__Paenibacillaceae.g__Brevibacillus	2.08754481			-
d__Bacteria.p__Actinobacteria.c__Actinobacteria.o__Micrococcales.f__Dermabacteraceae.g__Brachybacterium	2.175608441			-
d__Bacteria.p__Candidate_division_TM7	1.784141614			-
d__Bacteria.p__Actinobacteria.c__Actinobacteria.o__Bifidobacteriales.f__Bifidobacteriaceae.g__Scardovia	1.345700391			-
d__Bacteria.p__Firmicutes.c__Negativicutes.o__Selenomonadales.f__Veillonellaceae	3.665017825			-
d__Bacteria.p__Actinobacteria.c__Actinobacteria.o__Rubrobacterales.f__Rubrobacteriaceae	2.363925211			-
d__Bacteria.p__Firmicutes.c__Bacilli.o__Bacillales.f__Paenibacillaceae	2.08754481			-
d__Bacteria.p__Firmicutes.c__Clostridia.o__Clostridiales.f__Clostridiaceae_1.g__Clostridium_sensu_stricto_18	0			-
d__Bacteria.p__Actinobacteria.c__Actinobacteria.o__Micrococcales.f__Brevibacteriaceae.g__Brevibacterium	3.155892469			-

Table S10. Detailed data for linear discriminant analysis (LDA) of the faecal microbial OTUs between neonatal jaundice infants (NJI) and non-NJI at 0 months (continued)

Biomaker_names	Logarithm value	Groups	LDA_value	P_value
d__Bacteria.p__Firmicutes.c__Clostridia.o__Clostridiales.f__Lachnospiraceae.g__uncultured	3.922613022			-
d__Bacteria.p__Firmicutes.c__Bacilli.o__Lactobacillales.f__Leuconostocaceae.g__Leuconostoc	1.50965048			-
d__Bacteria.p__Firmicutes.c__Negativicutes.o__Selenomonadales.f__Veillonellaceae.g__Megamonas	3.076154791	non-NJI	2.856235	0.03409
d__Bacteria.p__Firmicutes.c__Bacilli.o__Bacillales.f__Bacillaceae	2.732929595			-
d__Bacteria.p__Proteobacteria.c__Deltaproteobacteria.o__Desulfovibrionales.f__Desulfovibrionaceae	1.034762106			-
d__Bacteria.p__Actinobacteria.c__Actinobacteria.o__Coriobacteriales	3.196682705			-
d__Bacteria.p__Firmicutes.c__Bacilli.o__Lactobacillales.f__Aerococcaceae.g__Facklamia	0			-
d__Bacteria.p__Proteobacteria.c__Alphaproteobacteria.o__Caulobacterales.f__Caulobacteraceae.g__Brevundimonas	2.845201431			-
d__Bacteria.p__Bacteroidetes.c__Bacteroidia.o__Bacteroidales.f__Bacteroidaceae.g__Bacteroides	4.515668565			-
d__Bacteria.p__Proteobacteria.c__Gammaproteobacteria.o__Enterobacteriales.f__Enterobacteriaceae	5.879302651			-
d__Bacteria.p__Bacteroidetes.c__Bacteroidia.o__Bacteroidales.f__Porphyromonadaceae.g__Dysgonomonas	0.753327667			-
d__Bacteria.p__Firmicutes.c__Bacilli.o__Bacillales.f__Bacillaceae.g__Bacillus	0			-
d__Bacteria.p__Actinobacteria.c__Actinobacteria.o__Actinomycetales.f__Actinomycetaceae.g__Actinobaculum	0			-
d__Bacteria.p__Actinobacteria.c__Actinobacteria.o__Rubrobacterales.f__Rubrobacteriaceae.g__Rubrobacter	2.363925211			-
d__Bacteria.p__Proteobacteria.c__Alphaproteobacteria.o__Rhizobiales.f__Brucellaceae.g__Pseudochrobactrum	1.774516966			-
d__Bacteria.p__Actinobacteria.c__Actinobacteria.o__Micrococcales.f__Intrasporangiaceae	2.36298484			-
d__Bacteria.p__Proteobacteria.c__Gammaproteobacteria.o__Enterobacteriales.f__Enterobacteriaceae.g__Citrobacter	3.843886531			-
d__Bacteria.p__Firmicutes.c__Erysipelotrichia.o__Erysipelotrichales.f__Erysipelotrichaceae.g__Holdemania	0			-
d__Bacteria.p__Bacteroidetes.c__Bacteroidia.o__Bacteroidales.f__S24_7	2.122761817			-
d__Bacteria.p__Proteobacteria.c__Alphaproteobacteria.o__Sphingomonadales.f__Sphingomonadaceae.g__Sphingopyxis	2.089905111			-
d__Bacteria	6			-
d__Bacteria.p__Firmicutes.c__Erysipelotrichia.o__Erysipelotrichales.f__Erysipelotrichaceae.g__Coprobacillus	2.786278077			-
d__Bacteria.p__Actinobacteria.c__Actinobacteria.o__Micrococcales.f__Micrococcaceae.g__Arthrobacter	3.398084749			-
d__Bacteria.p__Actinobacteria.c__Actinobacteria.o__Propionibacteriales.f__Nocardioidaceae	2.53571597			-
d__Bacteria.p__Bacteroidetes.c__Bacteroidia	5.057343369			-
d__Bacteria.p__Bacteroidetes.c__Sphingobacteriia.o__Sphingobacteriales.f__Sphingobacteriaceae.g__Sphingobacterium	2.564073979			-
d__Bacteria.p__Verrucomicrobia	1.230448921			-
d__Bacteria.p__Firmicutes.c__Clostridia.o__Clostridiales.f__Peptostreptococcaceae	3.170848204			-
d__Bacteria.p__Firmicutes.c__Clostridia.o__Clostridiales.f__Lachnospiraceae.g__Roseburia	1.648360011			-
d__Bacteria.p__Firmicutes.c__Clostridia.o__Clostridiales.f__Lachnospiraceae.g__Epulopiscium	0			-

Table S10. Detailed data for linear discriminant analysis (LDA) of the faecal microbial OTUs between neonatal jaundice infants (NJI) and non-NJI at 0 months (continued)

Biomaker_names	Logarithm value	Groups	LDA_value	P_value
d__Bacteria.p__Proteobacteria.c__Betaproteobacteria.o__Burkholderiales.f__Alcaligenaceae	2.42894429			-
d__Bacteria.p__Firmicutes.c__Bacilli.o__Bacillales.f__Staphylococcaceae.g__Macrococcus	2.510097975			-
d__Bacteria.p__Actinobacteria.c__Actinobacteria.o__Coriobacteriales.f__Coriobacteriaceae.g__Adlercreutzia	0			-
d__Bacteria.p__Proteobacteria.c__Epsilonproteobacteria.o__Campylobacterales.f__Campylobacteraceae	0			-
d__Bacteria.p__Actinobacteria.c__Actinobacteria.o__Corynebacteriales.f__Corynebacteriaceae.g__Corynebacterium	3.869437081			-
d__Bacteria.p__Firmicutes.c__Clostridia.o__Clostridiales.f__Ruminococcaceae.g__Butyricicoccus	1.014240439			-
d__Bacteria.p__Proteobacteria.c__Gammaproteobacteria.o__Pseudomonadales	4.440189908			-
d__Bacteria.p__Firmicutes.c__Bacilli.o__Lactobacillales.f__Carnobacteriaceae.g__Desemzia	1.708987125			-
d__Bacteria.p__Firmicutes.c__Clostridia.o__Clostridiales.f__Ruminococcaceae	3.040668257			-
d__Bacteria.p__Firmicutes.c__Clostridia.o__Clostridiales.f__Lachnospiraceae.g__Howardella	0			-
d__Bacteria.p__Proteobacteria.c__Alphaproteobacteria.o__Rhizobiales.f__Phyllobacteriaceae.g__Aliihoeflea	4.070314987			-
d__Bacteria.p__Firmicutes.c__Negativicutes.o__Selenomonadales.f__Veillonellaceae.g__Veillonella	3.5103886			-
d__Bacteria.p__Firmicutes.c__Clostridia.o__Clostridiales.f__Eubacteriaceae.g__Pseudoramibacter	0			-
d__Bacteria.p__Proteobacteria.c__Gammaproteobacteria.o__Pseudomonadales.f__Moraxellaceae.g__Acinetobacter	4.319248371			-
d__Bacteria.p__Bacteroidetes.c__Bacteroidia.o__Bacteroidales.f__Rikenellaceae.g__Alistipes	1.921686475			-
d__Bacteria.p__Proteobacteria.c__Gammaproteobacteria.o__Pseudomonadales.f__Moraxellaceae.g__Moraxella	1.135662602			-
d__Bacteria.p__Firmicutes.c__Bacilli.o__Lactobacillales.f__Lactobacillaceae.g__Pediococcus	1.908485019			-
d__Bacteria.p__Bacteroidetes.c__Bacteroidia.o__Bacteroidales.f__Porphyromonadaceae.g__Macellibacteroides	0			-
d__Bacteria.p__Bacteroidetes.c__Sphingobacteriia	2.581114914			-
d__Bacteria.p__Actinobacteria.c__Actinobacteria.o__Bifidobacteriales.f__Bifidobacteriaceae	4.623347513			-
d__Bacteria.p__Actinobacteria.c__Actinobacteria.o__Micrococcales.f__Cellulomonadaceae.g__Tropheryma	0			-
d__Bacteria.p__Actinobacteria.c__Actinobacteria.o__Corynebacteriales.f__Corynebacteriaceae	3.869984236			-
d__Bacteria.p__Firmicutes.c__Clostridia.o__Clostridiales.f__Lachnospiraceae.g__Anaerostipes	1.486666573			-
d__Bacteria.p__Firmicutes.c__Negativicutes.o__Selenomonadales.f__Acidaminococcaceae.g__Acidaminococcus	0			-
d__Bacteria.p__Actinobacteria.c__Actinobacteria.o__Pseudonocardiales	2.226599905			-
d__Bacteria.p__Actinobacteria.c__Actinobacteria.o__Micrococcales.f__Micrococcaceae.g__Kocuria	3.310480891			-
d__Bacteria.p__Actinobacteria	4.653096686			-
d__Bacteria.p__Bacteroidetes.c__Bacteroidia.o__Bacteroidales.f__Porphyromonadaceae.g__Porphyromonas	3.294172188			-
d__Bacteria.p__Actinobacteria.c__Actinobacteria.o__Coriobacteriales.f__Coriobacteriaceae.g__Collinsella	2.763178327			-
d__Bacteria.p__Actinobacteria.c__Actinobacteria.o__Micrococcales.f__Dermacoccaceae.g__Kytococcus	3.033490771			-

Table S10. Detailed data for linear discriminant analysis (LDA) of the faecal microbial OTUs between neonatal jaundice infants (NJI) and non-NJI at 0 months (continued)

Biomaker_names	Logarithm value	Groups	LDA_value	P_value
d__Bacteria.p__Bacteroidetes.c__Bacteroidia.o__Bacteroidales.f__S24_7.g__norank	3.383635868			-
d__Bacteria.p__Bacteroidetes.c__Flavobacteriia.o__Flavobacteriales	2.730243783			-
d__Bacteria.p__Firmicutes.c__Bacilli.o__Lactobacillales.f__Streptococcaceae	4.773560953			-
d__Bacteria.p__Actinobacteria.c__Actinobacteria.o__Propionibacteriales.f__Propionibacteriaceae.g__Propionibacterium	3.123797215			-
d__Bacteria.p__Proteobacteria.c__Gammaproteobacteria.o__Oceanospirillales	4.67346481			-
d__Bacteria.p__Proteobacteria.c__Alphaproteobacteria.o__Caulobacterales.f__Caulobacteraceae.g__Caulobacter	1.500602351			-
d__Bacteria.p__Firmicutes.c__Negativicutes.o__Selenomonadales.f__Acidaminococcaceae.g__Phascolarctobacterium	1.701855693			-
d__Bacteria.p__Actinobacteria.c__Actinobacteria.o__Coriobacteriales.f__Coriobacteriaceae.g__Gordonibacter	1.079181246			-
d__Bacteria.p__Actinobacteria.c__Actinobacteria.o__Corynebacteriales.f__Dietziaceae	2.954162077			-
d__Bacteria.p__Firmicutes.c__Clostridia.o__Clostridiales.f__Family_XI.g__Finegoldia	2.916804752			-
d__Bacteria.p__Proteobacteria.c__Gammaproteobacteria.o__Pasteurellales.f__Pasteurellaceae.g__Actinobacillus	1.833572058			-
d__Bacteria.p__Proteobacteria.c__Epsilonproteobacteria	0			-
d__Bacteria.p__Proteobacteria.c__Betaproteobacteria.o__Burkholderiales.f__Burkholderiaceae	3.566241023			-
d__Bacteria.p__Bacteroidetes.c__Bacteroidia.o__Bacteroidales.f__Rikenellaceae	1.921686475			-
d__Bacteria.p__Proteobacteria.c__Alphaproteobacteria.o__Rhizobiales.f__Methylobacteriaceae.g__Methylobacterium	1.096910013			-
d__Bacteria.p__Proteobacteria.c__Gammaproteobacteria.o__Pseudomonadales.f__Moraxellaceae	4.429367326			-
d__Bacteria.p__Bacteroidetes.c__Flavobacteriia.o__Flavobacteriales.f__Flavobacteriaceae.g__Empedobacter	0			-
d__Bacteria.p__Proteobacteria.c__Gammaproteobacteria	5.891351398			-
d__Bacteria.p__Proteobacteria.c__Epsilonproteobacteria.o__Campylobacterales.f__Campylobacteraceae.g__Campylobacter	0			-
d__Bacteria.p__Proteobacteria.c__Betaproteobacteria.o__Neisseriales	2.866779458			-
d__Bacteria.p__Firmicutes.c__Clostridia.o__Clostridiales.f__Family_XI.g__Parvimonas	1.673635185			-
d__Bacteria.p__Proteobacteria.c__Betaproteobacteria	4.031435396			-
d__Bacteria.p__Firmicutes.c__Bacilli.o__Bacillales.f__Family_XI.g__Gemella	4.16741088	non-NJI	3.920606	0.0132
d__Bacteria.p__Bacteroidetes.c__Bacteroidia.o__Bacteroidales.f__Porphyromonadaceae.g__Odoribacter	0			-
d__Bacteria.p__Firmicutes.c__Clostridia.o__Clostridiales.f__Lachnospiraceae.g__Incertae_Sedis	3.557607443			-
d__Bacteria.p__Fusobacteria.c__Fusobacteriia.o__Fusobacteriales.f__Fusobacteriaceae	3.758697466			-
d__Bacteria.p__Firmicutes.c__Bacilli.o__Lactobacillales.f__Carnobacteriaceae.g__Carnobacterium	1.423245874			-
d__Bacteria.p__Actinobacteria.c__Actinobacteria.o__Micrococcales.f__Dermacoccaceae	3.033490771			-
d__Bacteria.p__Firmicutes.c__Bacilli.o__Lactobacillales.f__Enterococcaceae.g__Enterococcus	4.322532839			-
d__Bacteria.p__Proteobacteria.c__Alphaproteobacteria.o__Rhizobiales.f__Rhizobiales_Incertae_Sedis.g__Agaricicola	1.541995036			-

Table S10. Detailed data for linear discriminant analysis (LDA) of the faecal microbial OTUs between neonatal jaundice infants (NJI) and non-NJI at 0 months (continued)

Biomaker_names	Logarithm value	Groups	LDA_value	P_value
d__Bacteria.p__Proteobacteria.c__Betaproteobacteria.o__Burkholderiales.f__Comamonadaceae.g__Pelomonas	3.109240969			-
d__Bacteria.p__Bacteroidetes.c__Flavobacteriia.o__Flavobacteriales.f__Flavobacteriaceae.g__Wautersiella	0			-
d__Bacteria.p__Proteobacteria.c__Gammaproteobacteria.o__Pseudomonadales.f__Moraxellaceae.g__Enhydrobacter	3.759579718			-
d__Bacteria.p__Firmicutes.c__Bacilli.o__Bacillales.f__Bacillaceae.g__Oceanobacillus	1.08517161			-
d__Bacteria.p__Proteobacteria.c__Alphaproteobacteria.o__Rhodobacterales.f__Rhodobacteraceae.g__Paracoccus	2.925655815			-
d__Bacteria.p__Actinobacteria.c__Actinobacteria.o__Propionibacteriales.f__Propionibacteriaceae	3.123797215			-
d__Bacteria.p__Fusobacteria.c__Fusobacteriia	3.758697466			-
d__Bacteria.p__Firmicutes.c__Clostridia.o__Clostridiales.f__Lachnospiraceae.g__Anaerosporobacter	1.190331698			-
d__Bacteria.p__Firmicutes	5.346988301			-
d__Bacteria.p__Actinobacteria.c__Actinobacteria.o__Bifidobacteriales.f__Bifidobacteriaceae.g__Parascardovia	0			-
d__Bacteria.p__Bacteroidetes.c__Bacteroidia.o__Bacteroidales.f__Prevotellaceae.g__Prevotella	3.312811826			-
d__Bacteria.p__Actinobacteria.c__Actinobacteria.o__Actinomycetales	2.559906625			-
d__Bacteria.p__Actinobacteria.c__Actinobacteria.o__Coriobacteriales.f__Coriobacteriaceae.g__Slackia	0			-
d__Bacteria.p__Proteobacteria.c__Gammaproteobacteria.o__Enterobacteriales.f__Enterobacteriaceae.g__Enterobacter	3.70028537			-
d__Bacteria.p__Actinobacteria.c__Actinobacteria.o__Propionibacteriales	3.223539204			-
d__Bacteria.p__Proteobacteria.c__Betaproteobacteria.o__Burkholderiales.f__Comamonadaceae.g__Tepidimonas	3.274504223			-
d__Bacteria.p__Firmicutes.c__Clostridia.o__Clostridiales.f__Clostridiaceae_1.g__Clostridium_sensu_stricto_1	5.066633985			-
d__Bacteria.p__Firmicutes.c__Clostridia.o__Clostridiales.f__Lachnospiraceae.g__Oribacterium	1.091080469			-
d__Bacteria.p__Actinobacteria.c__Actinobacteria.o__Micrococcales	4.014723498			-
d__Bacteria.p__Proteobacteria.c__Alphaproteobacteria.o__Rhodobacterales.f__Rhodobacteraceae.g__Rhodobacter	1.814025507			-
d__Bacteria.p__Bacteroidetes.c__Bacteroidia.o__Bacteroidales.f__Porphyromonadaceae.g__Parabacteroides	5.014880901			-
d__Bacteria.p__Firmicutes.c__Bacilli.o__Lactobacillales.f__Lactobacillaceae.g__Lactobacillus	3.086122446			-
d__Bacteria.p__Bacteroidetes.c__Bacteroidia.o__Bacteroidales.f__Prevotellaceae	3.31666913			-
d__Bacteria.p__Proteobacteria.c__Alphaproteobacteria.o__Rhizobiales.f__Rhizobiales_Incertae_Sedis	1.541995036			-
d__Bacteria.p__Firmicutes.c__Negativicutes.o__Selenomonadales.f__Veillonellaceae.g__Dialister	1.65481804			-
d__Bacteria.p__Proteobacteria.c__Alphaproteobacteria.o__Rickettsiales.f__mitochondria	2.04857127			-
d__Bacteria.p__Actinobacteria.c__Actinobacteria.o__Coriobacteriales.f__Coriobacteriaceae.g__Eggerthella	2.294098647			-
d__Bacteria.p__Proteobacteria.c__Betaproteobacteria.o__Rhodocyclales.f__Rhodocyclaceae.g__Methyloversatilis	1.74947865			-
d__Bacteria.p__Actinobacteria.c__Actinobacteria.o__Bifidobacteriales	4.623347513			-
d__Bacteria.p__Firmicutes.c__Erysipelotrichia.o__Erysipelotrichales.f__Erysipelotrichaceae.g__Catenibacterium	0			-

Table S10. Detailed data for linear discriminant analysis (LDA) of the faecal microbial OTUs between neonatal jaundice infants (NJI) and non-NJI at 0 months (continued)

Biomaker_names	Logarithm value	Groups	LDA_value	P_value
d__Bacteria.p__Firmicutes.c__Bacilli.o__Lactobacillales.f__Aerococcaceae.g__Aerococcus	2.383815366			-
d__Bacteria.p__Firmicutes.c__Clostridia.o__Clostridiales.f__Ruminococcaceae.g__Ruminococcus	2.067566768			-
d__Bacteria.p__Firmicutes.c__Clostridia.o__Clostridiales.f__Lachnospiraceae.g__Lachnospira	2.370451404			-
d__Bacteria.p__Proteobacteria.c__Gammaproteobacteria.o__Enterobacteriales.f__Enterobacteriaceae.g__Morganella	1.653212514			-
d__Bacteria.p__Bacteroidetes.c__Bacteroidia.o__Bacteroidales.f__Porphyromonadaceae.g__Barnesiella	0			-
d__Bacteria.p__Firmicutes.c__Clostridia.o__Clostridiales.f__Lachnospiraceae.g__Lachnoanaerobaculum	0.823908741			-
d__Bacteria.p__Cyanobacteria.c__Cyanobacteria.o__norank	2.346678899			-
d__Bacteria.p__Proteobacteria.c__Gammaproteobacteria.o__Enterobacteriales.f__Enterobacteriaceae.g__Escherichia_Shigella	5.765239014			-
d__Bacteria.p__Proteobacteria.c__Alphaproteobacteria.o__Sphingomonadales.f__Sphingomonadaceae.g__Sphingobium	1.853292519			-
d__Bacteria.p__Firmicutes.c__Bacilli.o__Lactobacillales.f__Carnobacteriaceae	2.941428607			-
d__Bacteria.p__Proteobacteria.c__Betaproteobacteria.o__Burkholderiales.f__Oxalobacteraceae.g__Herbaspirillum	2.036096345			-
d__Bacteria.p__Proteobacteria.c__Alphaproteobacteria.o__Rickettsiales	2.04857127			-
d__Bacteria.p__Actinobacteria.c__Actinobacteria.o__Corynebacteriales	3.922682217			-
d__Bacteria.p__Proteobacteria.c__Betaproteobacteria.o__Burkholderiales.f__Oxalobacteraceae	2.036096345			-
d__Bacteria.p__Firmicutes.c__Erysipelotrichia.o__Erysipelotrichales.f__Erysipelotrichaceae	3.44868056			-
d__Bacteria.p__Firmicutes.c__Erysipelotrichia.o__Erysipelotrichales.f__Erysipelotrichaceae.g__Allobaculum	1.297395711			-
d__Bacteria.p__Bacteroidetes.c__Flavobacteriia	2.730243783			-
d__Bacteria.p__Firmicutes.c__Clostridia.o__Clostridiales.f__Ruminococcaceae.g__Flavonifractor	1.760924848			-
d__Bacteria.p__Firmicutes.c__Clostridia.o__Clostridiales.f__Clostridiaceae_1	5.066633985			-
d__Bacteria.p__Actinobacteria.c__Actinobacteria.o__Coriobacteriales.f__Coriobacteriaceae.g__Cryptobacterium	0			-
d__Bacteria.p__Verrucomicrobia.c__Verrucomicrobiae.o__Verrucomicrobiales	1.230448921			-
d__Bacteria.p__Proteobacteria.c__Gammaproteobacteria.o__Xanthomonadales	3.856205086			-
d__Bacteria.p__Firmicutes.c__Clostridia	5.098560666			-
d__Bacteria.p__Firmicutes.c__Clostridia.o__Clostridiales.f__Eubacteriaceae.g__Eubacterium	0			-
d__Bacteria.p__Firmicutes.c__Clostridia.o__Clostridiales.f__Family_XI.g__Anaerococcus	3.233799616	non-NJI	2.925914	0.04632
d__Bacteria.p__Actinobacteria.c__Actinobacteria.o__Pseudonocardiales.f__Pseudonocardiaceae	2.226599905			-
d__Bacteria.p__Firmicutes.c__Clostridia.o__Clostridiales.f__Family_XI.g__Peptoniphilus	2.689160813			-
d__Bacteria.p__Actinobacteria.c__Actinobacteria.o__Pseudonocardiales.f__Pseudonocardiaceae.g__Saccharopolyspora	2.226599905			-
d__Bacteria.p__Bacteroidetes.c__Bacteroidia.o__Bacteroidales.f__Porphyromonadaceae	5.023088453			-
d__Bacteria.p__Firmicutes.c__Clostridia.o__Clostridiales.f__Lachnospiraceae.g__Robinsoniella	0			-

Table S10. Detailed data for linear discriminant analysis (LDA) of the faecal microbial OTUs between neonatal jaundice infants (NJI) and non-NJI at 0 months (continued)

Biomaker_names	Logarithm value	Groups	LDA_value	P_value
d__Bacteria.p__Actinobacteria.c__Actinobacteria.o__Corynebacteriales.f__Nocardiaceae.g__Rhodococcus	1.752048448			-
d__Bacteria.p__Bacteroidetes.c__Flavobacteriia.o__Flavobacteriales.f__Flavobacteriaceae.g__Chryseobacterium	2.730243783			-
d__Bacteria.p__Actinobacteria.c__Actinobacteria.o__Micrococcales.f__Microbacteriaceae	2.158362492			-
d__Bacteria.p__Proteobacteria.c__Gammaproteobacteria.o__Pasteurellales.f__Pasteurellaceae.g__Aggregatibacter	0			-
d__Bacteria.p__Proteobacteria.c__Betaproteobacteria.o__Neisseriales.f__Neisseriaceae.g__Eikenella	0			-
d__Bacteria.p__Proteobacteria.c__Alphaproteobacteria.o__Rhizobiales.f__Phyllobacteriaceae	4.128840663			-
d__Bacteria.p__Firmicutes.c__Clostridia.o__Clostridiales.f__Family_XIII.g__Mogibacterium	0			-
d__Bacteria.p__Firmicutes.c__Bacilli.o__Lactobacillales.f__Enterococcaceae	4.322532839			-
d__Bacteria.p__Proteobacteria.c__Betaproteobacteria.o__Burkholderiales.f__Burkholderiaceae.g__Lautropia	2.36486355			-
d__Bacteria.p__Actinobacteria.c__Actinobacteria.o__Coriobacteriales.f__Coriobacteriaceae	3.196682705			-
d__Bacteria.p__Proteobacteria.c__Betaproteobacteria.o__Burkholderiales	3.988640695			-
d__Bacteria.p__Candidate_division_TM7.c__norank	1.784141614			-
d__Bacteria.p__Proteobacteria.c__Alphaproteobacteria.o__Rhodobacterales.f__Rhodobacteraceae	2.958006125			-
d__Bacteria.p__Firmicutes.c__Bacilli.o__Lactobacillales.f__Lactobacillaceae	3.087367269			-
d__Bacteria.p__Proteobacteria.c__Alphaproteobacteria.o__Caulobacterales	3.426429925			-
d__Bacteria.p__Actinobacteria.c__Actinobacteria.o__Micrococcales.f__Intrasporangiaceae.g__Janibacter	2.36298484			-
d__Bacteria.p__Proteobacteria.c__Betaproteobacteria.o__Rhodocyclales	2.436162647			-
d__Bacteria.p__Proteobacteria.c__Betaproteobacteria.o__Rhodocyclales.f__Rhodocyclaceae.g__Zoogloea	2.336126046			-
d__Bacteria.p__Proteobacteria.c__Betaproteobacteria.o__Neisseriales.f__Neisseriaceae.g__Neisseria	2.866779458			-
d__Bacteria.p__Proteobacteria.c__Gammaproteobacteria.o__Enterobacteriales.f__Enterobacteriaceae.g__Serratia	2.573838205			-
d__Bacteria.p__Proteobacteria.c__Gammaproteobacteria.o__Pseudomonadales.f__Pseudomonadaceae.g__Pseudomonas	2.808661019			-
d__Bacteria.p__Actinobacteria.c__Actinobacteria.o__Micrococcales.f__Dermabacteraceae	2.403977964			-
d__Bacteria.p__Firmicutes.c__Bacilli.o__Bacillales.f__Family_XI	4.16741088	non-NJI	3.920574	0.0132
d__Bacteria.p__Actinobacteria.c__Actinobacteria.o__Coriobacteriales.f__Coriobacteriaceae.g__Atopobium	2.345373731			-
d__Bacteria.p__Actinobacteria.c__Actinobacteria.o__Rubrobacterales	2.363925211			-
d__Bacteria.p__Proteobacteria.c__Betaproteobacteria.o__Neisseriales.f__Neisseriaceae	2.866779458			-
d__Bacteria.p__Firmicutes.c__Bacilli.o__Lactobacillales.f__Carnobacteriaceae.g__Granulicatella	2.658488381			-
d__Bacteria.p__Proteobacteria.c__Alphaproteobacteria.o__Sphingomonadales	3.41808022			-
d__Bacteria.p__Firmicutes.c__Bacilli.o__Lactobacillales.f__Carnobacteriaceae.g__Atopostipes	1.927712462			-
d__Bacteria.p__Bacteroidetes.c__Sphingobacteriia.o__Sphingobacteriales.f__env_OPS_17	1.166331422			-

Table S10. Detailed data for linear discriminant analysis (LDA) of the faecal microbial OTUs between neonatal jaundice infants (NJI) and non-NJI at 0 months (continued)

Biomaker_names	Logarithm value	Groups	LDA_value	P_value
d__Bacteria.p__Firmicutes.c__Bacilli.o__Bacillales.f__Staphylococcaceae.g__Staphylococcus	3.948241757			-
d__Bacteria.p__Bacteroidetes.c__Sphingobacteriia.o__Sphingobacteriales.f__Sphingobacteriaceae	2.564073979			-
d__Bacteria.p__Verrucomicrobia.c__Verrucomicrobiae.o__Verrucomicrobiales.f__Verrucomicrobiaceae	1.230448921			-
d__Bacteria.p__Firmicutes.c__Erysipelotrichia.o__Erysipelotrichales.f__Erysipelotrichaceae.g__Solobacterium	1.661181443			-
d__Bacteria.p__Actinobacteria.c__Actinobacteria.o__Actinomycetales.f__Actinomycetaceae.g__Actinomyces	2.559906625			-
d__Bacteria.p__Actinobacteria.c__Actinobacteria.o__Micrococcales.f__Microbacteriaceae.g__Microbacterium	0.753327667			-
d__Bacteria.p__Bacteroidetes.c__Bacteroidia.o__Bacteroidales	5.057343369			-
d__Bacteria.p__Firmicutes.c__Bacilli.o__Lactobacillales.f__Aerococcaceae.g__Abiotrophia	2.08754481			-
d__Bacteria.p__Proteobacteria.c__Alphaproteobacteria.o__Rhizobiales.f__Methylobacteriaceae	1.096910013			-
d__Bacteria.p__Firmicutes.c__Bacilli.o__Lactobacillales	4.849468553			-
d__Bacteria.p__Actinobacteria.c__Actinobacteria.o__Coriobacteriales.f__Coriobacteriaceae.g__Enterorhabdus	0			-

Table S11. KEGG orthology functional terms identified by PICRUSt as different in neonatal jaundice infants (NJI) and non-NJI at 0 months

Biomaker_names	Logarithm value	Groups	LDA_value	P_value
dihydrofolate_reductase	3.151933721			-
lactaldehyde_dehydrogenase_glycolaldehyde_dehydrogenase	2.606243846			-
f_5_carboxyaminoimidazole_ribonucleotide_synthase	2.544565731			-
shikimate_dehydrogenase	3.105620176			-
putative_ATP_binding_cassette_transporter	2.484867552			-
phenylalanine_dehydrogenase	0			-
mycobactin_phenyloxazoline_synthetase	0			-
N_acetylglucosamine_6_sulfatase	0			-
biotin_synthase	2.596144249			-
tartrate_dehydrogenasedecarboxylase_D_malate_dehydrogenase	2.929735807			-
oxaloacetate_decarboxylase_gamma_subunit	2.182218125			-
small_subunit_ribosomal_protein_S21	2.630477626			-
photosystem_I_subunit_VIII	0			-
multidrug_efflux_pump	2.776585577			-
aromatic_L_amino_acid_decarboxylase	2.264322057			-
two_component_system_sensor_histidine_kinase_RpfC	0.365050958			-
octopinenopaline_transport_system_ATP_binding_protein	0			-
cyanuric_acid_amidohydrolase	0			-
LysW_gamma_L_lysineLysW_L_ornithine_carboxypeptidase	0.059503134			-
photosystem_II_PsbZ_protein	0			-
sirohydrochlorin_cobaltochelatase	2.442997973			-
f_3_deoxy_D_manno_octulosonate_8_phosphate_phosphatase_KDO_8_P_phosphatase	2.524701631			-
acetolactate_synthase_IIIIII_large_subunit	3.783980624			-
two_component_system_chemotaxis_family_response_regulator_PixH	0			-
MFS_transporter_DHA2_family_multidrug_resistance_protein	0.640326467			-
phosphoserine_aminotransferase	3.315801691			-
maleylacetoacetate_isomerase	1.958118369			-
two_component_system_OmpR_family_response_regulator_MtrA	1.012125972			-
two_component_system_chemotaxis_family_response_regulator_PixG	0			-
galactose_6_phosphate_isomerase	1.810816636			0.037373

Table S11. KEGG orthology functional terms identified by PICRUSt as different in neonatal jaundice infants (NJI) and non-NJI at 0 months (continued)

Biomaker_names	Logarithm value	Groups	LDA_value	P_value
small_subunit_ribosomal_protein_S5	2.655976638			-
small_subunit_ribosomal_protein_S4	2.658172515			-
small_subunit_ribosomal_protein_S7	2.656751058			-
small_subunit_ribosomal_protein_S6	2.655659969			-
small_subunit_ribosomal_protein_S1	2.589450659			-
L_arabinose_transport_system_substrate_binding_protein	1.966895974			-
small_subunit_ribosomal_protein_S3	2.656774024			-
small_subunit_ribosomal_protein_S2	2.656660647			-
acetyl_CoA_N6_hydroxylysine_acetyl_transferase	1.497834667			-
ceramide_glucosyltransferase	1.474684166			-
small_subunit_ribosomal_protein_S9	2.655679117			-
small_subunit_ribosomal_protein_S8	2.656832402			-
peptidenickel_transport_system_substrate_binding_protein	3.328825634			-
demethylspheroidene_O_methyltransferase	0			-
cytochrome_aa3_600_menaquinol_oxidase_subunit_II	0.351154079			-
f_6_phospho_beta_galactosidase	1.394270983			-
leucyl_tRNA_synthetase	2.656670563			-
cytochrome_aa3_600_menaquinol_oxidase_subunit_IV	0.351154079			-
anthranilate_synthase	1.639858814			-
tungstate_transport_system_ATP_binding_protein	1.112050863			-
protein_IpgB2	0			-
tryptophan_synthase_alpha_chain	3.063069677			-
ironzincmanganesecopper_transport_system_substrate_binding_protein	0.994740385			-
PTS_system_lactose_specific_IIC_component	1.718123427			-
glycine_hydroxymethyltransferase	3.584944854			-
cytochrome_c_oxidase_subunit_IV	0			-
enoyl_acyl_carrier_protein_reductase_I	3.024069323			-
pyruvate_orthophosphate_dikinase	2.871553608			-
translation_initiation_factor_1	2.520516836			-
tRNA_uridine_2_sulfurtransferase	2.649248003			-

Table S11. KEGG orthology functional terms identified by PICRUSt as different in neonatal jaundice infants (NJI) and non-NJI at 0 months (continued)

Biomaker_names	Logarithm value	Groups	LDA_value	P_value
cytochrome_c_oxidase_subunit_II	1.857253919			-
f_6_phosphogluconate_dehydrogenase	3.055804553			-
D_glycero_D_manno_heptose_1_7_bisphosphate_phosphatase	2.483599944			-
primosomal_protein_N_replication_factor_Y_superfamily_II_helicase	2.655371972			-
two_component_system_OmpR_family_bacitracin_resistance_sensor_histidine_kinase_BceS	0.360532238			-
ribulose_bisphosphate_carboxylase_large_chain	1.819717778			-
diazepam_binding_inhibitor_GABA_receptor_modulator_acyl_CoA_binding_protein	0			-
alkane_1_monooxygenase	1.27438742			-
apocytochrome_f	0			-
cobalt_precorrin_5A_hydrolase_precorrin_3B_C17_methyltransferase	1.162747261			-
anthranilate_1_2_dioxygenase_deaminating_decarboxylating_large_subunit	0			-
acetylglutamate_kinase	3.389179126			-
TetRAcrR_family_transcriptional_regulator_hemagglutininprotease_regulatory_protein	0			-
N2_citryl_N6_acetyl_N6_hydroxylysine_synthase	1.497834667			-
D_xylulose_reductase	0.118957621			-
oxalate_decarboxylase	0			-
precorrin_3B_C17_methyltransferase	2.071264599			-
spheroidene_monooxygenase	0			-
PTS_system_N_acetylgalactosamine_specific_IIC_component	1.998630962			-
f_1_4_dihydroxy_2_naphthoyl_CoA_hydrolase	0			-
mannopine_transport_system_permease_protein	0			-
sorbitolmannitol_transport_system_permease_protein	1.821809612			-
ribose_transport_system_permease_protein	2.799187465			-
rhamnose_transport_system_permease_protein	2.086818953			-
small_subunit_ribosomal_protein_S20	2.650229962			-
cysteinyl_tRNA_synthetase	2.665867661			-
N_acetyl_gamma_glutamyl_phosphate_reductase_acetylglutamate_kinase	0			-
RNA_polymerase_sigma_factor_for_flagellar_operon_FliA	2.911772194			-
sphinganine_1_phosphate_aldolase	0.287740551			-
vesicle_fusing_ATPase	0.18320737			-

Table S11. KEGG orthology functional terms identified by PICRUSt as different in neonatal jaundice infants (NJI) and non-NJI at 0 months (continued)

Biomaker_names	Logarithm value	Groups	LDA_value	P_value
glycolate_oxidase	1.795610388			-
ribose_5_phosphate_isomerase_A	3.15953252			-
phosphoenolpyruvate_carboxykinase_GTP	2.365440758			-
ribose_5_phosphate_isomerase_B	2.976982959			-
two_component_system_LuxR_family_response_regulator_TtrR	1.856453748			-
histidinol_phosphatase_PHP_family	2.651594201			-
UDP_sulfoquinovose_synthase	1.436942887			-
arylformamidase	2.417610347			-
maltose_6_phosphate_phosphatase	2.612345923			-
ribonuclease_HI	2.625296641			-
UDP_2_acetamido_3_amino_2_3_dideoxy_glucuronate_N_acetyltransferase	0.080504934			-
lipoprotein_LprA	0			-
PTS_system_sorbose_specific_IIB_component	2.0789187			-
lipoprotein_LprG	0			-
ornithine_cyclodeaminase	2.281446546			-
flagellar_hook_basal_body_complex_protein_FliE	2.480044957			-
agmatine_deiminase	1.583772691			-
N_acylmannosamine_kinase	1.790221356			-
shikimate_kinase_3_dehydroquinate_synthase	2.021730382			-
valyl_tRNA_synthetase	2.656822426			-
threonine_synthase	3.139863259			-
H_transporting_ATPase	0			0.0222296
alkaline_phosphatase_D	2.065076708			-
extradiol_dioxygenase	0			-
ironIII_transport_system_substrate_binding_protein	2.702387572			-
phosphoribosylaminoimidazolecarboxamide_formyltransferase	1.298098396			-
putative_spermidineputrescine_transport_system_ATP_binding_protein	2.602011782			-
acyl_CoA_thioesterase_YciA	2.49552715			-
N_acetylgalactosamine_6_phosphate_deacetylase	1.652595513			-
Kdo2_lipid_IVA_lauroyltransferase	2.544194363			-

Table S11. KEGG orthology functional terms identified by PICRUSt as different in neonatal jaundice infants (NJI) and non-NJI at 0 months (continued)

Biomaker_names	Logarithm value	Groups	LDA_value	P_value
type_VI_secretion_system_protein_VasG	2.786552468			-
large_subunit_ribosomal_protein_L7A	1.844847892			-
cobalt_precorrin_5A_hydrolase	2.063988867			-
type_VI_secretion_system_protein_VasD	2.631769899			-
chlorophyllide_a_reductase_subunit_Y	0			-
cyanate_lyase	1.402177603			-
phycocyanin_alpha_chain	0			-
homocitrate_synthase	0			-
ubiquinol_cytochrome_c_reductase_iron_sulfur_subunit	2.510163541			-
f_2_hydroxy_3_keto_5_methylthiopentenyl_1_phosphate_phosphatase	0			-
FMN_reductase	2.294031849			-
two_component_system_OmpR_family_bacitracin_resistance_response_regulator_BceR	0.360532238			-
uracil_DNA_glycosylase	2.918034227			-
xylulose_5_phosphatefructose_6_phosphate_phosphoketolase	1.326997759			0.0249747
negative_regulator_of_flagellin_synthesis_FlgM	2.864783158			-
heptosyltransferase_I	2.474782221			-
c_di_GMP_specific_phosphodiesterase	0			-
chorismate_mutase_prephenate_dehydratase	3.090329545			-
two_component_system_NtrC_family_sensor_histidine_kinase_KinB	0			-
maleate_isomerase	1.719723408			-
f_6_aminohexanoate_oligomer_exohydrolase	1.657244722			-
dissimilatory_sulfite_reductase_alpha_subunit	0.342352678			-
maltosemaltodextrin_transport_system_permease_protein	2.638291708			-
flagellar_biosynthesis_protein_FlhA	2.485418306			-
cytochrome_o_ubiquinol_oxidase_subunit_I	2.482214872			-
flagellar_biosynthesis_protein_FlhG	0.972263228			-
aspartate_semialdehyde_dehydrogenase	3.613086729			-
hydroxymethylglutaryl_CoA_reductase_NADPH	0			-
glutamine_amidotransferase	2.919871069			-
two_component_system_chemotaxis_family_CheBCheR_fusion_protein	1.139580324			-

Table S11. KEGG orthology functional terms identified by PICRUSt as different in neonatal jaundice infants (NJI) and non-NJI at 0 months (continued)

Biomaker_names	Logarithm value	Groups	LDA_value	P_value
PTS_system_sugar_specific_IIA_component	3.190565557			-
two_component_system_OmpR_family_response_regulator_RstA	1.981279906			-
f_2_3_dihydro_2_3_dihydroxybenzoate_dehydrogenase	1.966523141			-
lysyl_tRNA_synthetase_class_I	1.411293669			-
peptidoglycan_pentaglycine_glycine_transferase_the_first_glycine	0.479772801			-
alpha_aminoadipateglutamate_carrier_protein_LysW	0.059503134			-
bifunctional_enzyme_CysNCysC	2.560824739			-
geranylgeranyl_diphosphate_synthase_type_I	1.916590475			-
bis5_nucleosidyl_tetraphosphatase	0.582853384			-
arylesterase_paraoxonase	0			-
single_stranded_DNA_specific_exonuclease	3.111161133			-
L_ribulose_5_phosphate_3_epimerase	1.981953001			-
altronate_hydrolase	2.268903614			-
f_5_methylthioadenosine_phosphorylase	1.32230813			-
pyruvate_oxidase	1.184336905			-
ethylbenzene_dioxygenase_subunit_alpha	0.043825794			-
two_component_system_OmpR_family_response_regulator_VicR	1.630511648			-
type_III_pantothenate_kinase	2.247711695			0.037373
oligo_1_6_glucosidase	2.246065513			-
biphenyl_2_3_dioxygenase_subunit_alpha	1.411414468			-
dihydroxycyclohexadiene_carboxylate_dehydrogenase	1.439159812			-
phosphorylase_kinase_alphabeta_subunit	0			-
ureidoglycolate_lyase	1.712824491			-
T3SS_secreted_effector_EspG_like_protein	0			-
photosystem_II_PsbX_protein	0			-
peptidoglycan_glycosyltransferase	1.464180751			-
sulfate_adenylyltransferase	1.426920209			-
enoyl_CoA_hydratase	3.983839847			-
glycerol_1_phosphate_dehydrogenase_NADP	0.324399859			-
porphobilinogen_synthase	2.502544549			-

Table S11. KEGG orthology functional terms identified by PICRUSt as different in neonatal jaundice infants (NJI) and non-NJI at 0 months (continued)

Biomaker_names	Logarithm value	Groups	LDA_value	P_value
two_component_system_OmpR_family_clock_associated_histidine_kinase_SasA	0			-
f_2_keto_4_pentenoate_hydratase	2.486180101			-
f_6_oxocyclohex_1_ene_carbonyl_CoA_hydrolase	0			-
phenylalanyl_tRNA_synthetase_alpha_chain	2.656820578			-
proline_dehydrogenase	1.759630593			-
trimethylamine_N_oxide_reductase_cytochrome_c	0.640326467			-
phytoene_desaturase	0.881631586			-
phosphoribosylglycinamide_formyltransferase_1	2.941868137			-
neprilysin	0.295948006			-
phosphoribosylglycinamide_formyltransferase_2	2.851384387			-
diamine_N_acetyltransferase	2.547639715			-
phosphate_transport_system_substrate_binding_protein	3.16754965			-
isovaleryl_CoA_dehydrogenase	1.702483929			-
formate_dehydrogenase_N_beta_subunit	1.959826256			-
para_aminobenzoate_synthetase_component_I	2.532531711			-
K_transporting_ATPase_ATPase_A_chain	2.524929491			-
HK_exchanging_ATPase	1.166922023			0.0360487
formiminotetrahydrofolate_cyclodeaminase	1.108338801			-
mycobactin_polyketide_synthetase_MbtC	0			-
mycobactin_polyketide_synthetase_MbtD	0			-
two_component_system_response_regulator_FlrC	0.686396419			-
PTS_system_glucitolsorbitol_specific_IIA_component	2.502321561			-
glutamyl_tRNA_synthetase	3.019419876			-
preprotein_translocase_subunit_YajC	3.132590247			-
MFS_transporter_PAT_family_beta_lactamase_induction_signal_transducer_AmpG	2.544629987			-
peptidenickel_transport_system_ATP_binding_protein	3.424571027			-
ureidoacrylate_peracid_hydrolase	1.985859713			-
dipeptidyl_peptidase_4	2.264498526			-
photosynthetic_reaction_center_H_subunit	0			-
deoxyadenosine_kinase	0			-

Table S11. KEGG orthology functional terms identified by PICRUSt as different in neonatal jaundice infants (NJI) and non-NJI at 0 months (continued)

Biomaker_names	Logarithm value	Groups	LDA_value	P_value
xanthine_dehydrogenase_FAD_binding_subunit	0.926637543			-
rubredoxin_NAD_reductase	1.274574476			-
S_hydroxymethylglutathione_dehydrogenase_alcohol_dehydrogenase	3.719591637			-
acid_activated_urea_channel	0.135380579			0.0224867
proteasome_alpha_subunit	0.572257776			-
f_2_oxoglutarate_dehydrogenase_E2_component_dihydrolipoamide_succinyltransferase	2.99726568			-
sterol_14_demethylase	0.052538071			-
glutamate_transport_system_substrate_binding_protein	1.431604742			-
phosphoribosylaminoimidazole_succinocarboxamide_synthase	2.65677881			-
two_component_system_sensor_histidine_kinase_YesM	2.083961496			0.0249747
photosystem_II_CP43_chlorophyll_apoprotein	0			-
mandelate_racemase	0			-
bacterioferritin	2.535585434			-
two_component_system_NarL_family_uhpT_operon_response_regulator_UhpA	2.55095786			-
anaerobic_C4_dicarboxylate_transporter_DcuB	2.513698055			-
adenosylcobinamide_phosphate_synthase	2.284724672			-
ferredoxin__NADP_reductase	0			-
muconolactone_D_isomerase	1.223281759			-
endoglucanase	2.363796723			-
benzoate_CoA_ligase	0.824518629			-
glutamate_decarboxylase	3.24948943			-
small_subunit_ribosomal_protein_S11	2.656832402			-
small_subunit_ribosomal_protein_S10	2.655674913			-
small_subunit_ribosomal_protein_S13	2.655683759			-
small_subunit_ribosomal_protein_S12	2.656503843			-
small_subunit_ribosomal_protein_S15	2.656254091			-
f_3_oxo_5_alpha_steroid_4_dehydrogenase_2	1.11902902			-
small_subunit_ribosomal_protein_S17	2.656324967			-
small_subunit_ribosomal_protein_S16	2.656074363			-
small_subunit_ribosomal_protein_S19	2.656425697			-

Table S11. KEGG orthology functional terms identified by PICRUSt as different in neonatal jaundice infants (NJI) and non-NJI at 0 months (continued)

Biomaker_names	Logarithm value	Groups	LDA_value	P_value
small_subunit_ribosomal_protein_S18	2.656807454			-
medium_chain_acyl_CoA_synthetase	0			-
phosphoribosylformimino_5_aminoimidazole_carboxamide_ribotide_isomerase	2.904001049			-
f_3_dehydroquinate_dehydratase_I	2.421424651			-
two_component_system_NarL_family_sensor_histidine_kinase_UhpB	2.552814576			-
isochorismate_synthase	2.433675631			-
preprotein_translocase_subunit_SecY	3.146701867			-
dTDP_4_dehydrorhamnose_reductase	2.756100149			-
phenol_2_monooxygenase	1.666481024			-
aerobactin_synthase	1.497834667			-
pyruvate_dehydrogenase_E1_component_beta_subunit	3.037298376			-
f_4_hydroxybenzoate_polyprenyltransferase	2.493426623			-
two_component_system_NtrC_family_response_regulator_AlgB	0			-
peptidenickel_transport_system_permease_protein	3.489265341			-
alpha_D_ribose_1_methylphosphonate_5_triphosphate_synthase_subunit_PhnG	2.015460387			-
phosphotransferase_system_enzyme_I_PtsI	2.55998274			-
C4_dicarboxylate_transporter_DctM_subunit	2.015435169			-
alpha_D_ribose_1_methylphosphonate_5_triphosphate_synthase_subunit_PhnL	2.014612652			-
CTP_synthase	2.653416337			-
two_component_system_LytT_family_sensor_histidine_kinase_LytS	1.142722653			-
alpha_D_ribose_1_methylphosphonate_5_triphosphate_synthase_subunit_PhnH	2.015416706			-
alpha_D_ribose_1_methylphosphonate_5_triphosphate_synthase_subunit_PhnI	2.015460387			-
formate_dehydrogenase_N_alpha_subunit	2.016287819			-
two_component_system_NarL_family_sensor_histidine_kinase_DegS	0.387015783			0.0039478
f_2_methylaconitate_isomerase	2.539825665			-
peptidoglycan_pentaglycine_glycine_transferase_the_second_and_third_glycine	0.446523244			-
phosphotransferase_system_enzyme_I_PtsP	2.491164707			-
PTS_system_N_acetylgalactosamine_specific_IIA_component	2.068800021			-
multiple_sugar_transport_system_ATP_binding_protein	2.398565506			-
phosphonate_transport_system_ATP_binding_protein	2.090483438			-

Table S11. KEGG orthology functional terms identified by PICRUSt as different in neonatal jaundice infants (NJI) and non-NJI at 0 months (continued)

Biomaker_names	Logarithm value	Groups	LDA_value	P_value
tryptophan_2_monooxygenase	0.248693322			-
toxic_shock_syndrome_toxin_1	0			-
fucokinase	0.572981499			0.0063923
urate_oxidase	1.423625873			-
two_component_system_NtrC_family_sensor_histidine_kinase_GlrK	2.774641088			-
malonyl_CoA_decarboxylase	1.894067773			-
cystine_transport_system_permease_protein	1.980114029			-
heme_exporter_protein_D	1.818114481			-
alpha_D_ribose_1_methylphosphonate_5_phosphate_C_P_lyase	2.014931536			-
heme_exporter_protein_B	2.448300675			-
anthranilate_synthase_component_I	3.171430807			-
nitrous_oxide_reductase	1.151045093			-
thiaminase_transcriptional_activator_TenA	2.225660145			-
photosystem_II_PsbY_protein	0			-
CDP_diacylglycerol_pyrophosphatase	1.965444051			-
polyphosphate_glucokinase	1.918726992			-
geranylgeranyl_reductase	0.228955648			-
f_3_phosphoshikimate_1_carboxyvinyltransferase	2.950183599			-
two_component_system_LuxR_family_sensor_histidine_kinase_TtrS	1.85634534			-
bicarbonate_transport_system_substrate_binding_protein	0			-
L_arabinose_transport_system_ATP_binding_protein	1.966895974			-
penicillin_binding_protein_2_prime	0			-
UDP_4_amino_4_deoxy_L_arabinose_oxoglutarate_aminotransferase	2.949788079			-
transketolase	3.741437904			-
galactitol_1_phosphate_5_dehydrogenase	1.790535419			-
thiamine_pyrophosphokinase	2.30303461			-
UDP_N_acetyl_2_amino_2_deoxyglucuronate_dehydrogenase	1.735777223			-
thiamine_biosynthesis_protein_ThiI	2.810810503			-
aspartate_aminotransferase	3.780017439			-
two_component_system_OmpR_family_phosphate_regulon_response_regulator_PhoB	2.494290506			-

Table S11. KEGG orthology functional terms identified by PICRUSt as different in neonatal jaundice infants (NJI) and non-NJI at 0 months (continued)

Biomaker_names	Logarithm value	Groups	LDA_value	P_value
glycolate_oxidase_FAD_binding_subunit	1.594159984			-
leucyl_aminopeptidase	2.617363613			-
f_3_hydroxyacyl_CoA_dehydrogenase_enoyl_CoA_hydratase_3_hydroxybutyryl_CoA_epimerase	4.009377856			-
precorrin_2_dehydrogenase_sirohydrochlorin_ferrochelatase	1.961707188			0.0249747
phosphoserine_phosphatase	3.206473587			-
two_component_system_LytT_family_sensor_histidine_kinase_NatK	0			-
coproporphyrinogen_III_oxidase	2.492926086			-
malate_dehydrogenase	3.486723436			-
phytanoyl_CoA_hydroxylase	0.124116414			-
VA_type_HNa_transporting_ATPase_subunit_B	2.196045921			-
VA_type_HNa_transporting_ATPase_subunit_C	2.00242276			-
VA_type_HNa_transporting_ATPase_subunit_A	2.194939251			-
VA_type_HNa_transporting_ATPase_subunit_F	2.003262969			-
N_acetylneuraminate_lyase	2.230394044			-
isopenicillin_N_N_acyltransferase	1.879972815			-
serine_protease_Do	2.266402687			-
nitrate_reductase_nitrite_oxidoreductase_beta_subunit	2.343141859			-
VA_type_HNa_transporting_ATPase_subunit_I	2.19586093			-
glutamate_synthase_ferredoxin	2.165137265			0.0163092
AraC_family_transcriptional_regulator_regulatory_protein_of_adaptative_response_DNA_3_methyladenine_glycosylase_II	0.927819715			-
phosphosulfolactate_synthase	0			-
adenylyltransferase_and_sulfurtransferase	2.576396132			-
inorganic_pyrophosphatase	2.769099209			-
molecular_chaperone_Hsp31_and_glyoxalase_3	0.78296078			-
sulfate_transport_system_ATP_binding_protein	2.785402642			-
type_VI_secretion_system_protein_ImpL	2.932203377			-
UDP_N_acetyl_D_mannosaminuronic_acid_dehydrogenase	2.846750684			-
glycyl_tRNA_synthetase_beta_chain	2.508102035			-
f_3_phenylpropionatetrans_cinnamate_dioxygenase_subunit_beta	1.395134857			-
polyribonucleotide_nucleotidyltransferase	3.133282961			-

Table S11. KEGG orthology functional terms identified by PICRUSt as different in neonatal jaundice infants (NJI) and non-NJI at 0 months (continued)

Biomaker_names	Logarithm value	Groups	LDA_value	P_value
glutaminase	3.517970137			-
f_17beta_estradiol_17_dehydrogenase	0			-
cytochrome_c_oxidase_cbb3_type_subunit_III	1.972040018			-
osmoprotectant_transport_system_permease_protein	2.692004542			-
hydrogenase_large_subunit	2.387454165			-
N_acetyldiaminopimelate_deacetylase	1.799951352			-
RNA_polymerase_sigma_54_factor	2.90363101			-
alanine_racemase	3.148078713			-
f_6_carboxyhexanoate__CoA_ligase	0.240052402			-
maltose_6_phosphate_glucosidase	2.267407443			-
f_1L_myo_inositol_1_phosphate_cytidylyltransferase	0			-
photosystem_II_PsbT_protein	0			-
holo_ACP_synthase	2.554359115			-
proline_racemase	0.541502277			-
tetraacyldisaccharide_4_kinase	2.53599452			-
f_2_oxo_3_hexenedioate_decarboxylase	2.008752153			-
f_2_6_dioxo_6_phenylhexa_3_enoate_hydrolase	0.043825794			-
enoyl_acyl_carrier_protein_reductase_III	0			-
hydroxyacylglutathione_hydrolase	2.546423551			-
homocysteine_S_methyltransferase	1.992661252			-
oligogalacturonide_transport_system_permease_protein	2.253353968			-
competence_factor_transport_accessory_protein_ComB	1.062904361			-
myo_inositol_1_phosphate_synthase	1.660367265			-
two_component_system_AgrA_family_sensor_histidine_kinase_AgrC	1.676402937			-
PAH_dioxygenase_small_subunit	0			-
cytochrome_c_oxidase_cbb3_type_subunit_IV	1.943440871			-
PTS_system_N_acetylglucosamine_specific_IIA_component	0.258867441			-
alpha_glucoside_transport_system_ATP_binding_protein	1.010111702			-
chemosensory_pili_system_protein_ChpB_putative_protein_glutamate_methylesterase	0			-

Table S11. KEGG orthology functional terms identified by PICRUSt as different in neonatal jaundice infants (NJI) and non-NJI at 0 months (continued)

Biomaker_names	Logarithm value	Groups	LDA_value	P_value
agmatinase	2.589200062			-
PTS_system_beta_glucoside_arbutinsalicincellobiose_specific_IIC_component	2.263756099			-
f_2_dehydro_3_deoxygalactonokinase	2.23869486			-
lecithin_cholesterol_acyltransferase	0			-
two_component_system_cell_cycle_sensor_histidine_kinase_PleC	1.9542165			-
two_component_system_OmpR_family_osmolarity_sensor_histidine_kinase_EnvZ	2.489802561			-
PTS_system_nitrogen_regulatory_IIA_component	2.506862374			-
histidine_ammonia_lyase	2.347515566			-
citrate_lyase_subunit_alpha_citrate_CoA_transferase	2.66742528			-
light_independent_protochlorophyllide_reductase_subunit_L	0			-
sec_independent_protein_translocase_protein_TatE	2.265625973			-
hydroxydechloroatrazine_ethylaminohydrolase	1.971866965			-
adenosylcobinamide_GDP_ribazoletransferase	2.298711696			-
protochlorophyllide_reductase	0			-
oxaloacetate_decarboxylase_alpha_subunit	2.282028083			-
PTS_system_mannose_specific_IIC_component	3.243012366			-
ATP_binding_cassette_subfamily_B_bacterial_HlyBCyaB	0			-
threonine_phosphate_decarboxylase	2.216539935			-
photosystem_II_PsbI_protein	0			-
carbon_monoxide_dehydrogenase_large_subunit	2.28296223			-
neutral_amino_acid_transport_system_permease_protein	0			-
ribitol_2_dehydrogenase	1.77955359			-
hydroxymethylglutaryl_CoA_lyase	2.33971607			-
molybdopterin_synthase_catalytic_subunit	2.797367065			-
catalase_peroxidase	2.818319182			-
thiamine_transport_system_ATP_binding_protein	2.478172102			-
dimethylaniline_monooxygenase_N_oxide_forming	1.197849377			-
coenzyme_F420_0_L_glutamate_ligase_coenzyme_F420_1_gamma_L_glutamate_ligase	1.220729537			-
acid_phosphatase_class_A	2.170588787			-
acid_phosphatase_class_B	2.471809387			-

Table S11. KEGG orthology functional terms identified by PICRUSt as different in neonatal jaundice infants (NJI) and non-NJI at 0 months (continued)

Biomaker_names	Logarithm value	Groups	LDA_value	P_value
phosphatidylcholine_synthase	1.488463033			-
glycogen_phosphorylase	3.298437479			-
adenine_deaminase	2.583040376			-
protein_PII_uridylyltransferase	2.528288543			-
acyl_CoA_oxidase	1.640084689			-
L_rhamnose_isomerase	2.091466925			-
two_component_system_NtrC_family_C4_dicarboxylate_transport_sensor_histidine_kinase_DctB	1.665172206			-
flavin_reductase	1.998753677			-
L_2_hydroxycarboxylate_dehydrogenase_NAD	0.130112323			0.0091059
tryptophanyl_tRNA_synthetase	2.684802799			-
nitrogenase_molybdenum_iron_protein_alpha_chain	2.102246054			-
phosphogluconate_2_dehydrogenase	0			-
molecular_chaperone_HtpG	3.521948698			-
mycobactin_salicyl_AMP_ligase	0			-
phycocyanin_beta_chain	0			-
Rrf2_family_transcriptional_regulator_nitric_oxide_sensitive_transcriptional_repressor	2.497122307			-
molecular_chaperone_DnaK	3.192743287			-
D_sorbitol_dehydrogenase_acceptor	1.287455786			-
formamidopyrimidine_DNA_glycosylase	2.785536087			-
lipopolysaccharide_export_system_permease_protein	2.795079755			-
UDP_glucose_galactosylLPS_alpha_1_2_glucosyltransferase	0			-
phosphatidylglycerophosphatase_B	2.473581026			-
phosphatidylglycerophosphatase_A	2.453149724			-
S_2_hydroxy_acid_oxidase	0			-
ATP_dependent_Clp_protease_protease_subunit	3.006734			-
plastocyanin	0			-
protein_S_isoprenylcysteine_O_methyltransferase	0			-
pyridoxamine_5_phosphate_oxidase	2.510810162			-
anaerobic_dimethyl_sulfoxide_reductase_subunit_C_DMSO_reductase_anchor_subunit	2.476867292			-
chemotaxis_related_protein_WspB	0.243933721			-

Table S11. KEGG orthology functional terms identified by PICRUSt as different in neonatal jaundice infants (NJI) and non-NJI at 0 months (continued)

Biomaker_names	Logarithm value	Groups	LDA_value	P_value
chemotaxis_related_protein_WspD	0.243933721			-
putrescine_transport_system_permease_protein	2.32056892			-
pectate_lyase	2.077620515			-
DNA_polymerase_III_subunit_chi	3.192144805			-
YidCOxa1_family_membrane_protein_insertase	3.176767152	non-NJI	2.20372245	0.037373
neutral_amino_acid_transport_system_ATP_binding_protein	0			-
dethiobiotin_synthetase	2.802547827			-
f_5_nucleotidase	3.25784412			-
D_lactate_dehydrogenase	2.804998125			-
sarcosine_oxidase_subunit_beta	1.874080441			-
regulator_of_CtrA_degradation	1.394657945			-
demethylmenaquinone_methyltransferase_2_methoxy_6_polyprenyl_1_4_benzoquinol_methylase	2.91613332			-
aminotransferase	2.048302306			-
f_2_hydroxyisoflavone_reductase	0			-
phenylalanine_4_hydroxylase	1.662998941			-
citrate_synthase	3.376404533			-
phycobilisome_core_membrane_linker_protein	0			-
UDP_N_acetylglucosamine_glucosylLPS_alpha_1_2_N_acetylglucosaminyltransferase	0			-
glyoxylatehydroxypyruvate_reductase_A	3.178338799			-
anaerobic_dimethyl_sulfoxide_reductase_subunit_B_DMSO_reductase_iron__sulfur_subunit	2.585147974			-
ATP_phosphoribosyltransferase_regulatory_subunit	2.122341979			-
ribose_phosphate_pyrophosphokinase	3.313794229			-
f_2_methylcitrate_dehydratase	2.315626846			-
asparaginyl_tRNA_synthetase	2.578508068			-
citronellolcitronellal_dehydrogenase	0.905482911			-
glutamyl_endopeptidase	0			-
carbonic_anhydrase	2.753677813			-
D_alanyl_D_alanine_carboxypeptidase_penicillin_binding_protein_56	2.882354709			-
S_formylglutathione_hydrolase	2.79507255			-
carbon_monoxide_dehydrogenase_catalytic_subunit	2.47571001	non-NJI	2.05010306	0.0163092

Table S11. KEGG orthology functional terms identified by PICRUSt as different in neonatal jaundice infants (NJI) and non-NJI at 0 months (continued)

Biomaker_names	Logarithm value	Groups	LDA_value	P_value
arginine_pyruvate_transaminase	1.238393348			-
N_isopropylammelide_isopropylaminohydrolase	0			-
L_gulonolactone_oxidase	0.698096695			-
photosystem_II_Psb27_protein	0			-
phycocyanin_associated_rod	0			-
PTS_system_sorbose_specific_IID_component	2.079069318			-
mannopine_transport_system_ATP_binding_protein	0			-
two_component_system_OmpR_family_sensor_histidine_kinase_MtrB	1.005816734			-
phosphatidylethanolamine_N_methyltransferase	1.154981597			-
tRNA_2_thiouridine_synthesizing_protein_C	2.47813013			-
tRNA_2_thiouridine_synthesizing_protein_B	2.478045795			-
tRNA_2_thiouridine_synthesizing_protein_A	2.491676386			-
transcription_initiation_factor_TFIIB	0			-
tRNA_2_thiouridine_synthesizing_protein_E	2.479048976			-
tRNA_2_thiouridine_synthesizing_protein_D	2.478253869			-
all_trans_nonaprenyl_diphosphate_synthase	0			-
minor_extracellular_serine_protease_Vpr	0			-
primary_amine_oxidase	2.738735728			-
cyclomaltodextrinase_maltogenic_alpha_amylase_neopullulanase	0			0.0091059
f_2_enoate_reductase	0.62362458			-
F_type_H_transporting_ATPase_subunit_gamma	2.957960841			-
pyruvate_dehydrogenase_quinone	2.490792388			-
formimidoylglutamate_deiminase	1.780340073			-
f_2_aminoethylphosphonate_transport_system_permease_protein	2.074422106			-
N4_beta_N_acetylglucosaminyl_L_asparaginase	0.938605542			-
monofunctional_glycosyltransferase	0.343985124			-
f_2_3_dihydroxybenzoate_AMP_ligase	2.594305745			-
f_4_hydroxy_2_oxovalerate_aldolase	2.659536645			-
alpha_ribazole_phosphatase	1.998036899			-
cinnamoyl_CoA_phenyllactate_CoA_transferase	0.227468394			-

Table S11. KEGG orthology functional terms identified by PICRUSt as different in neonatal jaundice infants (NJI) and non-NJI at 0 months (continued)

Biomaker_names	Logarithm value	Groups	LDA_value	P_value
menaquinol_cytochrome_c_reductase_cytochrome_bc_subunit	0			-
two_component_system_LytT_family_sensor_histidine_kinase_AlgZ	1.007139257			-
cytochrome_o_ubiquinol_oxidase_subunit_III	2.482219376			-
malate_dehydrogenase_decarboxylating	1.72727671			-
ATP_dependent_RNA_helicase_DeaD	2.550384923			-
cystathionine_gamma_synthase	3.130123988			-
f_2_methylcitrate_synthase	2.322188857			-
f_3_isopropylmalate_dehydrogenase	3.248231636			-
two_component_system_NtrC_family_nitrogen_regulation_sensor_histidine_kinase_GlnL	2.492852251			-
phenylalanine_ammonia_lyase	0			-
f_4_hydroxyphenylacetate_3_monooxygenase	2.315324767			-
nucleoside_diphosphate_kinase	3.01447525			-
naphthalene_1_2_dioxygenase_subunit_alpha	0.811412571			-
aquacobalamin_reductase_NADPH_flavin_reductase	3.075192153			-
alanyl_tRNA_synthetase	2.656502425			-
creatinine_amidohydrolase	2.232525234			-
glycerate_dehydrogenase	2.574498949			-
DNA_polymerase_I	3.441241936			-
glucose_6_phosphate_isomerase	3.363085232			-
f_4_diphosphocytidyl_2_C_methyl_D_erythritol_kinase	2.635494017			-
cholesterol_oxidase	0.600185237			-
salicylate_hydroxylase	2.461435422			-
glutamate_1_semialdehyde_2_1_aminomutase	2.502940991			-
fibrinogen_binding_protein	0			-
zinc_transport_system_ATP_binding_protein	2.604872349			-
two_component_system_OmpR_family_response_regulator_PhoP	2.85315049			-
two_component_system_CitB_family_sensor_histidine_kinase_MalK	0			-
long_chain_acyl_CoA_dehydrogenase	1.683499127			-
thiamine_pyridinylase	0			-
f_6_phosphofructo_2_kinase_fructose_2_6_biphosphatase_3_160	0.855135301			-

Table S11. KEGG orthology functional terms identified by PICRUSt as different in neonatal jaundice infants (NJI) and non-NJI at 0 months (continued)

Biomaker_names	Logarithm value	Groups	LDA_value	P_value
DNA_replication_and_repair_protein_RecF	2.651905448			-
L_ascorbate_6_phosphate_lactonase	1.985131588			-
toxin_co_regulated_pilus_biosynthesis_protein_E	0			-
mannose_6_phosphate_isomerase	2.973945198			-
dUTP_pyrophosphatase	2.654959717			-
argininosuccinate_lyase	3.129582452			-
glutaminyl_tRNA_synthetase	2.554328859			-
toxin_co_regulated_pilus_biosynthesis_protein_T	0			-
fructose_transport_system_ATP_binding_protein	1.074071815			-
photosystem_I_subunit_VII	0.186475537			-
phenylalanyl_tRNA_synthetase_beta_chain	2.656715097			-
tungstate_transport_system_permease_protein	1.120417255			-
rhamnulokinase	2.370112469			-
flagella_basal_body_P_ring_formation_protein_FlgA	2.409611103			-
two_component_system_response_regulator_YcbB	1.811473979			-
glutaconyl_CoA_decarboxylase	2.157652639			-
microcin_C_transport_system_substrate_binding_protein	2.055424138			-
cyclic_di_GMP_phosphodiesterase_Gmr	1.5181414			-
beta_phosphoglucomutase	1.851972228			-
phycocyanobilin_lyase_subunit_alpha	0			-
signal_recognition_particle_subunit_SRP54	3.133903013			-
f_5_methylthioadenosineS_adenosylhomocysteine_deaminase	2.42653108			-
CuIAgI_efflux_system_periplasmic_protein_CusF	2.020523497			-
branched_chain_amino_acid_transport_system_permease_protein	3.42727522			-
branched_chain_amino_acid_transport_system_substrate_binding_protein	3.190080246			-
lactoseL_arabinose_transport_system_ATP_binding_protein	1.11363308			-
peptidyl_dipeptidase_A	0.585492246			-
glutathione_S_transferase	3.620653953			-
heptosyltransferase_III	2.475591196			-
PTS_system_mannose_specific_IID_component	3.210403043			-

Table S11. KEGG orthology functional terms identified by PICRUSt as different in neonatal jaundice infants (NJI) and non-NJI at 0 months (continued)

Biomaker_names	Logarithm value	Groups	LDA_value	P_value
nitrite_reductase_NADH_small_subunit	2.024556283			-
D_allose_transport_system_permease_protein	1.951920337			-
anomeric_MurNAcGlcNAc_kinase	1.673393344			-
acetate_kinase	3.453042066			-
D_proline_reductase_dithiol_PrdD	0.257146233			-
D_proline_reductase_dithiol_PrdE	0.533981447			-
D_proline_reductase_dithiol_PrdB	0			0.0495102
F_type_H_transporting_ATPase_subunit_c	2.957377313			-
f_2_3_diketo_5_methylthiopentyl_1_phosphate_enolase	0			-
D_proline_reductase_dithiol_PrdA	0.269453945			0.0161224
type_VI_secretion_system_protein	0			-
F_type_H_transporting_ATPase_subunit_b	2.962611364			-
phospholipidcholesterolgamma_HCH_transport_system_ATP_binding_protein	2.539972025			-
N_acyl_homoserine_lactone_hydrolase	0.816001978			-
ethanolamine_utilization_cobalamin_adenosyltransferase	1.786452194			-
f_2_hydroxy_6_oxohepta_2_4_dienoate_hydroxylase	0			-
tropinone_reductase_I	0			-
rhamnulose_1_phosphate_aldolase	2.405830928			-
PTS_system_galactitol_specific_IIA_component	2.801424544			-
protective_antigen	0			-
type_IV_secretion_system_protein_VirB3	1.845264979			-
type_IV_secretion_system_protein_VirB2	2.260986477			-
type_IV_secretion_system_protein_VirB5	1.96244607			-
type_IV_secretion_system_protein_VirB4	1.969121619			-
type_IV_secretion_system_protein_VirB7	0.957580793			-
type_IV_secretion_system_protein_VirB6	1.807441973			-
type_IV_secretion_system_protein_VirB9	1.975806833			-
type_IV_secretion_system_protein_VirB8	1.806445259			-
two_component_system_OmpR_family_sensor_histidine_kinase_QseC	2.777352275			-
f_3_isopropylmalateR_2_methylmalate_dehydratase_large_subunit	3.54320268			-

Table S11. KEGG orthology functional terms identified by PICRUSt as different in neonatal jaundice infants (NJI) and non-NJI at 0 months (continued)

Biomaker_names	Logarithm value	Groups	LDA_value	P_value
phosphomevalonate_kinase	1.618509884			-
L_tartrate_dehydratase_beta_subunit	1.796008966			-
pyruvate_ferredoxinflavodoxin_oxidoreductase	3.203460578			-
f_4_hydroxy_2_oxoheptanedioate_aldolase	2.099225348			-
DNA_polymerase_III_subunit_theta	3.173390006			-
adenylylsulfate_reductase_subunit_B	0.032421976			-
holo_ACP_synthase_triphosphoribosyl_dephospho_CoA_synthase	0			0.0161224
cellobiose_transport_system_permease_protein	1.67928509			-
RNA_polymerase_nonessential_primary_like_sigma_factor	2.479315697			-
ATP_binding_cassette_subfamily_B_bacterial_RtxB	2.311589269			-
F_type_H_transporting_ATPase_subunit_epsilon	2.957304665			-
two_component_system_NarL_family_competent_response_regulator_ComA	0			-
NADP_dependent_aldehyde_dehydrogenase	1.91790334			-
chorismate__pyruvate_lyase	2.478992483			-
glutamate_formiminotransferase	2.417680712			-
bla_regulator_protein_blaR1	0.283818431			-
cytochrome_aa3_600_menaquinol_oxidase_subunit_I	0.357789847			-
L_arabonate_dehydrase	0			-
nucleoside_deoxyribosyltransferase	0.440581982			-
aspartate_kinase	3.500852231			-
beta_galactosidase	3.207780596			-
aldehyde_dehydrogenase_NADP	0.516696243			-
anthranilate_synthasephosphoribosyltransferase	2.595820913			-
levanase	1.957010085			-
S_adenosylmethionine_diacylglycerol_3_amino_3_carboxypropyl_transferase	1.349386834			-
cellobiose_phosphorylase	0			-
cytochrome_c	3.131784846			-
general_L_amino_acid_transport_system_ATP_binding_protein	1.76738305			-
undecaprenyl_diphosphatase	2.673369781			-
putative_phosphotransacetylase	2.546452361	non-NJI	2.09923729	0.037373

Table S11. KEGG orthology functional terms identified by PICRUSt as different in neonatal jaundice infants (NJI) and non-NJI at 0 months (continued)

Biomaker_names	Logarithm value	Groups	LDA_value	P_value
serinethreonine_protein_phosphatase_Stp1	0			-
transaldolase	3.157355854			-
D_3_phosphoglycerate_dehydrogenase	3.428730021			-
cyclohexanone_monooxygenase	1.353280288			-
immunoglobulin_G_binding_protein_A	0			-
L_arabinose_transport_system_permease_protein	1.966474509			-
spermidineputrescine_transport_system_permease_protein	2.895797546			-
putative_multiple_sugar_transport_system_substrate_binding_protein	1.486771747			-
benzoatetoluate_1_2_dioxygenase_subunit_alpha	1.421466187			-
f_3_oxoacyl_acyl_carrier_protein_synthase_III	3.140692222			-
glycine_dehydrogenase_subunit_1	2.408405638			-
aureolysin	0			-
chaperonin_GroEL	3.384883622			-
photosystem_II_PsbU_protein	0			-
peptidoglycan_pentaglycine_glycine_transferase_the_fourth_and_fifth_glycine	0.343985124			-
RcsF_protein	2.47312454			-
f_2_hydroxy_3_oxopropionate_reductase	2.106001918			-
diacylglycerol_kinase_ATP	3.388214299			-
hydroxymethylglutaryl_CoA_synthase	2.223737131			-
glycine_dehydrogenase_subunit_2	2.408405638			-
two_component_system_OmpR_family_response_regulator_QseB	2.774520523			-
beta_fructofuranosidase	2.850120055			-
acetylornithine_deacetylase	3.097812404			-
para_aminobenzoate_synthetase	0.804149265			-
glyoxylate_reductase	1.353151953			-
N_acetylmannosamine_6_phosphate_2_epimerase_N_acetylmannosamine_kinase	0.394505937			-
lipoate___protein_ligase	2.549628939			-
bacteriochlorophyllide_a_dehydrogenase	0			-
f_4_alpha_glucanotransferase	2.365248182			-
serpin_B	0.638346196			0.0249747

Table S11. KEGG orthology functional terms identified by PICRUSt as different in neonatal jaundice infants (NJI) and non-NJI at 0 months (continued)

Biomaker_names	Logarithm value	Groups	LDA_value	P_value
UDP_glucose_4_epimerase	3.139997507			-
exodeoxyribonuclease_VII_small_subunit	2.647234366			-
N_acetylneuraminate_synthase	1.887587297			-
f_2_dehydro_3_deoxyphosphooctonate_aldolase_KDO_8_P_synthase	2.529970188			-
thymidine_phosphorylase	2.97016984			-
f_3_methylfumaryl_CoA_hydratase	1.517433581			-
cytochrome_c_oxidase_assembly_protein_subunit_15	2.177059034			-
aspartyl_tRNA_synthetase	2.662481654			-
cytochrome_c_oxidase_assembly_protein_subunit_11	1.563821647			-
type_IV_secretion_system_protein_VirB1	1.799715527			-
excinuclease_ABC_subunit_A	2.731501466			-
excinuclease_ABC_subunit_B	2.656364878			-
f_3_hydroxyacyl_CoA_dehydrogenase	2.399956617			-
exodeoxyribonuclease_V_beta_subunit	2.478755358			-
phosphocarrier_protein_HPr	2.475776406			-
lipid_A_ethanolaminephosphotransferase	1.970063732			-
two_component_system_chemotaxis_family_response_regulator_CheB	2.766489022			-
LPPG_FO_2_phospho_L_lactate_transferase	0.279368465			-
pectinesterase	2.281580727			-
mannonate_dehydratase	2.419341575			-
chorismate_mutase_prephenate_dehydrogenase	3.251342646			-
f_5_deoxynucleotidase	2.474202544			-
carbon_monoxide_dehydrogenase_small_subunit	2.773379479			-
two_component_system_CitB_family_sensor_histidine_kinase_CitS	0.878927699			-
malate_CoA_ligase_subunit_beta	1.331319353			-
UDP_N_acetylmuramate_dehydrogenase	2.959229913			-
para_aminobenzoate_synthetase_component_II	2.515536696			-
nitrogenase_delta_subunit	0			-
hyaluronoglucosaminidase	1.792687329			-
cobaltochelatase_CobN	1.506066446			-

Table S11. KEGG orthology functional terms identified by PICRUSt as different in neonatal jaundice infants (NJI) and non-NJI at 0 months (continued)

Biomaker_names	Logarithm value	Groups	LDA_value	P_value
DNA_directed_RNA_polymerase_subunit_alpha	3.133565029			-
D_glycero_alpha_D_manno_heptose_7_phosphate_kinase	0.127079152			0.0104056
cobalt_precorrin_7_C5_methyltransferase	1.790690821			-
two_component_system_sensor_histidine_kinase_FlrB	0.007555413			-
primosomal_replication_protein_N	2.752592009			-
proteasome_beta_subunit	0.577012702			-
putative_protease	2.906616898			-
acetyl_CoA_acyltransferase_2	0			-
PTS_system_D_glucosamine_specific_IIB_component	0.649807548			-
cobaltochelatase_CobS	1.417992905			-
cobaltochelatase_CobT	1.408415825			-
large_subunit_ribosomal_protein_L27	2.656694583			-
two_component_system_chemotaxis_family_sensor_kinase_CheA	2.775041588			-
large_subunit_ribosomal_protein_L25	2.569665682			-
large_subunit_ribosomal_protein_L24	2.65679429			-
large_subunit_ribosomal_protein_L23	2.656704161			-
large_subunit_ribosomal_protein_L22	2.656820677			-
large_subunit_ribosomal_protein_L21	2.65626739			-
phosphatidylethanolaminephosphatidyl_N_methylethanolamine_N_methyltransferase	0			-
ribulose_phosphate_3_epimerase	3.411377003			-
yersiniabactin_nonribosomal_peptide_synthetase	1.774296905			-
large_subunit_ribosomal_protein_L29	2.656301462			-
large_subunit_ribosomal_protein_L28	2.655301475			-
nitrogenase_molybdenum_iron_protein_beta_chain	2.102599941			-
f_5_oxopent_3_ene_1_2_5_tricarboxylate_decarboxylase_2_hydroxyhepta_2_4_diene_1_7_dioate_isomerase	3.15292465			-
p_hydroxybenzoate_3_monooxygenase	2.145394637			-
pyruvate_ferredoxin_oxidoreductase_gamma_subunit	1.576419826			-
succinate_semialdehyde_dehydrogenase	2.44227339			-
aliphatic_nitrilase	0.630308851			-
copper_chaperone	1.690248829			-

Table S11. KEGG orthology functional terms identified by PICRUSt as different in neonatal jaundice infants (NJI) and non-NJI at 0 months (continued)

Biomaker_names	Logarithm value	Groups	LDA_value	P_value
phycocyanobilin_lyase_subunit_beta	0			-
flavin_prenyltransferase	2.965812574			-
saccharopine_dehydrogenase_NAD_L_lysine_forming	2.586857502	non-NJI	2.12399104	0.0249747
peptide_chain_release_factor_subunit_1	0			-
dihydromonapterin_reductase_dihydrofolate_reductase	2.597430542			-
hexokinase	1.666176742			-
flagella_synthesis_protein_FlgN	2.383424843			-
PTS_system_lactose_specific_IIA_component	1.714363756			-
aldose_1_epimerase	2.778672535			-
PTS_system_mannitol_specific_IIC_component	2.365843033			-
transcription_termination_factor_Rho	2.575715897			-
PTS_system_sorbose_specific_IIC_component	2.079069318			-
two_component_system_NarL_family_vancomycin_resistance_sensor_histidine_kinase_VraS	0.358106428			-
arabinose_5_phosphate_isomerase	2.541513711			-
ring_1_2_phenylacetyl_CoA_epoxidase_subunit_PaaB	2.054702622			-
lactoseL_arabinose_transport_system_substrate_binding_protein	1.944576166			-
L_arabinonolactonase	1.772960523			-
thiopurine_S_methyltransferase	1.130205503			-
acetyl_CoA_decarbonylasesynthase_complex_subunit_delta	0.964380821			0.037373
sulfur_carrier_protein	2.540773148			-
xanthine_dehydrogenase_small_subunit	1.594354831			-
ribose_transport_system_substrate_binding_protein	3.151518982			-
DNA_cytosine_5_methyltransferase_1	3.124231785			-
DNA_polymerase_III_subunit_psi	3.171980305			-
two_component_system_response_regulator_RpfG	0			-
methyl_accepting_chemotaxis_protein_WspA	0.162059718			-
diaminohydroxyphosphoribosylaminopyrimidine_deaminase_5_amino_6_5_phosphoribosylaminouracil_reductase	3.203757646			-
sulfate_adenylyltransferase_subunit_2	3.155640674			-
flagellin	3.323436883			-
f_3_hydroxyanthranilate_3_4_dioxygenase	0.832058758			-

Table S11. KEGG orthology functional terms identified by PICRUSt as different in neonatal jaundice infants (NJI) and non-NJI at 0 months (continued)

Biomaker_names	Logarithm value	Groups	LDA_value	P_value
beta_1_4_mannosyl_glycoprotein_beta_1_4_N_acetylglucosaminyltransferase	1.549253953			-
hydroxypyruvate_reductase	1.954292612			-
DNA_primase	2.703513374			-
accessory_gene_regulator_B	2.232294847			-
sulfate_adenylyltransferase_subunit_1	3.124506311			-
secretion_monitor	2.774154536			-
menaquinone_dependent_protoporphyrinogen_oxidase	2.477038648			-
fumarate_hydratase_class_I	3.292124631			-
lysine_N6_hydroxylase	1.548717754			-
f_3_hydroxyacyl_acyl_carrier_protein_dehydratase	3.061658864			-
lia_operon_protein_LiaI	0			-
flagellar_transcriptional_activator_FlhD	2.86159852			-
f_3_phenylpropionatetrans_cinnamate_dioxygenase_ferredoxin_reductase_component	2.438389434			-
gamma_glutamyl_gamma_aminobutyraldehyde_dehydrogenase	1.843138539			-
methyl_accepting_chemotaxis_protein	3.268286274			-
flagellar_transcriptional_activator_FlhC	2.86159852			-
two_component_system_OmpR_family_copper_resistance_phosphate_regulon_response_regulator_CusR	2.546268292			-
L_ectoine_synthase	0.990924869			-
urea_transport_system_substrate_binding_protein	0			-
glutathionylspermidine_synthase	1.133709085			-
dihydropyrimidinase	2.776483769			-
glucosamine_6_phosphate_deaminase	2.669616536			-
diapolycopene_oxygenase	0.443960174			-
glycine_C_acetyltransferase	2.564624635			-
undecaprenyl_phosphate_4_deoxy_4_formamido_L_arabinose_transferase	2.77679912			-
formate_dehydrogenase_subunit_gamma	2.969834046			-
sigma_54_dependent_transcriptional_regulator_flagellar_regulatory_protein	0.086846415			-
monooxygenase	0.052637029			-
lycopene_cyclase_CruA	0			-
pyruvate_carboxylase_subunit_B	2.475860693	non-NJI	2.1225627	0.037373

Table S11. KEGG orthology functional terms identified by PICRUSt as different in neonatal jaundice infants (NJI) and non-NJI at 0 months (continued)

Biomaker_names	Logarithm value	Groups	LDA_value	P_value
N_carbamoylsarcosine_amidase	0.859748391			-
glutathione_transport_system_ATP_binding_protein	2.016570322			-
bifunctional_aspartokinase_homoserine_dehydrogenase_2	3.303375725			-
bifunctional_aspartokinase_homoserine_dehydrogenase_1	3.515527141			-
phosphoribosylanthranilate_isomerase	2.514554067			-
lycopene_cyclase_CruP	0			-
f_5_10_methylenetetrahydromethanopterin_reductase	0.326982903			-
butanol_dehydrogenase	2.636947065			-
photosystem_II_P680_reaction_center_D1_protein	0			-
aldehyde_dehydrogenase	2.096688356			-
chalcone_synthase	1.015508605			-
flagellar_hook_associated_protein_3_FlgL	2.403272562			-
aspartate_ammonia_lyase	2.549493429			-
glycerate_2_kinase	3.209842538			-
inhibitor_of_cysteine_peptidase	0.361076594			0.0102718
lysyl_tRNA_synthetase_class_II	2.658782513			-
citrate_lyase_subunit_beta_citryl_CoA_lyase	2.861717287			-
chlorophyllide_a_reductase_subunit_X	0			-
hexosaminidase	3.315591463			-
divinyl_protochlorophyllide_a_8_vinyl_reductase	0			-
undecaprenyl_phosphate_alpha_L_ara4FN_deformylase	2.77369682			-
thiamine_phosphate_diphosphorylase_hydroxyethylthiazole_kinase	0			-
chlorophyllide_a_reductase_subunit_Z	0			-
octopinenopaline_transport_system_permease_protein	1.294615067			-
dipeptide_transport_system_ATP_binding_protein	2.788950281			-
two_component_system_OmpR_family_aerobic_respiration_control_protein_ArcA	2.473210575			-
f_2_aminobenzoate_CoA_ligase	1.346688224			-
thiamine_phosphate_pyrophosphorylase	2.705532851			-
two_component_system_CitB_family_response_regulator_CitT	0.878927699			-
f_4_oxalmesaconate_hydratase	0.503534157			-

Table S11. KEGG orthology functional terms identified by PICRUSt as different in neonatal jaundice infants (NJI) and non-NJI at 0 months (continued)

Biomaker_names	Logarithm value	Groups	LDA_value	P_value
f_2_succinyl_5_enolpyruvyl_6_hydroxy_3_cyclohexene_1_carboxylate_synthase	2.519500775			-
two_component_system_CitB_family_response_regulator_CitB	2.586855421			-
cyclohexadienyl_dehydratase	1.751068978			-
methionyl_tRNA_formyltransferase	2.965912332			-
dihydroorotate_dehydrogenase_fumarate	2.746611351			-
iron_complex_transport_system_ATP_binding_protein	3.142442342			-
type_VI_secretion_system_secreted_protein_Hcp	2.975388344			-
arginine_transport_system_permease_protein	2.774213622			-
methicillin_resistance_protein	0.641956406			0.0163092
long_chain_fatty_acid___luciferin_component_ligase	2.073855361			-
acetyl_CoA_carboxylase_carboxyl_transferase_subunit_beta	3.414246836			-
vitamin_B12_transport_system_ATP_binding_protein	2.47311915			-
DNA_directed_RNA_polymerase_subunit_delta	2.103709652			-
stearoyl_CoA_desaturase_Delta_9_desaturase	1.891484363			-
pyruvate_water_dikinase	3.049340283			-
f_2_dehydro_3_deoxyglucarate_aldolase	1.967410791			-
hydroxymethylglutaryl_CoA_reductase	1.733790317			-
cysteine_synthase_A	3.367853143			-
rhamnose_transport_system_substrate_binding_protein	1.785779389			-
flagellar_hook_associated_protein_2	2.393988251			-
dehydrogluconokinase	0			-
PTS_system_D_glucosamine_specific_IIA_component	0.864548054			-
F_type_H_transporting_ATPase_subunit_beta	2.9572399			-
photosystem_I_4_8kDa_protein	0			-
formate__tetrahydrofolate_ligase	2.858242764			-
quinateshikimate_dehydrogenase	0.650519027			-
methylene_tetrahydromethanopterin_dehydrogenase	0.967306205			-
biphenyl_2_3_diol_1_2_dioxygenase	1.006620676			-
L_serine_dehydratase	3.509400396			-
cyclohexanecarboxyl_CoA_dehydrogenase	0			-

Table S11. KEGG orthology functional terms identified by PICRUSt as different in neonatal jaundice infants (NJI) and non-NJI at 0 months (continued)

Biomaker_names	Logarithm value	Groups	LDA_value	P_value
glucose_1_phosphate_thymidylyltransferase	3.130492258			-
dGTPase	2.626823499			-
succinylglutamate_desuccinylase	1.981053311			-
glutamate_racemase	2.656173168			-
taurine_dehydrogenase_large_subunit	0.817348629			-
f_6_kDa_early_secretory_antigenic_target	0.01114457			-
bifunctional_diaminopimelate_decarboxylase_aspartate_kinase	0.13744988			-
adenosylcobinamide_kinase_adenosylcobinamide_phosphate_guanylyltransferase	2.756091442			-
f_1_2_dihydroxy_3_keto_5_methylthiopentene_dioxygenase	2.266115079			-
f_2_oxoglutarate_dehydrogenase_E1_component	3.099139122			-
beta_ureidopropionase	2.091686773			-
thiamine_transport_system_substrate_binding_protein	2.480941619			-
adenosylhomocysteine_nucleosidase	2.877418204			-
exfoliative_toxin_AB	1.395797425			-
tryptophan_halogenase	0.942997535			-
f_4_hydroxybutyrate_dehydrogenase_sulfolactaldehyde_3_reductase	1.805954273			-
maleylacetate_reductase	0.764646589			-
arginineornithine_transport_system_permease_protein	0.914351658			-
hydroxybutyrate_dimer_hydrolase	0.508164444			-
nondiscriminating_glutamyl_tRNA_synthetase	1.667230253			-
DNA_polymerase_III_subunit_beta	3.355755797			-
superoxide_dismutase_Fe_Mn_family	3.658518283			-
nicotinate_nucleotide_pyrophosphorylase_carboxylating	2.572354233			-
D_citramalate_synthase	2.341683544			-
phosphatidate_cytidylyltransferase	3.107964507			-
mitochondrial_trans_2_enoyl_CoA_reductase	0			-
two_component_system_NtrC_family_nitrogen_regulation_response_regulator_NtrX	1.411256804			-
L_lactate_dehydrogenase_cytochrome	2.065591369			-
prolyl_oligopeptidase	1.169612794			-
two_component_system_NarL_family_invasion_response_regulator_UvrY	2.955660149			-

Table S11. KEGG orthology functional terms identified by PICRUSt as different in neonatal jaundice infants (NJI) and non-NJI at 0 months (continued)

Biomaker_names	Logarithm value	Groups	LDA_value	P_value
ornithine_carbamoyltransferase	2.928553584			-
threonyl_tRNA_synthetase	2.657296914			-
acetyl_CoApropionyl_CoA_carboxylase_biotin_carboxylase_biotin_carboxyl_carrier_protein	2.523033476			-
two_component_system_repressor_protein_LuxO	1.561605271			-
f_7_carboxy_7_deazaguanine_synthase	2.484465924			-
propionyl_CoA_synthetase	1.629617567			-
gamma_glutamyl_hercynylcysteine_S_oxide_hydrolase	2.492326458			-
chemotaxis_protein_MotA	2.983199082			-
nitric_oxide_synthase_bacterial	0.702665654			-
homospermidine_synthase	1.34008251			-
major_type_1_subunit_fimbrin_pilin	3.115537125			-
serine_O_acetyltransferase	3.434209948			-
ADP_L_glycero_D_manno_heptose_6_epimerase	2.482246404			-
sialic_acid_synthase	0			0.0131951
triphosphoribosyl_dephospho_CoA_synthase	2.556187432			-
amidophosphoribosyltransferase	3.00845911			-
PTS_system_sucrose_specific_IIA_component	0			-
L_aspartate_oxidase	2.872771889			-
UDP_N_acetylmuramate__alanine_ligase	2.959172062			-
sarcosine_oxidase_subunit_alpha	1.839840402			-
prolycopene_isomerase	0			-
DNA_3_methyladenine_glycosylase	1.983997972			-
methyl_accepting_chemotaxis_protein_I_serine_sensor_receptor	2.744691182			-
two_component_system_NarL_family_vancomycin_resistance_associated_response_regulator_VraR	0.493514959			-
zeta_carotene_desaturase	0			-
ethanolamine_ammonia_lyase_small_subunit	2.097931515			-
uridine_phosphorylase	2.892183448			-
omega_amidase	0			-
guanidinoacetate_N_methyltransferase	0			-
f_4_nitrophenyl_phosphatase	1.739473647			-

Table S11. KEGG orthology functional terms identified by PICRUSt as different in neonatal jaundice infants (NJI) and non-NJI at 0 months (continued)

Biomaker_names	Logarithm value	Groups	LDA_value	P_value
phosphoribosylformylglycinamidine_synthase	2.73394072			-
methylthioribose_1_phosphate_isomerase	2.202719724			-
two_component_system_CitB_family_response_regulator_MalR	0			-
lipoyloctanoyl_transferase	2.534778902			-
two_component_system_cell_cycle_response_regulator	1.477931255			-
N_acetylglucosamine_6_phosphate_deacetylase	2.738789419			-
teichoic_acid_transport_system_permease_protein	0.938650504			-
gluconolactonase	1.740850484			-
S_hydroxymethylglutathione_synthase	1.186490405			-
heptaprenyl_diphosphate_synthase	2.096998774			0.0249747
alpha_L_fucosidase	2.117705964			-
ubiquinone_biosynthesis_monooxygenase_Coq7	1.482207579			-
DNA_directed_RNA_polymerase_subunit_omega	3.07300425			-
oxepin_CoA_hydrolase_3_oxo_5_6_dehydrosuberyl_CoA_semialdehyde_dehydrogenase	2.05181915			-
argininosuccinate_synthase	3.101777218			-
large_subunit_ribosomal_protein_L34	2.64958465			-
large_subunit_ribosomal_protein_L35	2.656476855			-
nitroreductase	1.980266032			-
large_subunit_ribosomal_protein_L30	2.65611119			-
large_subunit_ribosomal_protein_L31	2.693112038			-
large_subunit_ribosomal_protein_L32	2.663788856			-
large_subunit_ribosomal_protein_L33	2.693908696			-
dolichyl_diphosphooligosaccharide__protein_glycosyltransferase	0			-
arginine_decarboxylase	2.731699215			-
quinoprotein_glucose_dehydrogenase	2.024805762			-
pantetheine_phosphate_adenylyltransferase	2.655902085			-
short_chain_Z_isoprenyl_diphosphate_synthase	0.992754443			-
cysteine_synthase_B	3.159744311			-
LEE_encoded_effector_EspG	0.209545046			-
malate_CoA_ligase_subunit_alpha	1.331319353			-

Table S11. KEGG orthology functional terms identified by PICRUSt as different in neonatal jaundice infants (NJI) and non-NJI at 0 months (continued)

Biomaker_names	Logarithm value	Groups	LDA_value	P_value
dihydropyrimidine_dehydrogenase_NADP	1.861445564			-
homoserine_kinase	2.835471735			-
pyrophosphate__fructose_6_phosphate_1_phosphotransferase	2.244869401			0.037373
D_serine_dehydratase	2.182175973			-
f_5_methyltetrahydropteroyltriglutamate__homocysteine_methyltransferase	3.19859642			-
all_trans_retinol_13_14_reductase	1.129802116			-
_trans_carveol_dehydrogenase	0			-
seryl_tRNA_synthetase	2.665336756			-
LuxR_family_transcriptional_regulator_quorum_sensing_system_regulator_SdiA	2.531403049			-
bicarbonate_transport_system_permease_protein	0			-
cyclohexa_1_5_dienecarbonyl_CoA_hydratase	0			-
lipid_IVA_palmitoyltransferase	2.774169763			-
glucose_1_phosphate_cytidylyltransferase	2.140429511			-
glutaryl_CoA_dehydrogenase	2.367403651			-
sn_glycerol_3_phosphate_transport_system_ATP_binding_protein	2.131077751			-
flagellar_P_ring_protein_precursor_FlgI	2.411856923			-
UDP_N_acetylglucosamine__N_acetylmuramyl_pentapeptide_pyrophosphoryl_undecaprenol_N_acetylglucosamine_transferase	3.134199087			-
putrescine_oxidase	0.621093761			-
biotin_transport_system_substrate_specific_component	2.25940113			-
PTS_system_galactosamine_specific_IIB_component	1.135697513			-
diaminopimelate_decarboxylase	2.968382835			-
invasin_D	0			-
f_4_5_dihydroxyphthalate_decarboxylase	1.273648938			-
ribonucleoside_diphosphate_reductase_subunit_M1	0			-
ribonucleoside_diphosphate_reductase_subunit_M2	0			-
lipopolysaccharide_transport_system_ATP_binding_protein	1.905439966			-
glutaconate_CoA_transferase_subunit_B	1.87941919			-
glutaconate_CoA_transferase_subunit_A	1.877512782			-
glycogen_synthase	0			-
PTS_system_sucrose_specific_IIC_component	2.45888331			-

Table S11. KEGG orthology functional terms identified by PICRUSt as different in neonatal jaundice infants (NJI) and non-NJI at 0 months (continued)

Biomaker_names	Logarithm value	Groups	LDA_value	P_value
erythronate_4_phosphate_dehydrogenase	2.519688177			-
ribose_transport_system_ATP_binding_protein	2.793362434			-
two_component_system_NarL_family_sensor_histidine_kinase_EvgS	1.177726946			-
ferredoxin	0			-
butyryl_CoA_dehydrogenase	2.92284489			-
DNA_directed_RNA_polymerase_subunit_beta	3.43489194			-
nitrogen_regulatory_protein_A	0.355350137			-
chemosensory_pili_system_protein_ChpC	0			-
enolase_phosphatase_E1	1.957331025			-
hydroxyquinol_1_2_dioxygenase	0.295031106			-
sulfate_transport_system_substrate_binding_protein	3.08348974			-
glycerol_3_phosphate_acyltransferase_PlsY	2.864658263			-
glycerol_3_phosphate_acyltransferase_PlsX	2.862196622			-
Lon_like_protease	1.73764874			-
D_alanine_transfer_protein	2.025459259			-
general_L_amino_acid_transport_system_substrate_binding_protein	1.884138515			-
pyrimidine_nucleoside_phosphorylase	2.073630464			-
phosphopantothenoylcysteine_decarboxylase	1.475951571			-
two_component_system_AgrA_family_response_regulator_AgrA	1.924118347			-
photosystem_I_subunit_XI	0			-
octaprenyl_diphosphate_synthase	2.534266249			-
outer_membrane_usher_protein	3.182177986			-
spermidineputrescine_transport_system_ATP_binding_protein	2.609585737			-
iron_regulated_surface_determinant_protein_A	0			-
adenosine_deaminase	2.810755681			-
purine_nucleosidase	2.709445827			-
PTS_system_cellobiose_specific_IIA_component	3.088750884			-
fused_signal_recognition_particle_receptor	3.133912408			-
acetyl_CoA_carboxylase_biotin_carboxylase_subunit	3.832176701			-
diguanylate_cyclase	1.443435769			-

Table S11. KEGG orthology functional terms identified by PICRUSt as different in neonatal jaundice infants (NJI) and non-NJI at 0 months (continued)

Biomaker_names	Logarithm value	Groups	LDA_value	P_value
f_15_cis_phytoene_desaturase	0			-
ribonuclease_HII	2.656264623			-
peptidyl_prolyl_cis_trans_isomerase_A_cyclophilin_A	2.524907363			-
pyochelin_synthetase	0			-
gamma_glutamyltranspeptidase_glutathione_hydrolase	3.135289086			-
PTS_system_mannose_specific_IIA_component	2.936984243			-
tRNA_dimethylallyltransferase	2.708491674			-
leucine_dehydrogenase	1.323688478			-
f_3_hydroxybutyrate_dehydrogenase	2.066230795			-
D_xylose_transport_system_ATP_binding_protein	2.004469923			-
formate_dehydrogenase_subunit_delta	1.51215024			-
oxalyl_CoA_decarboxylase	0.790432692			-
magnesium_protoporphyrin_IX_monomethyl_ester_oxidative_cyclase	1.718293544			-
methylmalonyl_CoAethylmalonyl_CoA_epimerase	2.307631613			-
two_component_system_OmpR_family_torCAD_operon_response_regulator_TorR	1.050632966			-
two_component_system_LytT_family_response_regulator_AlgR	1.121915389			-
f_3_5_cyclic_nucleotide_phosphodiesterase	0			-
thiamine_kinase	2.472319556			-
DNA_polymerase_III_subunit_alpha	3.357424481			-
signal_peptidase_I	3.159684549	non-NJI	2.36371392	0.0249747
formylmethanofuran_dehydrogenase_subunit_E	0.557113919			-
formylmethanofuran_dehydrogenase_subunit_A	0.969205988			-
formylmethanofuran_dehydrogenase_subunit_B	0.969205988			-
formylmethanofuran_dehydrogenase_subunit_C	0.969205988			-
dihydroneopterin_aldolase_2_amino_4_hydroxy_6_hydroxymethyldihydropteridine_diphosphokinase	2.067476883			-
malate_dehydrogenase_quinone	3.0345167			-
acyl_CoA_thioesterase_II	2.527968277			-
twitching_motility_two_component_system_response_regulator_PilG	1.451155404			-
phosphocarrier_protein_FPr	2.049398305			-
twitching_motility_two_component_system_response_regulator_PilH	1.448076219			-

Table S11. KEGG orthology functional terms identified by PICRUSt as different in neonatal jaundice infants (NJI) and non-NJI at 0 months (continued)

Biomaker_names	Logarithm value	Groups	LDA_value	P_value
haloacetate_dehalogenase	1.520760587			-
indole_3_glycerol_phosphate_synthase_phosphoribosylanthranilate_isomerase	3.075809386			-
f_4_hydroxy_4_methyl_2_oxoglutarate_aldolase	1.517039413			-
choloylglycine_hydrolase	2.900480442			-
mycobactin_peptide_synthetase_MbtF	0			-
mycobactin_peptide_synthetase_MbtE	0			-
R_R_butanediol_dehydrogenase_meso_butanediol_dehydrogenase_diacetyl_reductase	1.224372872			-
light_independent_protochlorophyllide_reductase_subunit_N	0			-
fumarylacetoacetase	1.256678391			-
beta_carotene_ketolase_CrtW_type	0			-
NADPH_quinone_oxidoreductase_subunit_4L	0			-
light_independent_protochlorophyllide_reductase_subunit_B	0			-
carboxymethylenebutenolidase	2.738984443			-
sulfite_dehydrogenase	0.433394567			-
D_alanyl_D_alanine_carboxypeptidase_D_alanyl_D_alanine_endopeptidase_penicillin_binding_protein_4	2.722047987			-
two_component_system_OmpR_family_aerobic_respiration_control_sensor_histidine_kinase_ArcB	2.473210575			-
alpha_glycerophosphate_oxidase	1.131120937			0.0249747
formate_dehydrogenase_iron_sulfur_subunit	2.969980007			-
allophanate_hydrolase	2.285494642			-
mannan_endo_1_4_beta_mannosidase	2.025540986			-
NADH_quinone_oxidoreductase_subunit_CD	2.493385645			-
heterodisulfide_reductase_subunit_A	0.627325544			-
precorrin_6Acobalt_precorrin_6A_reductase	2.10260071			-
competence_protein_ComK	0.380052877			-
glycerol_kinase	3.134811746			-
PTS_system_ascorbate_specific_IIC_component	3.04002626			-
glucose_1_phosphate_adenylyltransferase	2.957813167			-
competence_protein_ComQ	0			-
two_component_system_OmpR_family_sensor_histidine_kinase_VicK	1.628075568			-
propanediol_dehydratase_large_subunit	1.78668963			-

Table S11. KEGG orthology functional terms identified by PICRUSt as different in neonatal jaundice infants (NJI) and non-NJI at 0 months (continued)

Biomaker_names	Logarithm value	Groups	LDA_value	P_value
glutamate_5_semialdehyde_dehydrogenase	3.116690302			-
hemolysin_activationsecretion_protein	0.578895231			0.037373
chemotaxis_protein_methyltransferase_CheR	2.788015876			-
sarcosine_oxidase	0.951243988			-
processive_1_2_diacylglycerol_beta_glucosyltransferase	1.727133735			0.037373
coenzyme_F420_hydrogenase_subunit_beta	0.867975459			-
heptose_I_phosphotransferase	2.315310742			-
F_type_H_transporting_ATPase_subunit_a	2.963097997			-
sulfopyruvate_decarboxylase_subunit_alpha	0			-
UDP_N_acetylglucosamine_1_carboxyvinyltransferase	3.05995341	non-NJI	2.20193823	0.0249747
propionaldehyde_dehydrogenase	1.793808651			-
glycerol_3_phosphate_dehydrogenase	2.801369939			-
succinate_semialdehyde_dehydrogenase_glutarate_semialdehyde_dehydrogenase	3.407716658			-
ATP_binding_cassette_subfamily_C_bacterial_exporter_for_proteaselipase	0.594697946			-
nitrogenase_iron_protein_NifH	2.258588707			-
catalase	3.616527257			-
f_4_hydroxy_tetrahydrodipicolinate_reductase	3.145008303			-
kynurenine_3_monooxygenase	0			-
acyl_CoA_dehydrogenase	3.221620521			-
f_4_carboxymuconolactone_decarboxylase	2.932716981			-
cobalt_precorrin_6B_C15_methyltransferase	1.790741972			-
beta_alanine__pyruvate_transaminase	2.006160906			-
ribosylpyrimidine_nucleosidase	1.041957945			-
ironzincmanganesecopper_transport_system_permease_protein	0.994740385			-
two_component_system_NarL_family_response_regulator_DevR	0			-
glycerol_3_phosphate_dehydrogenase_subunit_C	2.471834817			-
glycerol_3_phosphate_dehydrogenase_subunit_B	2.471834817			-
glucan_1_3_beta_glucosidase	1.395083321			-
fructose_1_6_bisphosphatase_II_sedoheptulose_1_7_bisphosphatase	0.135711552			-
methionine_gamma_lyase	2.62809926			-

Table S11. KEGG orthology functional terms identified by PICRUSt as different in neonatal jaundice infants (NJI) and non-NJI at 0 months (continued)

Biomaker_names	Logarithm value	Groups	LDA_value	P_value
two_component_system_OmpR_family_phosphate_regulon_response_regulator_OmpR	2.487751233			-
two_component_system_NarL_family_sensor_histidine_kinase_RcsD	2.47312454			-
flagellar_hook_protein_FlgE	2.421359428			-
f_2_3_4_5_tetrahydropyridine_2_carboxylate_N_succinyltransferase	2.912248086			-
methanol_dehydrogenase_cytochrome_c_subunit_2	0			-
osmoprotectant_transport_system_ATP_binding_protein	2.327811836			-
f_4_hydroxybutyrate_dehydrogenase	0.550959663			-
double_stranded_uracil_DNA_glycosylase	1.976034022			-
cellulose_synthase_UDP_forming	2.561101309			-
UDP_N_acetylmuramoylalanine__D_glutamate_ligase	2.958027925			-
ribitol_5_phosphate_2_dehydrogenase	0			-
f_6_phosphogluconolactonase	2.875338919			-
cholera_toxin_transcriptional_activator	0			-
two_component_system_NtrC_family_nitrogen_regulation_sensor_histidine_kinase_NtrY	1.410915909			-
peroxisomal_2_4_dienoyl_CoA_reductase	0			-
N_acetylmuramic_acid_6_phosphate_etherase	2.53361465			-
sarcosine_dehydrogenase	1.523271427			-
glycine_oxidase	1.542415116			-
LEE_encoded_effector_Map	0			-
flotillin	1.530880909			-
AgrD_protein	0.593879471			-
two_component_system_NarL_family_sensor_histidine_kinase_NreB	0.394178043			-
two_component_system_CAI_1_autoinducer_sensor_kinasephosphatase_CqsS	0			-
benzoyl_CoA_reductase_subunit_D	0			-
benzoyl_CoA_reductase_subunit_A	0			-
f_3D_3_54_trihydroxycyclohexane_1_2_dione_acylhydrolase_decyclizing	2.027332342			-
benzoyl_CoA_reductase_subunit_C	0			-
benzoyl_CoA_reductase_subunit_B	0			-
mannose_1_phosphate_guanylyltransferase	2.711993284			-
f_2_3_dihydroxy_2_3_dihydrophenylpropionate_dehydrogenase	1.219062904			-

Table S11. KEGG orthology functional terms identified by PICRUSt as different in neonatal jaundice infants (NJI) and non-NJI at 0 months (continued)

Biomaker_names	Logarithm value	Groups	LDA_value	P_value
f_4_deoxy_L_threo_5_hexosulose_uronate_ketol_isomerase	1.996259555			-
DNA_polymerase_III_subunit_epsilon	3.477897625			-
aryl_sulfotransferase	0			-
trehalosemaltose_transport_system_permease_protein	0.705060179			-
succinylglutamic_semialdehyde_dehydrogenase	1.984542171			-
choline_dehydrogenase	2.580843964			-
f_2_3_dihydroxybenzoate_decarboxylase	1.217959071			-
carbamoyl_phosphate_synthase_small_subunit	2.956539863			-
pyochelin_biosynthesis_protein_PchD	0			-
glycine_betaineproline_transport_system_permease_protein	2.546741459			-
N_ethylmaleimide_reductase	2.524258999			-
homocitrate_synthase_NifV	1.798681036			-
type_IV_secretion_system_protein_VirD4	2.599978547			-
fructuronate_reductase	2.395756275			-
ribosome_biogenesis_GTPase_thiamine_phosphate_phosphatase	2.624704741			-
methylmalonyl_CoA_mutase_C_terminal_domain	1.146126135			-
MFS_transporter_NNP_family_nitratenitrite_transporter	2.604474749			-
autoinducer_2_binding_periplasmic_protein_LuxP	0			-
xanthine_dehydrogenase_YagS_FAD_binding_subunit	1.205341303			-
f_3_hydroxypropionyl_coenzyme_A_dehydratase	0			-
maltosemaltodextrin_transport_system_substrate_binding_protein	2.636638052			-
PTS_system_beta_glucoside_specific_IIB_component	0			-
f_1_4_dihydroxy_2_naphthoate_octaprenyltransferase	2.722648356			-
adenylosuccinate_lyase	2.964308938			-
YopJ_protease_family	0.093568814			-
polygalacturonase	0			-
phosphoribosyl_AMP_cyclohydrolase	2.172024546			-
f_3_vinyl_bacteriochlorophyllide_hydratase	0			-
aminopeptidase_N	2.577749954			-
f_2_hydroxy_6_oxonona_2_4_dienedioate_hydrolase	2.085045002			-

Table S11. KEGG orthology functional terms identified by PICRUSt as different in neonatal jaundice infants (NJI) and non-NJI at 0 months (continued)

Biomaker_names	Logarithm value	Groups	LDA_value	P_value
acyl_homoserine_lactone_synthase	0.595325618			-
oligogalacturonide_lyase	2.209193644			-
photosystem_I_subunit_IX	0			-
phosphoribosyl_ATP_pyrophosphohydrolase	2.170505127			-
stage_0_sporulation_protein_B_sporulation_initiation_phosphotransferase	0			-
phospholipidcholesterolgamma_HCH_transport_system_permease_protein	2.539998431			-
photosystem_I_subunit_IV	0			-
monoamine_oxidase	2.380273335			-
phycobilisome_core_linker_protein	0			-
two_component_system_CitB_family_cit_operon_sensor_histidine_kinase_CitA	2.586822199			-
f_5_6_7_8_tetrahydromethanopterin_hydro_lyase	0.97204017			-
ribonucleoside_triphosphate_reductase	2.990138329			-
histidine_transport_system_permease_protein	2.273966561			-
taurine_dioxygenase	2.288209297			-
f_2_aminoethylphosphonate_transport_system_ATP_binding_protein	1.77339211			-
PTS_system_ascorbate_specific_IIA_component	3.144661972			-
diacylglycerol_O_acyltransferase	1.025940395			-
PTS_system_ascorbate_specific_IIB_component	3.084233918			-
thiamine_transport_system_permease_protein	2.480896545			-
glutathione_peroxidase	2.830422174			-
methylenetetrahydrofolate_reductase_NADPH	3.199554559			-
type_IV_pili_sensor_histidine_kinase_and_response_regulator	1.132565618			-
aromatase	0			-
dolichyl_phosphate_beta_glucosyltransferase	0			-
photosystem_I_subunit_II	0			-
dimethylglycine_N_methyltransferase	0			-
formate_dehydrogenase	1.416656302			-
beta_carotene_ketolase_CrtO_type	0			-
acetyl_CoA_carboxylase_carboxyl_transferase_subunit_alpha	3.412278116			-
aldehyde_reductase	0			-

Table S11. KEGG orthology functional terms identified by PICRUSt as different in neonatal jaundice infants (NJI) and non-NJI at 0 months (continued)

Biomaker_names	Logarithm value	Groups	LDA_value	P_value
hydroxypyruvate_isomerase	2.441835791			-
two_component_system_sporulation_sensor_kinase_A	0			-
two_component_system_NarL_family_response_regulator_DegU	0			-
two_component_system_sporulation_sensor_kinase_E	0			-
PTS_system_fructose_specific_IIB_component	2.518215473			-
ferredoxin_nitrite_reductase	1.163551372			-
cobalt_precorrin_5B_C1_methyltransferase	2.234489738			-
anaerobic_magnesium_protoporphyrin_IX_monomethyl_ester_cyclase	0.309712043			0.0249747
NADH_quinone_oxidoreductase_subunit_H	2.507969607			-
phosphoglucosamine_mutase	2.598956203			-
putative_ABC_transport_system_ATP_binding_protein	0			0.0131951
alkanesulfonate_monooxygenase	2.244997076			-
two_component_system_cell_cycle_response_regulator_CpdR	1.696221394			-
bifunctional_oligoribonuclease_and_PAP_phosphatase_NrnA	2.255550716			-
precorrin_2_C20_methyltransferase_precorrin_3B_C17_methyltransferase	0.443073244			-
imidazolonepropionase	2.313278587			-
uridylate_kinase	2.656719309			-
flagellar_L_ring_protein_precursor_FlgH	2.411887093			-
putative_membrane_protein_PagO	0			-
plasmin_and_fibronectin_binding_protein_A	0			-
f_4_hydroxybenzoyl_CoA_thioesterase	1.781073075			-
sphingolipid_Delta_4_desaturase	0			-
dihydroorotate_dehydrogenase_electron_transfer_subunit	2.262061249			-
multidrug_resistance_protein_K	0.640326467			-
S_ureidoglycine_aminohydrolase	1.665672448			-
ATP_binding_cassette_subfamily_C_bacterial_RsaD	0			-
heptose_II_phosphotransferase	0.746638842			-
aspartate__ammonia_ligase	3.021103108			-
two_component_system_sensor_histidine_kinase_and_response_regulator	0			-
p_cymene_monooxygenase	0.325179446			-

Table S11. KEGG orthology functional terms identified by PICRUSt as different in neonatal jaundice infants (NJI) and non-NJI at 0 months (continued)

Biomaker_names	Logarithm value	Groups	LDA_value	P_value
heptosyltransferase_II	2.488305042			-
isocitrate_dehydrogenase	3.477903552			-
naphthalene_1_2_dioxygenase_ferredoxin_component	0.798104586			-
glucosylceramidase	2.56681849			-
chorismate_synthase	2.947952669			-
D_galactose_1_dehydrogenase	1.205906042			-
oligogalacturonide_transport_system_ATP_binding_protein	1.951717607			-
two_component_system_OmpR_family_response_regulator_BasR	2.774143755			-
PTS_system_N_acetylmuramic_acid_specific_IIB_component	0			-
phenylacetate_CoA_ligase	2.954750914			-
penicillin_binding_protein_2	2.900221599			-
cobIalamin_adenosyltransferase	2.580469439			-
f_7_cyano_7_deazaguanine_reductase	2.54060995			-
glycerol_3_phosphate_dehydrogenase_NADP	2.656456589			-
Delta7_sterol_5_desaturase	0			-
holdfast_attachment_protein_HfaB	0			-
holdfast_attachment_protein_HfaA	0.118474638			-
HTH_type_transcriptional_regulator_transcriptional_repressor_of_NAD_biosynthesis_genes	2.47333901			-
transitional_endoplasmic_reticulum_ATPase	1.713172404			-
crossover_junction_endodeoxyribonuclease_RuvC	2.608613114			-
f_5_hydroxyisourate_hydrolase	2.297746673			-
UDP_N_acetyl_D_galactosamine_dehydrogenase	2.092572378			-
type_IV_secretion_system_protein_VirB11	2.266646275			-
large_subunit_ribosomal_protein_L9	2.656738686			-
membrane_protein_involved_in_D_alanine_export	2.120968633			-
large_subunit_ribosomal_protein_L4	2.656719691			-
large_subunit_ribosomal_protein_L5	2.656408142			-
large_subunit_ribosomal_protein_L6	2.656638551			-
glutathione_independent_formaldehyde_dehydrogenase	1.571168114			-
signal_peptidase_II	2.670059603			-

Table S11. KEGG orthology functional terms identified by PICRUSt as different in neonatal jaundice infants (NJI) and non-NJI at 0 months (continued)

Biomaker_names	Logarithm value	Groups	LDA_value	P_value
LL_diaminopimelate_aminotransferase	2.423988535			0.037373
large_subunit_ribosomal_protein_L2	2.656783754			-
large_subunit_ribosomal_protein_L3	2.656752438			-
GDP_L_fucose_synthase	2.543674406			-
staphylococcal_complement_inhibitor	0			-
dimethylglycine_dehydrogenase	1.55735033			-
ATP_dependent_DNA_helicase_RecQ	2.733711565			-
f_2_iminoacetate_synthase	2.52079336			-
two_component_system_NarL_family_capsular_synthesis_sensor_histidine_kinase_RcsC	2.473695226			-
glucarate_dehydratase	1.975602864			-
bifunctional_UDP_N_acetylglucosamine_pyrophosphorylase_Glucosamine_1_phosphate_N_acetyltransferase	2.853897808			-
f_4_aminobutyrate_aminotransferase	2.593200388			-
ATP_dependent_DNA_helicase_RecG	2.770180706			-
formate_dehydrogenase_beta_subunit	0			-
anthranilate_1_2_dioxygenase_reductase_component	0			-
trimethylamine_N_oxide_reductase_cytochrome_c_cytochrome_c_type_subunit_TorC	0.658827117			-
fumarate_hydratase_subunit_beta	2.48208343	non-NJI	2.03421828	0.0249747
glutamin_asparagin_ase	0.872099838			-
ATP_dependent_RNA_helicase_RhlE	2.561539286			-
succinylarginine_dihydrolase	1.98342481			-
citrate_lyase_subunit_gamma_acyl_carrier_protein	2.191708027			-
L_ribulokinase	1.968446523			-
single_strand_DNA_binding_protein	3.517467653			-
salicylate_biosynthesis_isochorismate_synthase	0			-
methenyltetrahydromethanopterin_cyclohydrolase	0.966353188			-
ethylbenzene_dioxygenase_subunit_beta	0.33919131			-
MFS_transporter_NNP_family_putative_nitrate_transporter	0.343985124			-
DNA_polymerase_III_subunit_gammatau	3.350496103			-
flagellar_hook_associated_protein_1_FlgK	2.411189786			-
sn_glycerol_3_phosphate_transport_system_permease_protein	2.757085506			-

Table S11. KEGG orthology functional terms identified by PICRUSt as different in neonatal jaundice infants (NJI) and non-NJI at 0 months (continued)

Biomaker_names	Logarithm value	Groups	LDA_value	P_value
f_1_2_diacylglycerol_3_beta_galactosyltransferase	0			-
BlaI_family_transcriptional_regulator_methicillin_resistance_regulatory_protein	0			-
PTS_system_alpha_glucoside_specific_IIB_component	0			-
mannitol_1_phosphate_5_dehydrogenase	2.005787444			-
f_2_dehydro_3_deoxy_L_rhamnonate_aldolase	1.785480208			-
two_component_system_sporulation_sensor_kinase_B	0			-
GTP_cyclohydrolase_II	2.811818292			-
creatinase	1.655280805			-
f_2_aminoadipate_transaminase	0			-
CDP_4_dehydro_6_deoxyglucose_reductase_E3	1.431299499			-
CDP_4_dehydro_6_deoxyglucose_reductase_E1	0.210206494			-
deoxyribonuclease_IV	2.577994695			-
carotenoid_1_2_hydratase	0			-
translation_initiation_factor_eIF_2B_subunit_delta	0.238042493			-
glutamate_synthase_NADPHNADH_large_chain	3.217629738			-
PTS_system_cellobiose_specific_IIC_component	3.266938404			-
ADP_dependent_phosphofructokinaseglucokinase	0.658656366			0.0449512
chlorophyll_synthase	0			-
f_1_propanol_dehydrogenase	1.784915594			-
aspartyl_tRNAAsnglutamyl_tRNAGln_amidotransferase_subunit_B	2.47922205			-
hydroxymethylbilane_synthase	2.502585653			-
aspartyl_tRNAAsnglutamyl_tRNAGln_amidotransferase_subunit_A	2.639312126			-
thiazole_tautomerase_transcriptional_regulator_TenI	0.360337717			-
gamma_glutamyl_gamma_aminobutyrate_hydrolase	1.788209862			-
UDP_N_acetylglucosamine_acyltransferase	2.87398574			-
S_ribosylhomocysteine_lyase	3.153183486			-
squalene_hopenetetraprenyl_beta_curcumene_cyclase	0.289010406			-
malate_dehydrogenase_oxaloacetate_decarboxylatingNADP	3.012058439			-
photosystem_I_subunit_X	0			-
phospholipase_A1	3.201401162			-

Table S11. KEGG orthology functional terms identified by PICRUSt as different in neonatal jaundice infants (NJI) and non-NJI at 0 months (continued)

Biomaker_names	Logarithm value	Groups	LDA_value	P_value
f_3_dehydroquinate_dehydratase_shikimate_dehydrogenase	0			-
purine_binding_chemotaxis_protein_CheW	2.80888603			-
sucrose_phosphorylase	1.811425884			-
magnesium_chelatase_subunit_H	0			-
magnesium_chelatase_subunit_I	0.644226711			-
L_seryl_tRNASer_seleniumtransferase	2.961726147			-
f_3alphaor_20beta_hydroxysteroid_dehydrogenase	0.670448706			-
D_alanine__polyphosphoribitol_ligase_subunit_1	2.280299502			0.037373
two_component_system_response_regulator_YesN	2.257015163			0.0104056
D_alanine__polyphosphoribitol_ligase_subunit_2	2.191249599			-
magnesium_chelatase_subunit_D	1.098074008			-
regulator_of_sigma_E_protease	2.927447702			-
cytosine_deaminase	2.635424161			-
adenylate_kinase	2.966722938			-
two_component_system_phosphorelay_protein_LuxU	0			-
photosystem_II_13kDa_protein	0			-
f_2_polyprenyl_6_hydroxyphenyl_methylase_3_demethylubiquinone_9_3_methyltransferase	2.794247471			-
cobyrinic_acid_a_c_diamide_synthase	2.489375351			-
large_subunit_ribosomal_protein_L18	2.656832402			-
large_subunit_ribosomal_protein_L19	2.656783754			-
thiol_activated_cytolysin	1.224394801			-
large_subunit_ribosomal_protein_L17	2.656251726			-
large_subunit_ribosomal_protein_L14	2.656743528			-
pentachlorophenol_monooxygenase	0			-
urease_subunit_beta	2.496933023			-
large_subunit_ribosomal_protein_L13	2.656507707			-
large_subunit_ribosomal_protein_L10	2.656280218			-
large_subunit_ribosomal_protein_L11	2.656651112			-
f_2_oxoglutarate_ferredoxin_oxidoreductase_subunit_gamma	2.354460286			-
precorrin_6A_synthase	1.264649402			-

Table S11. KEGG orthology functional terms identified by PICRUSt as different in neonatal jaundice infants (NJI) and non-NJI at 0 months (continued)

Biomaker_names	Logarithm value	Groups	LDA_value	P_value
pyruvate_dehydrogenase_E1_component_alpha_subunit	3.039491459			-
triosephosphate_isomerase_TIM	3.456882847			-
acyl_lipid_omega_6_desaturase_Delta_12_desaturase	1.008011797			-
arylsulfatase_B	0			0.0131951
large_subunit_ribosomal_protein_L20	2.657814938			-
fructose_transport_system_permease_protein	1.071458253			-
two_component_system_OmpR_family_response_regulator_ResD	0.636394064			-
maltose_alpha_D_glucosyltransferase_alpha_amylase	2.061481854			-
corticosteroid_11_beta_dehydrogenase_isozyme_2	0			-
pyridoxine_4_dehydrogenase	0			-
anthraniloyl_CoA_monooxygenase	1.410682354			-
two_component_system_OmpR_family_sensor_histidine_kinase_PhoQ	2.774154536			-
two_component_system_OmpR_family_sensor_histidine_kinase_CreC	1.962321874			-
type_VI_secretion_system_secreted_protein_VgrG	2.633827536			-
proline_iminopeptidase	1.866060163			-
ectoine_hydroxylase	0.929209933			-
two_component_system_OmpR_family_response_regulator_CpxR	2.774314363			-
f_3_phenylpropionatetrans_cinnamate_dioxygenase_subunit_alpha	1.355596384			-
betaine_homocysteine_S_methyltransferase	0.455544519			-
f_2_dehydro_3_deoxygluconokinase	2.994051123			-
steroid_Delta_isomerase	2.084322025			-
elongation_factor_1_alpha	0			-
N_acetylglucosamine_transport_system_permease_protein	0.320699527			0.0144833
cephalosporin_C_deacetylase	1.509542972			0.0249747
ATP_binding_cassette_subfamily_B_bacterial_IrtA	0			-
ATP_binding_cassette_subfamily_B_bacterial_IrtB	0			-
hydrogen_cyanide_synthase_HcnB	0			-
photosystem_II_cytochrome_b559_subunit_beta	0			-
short_chain_fatty_acids_transporter	1.157857998			-
hydrogen_cyanide_synthase_HcnA	0			-

Table S11. KEGG orthology functional terms identified by PICRUSt as different in neonatal jaundice infants (NJI) and non-NJI at 0 months (continued)

Biomaker_names	Logarithm value	Groups	LDA_value	P_value
two_component_system_AgrA_family_response_regulator_ComE	1.339296473			-
flagellar_protein_FliOFliZ	2.385974765			-
serralysin	0			-
adenylylsulfate_kinase	2.820632303			-
dimethylaminetrimethylamine_dehydrogenase	1.257135232			-
isocitrate_lyase	2.788123906			-
acetyl_CoA_synthetase_ADP_forming	0.716041697			0.0463198
PTS_system_galactosamine_specific_IIC_component	1.049979163			-
geranylgeranyl_diphosphate_synthase_type_II	2.721186096			-
D_xylose_transport_system_permease_protein	2.005766085			-
cytochrome_c_oxidase_subunit_6a	0			-
f_8_amino_7_oxononanoate_synthase	2.571147773			-
aspartate_4_decarboxylase	2.311831699			0.037373
pyridoxal_5_phosphate_synthase_pdxS_subunit	1.540778278			-
heme_oxygenase_biliverdin_producing	0.851472279			-
PTS_system_maltoseglucose_specific_IIC_component	3.16580459			-
cyclohexadienyprephenate_dehydrogenase	2.126283136			-
two_component_system_OmpR_family_sensor_histidine_kinase_NblS	0			-
recombination_protein_RecA	2.658323342			-
general_L_amino_acid_transport_system_permease_protein	1.992919394			-
membrane_fusion_protein_RTX_toxin_transport_system	1.099365566			-
Fis_family_transcriptional_regulator_factor_for_inversion_stimulation_protein	2.479837215			-
immunoglobulin_G_binding_protein_Sbi	0			-
anaerobic_sulfite_reductase_subunit_C	1.846766832			-
CuIAgI_efflux_system_membrane_protein_CusASilA	2.085029209			-
recombination_protein_RecR	2.656894441			-
two_component_system_cell_cycle_response_regulator_DivK	1.747463343			-
L_fuculokinase	1.791982162			-
acetaldehyde_dehydrogenase	2.634909782			-
threonine_aldolase	2.89696401			-

Table S11. KEGG orthology functional terms identified by PICRUSt as different in neonatal jaundice infants (NJI) and non-NJI at 0 months (continued)

Biomaker_names	Logarithm value	Groups	LDA_value	P_value
DNA_mismatch_repair_protein_MutH	2.473911897			-
DNA_mismatch_repair_protein_MutL	2.631074108			-
DNA_mismatch_repair_protein_MutS	2.631139317			-
photosystem_I_subunit_III	0			-
formylmethanofuran__tetrahydromethanopterin_N_formyltransferase	0.966353188			-
UTP__glucose_1_phosphate_uridylyltransferase	3.269027613			-
cell_division_transport_system_permease_protein	2.629184812			-
aldehyde_dehydrogenase_NAD	3.522698448			-
DNA_ligase_NAD	3.438166284			-
type_III_secretion_protein_J	2.317483409			-
carbon_monoxide_dehydrogenase_iron_sulfur_subunit	0			-
type_III_secretion_protein_L	0.518914434			-
f_3_oxo_5_alpha_steroid_4_dehydrogenase_1	1.130571285			-
rhamnose_transport_system_ATP_binding_protein	1.78511303			-
type_III_secretion_protein_O	0			-
type_III_secretion_protein_C	2.313123811			-
pyruvate_ferredoxin_oxidoreductase_beta_subunit	1.531625881			-
type_III_secretion_protein_F	0			-
type_III_secretion_protein_X	0			-
photosystem_II_PsbJ_protein	0			-
D_threo_aldose_1_dehydrogenase	1.328554627			-
UDPglucose_6_dehydrogenase	3.147393651			-
type_III_secretion_protein_R	2.31531606			-
type_III_secretion_protein_S	2.316345125			-
fructokinase	3.380819164			-
type_III_secretion_protein_U	2.317483409			-
type_III_secretion_protein_V	2.31531606			-
type_III_secretion_protein_W	1.104043458			-
D_alanyl_D_alanine_carboxypeptidase	2.975887535			-
strictosidine_synthase	0			-

Table S11. KEGG orthology functional terms identified by PICRUSt as different in neonatal jaundice infants (NJI) and non-NJI at 0 months (continued)

Biomaker_names	Logarithm value	Groups	LDA_value	P_value
flagellar_basal_body_rod_protein_FlgC	2.484326921			-
flagellar_basal_body_rod_protein_FlgF	2.410080564			-
flagellar_basal_body_rod_protein_FlgG	2.490923822			-
homoserine_dehydrogenase	2.900568387			-
single_strand_selective_monofunctional_uracil_DNA_glycosylase	0			-
light_harvesting_protein_B_800_850_alpha_chain	0			-
scyllo_inosamine_4_phosphate_amidinotransferase_1	0			-
uridine_kinase	3.006704544			-
pyruvate_dehydrogenase_E1_component	3.095482966			-
transcription_repair_coupling_factor_superfamily_II_helicase	2.655320482			-
phosphatidylglycerol_lysyltransferase	1.93468741			-
two_component_system_NtrC_family_sensor_histidine_kinase_HydH	1.785982084			-
photosystem_I_subunit_XII	0			-
aconitate_hydratase_2_2_methylisocitrate_dehydratase	3.402817855			-
prolyl_tRNA_synthetase	2.65963907			-
glutamyl_tRNA_reductase	2.489648361			-
ethylmalonyl_CoA_mutase	1.428829113			-
secreted_effector_OspE	0			-
tryptophanase	2.425839777			-
chemotaxis_protein_methyltransferase_WspC	0			-
isoamylase	0			-
sulfite_reductase_NADPH_flavoprotein_alpha_component	2.485497415			-
aconitate_hydratase	3.433599496			-
imidazoleglycerol_phosphate_dehydratase	2.500575393			-
methyl_accepting_chemotaxis_protein_II_aspartate_sensor_receptor	2.727335196			-
D_ribulokinase	0			-
alpha_mannosidase	2.122812686			-
microsomal_epoxide_hydrolase	1.96635163			-
f_2_oxoisovalerate_dehydrogenase_E2_component_dihydrolipoyl_transacylase	1.923049771			-
menaquinol_cytochrome_c_reductase_cytochrome_b_subunit	0			-

Table S11. KEGG orthology functional terms identified by PICRUSt as different in neonatal jaundice infants (NJI) and non-NJI at 0 months (continued)

Biomaker_names	Logarithm value	Groups	LDA_value	P_value
aminomuconate_semialdehyde2_hydroxymuconate_6_semialdehyde_dehydrogenase	0.892349704			-
phosphate_butyryltransferase	1.520282254			-
glucoamylase	0.151415206			-
f_6_7_dimethyl_8_ribityllumazine_synthase	2.613690058			-
cobaltnickel_transport_protein	1.777077267			-
thiosulfate_sulfurtransferase	2.473342866			-
photosystem_II_oxygen_evolving_enhancer_protein_2	0			-
f_5_formyltetrahydrofolate_cyclo_ligase	2.652699138			0.037373
transaldolase_glucose_6_phosphate_isomerase	1.0532103			-
outer_membrane_protein	3.348486783			-
beta_glucuronidase	2.265702698			-
allantoate_deiminase	1.969080477			-
L_fucoseD_arabinose_isomerase	1.87913577			-
indolepyruvate_decarboxylase	2.47227092			-
D_xylose_transport_system_substrate_binding_protein	2.006360115			-
uridine_monophosphate_synthetase	0			-
two_component_system_NarL_family_nitratenitrite_response_regulator_NarP	2.318115502			-
GDP_mannose_6_dehydrogenase	0.195713245			-
ATP_dependent_Clp_protease_ATP_binding_subunit_ClpB	2.633373819			-
cyclohexanecarboxylate_CoA_ligase	0			-
ATP_dependent_Clp_protease_ATP_binding_subunit_ClpX	2.661926379			-
two_component_system_NarL_family_nitratenitrite_response_regulator_NarL	2.478714921			-
type_I_pantothenate_kinase	2.499061414			-
putative_peptidoglycan_lipid_II_flippase	2.544804779			-
phycobilisome_core_component	0			-
two_component_system_cell_cycle_sensor_histidine_kinase_and_response_regulator_CckA	1.707226041			-
nitrate_reductase_delta_subunit	2.343044624			-
D_arabinitol_4_dehydrogenase	2.081378042			-
two_component_system_NarL_family_response_regulator_NreC	0.644063502			-
histidine_transport_system_ATP_binding_protein	1.988301739			-

Table S11. KEGG orthology functional terms identified by PICRUSt as different in neonatal jaundice infants (NJI) and non-NJI at 0 months (continued)

Biomaker_names	Logarithm value	Groups	LDA_value	P_value
lipoyl_synthase	2.536156416			-
penicillin_binding_protein_1B	2.774532253			-
penicillin_binding_protein_1C	2.508294931			-
sialidase_1	2.403312603			-
f_2_oxoglutarate2_oxoacid_ferredoxin_oxidoreductase_subunit_alpha	2.919205546			-
nitric_oxide_reductase_subunit_B	1.446128605			-
nitric_oxide_reductase_subunit_C	1.332078552			-
two_component_system_NtrC_family_phosphoglycerate_transport_system_response_regulator_PgtA	1.103826805			-
riboflavin_synthase	2.594204462			-
f_3_oxoacyl_acyl_carrier_protein_synthase_II	3.196868821			-
acetoacetate_decarboxylase	0.90602472			-
acetyl_CoA_carboxylase_biotin_carboxyl_carrier_protein	3.416678883			-
putative_selenate_reductase_FAD_binding_subunit	0.651253857			-
type_VI_secretion_system_protein_ImpC	2.567859557			-
type_VI_secretion_system_protein_ImpB	2.567859557			-
type_VI_secretion_system_protein_ImpA	1.544135506			-
aspartate_racemase	2.426835533			-
type_VI_secretion_system_protein_ImpM	1.555721946			-
f_1_acyl_sn_glycerol_3_phosphate_acyltransferase	3.059706854			-
type_VI_secretion_system_protein_ImpK	2.631141528			-
type_VI_secretion_system_protein_ImpJ	2.627779549			-
type_VI_secretion_system_protein_ImpH	2.63243431			-
gluconokinase	2.802279592			-
clumping_factor_B	0			-
clumping_factor_A	0			-
fumarate_hydratase_subunit_alpha	2.498148624	non-NJI	2.05049647	0.0249747
histidinol_phosphate_aminotransferase	3.541139468			-
f_3_phosphoadenosine_5_phosphosulfate_synthase	0			-
oligogalacturonide_transport_system_substrate_binding_protein	1.953888762			-
argininosuccinate_lyase_amino_acid_N_acetyltransferase	0			-

Table S11. KEGG orthology functional terms identified by PICRUSt as different in neonatal jaundice infants (NJI) and non-NJI at 0 months (continued)

Biomaker_names	Logarithm value	Groups	LDA_value	P_value
f_3_isopropylmalateR_2_methylmalate_dehydratase_small_subunit	3.542768856			-
ubiquinol_cytochrome_c_reductase_cytochrome_b_subunit	2.500700924			-
glucose_6_phosphate_1_epimerase	2.543520372			-
threonine_dehydratase	3.380985771			-
bifunctional_non_homologous_end_joining_protein_LigD	1.802275985			-
cytochrome_P450_NADPH_cytochrome_P450_reductase	0.773624348			-
cobaltnickel_transport_system_permease_protein	2.895109754			-
endonuclease_VIII	2.308113662			-
f_3_oxoacid_CoA_transferase_subunit_B	2.133174934			-
f_3_oxoacid_CoA_transferase_subunit_A	2.123070704			-
amylosucrase	0.765104061			0.0249747
nicotinamide_nucleotide_amidase	2.832465906			-
sulfite_reductase_ferredoxin	0.562736985			-
thioredoxin_1	2.780444853			-
acyl_carrier_protein_S_malonyltransferase	2.942094386			-
two_component_system_NarL_family_sensor_histidine_kinase_DesK	1.633494123			-
D_sedoheptulose_7_phosphate_isomerase	2.483547916			-
sorbitol_6_phosphate_2_dehydrogenase	2.221737151			-
CDP_diacylglycerol___serine_O_phosphatidyltransferase	2.839879986			-
spermidine_synthase	2.691883928			-
protocatechuate_3_4_dioxygenase_beta_subunit	2.319817041			-
choline_phosphate_cytidylyltransferase	2.231821395			-
acetyl_CoA_decarbonylasesynthase_complex_subunit_gamma	0.964380821			0.037373
PTS_system_galactosamine_specific_IID_component	1.050757385			-
adenosylmethionine___8_amino_7_oxononanoate_aminotransferase	2.512462032			-
O_succinylhomoserine_sulfhydrylase	1.966080249			-
L_2_4_diaminobutyrate_decarboxylase	1.9609604			-
phosphotransacetylase	1.785480208			-
sn_glycerol_3_phosphate_transport_system_substrate_binding_protein	2.288523779			-
hemolysin	0			-

Table S11. KEGG orthology functional terms identified by PICRUSt as different in neonatal jaundice infants (NJI) and non-NJI at 0 months (continued)

Biomaker_names	Logarithm value	Groups	LDA_value	P_value
galactonate_dehydratase	1.997724365			-
cytochrome_b6_f_complex_subunit_8	0			-
branched_chain_amino_acid_aminotransferase	3.48382223			-
acetolactate_decarboxylase	2.400390814			-
cytochrome_b6	0			-
cytochrome_b6_f_complex_subunit_5	0			-
cytochrome_b6_f_complex_subunit_4	0			-
f_2_amino_4_deoxychorismate_synthase	0			-
fumarate_hydratase_class_II	3.290754167			-
pyruvate_carboxylase	2.351398277			-
acid_phosphatase	1.336987506			0.0104056
dihydroorotase	2.728220181			-
xylulokinase	2.555852515			-
acyl_acyl_carrier_protein_phospholipid_O_acyltransferase_long_chain_fatty_acid__acyl_carrier_protein_ligase	2.915662682	NJI	2.18180229	0.037373
UDP_4_amino_4_deoxy_L_arabinose_formyltransferase_UDP_glucuronic_acid_dehydrogenase_UDP_4_keto_hexauronic_acid_deca	3.074718788			-
N_acetyl_gamma_glutamyl_phosphate_reductase	3.330188961			-
hypoxanthine_phosphoribosyltransferase	2.956845456			-
flagellar_motor_switch_protein_FliG	2.779619487			-
f_2_ketocyclohexanecarboxyl_CoA_hydrolase	0.048508796			-
f_3_dehydro_L_gulonate_2_dehydrogenase	2.271734943			-
flagellar_motor_switch_protein_FliM	2.778674978			-
precorrin_6Y_C5_15_methyltransferase_decarboxylating	2.398964259			-
acyl_CoA_thioesterase	2.17812927			-
meso_butanediol_dehydrogenase_S_S_butanediol_dehydrogenase_diacetyl_reductase	2.667844758			-
f_1_pyrroline_5_carboxylate_dehydrogenase	2.062984888			-
two_component_system_NarL_family_sensor_histidine_kinase_LiaS	1.418114529			0.037373
N_acetylglucosamine_kinase	2.485811909			-
carboxylesterase_1	1.046010209			-
pyridoxine_5_phosphate_synthase	2.533424058			-
monofunctional_biosynthetic_peptidoglycan_transglycosylase	2.533279494			-

Table S11. KEGG orthology functional terms identified by PICRUSt as different in neonatal jaundice infants (NJI) and non-NJI at 0 months (continued)

Biomaker_names	Logarithm value	Groups	LDA_value	P_value
malonate_semialdehyde_dehydrogenase_acetylating_methylmalonate_semialdehyde_dehydrogenase	3.17357088			-
BlaI_family_transcriptional_regulator_penicillinase_repressor	0.278247817			-
starch_synthase	2.584754953			-
lipopolysaccharide_transport_system_permease_protein	1.893134323			-
alkanal_monooxygenase_alpha_chain	2.031394542			-
sirohydrochlorin_ferrochelatase	0			-
f_5_carboxymethyl_2_hydroxymuconate_isomerase	2.351410683			-
anaerobic_nitric_oxide_reductase_flavorubredoxin	1.960546377			-
chemotaxis_protein_MotC	1.336469111			-
f_2_oxoglutaramate_amidase	2.531383535			-
poly3_hydroxybutyrate_depolymerase	1.592768603			-
alpha_N_arabinofuranosidase	2.014367002			-
penicillin_binding_protein	1.895216822			-
accessory_colonization_factor_AcfC	0.020629212			-
accessory_colonization_factor_AcfA	0			-
aspartate_aminotransferase_chloroplastic	2.819930886			-
cytochrome_aa3_600_menaquinol_oxidase_subunit_III	0.304229834			-
accessory_colonization_factor_AcfD	0.640326467			-
AraC_family_transcriptional_regulator_mar_sox_rob_regulon_activator	2.180496513			-
light_harvesting_complex_1_beta_chain	0			-
leukocidinhemolysin_toxin_family_protein	0			-
S_ureidoglycine_glyoxylate_aminotransferase	1.966928381			-
competence_protein_ComX	1.875773734			0.037373
N_acetyllactosaminide_3_alpha_galactosyltransferase	0			0.0200963
L_2_4_diaminobutyric_acid_acetyltransferase	0.964460208			-
two_component_system_NtrC_family_C4_dicarboxylate_transport_response_regulator_DctD	1.659144954			-
protocatechuate_4_5_dioxygenase_alpha_chain	0.869359582			-
propanediol_dehydratase_small_subunit	1.812934514			-
dTMP_kinase	2.595495797			-
dihydroxyacetone_kinase_N_terminal_domain	2.482656886			-

Table S11. KEGG orthology functional terms identified by PICRUSt as different in neonatal jaundice infants (NJI) and non-NJI at 0 months (continued)

Biomaker_names	Logarithm value	Groups	LDA_value	P_value
manganeseiron_transport_system_substrate_binding_protein	2.429346568			-
nitric_oxide_dioxygenase	2.481804019			-
PTS_system_sucrose_specific_IIB_component	0.780252017			-
transglycosylase	0.343985124			-
xanthine_dehydrogenase_YagR_molybdenum_binding_subunit	1.23751431			-
nicotinamide_nucleotide_adenylyltransferase	0			-
orotidine_5_phosphate_decarboxylase	2.668568332			-
dihydrofolate_synthase_folylpolyglutamate_synthase	2.966097123			-
homoserine_O_succinyltransferase	3.070411554			-
excinuclease_ABC_subunit_C	2.656173448			-
tyrosyl_tRNA_synthetase	2.676505322			-
two_component_system_OmpR_family_sensor_histidine_kinase_TctE	2.075286492			-
limonene_1_2_monooxygenase	0			-
hydroxyethylthiazole_kinase	2.259816704			-
fructose_bisphosphate_aldolase_class_II	3.648003528			-
trehalose_6_phosphate_hydrolase	2.012330085			-
sodiumpotassium_transporting_ATPase_subunit_alpha	0			-
cholera_enterotoxin_subunit_A	0			-
butyrate_kinase	1.941241691			-
methyl_accepting_chemotaxis_protein_IV_peptide_sensor_receptor	2.674517104			-
bifunctional_NMN_adenylyltransferasenudix_hydrolase	1.390190587			-
mannuronan_5_epimerase	2.61212978			-
assimilatory_nitrate_reductase_catalytic_subunit	2.007334494			-
two_component_system_NtrC_family_response_regulator_AtoC	0.191415915			-
putrescine_transport_system_ATP_binding_protein	2.020534973			-
Fur_family_transcriptional_regulator_zinc_uptake_regulator	2.491570375			-
acetyl_CoA_acyltransferase	3.468410019	NJI	2.63040377	0.037373
fumarate_reductase_flavoprotein_subunit	3.449917192			-
threonine_3_dehydrogenase	2.489248111			-
evolved_beta_galactosidase_subunit_beta	2.225517917			-

Table S11. KEGG orthology functional terms identified by PICRUSt as different in neonatal jaundice infants (NJI) and non-NJI at 0 months (continued)

Biomaker_names	Logarithm value	Groups	LDA_value	P_value
gluconate_2_dehydrogenase	1.975265399			-
arginine_transport_system_substrate_binding_protein	2.590619161			-
alcohol_dehydrogenase_NADP	1.070589682			0.037373
MFS_transporter_SP_family_sugar_H_symporter	1.393707385			-
two_component_system_NarL_family_response_regulator_DesR	0.891890457			-
f_2_phosphosulfolactate_phosphatase	0.925711657			-
f_2_4_dichlorophenol_6_monooxygenase	0.722844304			-
L_arabinose_isomerase	2.056730201			-
allose_kinase	1.959441308			-
translocated_intimin_receptor	0			-
f_4_4_diapophytoene_synthase	0.292849475			-
two_component_system_OmpR_family_response_regulator_MprA	0.684524037			-
CDP_glucose_4_6_dehydratase	1.835252515			-
type_IV_secretion_system_protein_VirB10	1.97213611			-
gluconate_2_dehydrogenase_alpha_chain	0.827398226			-
enterobactin_synthetase_component_F	1.966170558			-
alpha_alpha_trehalase	2.292435112			-
inositol_oxygenase	0			-
heme_exporter_protein_C	2.448311928			-
selenide_water_dikinase	2.484447608			-
trehalose_synthase	0			0.0495102
CDP_diacylglycerol__inositol_3_phosphatidyltransferase	0			-
heme_exporter_protein_A	2.44823565			-
f_2_halobenzoate_1_2_dioxygenase_large_subunit	0			-
nitrate_reductase_nitrite_oxidoreductase_alpha_subunit	2.33783423			-
DNA_polymerase_III_subunit_delta	3.624441147			-
f_2_deoxy_D_gluconate_3_dehydrogenase	2.288521382			-
putatice_virulence_related_protein_PagC	0			-
nitrite_reductase_NO_forming	1.613233185			-
para_aminobenzoate_synthetase_4_amino_4_deoxychorismate_lyase	1.562257865			-

Table S11. KEGG orthology functional terms identified by PICRUSt as different in neonatal jaundice infants (NJI) and non-NJI at 0 months (continued)

Biomaker_names	Logarithm value	Groups	LDA_value	P_value
photosystem_II_P680_reaction_center_D2_protein	0			-
UDP_N_acetylmuramoyl_L_alanyl_D_glutamate__2_6_diaminopimelate_ligase	2.967138307			-
f_2_octaprenyl_6_methoxyphenol_hydroxylase	2.504814576			-
large_subunit_ribosomal_protein_L1	2.656600934			-
dihydroaeruginoic_acid_synthetase	0			-
manganeseiron_transport_system_ATP_binding_protein	2.429322718			-
polyphosphate_kinase	2.928561822			-
cytolysin_activating_lysine_acyltransferase	0			-
histidinol_dehydrogenase	2.9248451			-
urea_transport_system_ATP_binding_protein	0			-
pyruvate_carboxylase_subunit_A	0.211922308			-
small_subunit_ribosomal_protein_S14	2.678049961			-
aspartate_1_decarboxylase	2.828743055			-
fumarate_reductase_iron_sulfur_subunit	3.322214504			-
formate_dehydrogenase_N_gamma_subunit	2.471742883			-
glycyl_tRNA_synthetase_alpha_chain	2.508121056			-
cysteine_desulfurase_selenocysteine_lyase	2.916668666			-
ubiquinol_cytochrome_c_reductase_cytochrome_c_subunit	0.961348476			-
magnesium_protoporphyrin_O_methyltransferase	0			-
alkyldihydroxyacetonephosphate_synthase	0.85473863			-
f_3_methylcrotonyl_CoA_carboxylase_beta_subunit	1.713598211			-
thymidylate_synthase	3.129856402			-
DNA_replication_protein_DnaT	1.965016127			-
f_1_deoxy_D_xylulose_5_phosphate_synthase	2.914192401			-
aromatic_amino_acid_transaminase	3.393518215			-
F_type_H_transporting_ATPase_subunit_alpha	2.957263479			-
adenosylhomocysteinase	2.09064115			-
PepB_aminopeptidase	2.473210575			-
f_1_aminocyclopropane_1_carboxylate_deaminase	0.71550959			-
bifunctional_isochorismate_lyase_aryl_carrier_protein	2.021902038			-

Table S11. KEGG orthology functional terms identified by PICRUSt as different in neonatal jaundice infants (NJI) and non-NJI at 0 months (continued)

Biomaker_names	Logarithm value	Groups	LDA_value	P_value
DNA_3_methyladenine_glycosylase_I	2.562509062			-
UDP_N_acetyl_D_glucosamine_dehydrogenase	0.089928612			-
K_transporting_ATPase_ATPase_F_chain	0			-
glutamine_transport_system_substrate_binding_protein	2.201434639			-
arginyl_tRNA_synthetase	2.65662551			-
iron_complex_transport_system_substrate_binding_protein	3.210135628			-
aspartyl_protease_family_protein	1.760415665			-
hydrogenase_small_subunit	2.387530414			-
N_acetylmuramoyl_L_alanine_amidase	2.889702243			-
crotonyl_CoA_carboxylasereductase	1.430865972			-
tetrathionate_reductase_subunit_B	2.157580695			-
tetrathionate_reductase_subunit_C	2.157457364			-
PTS_system_lactose_specific_IIB_component	0			-
tetrathionate_reductase_subunit_A	2.157643667			-
RIO_kinase_1	1.041398293			-
f_6_phospho_3_hexuloisomerase	2.167111847			-
fructose_1_6_bisphosphatase_III	2.964748366	non-NJI	2.49824103	0.037373
zinc_transport_system_substrate_binding_protein	2.629407734			-
acyl_CoA_thioesterase_I	2.612888536			0.037373
f_6_phospho_beta_glucosidase	3.29367262			-
lactaldehyde_reductase	2.311868897			-
L_gulonate_5_dehydrogenase	1.965578763			-
two_component_system_OmpR_family_response_regulator_RegX3	1.43781683			-
f_2_oxo_hept_3_ene_1_7_dioate_hydratase	2.057212997			-
xanthine_dehydrogenase_molybdenum_binding_subunit	1.016028516			0.0249747
f_4_4_diaponeurosporenoate_glycosyltransferase	0.441292073			-
glycine_betaineproline_transport_system_ATP_binding_protein	2.547124163			-
phosphoadenosine_phosphosulfate_reductase	2.495736462			-
histidine_phosphotransferase_ChpT	1.707226041			-
intimin	0			-

Table S11. KEGG orthology functional terms identified by PICRUSt as different in neonatal jaundice infants (NJI) and non-NJI at 0 months (continued)

Biomaker_names	Logarithm value	Groups	LDA_value	P_value
f_3_phenylpropionatetrans_cinnamate_dioxygenase_ferredoxin_component	1.892337266			-
hydroxymethylpyrimidine_kinase_phosphomethylpyrimidine_kinase_thiamine_phosphate_diphosphorylase	1.042184618			-
N_acetylornithine_carbamoyltransferase	0			-
PTS_system_glucose_specific_IIB_component	2.552770001			-
two_component_system_autoinducer_2_sensor_kinasephosphatase_LuxQ	0			-
anthranilate_synthase_component_II	2.653025982			-
UDP_N_acetylglucosamine_2_epimerase_non_hydrolysing	3.003966893			-
CDP_paratose_2_epimerase	0.553973785			-
penicillin_binding_protein_1	0.648026183			-
malate_dehydrogenase_NADP	0			-
c_di_GMP_phosphodiesterase	1.897455673			-
K_transporting_ATPase_ATPase_B_chain	2.524439005			-
lipooligosaccharide_transport_system_ATP_binding_protein	0.823182326			-
phosphomethylpyrimidine_synthase	2.567687037			-
NADPH_quinone_oxidoreductase_subunit_I	0			-
PTS_system_glucitolsorbitol_specific_IIC_component	2.617756277			-
PTS_system_N_acetylgalactosamine_specific_IIB_component	2.678983607			-
NADPH_quinone_oxidoreductase_subunit_J	0			-
NADPH_quinone_oxidoreductase_subunit_M	0			-
NADPH_quinone_oxidoreductase_subunit_L	0			-
NADPH_quinone_oxidoreductase_subunit_N	0			-
two_component_system_OmpR_family_sensor_histidine_kinase_RstB	1.976554842			-
bacitracin_transport_system_ATP_binding_protein	0.619930947			-
salicylate_synthetase	2.081997943			-
type_II_pantothenate_kinase	1.754292794			-
NADPH_quinone_oxidoreductase_subunit_3	0			-
NADPH_quinone_oxidoreductase_subunit_2	0			-
two_component_system_chemotaxis_family_response_regulator_Rcp1	0			-
NADPH_quinone_oxidoreductase_subunit_4	0			-
two_component_system_cell_cycle_response_regulator_CtrA	1.707226041			-

Table S11. KEGG orthology functional terms identified by PICRUSt as different in neonatal jaundice infants (NJI) and non-NJI at 0 months (continued)

Biomaker_names	Logarithm value	Groups	LDA_value	P_value
NADPH_quinone_oxidoreductase_subunit_6	0			-
glutathione_transport_system_permease_protein	2.27574125			-
cytochrome_c6	0			-
lauroyl_Kdo2_lipid_IVA_myristoyltransferase	2.473221354			-
chitin_deacetylase	0.641221246			-
pyruvate_dehydrogenase_E2_component_dihydrolipoamide_acetyltransferase	3.212264212			-
exopolyphosphatase	1.16302173			-
flagellar_assembly_protein_FliH	2.44131406			-
flagellar_biosynthetic_protein_FlhB	2.410078416			-
penicillin_binding_protein_2X	1.787025449			-
nucleoside_triphosphatase	0			-
dihydropteroate_synthase	2.581496495			-
dTDP_4_dehydrorhamnose_3_5_epimerase	2.811114844			-
UDPglucose__hexose_1_phosphate_uridylyltransferase	3.033742221			-
two_component_system_NarL_family_nitratenitrite_sensor_histidine_kinase_NarX	2.478243643			-
putative_glutamine_transport_system_ATP_binding_protein	1.670603529			-
two_component_system_OmpR_family_response_regulator_PrrA	0			-
two_component_system_NarL_family_nitratenitrite_sensor_histidine_kinase_NarQ	1.519484013			-
phosphopentomutase	2.834512144			-
penicillin_binding_protein_2B	1.843647577			-
penicillin_binding_protein_2A	2.1496495			-
D_beta_D_heptose_7_phosphate_kinase_D_beta_D_heptose_1_phosphate_adenosyltransferase	2.780454005			-
molybdopterin_synthase_sulfur_carrier_subunit	2.793678252			-
f_2_dehydropantoate_2_reductase	2.646210272			-
phospholipid_transport_system_transporter_binding_protein	2.474712353			-
photosystem_II_cytochrome_c550	0			-
clavaminate_synthase	0			-
carbon_storage_regulator	2.933795459			-
f_2_pyrone_4_6_dicarboxylate_lactonase	1.478683347			-

Table S11. KEGG orthology functional terms identified by PICRUSt as different in neonatal jaundice infants (NJI) and non-NJI at 0 months (continued)

Biomaker_names	Logarithm value	Groups	LDA_value	P_value
acylglycerol_lipase	0			-
molybdate_transport_system_permease_protein	2.555959978			-
succinate_dehydrogenase_fumarate_reductase_flavoprotein_subunit	3.350549525			-
glutamateaspartate_transport_system_ATP_binding_protein	2.777630328			-
PTS_system_trehalose_specific_IIA_component	0			-
ironIII_transport_system_permease_protein	2.692636665			-
f_2_methylfumaryl_CoA_hydratase	1.715069832			-
mannopine_transport_system_substrate_binding_protein	0			-
FO_synthase_subunit_1	0			-
f_3_4_dihydroxy_2_butanone_4_phosphate_synthase_GTP_cyclohydrolase_II	2.606537744			-
NADPH_quinone_oxidoreductase_subunit_1	0			-
valine__pyruvate_aminotransferase	2.473267617			-
f_2_oxoisovalerate_dehydrogenase_E1_component	2.090104758			-
acetyl_CoA_C_acetyltransferase	3.984967469			-
putative_glutamine_transport_system_substrate_binding_protein	1.736151201			-
urease_subunit_alpha	2.622352563			-
dextransucrase	0.149826239			-
allantoicase	1.032318813			-
large_subunit_ribosomal_protein_L15	2.656714489			-
L_ribulose_5_phosphate_4_epimerase	2.570496615			-
NADPH_quinone_oxidoreductase_subunit_5	0.422473303			-
bifunctional_chitinaselysozyme	0			-
PTS_system_mannose_specific_IIB_component	3.246561417			-
two_component_system_OmpR_family_KDP_operon_response_regulator_KdpE	2.791016618			-
citrate_succinate_antiporter	0.776428847			-
lysophospholipase	2.519420389			-
f_1__4_alpha_D_glucan_1_alpha_D_glucosylmutase	1.9589415			-
phosphoenolpyruvate_carboxykinase_ATP	3.323037776			-
succinyl_CoA_synthetase_beta_subunit	3.23197835			-
fumarate_reductase_subunit_C	3.322214504			-
farnesyl_diphosphate_synthase	2.795375558			-

Table S11. KEGG orthology functional terms identified by PICRUSt as different in neonatal jaundice infants (NJI) and non-NJI at 0 months (continued)

Biomaker_names	Logarithm value	Groups	LDA_value	P_value
fumarate_reductase_subunit_D	3.322188541			-
D_alanine_transaminase	2.217363602			-
PTS_system_beta_glucoside_arbutinsalicincellobiose_specific_IIB_component	0			-
phycocyanobilin_ferredoxin_oxidoreductase	0			-
f_7_cyano_7_deazaguanine_synthase	2.532658131			-
aldehyde_ferredoxin_oxidoreductase	0.661894906			-
phospholipidcholesterolgamma_HCH_transport_system_substrate_binding_protein	2.492087593			-
methylmalonyl_CoA_carboxyltransferase_5S_subunit	0.302463052			-
methylmalonyl_CoA_mutase	2.980456832			-
two_component_system_NarL_family_response_regulator_EvgA	1.112405418			-
DNA_polymerase_beta	1.560513621			-
phospho_N_acetylmuramoyl_pentapeptide_transferase	2.958686448			-
exodeoxyribonuclease_VII_large_subunit	2.656161891			-
homogentisate_phytyltransferase_homogentisate_geranylgeranyltransferase	0			-
beta_mannosidase	1.636162904			-
pantoate_ligase_cytidylate_kinase	0			-
phosphogluconate_dehydratase	2.792358212			-
NADH_dehydrogenase_ubiquinone_Fe_S_protein_8	0			-
glycolate_oxidase_iron_sulfur_subunit	1.603457394			-
arylamine_N_acetyltransferase	0.645066631			-
f_2_succinyl_6_hydroxy_2_4_cyclohexadiene_1_carboxylate_synthase	2.47338259			-
menaquinol_cytochrome_c_reductase_iron_sulfur_subunit	0			-
D_allose_transport_system_substrate_binding_protein	1.952242688			-
C_terminal_binding_protein	0			-
ATP_dependent_Lon_protease	2.678015313			-
MFS_transporter_NRE_family_putaive_nickel_resistance_protein	1.406723133			-
geranyl_CoA_carboxylase_beta_subunit	0.369001554			-
nicotinamidasepyrazinamidase	2.679333767			0.0249747
propionate_kinase	1.650564269			-
glycyl_tRNA_synthetase	2.19484304			-

Table S11. KEGG orthology functional terms identified by PICRUSt as different in neonatal jaundice infants (NJI) and non-NJI at 0 months (continued)

Biomaker_names	Logarithm value	Groups	LDA_value	P_value
carboxylesterase_2	2.029270794			-
formate_dehydrogenase_alpha_subunit	0			-
nitronate_monooxygenase	1.991816808			-
phosphopantothenoylcysteine_decarboxylase_phosphopantothenate__cysteine_ligase	2.913168755			-
phosphate_transport_system_ATP_binding_protein	2.694653296			0.037373
salicylaldehyde_dehydrogenase	0.465743561			-
trehalose_6_phosphate_synthase	2.104643783			-
f_2_aminoethylphosphonate_transport_system_substrate_binding_protein	1.77339211			-
sodium_transport_system_permease_protein	1.190111392			0.0064853
ornithine__oxo_acid_transaminase	1.821445449			-
f_2_octaprenyl_3_methyl_6_methoxy_1_4_benzoquinol_hydroxylase	2.477862729			-
gentisate_1_2_dioxygenase	1.806639076			-
urea_carboxylase	2.262812143			-
nicotinate_nucleotide__dimethylbenzimidazole_phosphoribosyltransferase	2.324370836			-
glucose_6_phosphate_isomerase_archaeal	2.724640759			-
acetolactate_synthase_II_small_subunit	3.251361825			-
ribonuclease_III	2.95783949			-
dihydroxyacetone_kinase_C_terminal_domain	2.482710585			-
GTP_binding_protein_LepA	2.656854976			-
surface_protein_G	0			-
neutral_peptidase_B	0			-
acyl_acyl_carrier_protein_desaturase	0.560023994			-
glycine_betaineproline_transport_system_substrate_binding_protein	2.5636175			-
spermidineputrescine_transport_system_substrate_binding_protein	2.603234019			-
PTS_system_mannitol_specific_IIA_component	1.910899261			-
taurine_pyruvate_aminotransferase	0			-
dipeptidase_D	2.611290915			-
trans_2_3_dihydro_3_hydroxyanthranilate_isomerase	2.590905247			-
maleamate_amidohydrolase	0			-
alpha_D_ribose_1_methylphosphonate_5_triphosphate_diphosphatase	2.018158266			-

Table S11. KEGG orthology functional terms identified by PICRUSt as different in neonatal jaundice infants (NJI) and non-NJI at 0 months (continued)

Biomaker_names	Logarithm value	Groups	LDA_value	P_value
formyltetrahydrofolate_deformylase	2.818928128			-
cyclic_di_GMP_phosphodiesterase_flagellum_assembly_factor_TipF	1.112084933			-
gamma_glutamylputrescine_synthase	1.788767167			-
f_2_oxoglutarate2_oxoacid_ferredoxin_oxidoreductase_subunit_beta	2.929935419			-
f_3_oxoadipate_enol_lactonase_4_carboxymuconolactone_decarboxylase	1.296534978			-
f_3_oxosteroid_1_dehydrogenase	0.196439305			-
glucuronate_isomerase	2.261333655			-
f_5_amino_6_5_phosphoribosylaminouracil_reductase	0.046889298			-
VA_type_HNa_transporting_ATPase_subunit_D	2.19590301			-
fructose_1_6_bisphosphate_aldolasephosphatase	0.358774479			-
spermidine_dehydrogenase	1.156755479			-
enolase	3.485235476			-
VA_type_HNa_transporting_ATPase_subunit_E	2.193801571			-
ketoreductase	0			-
flagellar_biosynthetic_protein_FliR	2.410425875			-
glycerol_dehydratase_medium_subunit	0.269208628			-
precorrin_2cobalt_factor_2_C20_methyltransferase	2.474374178			-
adenylate_cyclase_class_1	2.77425135			-
adenylate_cyclase_class_2	1.106100087			-
putative_ABC_transport_system_permease_protein	0			-
PTS_system_alpha_glucoside_specific_IIC_component	2.277056346			-
VA_type_HNa_transporting_ATPase_subunit_K	2.195326087			-
ethylbenzene_dioxygenase_ferredoxin_component	0.051735201			-
aspartate_carbamoyltransferase_regulatory_subunit	2.853754201			-
CDP_L_myo_inositol_myo_inositolphosphotransferase	0			-
BirA_family_transcriptional_regulator_biotin_operon_repressor_biotin_acetyl_CoA_carboxylase_ligase	2.657774655			-
PTS_system_glucitolsorbitol_specific_IIB_component	0.647454661			-
perosamine_synthetase	0			-
pyridoxine_kinase	2.682973943			-
tungstate_transport_system_substrate_binding_protein	1.126463633			-

Table S11. KEGG orthology functional terms identified by PICRUSt as different in neonatal jaundice infants (NJI) and non-NJI at 0 months (continued)

Biomaker_names	Logarithm value	Groups	LDA_value	P_value
photosynthetic_reaction_center_M_subunit	0			-
xylose_isomerase	2.448004731			-
vitamin_B12_transport_system_substrate_binding_protein	2.47311915			-
propionyl_CoA_carboxylase_alpha_chain	2.065346506			-
catechol_2_3_dioxygenase	2.606045148			-
NADH_quinone_oxidoreductase_subunit_D	1.550241851			-
NADH_quinone_oxidoreductase_subunit_E	2.593309943			-
NADH_quinone_oxidoreductase_subunit_F	2.59074606			-
two_component_system_chemotaxis_family_response_regulator_CheY	2.836667936			-
NADH_quinone_oxidoreductase_subunit_A	2.507969607			-
NADH_quinone_oxidoreductase_subunit_B	2.50757285			-
NADH_quinone_oxidoreductase_subunit_C	1.549156602			-
NADH_quinone_oxidoreductase_subunit_L	2.507955554			-
NADH_quinone_oxidoreductase_subunit_M	2.507993788			-
NADH_quinone_oxidoreductase_subunit_N	2.507969607			-
two_component_system_chemotaxis_family_response_regulator_CheV	1.811623475			-
NADH_quinone_oxidoreductase_subunit_I	2.507955554			-
NADH_quinone_oxidoreductase_subunit_J	2.507969607			-
NADH_quinone_oxidoreductase_subunit_K	2.507969607			-
trans_polycis_decaprenyl_diphosphate_synthase	0			-
ipoprotein_LpqH	0			-
two_component_system_chemotaxis_family_sensor_histidine_kinase_and_response_regulator_WspE	0.192604332			-
exopolyphosphatase_guanosine_5_triphosphate_3_diphosphate_pyrophosphatase	3.078549293			-
fibronectin_binding_protein_1	0			-
malate_synthase	2.979911618			-
O_succinylbenzoic_acid__CoA_ligase	2.59052491			-
protein_Map	0			-
thymidylate_synthase_FAD	1.437490974			0.0039478
PTS_system_fructose_specific_IID_component	2.27681717			-
ironIII_transport_system_ATP_binding_protein	2.632888312			-

Table S11. KEGG orthology functional terms identified by PICRUSt as different in neonatal jaundice infants (NJI) and non-NJI at 0 months (continued)

Biomaker_names	Logarithm value	Groups	LDA_value	P_value
dipeptide_transport_system_permease_protein	2.787268308			-
phosphatidylserine_decarboxylase	2.538155278			-
cysteine_desulfurase	3.085219663			-
replication_factor_A1	0			-
UDP_N_acetylmuramoyl_L_alanyl_D_glutamate_L_lysine_ligase	1.382028296			-
methylmalonyl_CoA_mutase_N_terminal_domain	1.231001554			-
ethanolamine_utilization_protein_EutA	1.788612904			-
f_3_hydroxyisobutyrate_dehydrogenase	2.17085041			-
alpha_glucoside_transport_system_permease_protein	1.658236974			-
maltose_phosphorylase	1.091915847			0.0249747
f_3_hydroxypropanoate_dehydrogenase	1.973995134			-
AI_2_transport_system_ATP_binding_protein	1.95651297			-
aspartyl_tRNAAsnglutamyl_tRNAGln_amidotransferase_subunit_C	2.488327213			-
cobaltnickel_transport_system_ATP_binding_protein	2.936015163			-
f_6_phosphofructokinase_1	3.857676343			-
gamma_butyrobetaine_dioxygenase	1.053436254			-
sodium_transport_system_ATP_binding_protein	1.481128998			0.0104056
methyl_accepting_chemotaxis_protein_III_ribose_and_galactose_sensor_receptor	1.810465757			-
glucan_endo_1_3_beta_D_glucosidase	0			-
f_1_phosphofructokinase	2.579015707			-
thymidine_kinase	2.899922806			-
isocitrate_dehydrogenase_NAD	1.680773951			-
UDP_3_O_3_hydroxymyristoyl_N_acetylglucosamine_deacetylase	2.535984338			-
fibronectin_binding_protein_B	0			-
fibronectin_binding_protein_A	0			-
two_component_system_OmpR_family_catabolic_regulation_response_regulator_CreB	1.962321874			-
cytidylate_kinase	2.710865558			-
two_component_system_OmpR_family_sensor_histidine_kinase_CpxA	2.774272947			-
glycine_cleavage_system_H_protein	2.870695266			-
vibriolysin	0			-

Table S11. KEGG orthology functional terms identified by PICRUSt as different in neonatal jaundice infants (NJI) and non-NJI at 0 months (continued)

Biomaker_names	Logarithm value	Groups	LDA_value	P_value
f_3_dehydroquinate_dehydratase_II	2.892159066			-
two_component_system_NtrC_family_phosphoglycerate_transport_system_sensor_histidine_kinase_PgtB	1.103826805			-
putative_glutamine_transport_system_permease_protein	1.771025511			-
histidine_transport_system_substrate_binding_protein	1.96528797			-
two_component_system_cell_cycle_sensor_histidine_kinase_DivJ	1.654815242			-
f_2_3_dihydroxyethylbenzene_1_2_dioxygenase	0.051735201			-
HSP20_family_protein	2.220890815			-
NAD_synthase	2.060866462			-
tartronate_semialdehyde_synthase	1.523727712			-
lipopolysaccharide_export_system_ATP_binding_protein	2.536710987			-
zinc_resistance_associated_protein	1.785982084			-
photosystem_II_PsbK_protein	0			-
XTPdITP_diphosphohydrolase	2.565146484			-
putative_selenate_reductase	1.793587445			-
f_3_dehydro_L_gulonate_6_phosphate_decarboxylase	2.267563279			-
saccharopine_dehydrogenase_NADP_L_glutamate_forming	0			-
linalool_8_monooxygenase	0.212271676			-
uroporphyrin_III_C_methyltransferase	2.50357876			-
transmembrane_regulatory_protein_ToxS	0			-
two_component_system_OmpR_family_sensor_histidine_kinase_TorS	1.038017075			-
NADH_dehydrogenase	2.623984467			-
f_3_hydroxyacyl_acyl_carrier_protein_dehydratase_trans_2_decenoyl_acyl_carrier_protein_isomerase	2.79193093			-
methanol_dehydrogenase_cytochrome_c_subunit_1	0			-
photosystem_II_PsbL_protein	0			-
aryl_alcohol_dehydrogenase	1.154849236			-
two_component_system_OmpR_family_response_regulator_NblR	0			-
alcohol_dehydrogenase_propanol_preferring	2.965268843			-
PTS_system_cellobiose_specific_IIB_component	3.201030008			-
f_2_dehydro_3_deoxyphosphogalactonate_aldolase	2.20512421			-
tagaturonate_reductase	2.235879			-

Table S11. KEGG orthology functional terms identified by PICRUSt as different in neonatal jaundice infants (NJI) and non-NJI at 0 months (continued)

Biomaker_names	Logarithm value	Groups	LDA_value	P_value
glutamate_dehydrogenase_NADP	3.208986513			-
glutathione_synthase	2.794182637			-
nitrite_reductase_cytochrome_c_552	2.761804447			-
PTS_system_maltoseglucose_specific_IIB_component	1.713613025			-
glutamate_synthase_NADPHNADH_small_chain	3.463787762			-
preprotein_translocase_subunit_SecF	2.794295019			-
preprotein_translocase_subunit_SecG	3.132581897			-
preprotein_translocase_subunit_SecD	2.786790192			-
preprotein_translocase_subunit_SecE	3.130110763			-
preprotein_translocase_subunit_SecB	2.970807772			-
preprotein_translocase_subunit_SecA	3.152972745			-
pilus_assembly_protein_FlpPilA	2.052769951			-
general_secretion_pathway_protein_S	2.074017546			-
general_secretion_pathway_protein_N	2.07975062			-
general_secretion_pathway_protein_O	2.290907649			-
general_secretion_pathway_protein_L	2.527974575			-
general_secretion_pathway_protein_M	2.518750277			-
general_secretion_pathway_protein_J	2.528030189			-
general_secretion_pathway_protein_K	2.528108002			-
general_secretion_pathway_protein_H	2.527573374			-
general_secretion_pathway_protein_I	2.519429778			-
general_secretion_pathway_protein_F	2.530386424			-
general_secretion_pathway_protein_G	2.53421818			-
general_secretion_pathway_protein_D	2.540764819			-
general_secretion_pathway_protein_E	2.532718156			-
general_secretion_pathway_protein_C	2.518187309			-
phosphate_acetyltransferase	3.41760456			-
homogentisate_1_2_dioxygenase	1.833902597			-
osmoprotectant_transport_system_substrate_binding_protein	2.300552181			-
indole_3_glycerol_phosphate_synthase	2.417492356			-

Table S11. KEGG orthology functional terms identified by PICRUSt as different in neonatal jaundice infants (NJI) and non-NJI at 0 months (continued)

Biomaker_names	Logarithm value	Groups	LDA_value	P_value
nitrilase	2.136463165			-
PTS_system_2_O_A_mannosyl_D_glycerate_specific_IIC_component	1.527986972			-
f_2_4_dihydroxyacetophenone_dioxygenase	1.087964443			-
hydroxymethylpyrimidinephosphomethylpyrimidine_kinase	2.939352199			-
MbtH_protein	0.056282223			-
ferrochelatase	2.503107142			-
cytochrome_c_oxidase_cbb3_type_subunit_II	1.965642447			-
cobalamin_biosynthesis_protein_CobW	1.581332599			-
aminobutyraldehyde_dehydrogenase	2.287785165			-
beta_glucosidase	3.380159352			-
putative_spermidineputrescine_transport_system_permease_protein	2.906018721			-
PTS_system_fructose_specific_IIA_component	2.82730074			-
alanine_glyoxylate_transaminase_serine_glyoxylate_transaminase_serine_pyruvate_transaminase	2.663855827			-
minimal_PKS_acyl_carrier_protein	0			-
flagellar_hook_length_control_protein_FliK	2.384692149			-
two_component_system_OmpR_family_sensor_histidine_kinase_PrrB	0			-
raffinosestachyosemelibiose_transport_system_permease_protein	1.952201341			-
DNA_end_binding_protein_Ku	1.391551245			-
f_2_C_methyl_D_erythritol_2_4_cyclodiphosphate_synthase	2.591492236			-
lipoprotein_releasing_system_permease_protein	2.828730183			-
two_component_system_CitB_family_sensor_histidine_kinase_DcuS	1.965325726			-
myo_inositol_1or_4_monophosphatase	3.16856334			-
two_component_system_OmpR_family_sensor_histidine_kinase_MprB	0.559115349			-
two_component_system_OmpR_family_sensor_histidine_kinase_CssS	0.318754474			-
lipid_A_disaccharide_synthase	2.536020183			-
valine_dehydrogenase_NAD	0			-
FO_synthase_subunit_2	0			-
type_IV_pilus_assembly_protein_PilA	1.602048984			-
cyclomaltodextrin_glucanotransferase	0			-
two_component_system_OmpR_family_manganese_sensing_response_regulator	0			-

Table S11. KEGG orthology functional terms identified by PICRUSt as different in neonatal jaundice infants (NJI) and non-NJI at 0 months (continued)

Biomaker_names	Logarithm value	Groups	LDA_value	P_value
type_IV_pilus_assembly_protein_PilK	0			-
inosine_kinase	2.473097586			-
acetate_CoAacetoacetate_CoA_transferase_alpha_subunit	1.253262696			-
enterobactin_synthetase_component_D	1.960354496			-
neutral_amino_acid_transport_system_substrate_binding_protein	0			-
propanediol_dehydratase_medium_subunit	1.79877395			-
glutamate_5_kinase	3.117277628			-
malonyl_CoA_O_methyltransferase	2.526120573			-
aspartate_carbamoyltransferase_catalytic_subunit	2.956741783			-
tyrosine_phenol_lyase	0			-
f_60_kDa_SS_ARo_ribonucleoprotein	0.858461006			-
phospholipid_transport_system_substrate_binding_protein	2.480394674			-
f_2_isopropylmalate_synthase	3.292051939			-
N_glycosylaseDNA_lyase	1.911760595			0.0064853
cell_division_protein_FtsQ	2.588330113			-
propionate_CoA_transferase	2.412917589			-
PTS_system_N_acetylmuramic_acid_specific_IIC_component	2.72606411			-
tagatose_6_phosphate_kinase	2.492068295			-
exodeoxyribonuclease_I	2.485481232			-
UDP_2_acetamido_2_deoxy_ribo_hexuluronate_aminotransferase	0.084157247			-
cell_division_protein_FtsZ	2.666611002			-
two_component_system_LuxR_family_sensor_histidine_kinase_DctS	0.318992503			-
cell_division_protein_FtsA	2.548346965			-
ATP_binding_cassette_subfamily_C_bacterial_competence_factor_transporting_protein	1.248142988			0.0200963
quinate_dehydrogenase_quinone	2.187727018			-
fructose_transport_system_substrate_binding_protein	1.073030165			-
exodeoxyribonuclease_X	1.970754952			-
f_6_hydroxynicotinate_3_monooxygenase	0			-
alkaline_phosphatase	3.221435562			-
microcin_C_transport_system_permease_protein	2.323214512			-

Table S11. KEGG orthology functional terms identified by PICRUSt as different in neonatal jaundice infants (NJI) and non-NJI at 0 months (continued)

Biomaker_names	Logarithm value	Groups	LDA_value	P_value
delta_hemolysin	0			-
F_type_H_transporting_ATPase_subunit_delta	2.956440503			-
f_3_hydroxypropionate_dehydrogenase_NADP	0			-
ring_1_2_phenylacetyl_CoA_epoxidase_subunit_PaaE	2.054849045			-
ring_1_2_phenylacetyl_CoA_epoxidase_subunit_PaaD	2.054702622			-
ring_1_2_phenylacetyl_CoA_epoxidase_subunit_PaaC	2.054702622			-
citronellyl_CoA_dehydrogenase	0.420423866			-
ring_1_2_phenylacetyl_CoA_epoxidase_subunit_PaaA	2.054702622			-
DNA_mismatch_repair_protein_MutS2	2.248730753			-
two_component_system_NtrC_family_sensor_histidine_kinase_PilS	1.142321217			-
carbon_monoxide_dehydrogenase_medium_subunit	2.260336297			-
exodeoxyribonuclease_V_gamma_subunit	2.478766			-
guanylate_kinase	2.665813803			-
outer_membrane_pore_protein_C	2.27059628			-
thiosulfate_reductase_cytochrome_b_subunit	1.155615198			-
outer_membrane_pore_protein_F	2.326010007			-
uroporphyrinogen_III_methyltransferase_synthase	2.029455621			0.0249747
dCMP_deaminase	2.262276941			-
oxaloacetate_decarboxylase	0.618744495			-
DNA_repair_protein_RecO_recombination_protein_O	2.654434955			-
glutamate_formiminotransferase_formiminotetrahydrofolate_cyclodeaminase	0.463946049			-
PadR_family_transcriptional_regulator_regulatory_protein_AphA	0			-
f_3_oxoadipyl_CoA_thiolase	0			-
maltogenic_alpha_amylase_160	0			-
vitamin_B12_transport_system_permease_protein	2.47311915			-
xanthine_dehydrogenase_YagT_iron_sulfur_binding_subunit	1.214338435			-
urease_subunit_gammabeta	0			-
sulfite_oxidase	0			-
penicillin_binding_protein_1A	3.1735532			-
naphthalene_1_2_dioxygenase_ferredoxin_reductase_component	0.780902556			-

Table S11. KEGG orthology functional terms identified by PICRUSt as different in neonatal jaundice infants (NJI) and non-NJI at 0 months (continued)

Biomaker_names	Logarithm value	Groups	LDA_value	P_value
phycobilisome_rod_core_linker_protein	0			-
glycerol_3_phosphate_cytidylyltransferase	1.828059088			-
L_tartrate_dehydratase_alpha_subunit	1.79590624			-
NADP_transhydrogenase_subunit_alpha	2.570581719			-
ketoreductase_RED1	0.155226456			-
f_5_6_dimethylbenzimidazole_synthase	1.535932402			-
benzoatetoluate_1_2_dioxygenase_reductase_component	1.306285754			-
biotinmethionine_sulfoxide_reductase	1.965276699			-
DNA_3_methyladenine_glycosylase_II	2.491362918			-
raffinosestachyosemelibiose_transport_system_substrate_binding_protein	1.628974671			-
GTP_pyrophosphokinase	2.698658437			-
O_antigen_ligase	2.376410479			-
homoserine_kinase_type_II	1.968395935			-
cell_division_protein_FtsI_penicillin_binding_protein_3	2.965906712			-
staphylococcal_enterotoxin	0.339166168			-
aspartate_dehydrogenase	1.71900051			-
flagellar_rod_protein_FlaI	0			-
GDPmannose_4_6_dehydratase	2.541926307			-
f_5_dehydro_4_deoxyglucarate_dehydratase	0.585532353			-
phosphoenolpyruvate_carboxylase	3.229618301			-
large_subunit_ribosomal_protein_L16	2.65677272			-
maltooligosyltrehalose_trehalohydrolase	0.821900768			-
ATP_binding_cassette_subfamily_B_bacterial_RtxE	1.099365566			-
N_acylglucosamine_6_phosphate_2_epimerase	2.349783513			-
betaine_aldehyde_dehydrogenase	2.546665819			-
putative_dinucleoside_polyphosphate_hydrolase	2.493193731			-
heterodisulfide_reductase_subunit_B	0.906481952			-
heterodisulfide_reductase_subunit_C	0.621476952			-
ATP_synthase_in_type_III_secretion_protein_N	2.315285048			-
ATP_binding_cassette_subfamily_C_bacterial_PrsD	0			-

Table S11. KEGG orthology functional terms identified by PICRUSt as different in neonatal jaundice infants (NJI) and non-NJI at 0 months (continued)

Biomaker_names	Logarithm value	Groups	LDA_value	P_value
GTP_cyclohydrolase_I	2.591092002			-
holo_acyl_carrier_protein_synthase	2.548877206			-
PTS_system_fructose_specific_IIC_component	2.865006554			-
f_5_deoxy_glucuronate_isomerase	2.023503426			-
bacitracin_transport_system_permease_protein	0.614600464			-
arginine_N_succinyltransferase	1.998121754			-
IMP_cyclohydrolase	0			-
long_chain_acyl_CoA_synthetase	3.69156707			-
two_component_system_OmpR_family_sensor_histidine_kinase_SenX3	1.436300618			-
glutamateaspartate_transport_system_substrate_binding_protein	2.779372892			-
D_cysteine_desulfhydrase	2.565630415			-
nickel_transport_protein	0.522320327			0.0249747
alanine_synthesizing_transaminase	3.382833477			-
undecaprenol_kinase	0			-
L_asparaginase	2.961493981			-
acetyl_CoA_synthase	1.403763198			0.0163092
benzoylformate_decarboxylase	0.127147913			-
MSHA_pilin_protein_MshD	0.081256386			-
cytidine_deaminase	2.9256074			-
MSHA_pilin_protein_MshB	0			-
MSHA_pilin_protein_MshC	0.054839378			-
MSHA_pilin_protein_MshA	0.360464766			-
f_2_keto_myo_inositol_isomerase	1.912146465			-
dihydroxy_acid_dehydratase	3.400193531			-
microcin_C_transport_system_ATP_binding_protein	2.038825075			-
iduronate_2_sulfatase	1.115358696			-
diaminopimelate_epimerase	2.920828356			-
isopentenyl_diphosphate_Delta_isomerase	2.489090192			-
photosynthetic_reaction_center_L_subunit	0			-
ATP_dependent_RNA_helicase_RhlB	2.478203985			-

Table S11. KEGG orthology functional terms identified by PICRUSt as different in neonatal jaundice infants (NJI) and non-NJI at 0 months (continued)

Biomaker_names	Logarithm value	Groups	LDA_value	P_value
polyA_polymerase	2.566942177			-
PTS_system_trehalose_specific_IIB_component	0.771683145			-
orotate_phosphoribosyltransferase	2.659334737			-
isopenicillin_N_epimerase	0			-
pyruvate_ferredoxin_oxidoreductase_delta_subunit	1.272847886			-
f_3_oxoadipate_enol_lactonase	2.168066645			-
f_6_deoxyerythronolide_B_synthase	0			-
two_component_system_NtrC_family_response_regulator_HydG	2.124075487			-
acetyl_CoA_synthetase	3.382243748			-
glycogen_debranching_enzyme	2.409319952			-
chromosomal_replication_initiator_protein	2.957737223			-
aerotaxis_receptor	2.820366701			-
aerobic_C4_dicarboxylate_transport_protein	2.477794357			-
gamma_glutamylputrescine_oxidase	2.442072925			-
glutamate_dehydrogenase	2.26465773			-
isoleucyl_tRNA_synthetase	2.656857656			-
f_1_deoxy_D_xylulose_5_phosphate_reductoisomerase	2.603196445			-
dihydroneopterin_aldolase_7_8_dihydroneopterin_epimerase	2.545427308			-
methylenetetrahydrofolate_dehydrogenase_NADP_methenyltetrahydrofolate_cyclohydrolase	3.4531651			-
FPRL1_inhibitory_protein	0			-
D_methionine_transport_system_substrate_binding_protein	2.741734114			-
phosphoglycerate_kinase	3.258900127			-
f_2S_methylsuccinyl_CoA_dehydrogenase	1.4281032			-
homoisocitrate_dehydrogenase	0			-
nitrite_reductase_NADH_large_subunit	2.302541393			-
dihydrolipoamide_dehydrogenase	3.694774568			-
glycosyl_4_4_diaponeurosporenoate_acyltransferase	0.455954631			-
acylpyruvate_hydrolase	0			-
cytochrome_c_type_protein_NapB	2.319178821			-
mannitol_2_dehydrogenase	1.511147307			-

Table S11. KEGG orthology functional terms identified by PICRUSt as different in neonatal jaundice infants (NJI) and non-NJI at 0 months (continued)

Biomaker_names	Logarithm value	Groups	LDA_value	P_value
cag_pathogenicity_island_protein_22	0			0.0360487
zeaxanthin_glucosyltransferase	0			-
sedoheptulokinase	0			0.0326303
STE24_endopeptidase	0.774659387			-
phospholipase_C	1.49424723			-
N_acetylglucosaminyldiphosphoundecaprenol_N_acetyl_beta_D_mannosaminyltransferase	2.013568315			-
PTS_system_N_acetylglucosamine_specific_IIB_component	0.676486603			0.0249747
subtilisin	0			-
adenylylsulfate_reductase_subunit_A	0.364021708			-
dipeptide_transport_system_substrate_binding_protein	2.790391832			-
adenosinetriphosphatase	0			-
replicative_DNA_helicase	3.162815375			-
UDP_MurNAc_hydroxylase	0			-
undecaprenyl_phosphate_alpha_L_ara4N_flippase_subunit_ArnF	2.47218813			-
manganesezinciron_transport_system_permease_protein	1.482553393			-
undecaprenyl_phosphate_alpha_L_ara4N_flippase_subunit_ArnE	2.472850197			-
phosphoribosyl_1_2_cyclic_phosphate_phosphodiesterase	2.12117675			-
succinylornithine_aminotransferase	1.966019878			-
mevalonate_kinase	1.923142337			-
glutamate_transport_system_permease_protein	1.732634738			-
PTS_system_sorbose_specific_IIA_component	2.0789187			-
PTS_system_mannitol_specific_IIB_component	0.013715317			-
fructan_beta_fructosidase	0			0.0360487
formamidase	1.558905053			-
gingipain_R	0.019953336			-
isopenicillin_N_synthase	0			-
f_3_4_dihydroxy_2_butanone_4_phosphate_synthase	2.499853194			-
pullulanase	2.264170258			-
nitroreductase_dihydropteridine_reductase	2.590806817			-
f_5_nucleotidase_UDP_sugar_diphosphatase	3.082194363			-

Table S11. KEGG orthology functional terms identified by PICRUSt as different in neonatal jaundice infants (NJI) and non-NJI at 0 months (continued)

Biomaker_names	Logarithm value	Groups	LDA_value	P_value
citronellyl_CoA_synthetase	0.624351967			-
anaerobic_dimethyl_sulfoxide_reductase_subunit_A	2.490807535			-
f_4_hydroxyproline_epimerase	0.693728758			-
anthranilate_1_2_dioxygenase_deaminating_decarboxylating_small_subunit	0			-
PTS_system_trehalose_specific_IIC_component	2.781908968			-
adenylosuccinate_synthase	2.958350296			-
DSF_synthase	1.796343387			-
two_component_system_OmpR_family_sensor_histidine_kinase_KdpD	2.554066058			-
flavin_reductase_ActVB	0			-
N_acetylglucosamine_transport_system_substrate_binding_protein	1.838538393			-
sucrose_synthase	0			-
GMP_reductase	2.010792004			-
acetaldehyde_dehydrogenase_alcohol_dehydrogenase	3.729863722			-
AI_2_transport_system_permease_protein	2.268138106			-
limonene_1_2_epoxide_hydrolase	0.268953738			-
arginine_deiminase	1.597983715			-
mannosyl_3_phosphoglycerate_phosphatase	1.558803826			-
nicotinate_phosphoribosyltransferase	2.592003444			-
methylglutaconyl_CoA_hydratase	1.538816341			-
NAD_diphosphatase	2.898282081			-
C4_dicarboxylate_transporter_DctQ_subunit	1.150163871			-
nitrate_reductase_gamma_subunit	2.343044624			-
two_component_system_response_regulator_stage_0_sporulation_protein_A	2.177250525			-
two_component_system_response_regulator_stage_0_sporulation_protein_F	0.577662385			-
two_component_system_OmpR_family_alkaline_phosphatase_synthesis_response_regulator_PhoP	2.036861353			0.037373
N_N_dimethylformamidase	0.860939034			-
alpha_glucoside_transport_system_substrate_binding_protein	1.364486308			-
levansucrase	0.809304725			-
UDP_N_acetylmuramoyl_tripeptide__D_alanyl_D_alanine_ligase	3.135083776			-
f_2_oxoisovalerate_dehydrogenase_E1_component_beta_subunit	1.778011039			-

Table S11. KEGG orthology functional terms identified by PICRUSt as different in neonatal jaundice infants (NJI) and non-NJI at 0 months (continued)

Biomaker_names	Logarithm value	Groups	LDA_value	P_value
minimal_PKS_chain_length_factor_CLFKS_beta	0			-
MFS_transporter_OPA_family_phosphoglycerate_transporter_protein	0.194438019			-
pyrroline_5_carboxylate_reductase	2.975155781			-
phosphoribulokinase	2.788293235			-
N_acylglucosamine_2_epimerase	1.76713398			0.037373
sulfite_reductase_NADPH_hemoprotein_beta_component	2.497006248			-
photosystem_II_CP47_chlorophyll_apoprotein	0			-
acetoacetyl_CoA_synthetase	1.435800942			-
Tat_targeted_selenate_reductase_subunit_YnfF	0.781514738			-
Tat_targeted_selenate_reductase_subunit_YnfE	1.957703457			-
f_1_4_alpha_glucan_branching_enzyme	2.613245124			-
staphylokinase	0			-
flagellar_basal_body_rod_modification_protein_FlgD	2.483916109			-
lysineargininеornithine_transport_system_substrate_binding_protein	2.266447078			-
ribonucleoside_diphosphate_reductase_alpha_chain	3.145930463			-
f_2_dehydro_3_deoxyphosphogluconate_aldolase_4S_4_hydroxy_2_oxoglutarate_aldolase	3.405151725			-
ethanolamine_ammonia_lyase_large_subunit	2.0979063			-
acetyl_CoApropionyl_CoA_carboxylase	0.11537322			-
f_4_hydroxy_3_polyprenylbenzoate_decarboxylase	2.60155309			-
chemotaxis_protein_CheZ	2.393929616			-
serinealanine_adding_enzyme	1.452130129			-
zona_occludens_toxin	0			-
UDP_2_3_diacylglucosamine_hydrolase	2.521794905			-
glycerol_dehydrogenase	2.933505721			-
ferredoxin_nitrate_reductase	0			-
L_ascorbate_oxidase	0			-
uroporphyrinogen_decarboxylase	2.533737647			-
D_methionine_transport_system_ATP_binding_protein	2.613174759			-
histidyl_tRNA_synthetase	2.658606011			-
f_3_deoxy_D_manno_octulosonic_acid_transferase	2.536020868			-

Table S11. KEGG orthology functional terms identified by PICRUSt as different in neonatal jaundice infants (NJI) and non-NJI at 0 months (continued)

Biomaker_names	Logarithm value	Groups	LDA_value	P_value
glutamine_transport_system_permease_protein	2.198823808			-
pantoate__beta_alanine_ligase	2.841104242			-
haloalkane_dehalogenase	1.180197577			-
formate_dehydrogenase_major_subunit	3.535428736			-
two_component_system_NarL_family_sensor_histidine_kinase_ComP	0			-
kynureninase	1.576080757			-
two_component_system_OmpR_family_phosphate_regulon_sensor_histidine_kinase_PhoR	2.607264822			-
xanthosine_phosphorylase	1.957126307			-
f_3_oxo_5_6_didehydrosuberyl_CoA3_oxoadipyl_CoA_thiolase	2.013402344			-
arginineornithine_transport_system_substrate_binding_protein	0			-
aminoacrylate_peracid_reductase	1.965053984			-
FADH2_O2_dependent_halogenase	0			-
cytochrome_d_ubiquinol_oxidase_subunit_I	3.167564993			-
linoleoyl_CoA_desaturase	1.488277148			-
thiamine_thiazole_synthase	1.71462241			-
f_3_hexulose_6_phosphate_synthase_6_phospho_3_hexuloisomerase	0			-
thimet_oligopeptidase	0.514190172			-
farnesyl_diphosphate_farnesyltransferase	0			-
phosphoribosylamine__glycine_ligase	2.654659559			-
f_3_oxoacyl_acyl_carrier_protein_synthase_I	3.110303183	NJI	2.33785085	0.037373
phosphoenolpyruvate_phosphomutase	0.158737154			-
putative_tricarboxylic_transport_membrane_protein	2.76567512			-
CDP_diacylglycerol__glycerol_3_phosphate_3_phosphatidyltransferase	2.705006425			-
alcohol_dehydrogenase	3.230626751			-
flagellar_biosynthetic_protein_FliRFlhB	0.465568253			-
f_2_C_methyl_D_erythritol_4_phosphate_cytidylyltransferase_2_C_methyl_D_erythritol_2_4_cyclodiphosphate_synthase	1.762625806			-
protocatechuate_4_5_dioxygenase_beta_chain	1.641007336			-
D_alanine_D_alanine_ligase	3.232482206			-
photosystem_II_PsbH_protein	0			-
SecDSecF_fusion_protein	2.396323086			-

Table S11. KEGG orthology functional terms identified by PICRUSt as different in neonatal jaundice infants (NJI) and non-NJI at 0 months (continued)

Biomaker_names	Logarithm value	Groups	LDA_value	P_value
chemotaxis_protein_CheX	0.927518219			-
xanthine_dehydrogenase_large_subunit	1.594354831			-
D_ribose_pyranase	2.512056512			-
UDP_N_acetylglucosamineUDP_N_acetylgalactosamine_diphosphorylase	0.109161911			0.0360487
urea_transport_system_permease_protein	0			-
alpha_alpha_trehalose_phosphorylase	0			-
PTS_system_beta_glucoside_specific_IIC_component	2.36823985			-
atrazine_chlorohydrolase	0.896202115			-
ribose_1_5_bisphosphokinase	2.008170136			-
arginine_transport_system_ATP_binding_protein	2.473183626			-
branched_chain_amino_acid_transport_system_ATP_binding_protein	3.42679493			-
sec_independent_protein_translocase_protein_TatB	2.79819778			-
pyrimidine_operon_attenuation_protein_uracil_phosphoribosyltransferase	1.838585459			0.0249747
chemotaxis_protein_CheC	1.84289449			0.0249747
sulfate_transport_system_permease_protein	3.086453026			-
sec_independent_protein_translocase_protein_TatC	2.854437119			-
kinase_associated_protein_B	0.350730484			-
ribokinase	2.695011741			-
acylphosphatase	2.844557846			-
modification_methylase	1.407495501			-
alanine_dehydrogenase	2.540221357			-
phosphate_propanoyltransferase	1.784915594			-
nitrile_hydratase_subunit_alpha	2.492183702			-
galactokinase	2.892445905			-
sec_independent_protein_translocase_protein_TatA	2.838299923			-
chemotaxis_protein_CheD	1.929166125			-
C4_dicarboxylate_binding_protein_DctP	1.893747957			-
PAH_dioxygenase_large_subunit	0			-
quinolinate_synthase	2.571800598			-
flagellar_M_ring_protein_FliF	2.477886809			-

Table S11. KEGG orthology functional terms identified by PICRUSt as different in neonatal jaundice infants (NJI) and non-NJI at 0 months (continued)

Biomaker_names	Logarithm value	Groups	LDA_value	P_value
diaminobutyrate_2_oxoglutarate_transaminase	2.452108075			-
cystathione_beta_lyase	2.906905276			-
carboxynorspermidine_decarboxylase	2.156736371			-
penicillin_binding_protein_3	0.64501512			-
two_component_system_OmpR_family_manganese_sensing_sensor_histidine_kinase	0			-
type_IV_secretion_system_protein_TrbL	1.763761831			-
two_component_system_OmpR_family_response_regulator_CssR	0.348833578			0.0463198
GMP_synthase_glutamine_hydrolysing	3.084940492			-
S_adenosylmethionine_synthetase	3.032024662			-
cystine_transport_system_ATP_binding_protein	1.96801098			-
mycobactin_lysine_N_oxygenase	0			-
hydrogen_cyanide_synthase_HcnC	0			-
glucosamine__fructose_6_phosphate_aminotransferase_isomerizing	3.110036276			-
f_4_hydroxyacetophenone_monooxygenase	0			-
f_1_phosphatidylinositol_phosphodiesterase	0			-
amidase	2.849256221			-
molybdopterin_adenylyltransferase	2.775991314			-
response_regulator_aspartate_phosphatase_B	0			-
formate_C_acetyltransferase	3.386955376			-
holin_like_protein	0.992829434			-
anaerobic_nitric_oxide_reductase_transcription_regulator	1.961020431			-
oxygen_independent_coproporphyrinogen_III_oxidase	2.899200034			-
arylsulfatase	2.479653941			-
bis5_nucleosyl_tetraphosphatase_symmetrical	2.479916207			-
methyl_galactoside_transport_system_substrate_binding_protein	2.512159593			-
two_component_system_NtrC_family_response_regulator_GlrR	2.774568279			-
ubiquinol_cytochrome_c_reductase_cytochrome_c1_subunit	2.337136063			-
protocatechuate_3_4_dioxygenase_alpha_subunit	2.315727814			-
glutathione_reductase_NADPH	2.806184284			-
phosphoglycerate_transport_regulatory_protein_PgtC	1.103826805			-

Table S11. KEGG orthology functional terms identified by PICRUSt as different in neonatal jaundice infants (NJI) and non-NJI at 0 months (continued)

Biomaker_names	Logarithm value	Groups	LDA_value	P_value
tagatose_1_6_diphosphate_aldolase	2.016445885			0.037373
pyrimidine_oxygenase	1.967561915			-
uroporphyrinogen_III_synthase	2.535489882			-
membrane_fusion_protein_multidrug_efflux_system	3.171082702			-
phosphopantothenate_cysteine_ligase	1.47716057			-
PTS_system_beta_glucoside_specific_IIA_component	0			-
nitrogen_regulatory_protein_P_II_1	2.552296657			-
UDP_galactopyranose_mutase	2.531499755			-
galactarate_dehydratase	1.969098492			-
bicarbonate_transport_system_ATP_binding_protein	0			-
N_formylglutamate_deformylase	0.802127767			-
ADP_ribose_pyrophosphatase	2.600866389			-
phytoene_synthase	1.517883965			-
serinethreonine_protein_kinase_PpkA	0			-
trehalose_6_phosphate_phosphatase	1.998818474			-
acetaldehyde_dehydrogenase_acetylating	0			-
acetylornithineN_succinyldiaminopimelate_aminotransferase	3.267852647			-
nitric_oxide_reductase_FlRd_NAD_reductase	1.960546377			-
f_3_hexulose_6_phosphate_synthase	1.434993468			-
oligoribonuclease	2.52094363			-
NADPH_quinone_oxidoreductase_subunit_K	0			-
f_2_haloacid_dehalogenase	2.106369963			-
muconate_cycloisomerase	1.835322322			-
L_rhamnose_isomerase_sugar_isomerase	0.16308925			-
f_3_dehydroquinate_synthase_II	0			-
benzoatetoluate_1_2_dioxygenase_subunit_beta	1.227983393			-
cyclic_pyranopterin_monophosphate_synthase	2.826719073			-
oligopeptidase_B	2.829221269			-
hydroxylamine_dehydrogenase	0			-
bacterialarchaeal_transporter_family_2_protein	2.741174609			-

Table S11. KEGG orthology functional terms identified by PICRUSt as different in neonatal jaundice infants (NJI) and non-NJI at 0 months (continued)

Biomaker_names	Logarithm value	Groups	LDA_value	P_value
f_2_oxoglutarate_ferredoxin_oxidoreductase_subunit_delta	2.35044529			-
cystathionine_beta_lyase	3.007461378			-
UDP_D_galactose_glucosylLPS_alpha_1_6_D_galactosyltransferase	0.267264383			-
two_component_system_OmpR_family_response_regulator_SaeR	0.774697837			0.0277684
f_5_methyltetrahydrofolate__homocysteine_methyltransferase	3.293629755			-
f_4_amino_4_deoxy_L_arabinose_transferase	2.490663905			-
L_fuconolactonase	1.814162592			-
light_harvesting_protein_B_800_850_beta_chain	0			-
L_xylulokinase	2.542096598			-
urease_subunit_gamma	2.497370629			-
ribonuclease_HIII	1.521132822			-
precorrin_3B_synthase	1.343711559			-
thioredoxin_reductase_NADPH	3.097410097			-
arginine_kinase	0			-
assimilatory_nitrate_reductase_electron_transfer_subunit	0			-
NADH_dehydrogenase_ubiquinone_flavoprotein_2	0			-
PTS_system_N_acetylglucosamine_specific_IIC_component	2.817923831			-
succinyl_diaminopimelate_desuccinylase	2.948878025			-
tagatose_1_6_diphosphate_aldolase_GatYKbaY	2.476822992			-
arylsulfatase_A	2.017678629			-
trans_o_hydroxybenzylidenepyruvate_hydratase_aldolase	1.403315458			-
carbonyl_reductase_1	0.178896812			-
cell_division_protein_FtsW	2.670722739			-
molybdopterin_molybdotransferase	2.58266457			-
cystathionine_beta_synthase	2.552400481			-
molybdate_transport_system_substrate_binding_protein	2.614325277			-
putative_selenate_reductase_molybdopterin_binding_subunit	0.6420864			-
geranyl_CoA_carboxylase_alpha_subunit	0.369001554			-
menaquinone_specific_isochorismate_synthase	2.776469775			-
TorA_specific_chaperone	0.702078985			-

Table S11. KEGG orthology functional terms identified by PICRUSt as different in neonatal jaundice infants (NJI) and non-NJI at 0 months (continued)

Biomaker_names	Logarithm value	Groups	LDA_value	P_value
pyrophosphatase_PpaX	0.589077334			0.037373
f_4_hydroxy_tetrahydrodipicolinate_synthase	3.457273722			-
f_5_carboxyaminoimidazole_ribonucleotide_mutase	2.656251788			-
bilirubin_oxidase	0.272649833			-
two_component_system_OmpR_family_sensor_histidine_kinase_YxdK	0			-
PTS_system_galactitol_specific_IIB_component	2.803840626			-
D_alanyl_D_alanine_dipeptidase	1.968932608			-
undecaprenyl_diphosphate_synthase	2.659424255			-
f_4_oxalocrotonate_tautomerase	2.856302056			-
serine_aspartate_repeat_containing_protein_CDE	0			-
lycopene_beta_cyclase	0.446198769			-
f_1_hydroxycarotenoid_3_4_desaturase	0			-
f_4_hydroxybutyryl_CoA_dehydratase_vinylacetyl_CoA_Delta_isomerase	2.613603085	non-NJI	2.18076308	0.0163092
glutathionylspermidine_amidasesynthetase	2.588370225			-
NADH_quinone_oxidoreductase_subunit_G	2.577307293			-
ribonuclease_PMRP_protein_subunit_POP5	0.509662564			-
iron_complex_transport_system_permease_protein	3.268666861			-
putative_colanic_acid_biosysnthesis_UDP_glucose_lipid_carrier_transferase	2.131377852			-
long_chain_fatty_acid__acyl_carrier_protein_ligase	0			-
f_2_C_methyl_D_erythritol_4_phosphate_cytidylyltransferase	2.658338204			-
f_1_3_propanediol_dehydrogenase	0.903101489			-
f_5_methylthioribose_kinase	1.979679474			-
adhesininvasin	2.320236431			-
cardiolipin_synthase_AB	2.71141634			-
putative_sodiumglutamine_symporter	0			-
cystine_transport_system_substrate_binding_protein	1.965322813			-
phosphonate_transport_system_substrate_binding_protein	2.095470906			-
KDO_transferase_III	0			-
tryptophan_2_3_dioxygenase	1.454275059			-
f_4_2_carboxyphenyl_2_oxobut_3_enoate_aldolase	0.20007755			-

Table S11. KEGG orthology functional terms identified by PICRUSt as different in neonatal jaundice infants (NJI) and non-NJI at 0 months (continued)

Biomaker_names	Logarithm value	Groups	LDA_value	P_value
hemin_transport_system_permease_protein	0.343985124			-
AMP_nucleosidase	2.528683105			-
phosphoglucomutase	3.367137439			-
f_6_pyruvoyltetrahydropterin6_carboxytetrahydropterin_synthase	2.528628469			-
purine_nucleoside_phosphorylase	3.208685517			-
f_4_aminobutyrate_aminotransferase_S_3_amino_2_methylpropionate_transaminase	2.688537589			-
f_2_amino_4_hydroxy_6_hydroxymethyldihydropteridine_diphosphokinase	2.627085866			-
fatty_acid_synthase_bacteria_type	1.897106937			-
triosedihydroxyacetone_kinase_FAD_AMP_lyase_cyclizing	2.275569121			-
precorrin_8Xcobalt_precorrin_8_methylmutase	2.123171448			-
acetolactate_synthase_IIII_small_subunit	3.466171188			-
two_component_system_OmpR_family_response_regulator_BaeR	2.478046977			-
lipoprotein_releasing_system_ATP_binding_protein	2.540810247			-
f_3_hydroxybutyryl_CoA_dehydrogenase	2.86968868			-
photosystem_I_P700_chlorophyll_a_apoprotein_A2	0			-
manganesezinciron_transport_system_substrate_binding_protein	1.182453964			-
succinate_dehydrogenase_fumarate_reductase_iron_sulfur_subunit	3.268486856			-
trehalosemaltose_transport_system_substrate_binding_protein	0.404030183			-
methyl_galactoside_transport_system_ATP_binding_protein	1.980215933			-
platelet_activating_factor_acetylhydrolase	1.177792012			-
PTS_system_glucose_specific_IIC_component	3.088056933			-
f_3_phytase	0.211095698			-
photosystem_I_P700_chlorophyll_a_apoprotein_A1	0			-
toxin_AB	0.504226658			-
glutamate_transport_system_ATP_binding_protein	1.507474511			-
NADPH_quinone_oxidoreductase_subunit_H	0			-
manganesezinciron_transport_system_ATP__binding_protein	1.188846866			-
sulfoacetaldehyde_acetyltransferase	0.869445244			-
glyceraldehyde_3_phosphate_dehydrogenase_NADP	1.788919347			0.0249747
oxaloacetate_decarboxylase_beta_subunit	2.377715781			-

Table S11. KEGG orthology functional terms identified by PICRUSt as different in neonatal jaundice infants (NJI) and non-NJI at 0 months (continued)

Biomaker_names	Logarithm value	Groups	LDA_value	P_value
two_component_system_OmpR_family_sensor_histidine_kinase_SaeS	0.775817156			0.0277684
flagellar_FliJ_protein	2.419022481			-
f_2_oxoisovalerate_dehydrogenase_E1_component_alpha_subunit	1.776979388			-
lactate_2_monooxygenase	0			-
S_adenosylmethionine_decarboxylase	2.345219659			-
acetoacetyl_CoA_reductase	2.154043438			-
dCTP_deaminase	2.531263806			-
f_5_oxoprolinase_ATP_hydrolysing	1.497462104			-
S_adenosylmethionine_diacylgycerolhomoserine_N_methlytransferase	1.338387552			-
f_3_methyl_2_oxobutanoate_hydroxymethyltransferase	2.60151995			-
oxygen_dependent_protoporphyrinogen_oxidase	1.241326801			-
methylthioribulose_1_phosphate_dehydratase	1.957363784			-
f_3_hydroxyacyl_CoA_dehydrogenase_enoyl_CoA_hydratase_3_hydroxybutyryl_CoA_epimerase_enoyl_CoA_isomerase	4.150621995			-
methylglyoxal_synthase	2.627030432			-
photosystem_II_PsbM_protein	0			-
riboflavin_kinase_FMN_adenylyltransferase	2.956771056			-
two_component_system_NtrC_family_response_regulator_PilR	1.146630907			-
NAD_kinase	2.65821392			-
flagellar_motor_switch_protein_FliNFliY	2.785789676			-
DNA_adenine_methylase	2.54098957			-
phosphoribosylformylglycinamidine_cyclo_ligase	2.652090517			-
NAD_synthase_glutamine_hydrolysing	2.543552167			-
f_3_5_cyclic_AMP_phosphodiesterase	2.779193072			-
alpha_amylase	2.663522577			-
deoxyguanosine_kinase	1.522757058			-
dissimilatory_sulfite_reductase_beta_subunit	0.342352678			-
cystathionine_gamma_lyase	2.578318129			-
N_sulfoglucosamine_sulfohydrolase	0			-
two_component_system_NarL_family_response_regulator_YdfI	0			-
f_3S_malyl_CoA_thioesterase	0.105527123			-

Table S11. KEGG orthology functional terms identified by PICRUSt as different in neonatal jaundice infants (NJI) and non-NJI at 0 months (continued)

Biomaker_names	Logarithm value	Groups	LDA_value	P_value
phycocyanin_associated_rod_linker_protein	0			-
polybeta_D_mannuronate_lyase	0			-
f_2_3_dihydroxyphenylpropionate_1_2_dioxygenase	2.081614986			-
sulfur_carrier_protein_ThiS_adenylyltransferase	2.777392463			-
arginineornithine_transport_system_ATP_binding_protein	0			-
adenosine_kinase	0.766188503			-
two_component_system_OmpR_family_response_regulator_RpaB	0.233484297			-
two_component_system_OmpR_family_response_regulator_RpaA	0			-
glucose_1_dehydrogenase	1.477238332			0.0249747
ATP_binding_cassette_subfamily_C_bacterial_LapB	0			-
methylisocitrate_lyase	2.32265887			-
f_2_3_cyclic_nucleotide_2_phosphodiesterase_3_nucleotidase_5_nucleotidase	1.658610673			-
large_subunit_ribosomal_protein_L7L12	2.656614323			-
ornithine_decarboxylase	2.843309184			-
putative_multiple_sugar_transport_system_permease_protein	1.465880633			-
flagellar_biosynthetic_protein_FliP	2.478442743			-
exodeoxyribonuclease_V_alpha_subunit	2.542648044			-
f_4_4_diapophytoene_desaturase	0.508559795			-
carnitine_O_acetyltransferase	0.043107794			-
uracil_phosphoribosyltransferase	2.655682506			-
UDP_GlcNAc3NAcA_epimerase	0			-
lia_operon_protein_LiaF	0			-
glucose_6_phosphate_1_dehydrogenase	3.181158886			-
f_4_coumarate__CoA_ligase	0			-
hippurate_hydrolase	2.374794305			-
carbamoyl_phosphate_synthase_large_subunit	3.045087938			-
type_I_protein_arginine_methyltransferase	0			-
peroxiredoxin_alkyl_hydroperoxide_reductase_subunit_C	2.654115552			-
large_subunit_ribosomal_protein_L36	2.616452677			-
L_rhamnonate_dehydratase	1.778321065			-

Table S11. KEGG orthology functional terms identified by PICRUSt as different in neonatal jaundice infants (NJI) and non-NJI at 0 months (continued)

Biomaker_names	Logarithm value	Groups	LDA_value	P_value
endonuclease_III	2.656571419			-
adenylate_cyclase	2.623257493			-
trimethylamine___corrinoid_protein_Co_methyltransferase	1.995281265			-
formiminoglutamase	2.090124412			-
O_succinylbenzoate_synthase	2.476699739			-
cyclase	2.920162966			-
two_component_system_AgrA_family_sensor_histidine_kinase_ComD	1.362839283			-
DNA_helicase_II_ATP_dependent_DNA_helicase_PcrA	3.183004152			-
cytochrome_o_ubiquinol_oxidase_subunit_IV	2.482214872			-
nonribosomal_peptide_synthetase_DhbF	0			-
D_lactate_dehydrogenase_cytochrome	1.457540037			-
nicotinate_nucleotide_adenylyltransferase	2.650620775			-
two_component_system_OmpR_family_sensor_histidine_kinase_BaeS	2.478025616			-
beta_ureidopropionase_N_carbamoyl_L_amino_acid_hydrolase	1.877611845			-
f_2_3_bisphosphoglycerate_dependent_phosphoglycerate_mutase	3.943879707			-
carbamate_kinase	3.161005555			-
glutamateaspartate_transport_system_permease_protein	3.078433254			-
chitinase	2.231850002			-
cytochrome_o_ubiquinol_oxidase_subunit_II	2.482214872			-
malate_dehydrogenase_oxaloacetate_decarboxylating	3.092893897			-
phospholipase_D12	1.753960824			-
f_2_3_cyclic_nucleotide_2_phosphodiesterase_3_nucleotidase	2.86100779			-
f_3_deoxy_7_phosphoheptulonate_synthase	3.468567972			-
succinate_dehydrogenase_fumarate_reductase_membrane_anchor_subunit	3.193340411			-
f_4_phytase_acid_phosphatase	2.438974828			-
ureidoglycolate_dehydrogenase_NAD	2.316529224			-
zinc_transport_system_permease_protein	2.604813131			-
glutamine_synthetase	3.786205258			-
PTS_system_D_glucosamine_specific_IIC_component	2.241276988			-
invasin_B	0			-

Table S11. KEGG orthology functional terms identified by PICRUSt as different in neonatal jaundice infants (NJI) and non-NJI at 0 months (continued)

Biomaker_names	Logarithm value	Groups	LDA_value	P_value
two_component_system_LytT_family_response_regulator_LytT	1.020506784			-
putrescine_aminotransferase	2.019758389			-
f_3_carboxy_cis_cis_muconate_cycloisomerase	2.142416932			-
f_5_carboxymethyl_2_hydroxymuconic_semialdehyde_dehydrogenase	2.344473313			-
proteasome_associated_ATPase	1.435877614			-
glycine_amidinotransferase	0.555651916			-
thiosulfate3_mercaptopyruvate_sulfurtransferase	3.412639255			-
flagellum_specific_ATP_synthase	2.460321361			-
serine_protease	1.038054107			-
UDP_glucosegalactose_glucosylLPS_alpha_1_2_glucosylgalactosyltransferase	0.608825249			-
f_2_aminoethylphosphonate_pyruvate_transaminase	2.169989988			-
gallate_dioxygenase	0			-
two_component_system_NarL_family_response_regulator_LiaR	1.421086339			0.037373
anthranilate_phosphoribosyltransferase	2.439458189			-
methylmalonyl_CoA_decarboxylase	1.780634201			-
vanillate_monooxygenase	1.991927948			-
lipooligosaccharide_transport_system_permease_protein	0.889433987			-
mannosyl_glycoprotein_endo_beta_N_acetylglucosaminidase	1.564174687			-
two_component_system_NarL_family_response_regulator_fimbrial_Z_protein_FimZ	1.495485187			-
f_5_dehydro_2_deoxygluconokinase	2.023503426			-
LuxR_family_transcriptional_regulator_capsular_biosynthesis_positive_transcription_factor	2.266251575			-
alpha_ketoglutarate_dependent_2_4_dichlorophenoxyacetate_dioxygenase	0			-
adenine_phosphoribosyltransferase	2.594738837			-
toxin_co_regulated_pilin	0			-
chemosensory_pili_system_protein_ChpA_sensor_histidine_kinaseresponse_regulator	1.440392052			-
glucose_1_phosphatase	2.473172846			-
NADPH_dehydrogenase_quinone	2.372417992			-
f_3_4_dihydroxyphenylacetate_2_3_dioxygenase	2.33591592			-
shikimate_kinase	3.119015515			-
glutamate__cysteine_ligase	2.842948216			-

Table S11. KEGG orthology functional terms identified by PICRUSt as different in neonatal jaundice infants (NJI) and non-NJI at 0 months (continued)

Biomaker_names	Logarithm value	Groups	LDA_value	P_value
GTP_3_8_cyclase	2.843077539			-
thiol_disulfide_interchange_protein_DsbA	2.558820242			-
D_amino_acid_dehydrogenase	2.555794336			-
alpha_N_acetylglucosaminidase	1.616412177			-
f_3_methylcrotonyl_CoA_carboxylase_alpha_subunit	1.671279972			-
gluconate_2_dehydrogenase_gamma_chain	0.844108268			-
guanosine_3_5_bisdiphosphate_3_pyrophosphohydrolase	2.486994428			-
phosphoglycolate_phosphatase	2.85026849			-
f_4_hydroxyphenylpyruvate_dioxygenase	2.382720379			-
CRPFNR_family_transcriptional_regulator_cyclic_AMP_receptor_protein	3.079060117			-
PTS_system_N_acetylgalactosamine_specific_IID_component	1.98328924			-
phosphoribosylaminoimidazolecarboxamide_formyltransferase_IMP_cyclohydrolase	3.432145387			-
xanthine_dehydrogenase_iron_sulfur_binding_subunit	0.634025868			-
f_3_deoxy_manno_octulosonate_cytidylyltransferase_CMP_KDO_synthetase	2.565088983			-
beta_lysine_5_6_aminomutase_alpha_subunit	0.528375389			-
f_3_deoxy_D_manno_octulosonic_acid_kinase	0.936409897			-
myo_inositol_2_dehydrogenase_D_chiro_inositol_1_dehydrogenase	2.668617202			-
N_carbamoylputrescine_amidase	1.917551922			-
phosphonoacetaldehyde_hydrolase	1.989053642			-
twitching_motility_protein_PilJ	1.445234922			-
cell_division_transport_system_ATP_binding_protein	2.638436317			-
sorbitolmannitol_transport_system_substrate_binding_protein	1.525808031			-
f_3_deoxy_7_phosphoheptulonate_synthase_chorismate_mutase	0.997274854			-
PTS_system_galactitol_specific_IIC_component	2.924322967			-
chitosanase	0			-
aminocarboxymuconate_semialdehyde_decarboxylase	0.508884809			-
cellobiose_transport_system_substrate_binding_protein	1.37882088			-
unspecific_monooxygenase	1.479635925			-
signal_peptidase_endoplasmic_reticulum_type	0.939250426			-
AI_2_transport_system_substrate_binding_protein	1.972298926			-

Table S11. KEGG orthology functional terms identified by PICRUSt as different in neonatal jaundice infants (NJI) and non-NJI at 0 months (continued)

Biomaker_names	Logarithm value	Groups	LDA_value	P_value
isohexenylglutaconyl_CoA_hydratase	0			-
MFS_transporter_OPA_family_hexose_phosphate_transport_protein_UhpT	2.017235812			-
f_5_phosphate_synthase_pdxT_subunit	1.515972294			-
tocopherol_O_methyltransferase	0			-
lactoylglutathione_lyase	2.756558661			-
putative_spermidineputrescine_transport_system_substrate_binding_protein	2.606898542			-
hemolysin_D	0			-
prostaglandin_H2_D_isomerase_glutathione_transferase	2.424131737			-
O_acetylhomoserine_thiol_lyase	2.719777181			-
catechol_1_2_dioxygenase	2.2699164			-
urocanate_hydratase	2.536803635			-
two_component_system_NtrC_family_nitrogen_regulation_response_regulator_GlnG	2.495562914			-
alpha_glucosidase	2.886691225			-
two_component_system_NtrC_family_sensor_histidine_kinase_AtoS	0			-
D_erythrose_4_phosphate_dehydrogenase	2.477883314			-
phosphate_transport_system_permease_protein	2.967736948			-
f_4_hydroxythreonine_4_phosphate_dehydrogenase	2.674358527			-
dephospho_CoA_kinase	2.656704047			-
diaminopimelate_dehydrogenase	2.161007039			-
f_6_hydroxycyclohex_1_ene_1_carbonyl_CoA_dehydrogenase	0			-
two_component_system_OmpR_family_sensor_histidine_kinase_BasS	2.266321009			-
L_fuculose_phosphate_aldolase	2.139432403			-
RHH_type_transcriptional_regulator_proline_utilization_regulon_repressor_proline_dehydrogenase_delta_1_pyrroline_5_carboxylate_	3.077462526			-
two_component_system_chemotaxis_family_response_regulator_WspR	0.228559887			-
xylan_1_4_beta_xylosidase	2.196531309			-
prolyl_4_hydroxylase	0.750244039			-
biotin_acetyl_CoA_carboxylase_ligase_type_III_pantothenate_kinase	0.283290428			-
methionyl_tRNA_synthetase	2.961206795			-
p_cumate_2_3_dioxygenase_subunit_alpha	0.154514523			-
glycerol_dehydratase_large_subunit	0.288417962			-

Table S11. KEGG orthology functional terms identified by PICRUSt as different in neonatal jaundice infants (NJI) and non-NJI at 0 months (continued)

Biomaker_names	Logarithm value	Groups	LDA_value	P_value
photosystem_II_oxygen_evolving_enhancer_protein_1	0			-
fructose_bisphosphate_aldolase_class_I	3.477985704			-
two_component_system_chemotaxis_family_response_regulator_WspF	0.243933721			-
benzylsuccinate_CoA_transferase_BbsE_subunit	0			-
cytochrome_b6_f_complex_iron_sulfur_subunit	0			-
malyl_CoAS_citramalyl_CoA_lyase	1.806739288			-
ribonucleoside_diphosphate_reductase_beta_chain	3.117007919			-
f_3_oxoadipate_CoA_transferase_beta_subunit	1.783600884			-
flagellar_biosynthetic_protein_FliQ	2.478269042			-
capsular_polysaccharide_transport_system_permease_protein	1.515737778			-
f_4_guanidinobutyraldehyde_dehydrogenase_NAD_dependent_aldehyde_dehydrogenase	0			-
holliday_junction_DNA_helicase_RuvB	2.657250393			-
holliday_junction_DNA_helicase_RuvA	2.652324728			-
dextranase	0			-
octopinenopaline_transport_system_substrate_binding_protein	0.343713757			-
hydroxylamine_reductase	1.110471999			-
allophycocyanin_alpha_subunit	0			-
f_2_methylfumaryl_CoA_isomerase	0.324428139			-
N_methylhydantoinase_B	1.75303528			-
superoxide_dismutase_Cu_Zn_family	3.181839073			-
N_methylhydantoinase_A	1.776966651			-
f_6_aminohexanoate_cyclic_dimer_hydrolase	0			-
two_component_system_OmpR_family_heavy_metal_sensor_histidine_kinase_CusS	2.518988431			-
ADP_ribose_diphosphatase	2.477953161			-
yersiniabactin_nonribosomal_peptidepolyketide_synthase	0			-
Nif_specific_regulatory_protein	1.788894449			-
caffeoyl_CoA_O_methyltransferase	0.888490522			-
succinyl_CoA_synthetase_alpha_subunit	3.23197835			-
chemotaxis_protein_MotD	0.982642149			-
phosphinothricin_acetyltransferase	2.430444519			-

Table S11. KEGG orthology functional terms identified by PICRUSt as different in neonatal jaundice infants (NJI) and non-NJI at 0 months (continued)

Biomaker_names	Logarithm value	Groups	LDA_value	P_value
chemotaxis_protein_MotB	2.912511628			-
tryptophan_synthase_beta_chain	3.185845402			-
type_III_secretion_protein_Q	2.315274317			-
f_3_dehydroshikimate_dehydratase	0.002420816			-
LysR_family_transcriptional_regulator_transcriptional_activator_AphB	0			-
type_III_secretion_protein_T	2.317483409			-
homoserine_O_acetyltransferase	1.721573971			-
alanine_glyoxylate_transaminase_R_3_amino_2_methylpropionate_pyruvate_transaminase	0			-
thiazole_synthase	2.544495712			-
methyl_galactoside_transport_system_permease_protein	1.980466449			-
cytochrome_c_oxidase_subunit_I	1.865600027			-
beta_N_acetylhexosaminidase	3.032236908			-
nondiscriminating_aspartyl_tRNA_synthetase	0.525815537			0.0163092
propionyl_CoA_carboxylase_beta_chain	2.562979057			-
sarcosine_oxidase_subunit_gamma	1.672556399			-
naphthoate_synthase	2.518653533			-
two_component_system_LuxR_family_response_regulator_DctR	1.830937808			-
f_1_2_dihydroxynaphthalene_dioxygenase	0.043825794			-
glucokinase	3.555257932			-
D_allose_transport_system_ATP_binding_protein	1.952176901			-
riboflavin_kinase	0			-
cobIIyrinic_acid_a_c_diamide_reductase	0.996638988			-
cobalamin_biosynthetic_protein_CobC	1.404314271			-
ribonuclease_E	2.528810306			-
pertactin	0			-
triacylglycerol_lipase	1.68970597			-
ribonuclease_J	2.172461984			-
f_3_dehydroquinate_synthase	2.93408072			-
polyhydroxyalkanoate_synthase	1.658300752			-
alcohol_dehydrogenase_cytochrome_c	1.244458887			-

Table S11. KEGG orthology functional terms identified by PICRUSt as different in neonatal jaundice infants (NJI) and non-NJI at 0 months (continued)

Biomaker_names	Logarithm value	Groups	LDA_value	P_value
cardiolipin_synthase_C	1.971704647			-
folylpolyglutamate_synthase	1.275578916			0.0098755
alanine_adding_enzyme	1.619420343			-
ribonuclease_R	2.633101282			-
benzaldehyde_dehydrogenase_NAD	0.831343894			-
MFS_transporter_OPA_family_sugar_phosphate_sensor_protein_UhpC	2.618082496			-
f_5_aminovalerate4_aminobutyrate_aminotransferase	0			-
methylaspartate_ammonia_lyase	2.265400084			-
K_transporting_ATPase_ATPase_C_chain	2.52440559			-
ribonuclease_Z	2.543711524			-
cytochrome_c_oxidase_cbb3_type_subunit_I	1.95975379			-
flagellar_basal_body_rod_protein_FlgB	2.478604093			-
acetyl_CoA_hydrolase	1.304955976			-
capsular_polysaccharide_transport_system_ATP_binding_protein	1.338362101			-
putative_multiple_sugar_transport_system_ATP_binding_protein	1.465880633			-
lysine_2_3_aminomutase	1.823479371			-
f_5_methyltetrahydrofolate_corrinoidiron_sulfur_protein_methyltransferase	0			-
tocopherol_cyclase	0			-
two_component_system_LytT_family_response_regulator_NatR	0			-
f_3alpha_hydroxysteroid_3_dehydrogenase_chlordecone_reductase	0.095112929			-
D_amino_acid_oxidase	0.111092839			-
f_3_oxoacid_CoA_transferase	0			-
vanillate_monooxygenase_ferredoxin_subunit	1.998935194			-
phosphocarrier_protein_NPr	2.473284771			-
histidine_decarboxylase	0.232854486			-
phosphoribosyl_ATP_pyrophosphohydrolase_phosphoribosyl_AMP_cyclohydrolase	3.121335592			-
glutathione_transport_system_substrate_binding_protein	2.031961966			-
KDO_II_ethanolaminephosphotransferase	2.266355721			-
xanthine_phosphoribosyltransferase	2.628775011			-
sucrose_phosphate_synthase	0			-

Table S11. KEGG orthology functional terms identified by PICRUSt as different in neonatal jaundice infants (NJI) and non-NJI at 0 months (continued)

Biomaker_names	Logarithm value	Groups	LDA_value	P_value
serinethreonine_protein_kinase_PknG	0.585360459			-
imidazoleglycerol_phosphate_dehydratase_histidinol_phosphatase	3.117330688			-
outer_membrane_protein_CuIAgI_efflux_system	1.668653857			-
ATP_phosphoribosyltransferase	2.876687515			-
pectate_disaccharide_lyase	1.77298755			-
molybdate_transport_system_ATP_binding_protein	2.80785498			-
alpha_galactosidase	3.100973434			-
IMP_dehydrogenase	3.028739268			-
yersiniabactin_salicyl_AMP_ligase	1.774197798			-
tRNA_nucleotidyltransferase_CCA_adding_enzyme	2.980335078			-
putative_serine_protease_PepD	1.44047236			-
ethanolamine_phosphate_phospho_lyase	0			-
phenylacetaldehyde_dehydrogenase	2.340186985			-
cytochrome_c_oxidase_subunit_III	1.820314834			-
rhamnosyltransferase	2.039060611			0.037373
inosose_dehydratase	2.225935303			-
f_4_phosphopantetheinyl_transferase	1.971261832			-
N_succinyldiaminopimelate_aminotransferase	1.736829334			-
diphosphomevalonate_decarboxylase	1.621690588			-
two_component_system_CitB_family_response_regulator_DcuR	2.180674472			-
NADP_transhydrogenase	2.478500522			-
holin_like_protein_LrgB	0.992829434			-
malate_Na_symporter	1.773361779			-
isochorismate_pyruvate_lyase	1.816558652			-
flavin_reductase_NADH	2.771747794			-
light_harvesting_complex_1_alpha_chain	0			-
phosphoserine_homoserine_phosphotransferase	1.762871423			0.0163092
acetylornithine_aminotransferase	2.482521355			-
photosynthetic_reaction_center_PufX_protein	0			-
lactoseL_arabinose_transport_system_permease_protein	1.708235941			-

Table S11. KEGG orthology functional terms identified by PICRUSt as different in neonatal jaundice infants (NJI) and non-NJI at 0 months (continued)

Biomaker_names	Logarithm value	Groups	LDA_value	P_value
protoheme_IX_farnesyltransferase	2.801483766			-
penicillin_amidase	0.873658732			-
NADP_transhydrogenase_subunit_beta	2.531440401			-
ribonuclease_HI_DNA_polymerase_III_subunit_epsilon	1.953897713			-
enoyl_acyl_carrier_protein_reductase_II	2.597652082			-
internalin_A	0			-
cytochrome_d_ubiquinol_oxidase_subunit_II	3.033456461			-
precorrin_4cobalt_precorrin_4_C11_methyltransferase	2.110585853			-
tyrosinase	0.85631557			-
hemin_transport_system_ATP_binding_protein	0.343985124			-
hexulose_6_phosphate_isomerase	1.998038083			-
asparagine_synthase_glutamine_hydrolysing	2.594753634			-
dihydroneopterin_triphosphate_diphosphatase	2.474933511			-
f_2_hydroxychromene_2_carboxylate_isomerase	1.404779842			-
pyruvate_ferredoxin_oxidoreductase_alpha_subunit	1.570817455			-
f_5_guanidino_2_oxopentanoate_decarboxylase	0			-
L_lactate_dehydrogenase	3.02059942			-
glycerophosphoryl_diester_phosphodiesterase	2.804706735			-
NiFe_hydrogenase_1_B_type_cytochrome_subunit	1.788946806			0.037373
isorenieratene_synthase	0			-
f_5_aminolevulinate_synthase	1.728715824			-
ketol_acid_reductoisomerase	3.250321148			-
dTDP_glucose_4_6_dehydratase	3.364426045			-
thiosulfate_reductase_polysulfide_reductase_chain_A	0.763553334			0.0098755
f_3_3_hydroxy_phenylpropionate_hydroxylase	2.154663402			-
f_2_hydroxycyclohexanecarboxyl_CoA_dehydrogenase	0.491786771			-
yersiniabactin_synthetase_thiazolinyl_reductase_component	1.774197798			-
tetrahydrodipicolinate_N_acetyltransferase	0.796037674			-
allantoinase	1.4139252			-
citrate_pro_3S_lyase_ligase	2.554755956			-

Table S11. KEGG orthology functional terms identified by PICRUSt as different in neonatal jaundice infants (NJI) and non-NJI at 0 months (continued)

Biomaker_names	Logarithm value	Groups	LDA_value	P_value
beta_carotene_hydroxylase	0.245084902			-
fructose_1_6_bisphosphatase_I	3.388304824			-
serum_resistance_protein	0			-
acyl_carrier_protein_phosphodiesterase	2.47312454			-
guanidinobutyrase	0			-
glutamate_N_acetyltransferase_amino_acid_N_acetyltransferase	2.949589191			-
minimal_PKS_ketosynthase_KSKS_alpha	0			-
two_component_system_NarL_family_captular_synthesis_response_regulator_RcsB	2.47611527			-
two_component_system_OmpR_family_response_regulator_TctD	2.054545219			-
L_iditol_2_dehydrogenase	2.759492546			-
f_4_hydroxy_3_methylbut_2_en_1_yl_diphosphate_reductase	2.616070831			-
prephenate_dehydratase	2.396618762			-
copper_homeostasis_protein_lipoprotein	2.491104919			-
D_methionine_transport_system_permease_protein	2.645262024			-
E_4_hydroxy_3_methylbut_2_enyl_diphosphate_synthase	2.614318998			-
trimethylamine_corrinoid_protein	0			0.0326303
phosphonate_transport_system_permease_protein	2.305048755			-
teichoic_acid_transport_system_ATP_binding_protein	0.657131864			-
f_2_hydroxy_4_carboxymuconate_semialdehyde_hemiacetal_dehydrogenase	0.946173038			-
membrane_fusion_protein_CuIAgI_efflux_system	2.0886949			-
periplasmic_nitrate_reductase_NapA	2.31925963			-
adenosylcobyric_acid_synthase	2.283281674			-
lysine_biosynthesis_protein_LysW___L_2_aminoadipate_ligase	0.203652523			-
dehydrogenasereductase_SDR_family_member_4	0			-
evolved_beta_galactosidase_subunit_alpha	2.22748807			-
MurNAc_alpha_1_phosphate_uridylyltransferase	0.559119092			-
allophycocyanin_beta_subunit	0			-
f_6_phospho_5_dehydro_2_deoxy_D_gluconate_aldolase	0			-
localization_factor_PodJL	1.354020754			-
succinate_dehydrogenase_fumarate_reductase_cytochrome_b_subunit	3.235704528			-

Table S11. KEGG orthology functional terms identified by PICRUSt as different in neonatal jaundice infants (NJI) and non-NJI at 0 months (continued)

Biomaker_names	Logarithm value	Groups	LDA_value	P_value
ATP_binding_cassette_subfamily_B_bacterial_MsbA	2.526186344			-
endonuclease_G_mitochondrial	1.54324115			0.037373
putative_GTP_pyrophosphokinase	1.575518131			-
f_3_oxoacyl_acyl_carrier_protein_reductase	3.726433293			-
pyruvate_kinase	3.791174729			-
sulfotransferase	0			-
glycine_dehydrogenase	3.00458898			-
dolichol_phosphate_mannosyltransferase	2.162202797			-
manganeseiron_transport_system_permease_protein	2.730376564			-
acetate_CoAacetoacetate_CoA_transferase_beta_subunit	1.02867632			-
lysine_decarboxylase	2.52607966			-
macrolide_transport_system_ATP_bindingpermease_protein	2.552420463			-
outer_membrane_protein_OmpU	0			-
AG_specific_adenine_glycosylase	2.647444528			-
host_factor_I_protein	2.985690667			-
GcrA_cell_cycle_regulator	1.409753538			-
DNA_polymerase_III_subunit_alpha_Gram_positive_type	2.780852947			-
uroporphyrin_III_C_methyltransferase_precorrin_2_dehydrogenase_sirohydrochlorin_ferrochelatase	3.089708558			-
photosystem_II_cytochrome_b559_subunit_alpha	0			-
guanine_deaminase	2.184863336			-
glutamine_transport_system_ATP_binding_protein	2.196790878			-
cis_2_3_dihydrobiphenyl_2_3_diol_dehydrogenase	0			-
NADP_dependent_alcohol_dehydrogenase	2.529669754			-
two_component_system_OmpR_family_sensor_histidine_kinase_ResE	0.389895791			-
UDP_glucose_heptosylLPS_alpha_1_3_glucosyltransferase	2.473175011			-
medium_chain_acyl_acyl_carrier_protein_hydrolase	1.601256132			-
f_2_hydroxyglutarate_dehydrogenase	0			-
f_4_amino_4_deoxychorismate_lyase	2.528951798			-
amino_acid_N_acetyltransferase	2.95963529			-
ironzincmanganesecopper_transport_system_ATP_binding_protein	0.994740385			-

Table S11. KEGG orthology functional terms identified by PICRUSt as different in neonatal jaundice infants (NJI) and non-NJI at 0 months (continued)

Biomaker_names	Logarithm value	Groups	LDA_value	P_value
twitching_motility_protein_PilI	1.43846607			-
phosphonopyruvate_decarboxylase	0			0.0163092
pyruvate_decarboxylase	0			-
coccolysin	0.867987176			-
carboxynorspermidine_synthase	0			-
galactosamine_6_phosphate_isomerase	1.748662443			-
acetophenone_carboxylase	0			-
glycerol_3_phosphate_O_acyltransferase	2.774494148			-
glyceraldehyde_3_phosphate_dehydrogenase	3.537992282			-
f_3_oxoadipate_CoA_transferase_alpha_subunit	1.78349518			-
ribulose_bisphosphate_carboxylase_small_chain	1.71847519			-
MSHA_biogenesis_protein_MshE	0.054839378			-
two_component_system_sensor_histidine_kinase_YcbA	1.808716993			-
allophycocyanin_B	0			-
pimeloyl_acyl_carrier_protein_methyl_ester_esterase	2.519668284			-
beta_lactamase_class_C	2.956393074			-
alpha_methylacyl_CoA_racemase	1.765787776			-
two_component_system_NarL_family_sensor_histidine_kinase_BarA	2.466827968			-
flagellar_protein_FliS	2.436729365			-
UDP_3_O_3_hydroxymyristoyl_glucosamine_N_acyltransferase	2.536479759			-
cellulose_1_4_beta_cellobiosidase	0			-
flagellar_protein_FliT	2.384560203			-
aminomethyltransferase	3.15482861			-
f_2_phospho_L_lactate_guanylyltransferase	0.350541146			-
two_component_system_cell_cycle_response_regulator_PopA	0			-
arginase	2.756784408			-
f_3_2_trans_enoyl_CoA_isomerase_mitochondrial	0			-
fructose_1_6_bisphosphatase_II	3.269389724			-
methylaspartate_mutase_sigma_subunit	2.293717528			-
sucrose_6_phosphatase_160_	3.219651291			-

Table S11. KEGG orthology functional terms identified by PICRUSt as different in neonatal jaundice infants (NJI) and non-NJI at 0 months (continued)

Biomaker_names	Logarithm value	Groups	LDA_value	P_value
FO_synthase	0.092003487			-
putrescine_transport_system_substrate_binding_protein	2.020761835			-
chorismate_mutase	2.748638779			-
DNA_excision_repair_protein_ERCC_4	0			-
UDP_glucose_glucosylLPS_alpha_1_3_glucosyltransferase	2.315310742			-
DNA_excision_repair_protein_ERCC_3	1.18317463			-
DNA_excision_repair_protein_ERCC_2	0.561985723			-
deoxyribose_phosphate_aldolase	2.657001527			-
choline_monooxygenase	0			-
prephenate_dehydrogenase	2.636524846			-
N_acylneuraminate_cytidylyltransferase	1.438090868			-
phosphomannomutase	2.800080613			-
thiamine_monophosphate_kinase	2.531248041			-
aminoacrylate_hydrolase	1.965083614			-
sarcosine_oxidase_subunit_delta	1.836902179			-
exodeoxyribonuclease_III	2.706407157			-

Table S12. Unweighted UniFrac distance of each sample between pre-treatment and post-treatment

sample_ID	Standard deviation	Proportion of Variance	Cumulative Proportion
PC1	0.348134542	0.348	0.348
PC2	0.228364249	0.14974	0.49774
PC3	0.181451046	0.09454	0.59227
PC4	0.175337445	0.08827	0.68055
PC5	0.156704958	0.07051	0.75106
PC6	0.147616815	0.06257	0.81362
PC7	0.125386606	0.04514	0.85877
PC8	0.122387378	0.04301	0.90177
PC9	0.116324671	0.03885	0.94063
PC10	0.107304904	0.03306	0.97369
PC11	0.095725982	0.02631	1
PC12	5.34E-17	0	1

Table S13. NMDS Unweighted UniFrac of each sample between pre-treatment and post-treatment

sample_ID	MDS1	MDS2
AI020_1	-0.274986452166265	-0.00698752562427391
BI020_1	-0.0701534628969059	-0.19256024664639
CI006_3	0.115660336479297	0.0875891983377503
CI009_1	-0.216302290273465	-0.0619533323253541
CI013_1	-0.354040162348565	0.0132801292387665
CI017_1	-0.0434113380578255	0.0670804905370953
AI020_3	0.108376517709989	0.119534568402496
BI020_2	0.102028182484933	0.00258624239226179
CI006_4	0.0844321438875497	0.0552965929747917
CI009_2	0.252352223606678	-0.267303015224853
CI013_3	0.0473280059973772	0.136077371479679
CI017_2	0.248716295577203	0.0473595264580299

Table S14. Weighted UniFrac distance of each sample in neonatal jaundice infants (NJI) at 0, 1, 3, 6 and 12 months

sample_ID	Standard deviation	Proportion of Variance	Cumulative Proportion
PC1	0.813902866	0.52743	0.52743
PC2	0.660513349	0.34737	0.8748
PC3	0.26274363	0.05497	0.92976
PC4	0.196841914	0.03085	0.96062
PC5	0.116773323	0.01086	0.97147
PC6	0.094681349	0.00714	0.97861
PC7	0.0744328	0.00441	0.98302
PC8	0.068216812	0.00371	0.98673
PC9	0.055463856	0.00245	0.98918
PC10	0.049304153	0.00194	0.99111
PC11	0.04625224	0.0017	0.99281
PC12	0.043939124	0.00154	0.99435
PC13	0.038270003	0.00117	0.99552
PC14	0.033117488	0.00087	0.99639
PC15	0.029499026	0.00069	0.99708
PC16	0.027556553	6.00E-04	0.99769
PC17	0.024575425	0.00048	0.99817
PC18	0.021993377	0.00039	0.99855
PC19	0.020760262	0.00034	0.9989
PC20	0.018159494	0.00026	0.99916
PC21	0.017311714	0.00024	0.9994
PC22	0.016120578	0.00021	0.99961
PC23	0.014833725	0.00018	0.99978
PC24	0.011962673	0.00011	0.99989
PC25	0.01042083	9.00E-05	0.99998
PC26	0.004897793	2.00E-05	1
PC27	2.54E-16	0	1

Table S15. The identified key OTUs of a heatmap between pre-treatment and post-treatment

OTU	AI020_1	BI020_1	CI006_3	CI009_1	CI013_1	CI017_1	AI020_3	BI020_2	CI006_4	CI009_2	CI013_3	CI017_2
OTU97 (Bacteroides)	3.53E-05	0.00010015	0	0.00075926	0	4.48E-05	0	0	0	0	0	0
OTU310 (Arthrobacter)	0.00153765	0.00057056	0.00010272	0.00338939	0.00158983	0.00029317	0	0	0.000115857	0	0	0
OTU53 (Prevotella)	0.00044224	0.00436718	0	0.00649463	0.00049979	0.00035323	0	0	0	0	0	0
OTU292 (Roseburia)	3.11E-05	0.00017906	0	0.00033417	3.62E-05	0	0	0	0	0	0	0
OTU339 (Pseudobutyrivibrio)	9.64E-05	0.00023672	0	0.00032507	3.96E-05	0	0	0	0	0	0	0
OTU331 (Faecalibacterium)	8.78E-05	0.00342637	0	0.00117299	0.00011421	5.90E-05	0	0	7.43E-05	0	0.000119212	5.36E-05
OTU370 (Escherichia-Shigella)	0.98224095	0.99038552	0.05678	0.9186296	0.97515293	0.97790964	0.093695893	0.57682287	0.093343272	0.000128385	0.634700293	0.964707844
OTU158 (Pelagibacterium)	0.01254966	0	0	0.03007033	0.01627825	0	0	0	0	0	0	0
OTU276 (Nesterenkonia)	0.00097656	0	0.00010272	0.00227778	0.00095435	0	0	0	0	0	0	0
OTU365 (Ralstonia)	0.00034265	0	0	0.00265968	0.00220044	0.00136608	0	0	0.000124601	4.77E-05	0	0
OTU73 (Rhizobium)	4.93E-05	0	0	0.00153898	0.00082432	0.00024329	0	0	0	0	0	0
OTU320 (Sphingomonas)	0.00057716	0	0	0.00245282	0.00181937	9.37E-05	0	0	0	0	0	0
OTU66 (Acinetobacter)	8.99E-05	0.00010015	0.00029313	0.00065014	0.00012325	4.28E-05	0	0	0.000443753	0	0	0
OTU395 (Escherichia-Shigella)	0.00017775	0.00018513	0.0002305	0.00045919	0.00026912	0.00030945	0	0.00017608	0	0	0	0.000213084
OTU256 (Clostridium_sensu_stricto)	5.57E-05	0	0	0	0	4.58E-05	0.000810648	0	0.001182612	0	0.000225177	0.002700509
OTU435 (Dolosigranulum)	0	0	0	0	0	0	0.000180633	0	0	0	0.000145703	0
OTU169 (Gemella)	0	0	0	0	0	4.58E-05	0.002467189	0.00215967	0.00298604	0.000256771	0.000481262	0.000246423
OTU78 (Bifidobacterium)	0.00042296	0	0.03690212	0.00503067	0	0.0145637	0.803453183	0.01532972	0.237175984	0.034515905	0.078948995	0.005327091
OTU153 (Veillonella)	0	9.41E-05	0.03115221	7.27E-05	4.52E-05	0.00029826	0.01053842	0.0101731	0.001630737	0.0011423	0.217940906	0.000310203
OTU145 (Streptococcus)	5.57E-05	0	0.83659495	0.00050238	0	0.00254384	0.065270355	0.34955782	0.6388006	0.858323485	0.046443962	0.001839477
OTU354 (Streptococcus)	2.57E-05	0	0.01682881	0	5.31E-05	0.00168266	0.00857348	0.02535189	0.013598944	0.004600475	0.002666808	0.003976111
OTU172 (Streptococcus)	0	0	0.00051862	0.00039554	0	5.70E-05	0.002167601	0.00171408	0.00049403	0.099340626	0	0.000271066
OTU351 (Streptococcus)	0	0	9.27E-05	0	0	4.78E-05	0.000180633	0.00068635	0	0	0	0.00012756
OTU360 (Enterobacter)	0.00020559	0.00035508	0.01977266	0.02278462	0	0	0.012115658	0.01642213	0.009277273	0.00164432	0.018071598	0.002739647
OTU168 (Actinomyces)	0	0	0	0	0	0	0	0.00048512	0.000115857	0	0.000110381	4.35E-05
OTU280 (Clostridium_sensu_stricto)	0	0	0.00062886	0	0	0	0.000546306	0.00112116	0.000636118	0	0.000145703	0.017443866

Table S16. Faecal bacterial composition in each sample at the phylum level between pre-treatment and post-treatment

phylum	AI020_1	BI020_1	CI006_3	CI009_1	CI013_1	CI017_1	AI020_3	BI020_2	CI006_4	CI009_2	CI013_3	CI017_2
Actinobacteria	0.00642	0.20624	0.0211	0.01522	0.00283	0.018123	0.24912	0.52608	0.296716	0.21893	0.05942	0.09698
Bacteroidetes	0.00115	0.4361	0.00225	0.00453	0.00127	0.000922	0.53125	0.0264	0.000583	0.01676	0.56281	0.00026
Candidate_division_TM7	0	0	4.60E-05	0	0	5.30E-05	0.00102	3.70E-05	8.90E-05	0	5.60E-05	0
Cyanobacteria	4.50E-05	0	0	0	0	0.000773	0	0	0	0	3.20E-05	0
Firmicutes	0.00135	0.00612	0.36994	0.07234	0.0197	0.015382	0.11469	0.12635	0.462884	0.73987	0.11353	0.10127
Fusobacteria	0.00018	0	0	3.30E-05	0.03417	4.10E-05	0	0	0	0	0	0
Proteobacteria	0.99086	0.35154	0.60667	0.90778	0.94204	0.964706	0.10392	0.32113	0.239696	0.02444	0.26414	0.8015
Verrucomicrobia	0	0	0	0.0001	0	0	0	0	3.20E-05	0	0	0

Table S17. Faecal bacterial composition in each sample at the genus level between pre-treatment and post-treatment

genus	AI020 1	BI020 1	CI006 3	CI009 1	CI013 1	CI017 1	AI020 3	BI020 2	CI006 4	CI009 2	CI013 3	CI017 2
Abiotrophia	0	0	0	0	0	######	0	0	0	0	######	0
Acinetobacter	0.0004	######	0.0047	0.0006	0.0004	0.0001	0	######	0.0049	0	######	######
Actinobacillus	0	0	0.0004	0	0	0	0	0	######	0	0	0
Actinomyces	0	0	0.0002	######	0	0.0002	0.0006	0.0006	0.0093	######	######	0.0002
Aerococcus	######	0	0	######	######	0	0	0	0	0	0	0
Aggregatibacter	0	0	0	0	0	0	0	0	0	0	0	######
Akkermansia	0	0	0	0.0001	0	0	0	0	######	0	0	0
Aliihoeflea	0.008	0	0	0.0075	0.0064	0	0	0	0	0	0	0
Alistipes	0	0	0	0	0	0	0.0002	0	0	0	0	0
Alloprevotella	0	0	0	0.0003	0	0	0	0	0	0	0	0
Anaerococcus	######	0	0	0.0003	0	0	0.0033	0.0001	######	0	0	0
Anaerosporobacter	0	0	######	0	0	0	0	0	######	0	0	0
Anaerostipes	0	0.0002	0	0	0	0	0	0.0002	0	0	0	0
Anoxybacillus	######	0	0	0	0	0	0	0	0	0	0	0
Aquabacterium	0.0009	0	0	0.001	0.0012	0	0	0	0	0	0	0
Arthrobacter	0.0014	0.0002	######	0.0015	0.0014	0.0003	0	0	######	0	0	0
Atopobium	0	0	0.0003	0	0	######	0.0009	0.0001	0	0	0.0029	0.0002
Bacteroides	0.0006	0.194	######	0.0008	0.0007	0.0005	0.2947	0.02	0.0001	0.0153	0.5395	0.0003
Bifidobacterium	0.0022	0.2059	0.0185	0.0093	######	0.016	0.1927	0.52	0.2085	0.2128	0.0187	0.0043
Bilophila	0	0	0	0	0	0	0	0	0	0	0.0001	0
Blautia	######	0.0003	######	0	######	0	0.0003	######	0.009	0	0	0
Brevibacterium	######	0	0	0	######	0	0	0	0	0	0	0
Brevundimonas	######	0	######	0	######	0	######	0	######	0	0	0
Campylobacter	0	0	0	0	0	0	0	0	0	0	######	0
Candidate_division_TM7_norank	0	0	######	0	0	######	0.001	######	######	0	######	0
Caulobacter	######	0	0	######	######	0	0	0	0	0	0	0
Chryseobacterium	0	0	0	######	0	######	0	0	######	0	0	0
Citrobacter	0.0003	######	0.0407	0.0006	0	0.0002	0.0059	0.0005	0.0214	######	0.0006	0.0066
Clostridium_sensu_stricto	######	0	0.004	######	0	0.0044	0.0006	0.0021	0.0105	0	0.0013	0.0158
Collinsella	0.0003	######	0	######	######	0	0.0508	0.0003	0	######	0.0359	0
Comamonadaceae_unclassified	0.0008	0	0	0.0008	0.0011	0	0	0	0	0	0	0
Coprobacillus	0	0	0	0	0.0037	0	0	0	0	0	0	0
Coprococcus	0	######	0	0	######	0	0	######	0	0	0	0
Coriobacteriaceae_uncultured	0	0	0	######	0	0	0.0002	0	0	0.0047	0	0
Corynebacterium	0	######	######	0.0029	0.0002	0	######	0.0002	0.0009	0.0013	######	0
Curvibacter	0	0	0	0	0	######	0	0	0	0	######	0
Cyanobacteria_norank	######	0	0	0	0	0.0008	0	0	0	0	######	0
Dermabacter	0	######	0	0	0	0	0	0	0	0	0	0
Dialister	0	0	0	0.0001	0	0	0	0	0	0	0	0
Dolosigranulum	0	0	0	0	0	0	######	0	0	0	######	0
Dorea	0	######	0	0	0	0	0.0005	0	0	0	0	######
Dysgonomonas	0	0	0	0	0	0	0.0002	0	0	0	0	0
Eggerthella	######	0	0	0	0	0	0	0.004	######	######	0.0007	0
Enhydrobacter	0	0	0	0	######	0	0	0	0	0	0	0
Enterobacter	0.0002	0.0003	0.0171	0.0124	0	0	0.0042	0.0073	0.0078	0.0012	0.0068	0.0028
Enterobacteriaceae_unclassified	0.0001	0.0005	0.0792	0.0048	0	######	0.0029	0.0058	0.0193	0.001	0.0088	0.0039
Enterococcus	0	0	0.0003	0.0181	######	0.0005	0.0021	0.0045	0.0174	0	######	0.0474
Erysipelotrichaceae_Incertae_Sedis	0	0	######	0	0.0131	0	0.0009	0	######	0	0.0278	######
Erysipelotrichaceae_uncultured	######	######	0	0	0	0	0.0002	0	######	0	0	0
Escherichia-Shigella	0.9175	0.3264	0.0228	0.4043	0.8626	0.961	0.0213	0.1606	0.0427	######	0.1438	0.6657
Faecalibacterium	######	0.0011	0	0.0005	0.0001	######	0	0	######	0	######	######
Finegoldia	0	0	0	0	0	0	0	0	0.0003	0	0	0
Flavonifractor	0	0	######	0	0.0003	######	######	0	0	0	0.0023	0
Fusobacterium	0.0002	0	0	######	0.0342	######	0	0	0	0	0	0
Gardnerella	0	0	0	0	0	0	0	######	0	0	######	0
Gemella	0	0	0	0	0	######	0.0006	0.0006	0.0014	0.0002	0.0001	0.0002
Gordonibacter	######	0	######	0	0	0	0	0	0.01	0	0	0
Granulicatella	0	0	0.0003	0	0	0	0	0.0001	0.0667	0	######	0
Haemophilus	0	0.0006	0.0003	0.0002	0	0.0001	0.0037	0.0014	######	0	######	0.0001
Halomonas	0.036	0	0	0.0312	0.0403	0	0	0	0	0	0	0
Herbaspirillum	0.0001	0	0	######	0.0004	0	0	0	0	0	0	0
Hyphomonadaceae_norank	0.0018	0	0	0.0012	0.0023	0	0	0	0	0	0	0
Klebsiella	0.0054	0.0236	0.3133	0.4194	0.0003	0.0012	0.0607	0.1441	0.1099	0.0221	0.1035	0.0913
Kocuria	0.0011	0	0	0.0001	0.0001	0	0	0	0	0	0	0
Kytococcus	######	0	0	0	0	0	0	0	0	0	0	0
Lachnoanaerobaculum	0	0	0	0	######	0	0	0	0	0	0	0
Lachnospira	0	######	0	0.0005	0	0	0	0	0	0	0	0
Lachnospiraceae_Incertae_Sedis	0.0002	0.0002	0.0002	0.0004	0.0017	0.0002	0.0667	0.0011	######	0.0089	0.0183	0.0001
Lachnospiraceae_unclassified	0	0.0001	0	######	0	0	0	0	0	0	0	0
Lactobacillales_unclassified	0	0	0.0002	0	0	0	0	######	0	0	0	0
Lactobacillus	0.0001	0.0013	0.0027	0.0008	0	0.0019	0.0067	0.0002	0.0021	0	######	0.0002

Table S17. Faecal bacterial composition in each sample at the genus level between pre-treatment and post-treatment (continued)

genus	AI020 1	BI020 1	CI006 3	CI009 1	CI013 1	CI017 1	AI020 3	BI020 2	CI006 4	CI009 2	CI013 3	CI017 2
Lactococcus	0.0001	0	0	0	0.0002	0	0	######	0	0	0	0
Megamonas	0	0	######	0.0002	0	0	0	0	0	0	######	0
Megasphaera	######	0	0	0.0001	0	0.0002	0	######	0	0	0	0
Methyloversatilis	0	0	0	######	######	0	0	0	0	0	0	0
Microbacteriaceae_unclassified	0.0002	0	0	0.0002	0	0	0	0	0	0	0	0
Micrococcus	0	0	0	0	######	0	0	0	0	0	0	0
Morganella	0	0	0	0	0	0	######	0	0	0	0	0
Negativicoccus	0	0	0	0	0	0	0	0	0.0073	0	######	0
Neisseria	0	######	0	0	0	0	0.0004	0	######	0	0.0001	0
Nesterenkonia	0.0009	0	######	0.001	0.0008	0	0	0	0	0	0	0
Novosphingobium	0.0004	0	0	0.0004	0.0005	0	0	0	0	0	0	0
Oceanobacillus	0	0	0	0	######	0	0	0	0	0	0	0
Parabacteroides	######	0.2407	0.0021	######	######	######	0.2362	0.0063	0.0004	0.0015	0.0233	0
Paraprevotella	######	0	0	0	0	0	0	0	0	0	0	0
Parascardovia	0	0	0	0	0	0	0	0	0	0	0.0002	0
Parasutterella	0	0	0	0	0.0002	0	0	0	0	0	0	0
Pediococcus	0	0	0	0.0005	0	0	0	0	0	0	0	0
Pelagibacterium	0.0117	0	0	0.0132	0.0144	0	0	0	0	0	0	0
Pelomonas	0.0009	0	0	0.0011	0.0009	0	0	0	0	0	0	0
Peptoniphilus	0	0	0	0	0	0	######	######	0	0	0	0
Peptostreptococcaceae_Incertae_Sedis	0	######	0.0001	0	0	0.0001	0.0006	######	0.0002	0	0	0.0009
Peptostreptococcus	0	0	0	0	0	0	0	0	0	0	0	######
Phascolarctobacterium	0	0	0	0	0	0	######	0	0	0	0.001	0
Phyllobacteriaceae_unclassified	0.0009	0	0	0.0012	0.0012	0	0	0	0	0	0	0
Porphyromonas	0	0	######	0	0	0	0	0	0	0	0	0
Prevotella	0.0004	0.0014	0	0.0034	0.0005	0.0003	0	0.0001	0	0	0	0
Propionibacterium	0	0	0.0001	0	0	0	0	0.0002	0.0655	0	0	0
Proteus	0	0	0	0	0	0	######	0	0	0	0	0
Pseudobutyrivibrio	######	######	0	0.0001	######	0	0	0	0	0	0	0
Pseudochrobactrum	######	0	0	######	######	0	0	0	######	0	0	0
Psychrobacter	0	0	0	0	0.0002	0	0	0	0	0	0	0
Ralstonia	0.0003	0	0	0.0012	0.0019	0.0013	0	0	######	######	0	0
Raoultella	######	0	0.0083	0.0001	0	######	0.0007	######	0.0047	0	######	0.001
Rhizobium	######	0	0	0.0007	0.0007	0.0002	0	0	0	0	0	0
Rhodobacter	0.0003	0	0	0	0	0	0	0	0	0	0	0
Roseburia	######	######	0	0.0001	######	0	0	0	0	0	0	0
Rothia	0	0	0.0016	0	0	0.0015	0.0033	0.0006	0.0024	0	0.0008	0.0922
Ruminococcaceae_Incertae_Sedis	0	0	0	0	0	0	######	0	0	0	0	0
Ruminococcaceae_uncultured	######	0.0002	0	0	0	######	######	0	0	0	0.001	0
Ruminococcus	######	0.0001	0	0	0	0	0	0.0001	0	0	0	######
S24-7_norank	######	0	0	0	0	0	0	0	0	0	0	0
Saccharopolyspora	0.0001	0	0	0.0001	######	0	0	0	0	0	0	0
Salmonella	0.0002	0	0.1181	0.0005	0	######	0.0011	0.0007	0.0283	0	0.0002	0.03
Scardovia	0	0	0.0001	0	0	0	0.0004	0	0	0	0	0
Serratia	######	######	0.0017	0.0002	0.0002	######	0.0002	0.0006	0.0005	0	0.0002	0
Sphingomonas	0.0005	0	0	0.0011	0.0016	######	0	0	0	0	0	0
Sphingopyxis	0	######	0	0	0	0	0	0	0	0	0	0
Staphylococcus	######	0.0019	0.0009	0.0497	0.0003	0.0005	0.0007	0.0022	0.0234	0.1453	0.0006	0.0022
Stenotrophomonas	######	0	0	0	0	0	0	0	0	0	0	0
Streptococcus	0.0001	0	0.3484	0.0004	######	0.0069	0.0287	0.1114	0.3233	0.5847	0.0115	0.0341
Subdoligranulum	######	0.0002	0	######	0	0	0	######	0	0	0	######
Sutterella	0	######	0	######	0	0	0.0025	0	0	0	0	0
Veillonella	0	######	0.0126	######	######	0.0004	0.0025	0.0032	0.001	0.0007	0.0494	0.0002
Weissella	0	0	0	0.0001	0	0	0	0	0	0	0	0
Xanthomonadaceae_uncultured	0.0036	0	0	0.0037	0.0049	0	0	0	0	0	0	0
Zoogloea	######	0	0	0.0001	######	0	0	0	0	0	0	0

Table S18. Microbial composition and comparison at the phylum level between pre-treatment (0 months) and post-treatment (1 month)

ID	M0-median	M0-mean	M0-se	M1-median	M1-mean	M1-se	p-value	z-score	Sig_mark	q-value
Proteobacteria	0.9249(0.6819,0.959)	0.7939317	0.10545412	0.2519(0.1379,0.3069)	0.29246983	0.11119159	0.008658	-2.6252708	**	0.034632
Firmicutes	0.0175(0.0084,0.0592)	0.0808028	0.05875832	0.1205(0.1138,0.3788)	0.276433	0.10884938	0.025974	-2.2265999	*	0.0568813
Actinobacteria	0.0167(0.0086,0.0204)	0.044988	0.03237647	0.234(0.1275,0.2848)	0.241207	0.06798037	0.008658	-2.6252708	**	0.034632
Bacteroidetes	0.0018(0.0012,0.004)	0.0743705	0.07234794	0.0216(0.0046,0.405)	0.1896785	0.11315197	0.5887446	-0.5406562		0.672851
Fusobacteria	<0.0001(<0.0001,0.0001)	0.0057372	0.00568623	<0.0001(<0.0001,<0.0001)	0	0	0.0284407	-2.1911538	*	0.0568813
Candidate_division_TM7	<0.0001(<0.0001,<0.0001)	0.0000165	1.0475E-05	<0.0001(<0.0001,<0.0001)	0.000201	0.00016519	0.199856	-1.2819619		0.3197696
Cyanobacteria	<0.0001(<0.0001,<0.0001)	0.0001363	0.00012755	<0.0001(<0.0001,<0.0001)	5.3333E-06	5.3333E-06	0.4619505	-0.7356389		0.6159339
Verrucomicrobia	<0.0001(<0.0001,<0.0001)	0.000017	0.000017	<0.0001(<0.0001,<0.0001)	5.3333E-06	5.3333E-06	1	0		1

Table S19. Difference of fecal microbial communities at the genus level between pre-treatment (M0) and post-treatment (M1)

ID	M0-median	M0-mean	M0-se	M1-median	M1-mean	M1-se	p-value	z-score	Sig_mark	q-value
Escherichia-Shigella	0.6335(0.3459,0.9038)	0.58242367	0.1575417	0.0932(0.0266,0.1564)	0.1723395	0.1022471	0.0649351	-1.8457049		0.25374847
Streptococcus	0.0003(<0.0001,0.0053)	0.0593175	0.0578301	0.0728(0.0301,0.2703)	0.182291	0.0933745	0.0411255	-2.04226208	*	0.25374847
Bifidobacterium	0.0127(0.0039,0.0179)	0.04198917	0.0329173	0.2006(0.0622,0.2117)	0.192825	0.0760554	0.0649351	-1.8457049		0.25374847
Klebsiella	0.0145(0.0023,0.2409)	0.12720433	0.0769365	0.0974(0.0683,0.1083)	0.0885875	0.0172816	0.4848485	-0.69852571		0.50592885
Bacteroides	0.0007(0.0005,0.0008)	0.0327845	0.0322383	0.0176(0.004,0.226)	0.1449755	0.0917315	0.3939394	-0.85249503		0.49760766
Parabacteroides	<0.0001(<0.0001,0.0016)	0.04050633	0.0400374	0.0039(0.0007,0.019)	0.0446032	0.0384799	0.4848485	-0.69852571		0.50592885
Staphylococcus	0.0007(0.0003,0.0016)	0.0088765	0.0081675	0.0022(0.0011,0.0181)	0.0290685	0.0235286	0.1796537	-1.34182212		0.43116883
Salmonella	0.0001(<0.0001,0.0005)	0.01981917	0.0196627	0.0009(0.0003,0.0215)	0.0100338	0.0060517	0.3751171	-0.88692899		0.49760766
Enterobacteriaceae_unclassified	0.0003(<0.0001,0.0037)	0.01412183	0.0130447	0.0048(0.0032,0.008)	0.0069492	0.0026948	0.1796537	-1.34182212		0.43116883
Halomonas	0.0156(<0.0001,0.0348)	0.017924	0.0081017	<0.0001(<0.0001,<0.0001)	0	0	0.07401	-1.78655173		0.25374847
Rothia	<0.0001(<0.0001,0.0012)	0.00051733	0.0003272	0.0016(0.0007,0.0031)	0.0165757	0.0151319	0.1147105	-1.57736972		0.34413143
Lachnospiraceae_Incertae_Sedis	0.0002(0.0002,0.0004)	0.0004935	0.0002336	0.005(0.0004,0.0159)	0.0158612	0.0105646	0.4848485	-0.69852571		0.50592885
Enterococcus	0.0002(<0.0001,0.0005)	0.003172	0.0029947	0.0033(0.0005,0.0142)	0.0118952	0.0075842	0.3751171	-0.88692899		0.49760766
Collinsella	<0.0001(<0.0001,<0.0001	0.000076	5.377E-05	0.0002(<0.0001,0.027)	0.014508	0.0093216	0.3699763	-0.89651769		0.49760766
Citrobacter	0.0003(<0.0001,0.0005)	0.0069805	0.0067483	0.0032(0.0005,0.0064)	0.0058293	0.0033236	0.3939394	-0.85249503		0.49760766
Veillonella	<0.0001(<0.0001,0.0003)	0.00218483	0.0020851	0.0017(0.0008,0.003)	0.0094995	0.0079927	0.0649351	-1.8457049		0.25374847
Granulicatella	<0.0001(<0.0001,<0.0001	0.0000445	0.0000445	<0.0001(<0.0001,<0.0001)	0.0111422	0.0111118	0.3407545	-0.95267558		0.49760766
Propionibacterium	<0.0001(<0.0001,<0.0001	1.9167E-05	1.917E-05	<0.0001(<0.0001,0.0002)	0.0109518	0.0109061	0.4619505	-0.73563895		0.50592885
Enterobacter	0.0003(<0.0001,0.0094)	0.00501517	0.0031502	0.0055(0.0032,0.0072)	0.0050182	0.0011028	0.3776424	-0.88224859		0.49760766
Erysipelotrichaceae_Incertae_Sedis	<0.0001(<0.0001,<0.0001	0.002189	0.0021796	<0.0001(<0.0001,0.0007)	0.0048015	0.0045997	0.3471635	-0.94010539		0.49760766
Pelagibacterium	0.0059(<0.0001,0.0129)	0.00655733	0.0029529	<0.0001(<0.0001,<0.0001)	0	0	0.07401	-1.78655173		0.25374847
Clostridium_sensu_stricto	<0.0001(<0.0001,0.003)	0.00141183	0.0008757	0.0017(0.0008,0.0084)	0.0050547	0.0026619	0.2945516	-1.0481888		0.49760766
Fusobacterium	<0.0001(<0.0001,0.0001)	0.00573717	0.0056862	<0.0001(<0.0001,<0.0001)	0	0	0.0284407	-2.19115383	*	0.25374847
Gordonibacter	<0.0001(<0.0001,<0.0001	0.000012	7.937E-06	<0.0001(<0.0001,<0.0001)	0.0016725	0.0016725	0.7525538	-0.31527383		0.75255375

Table S20. Detailed data for linear discriminant analysis (LDA) of the faecal microbial OTUs between pre-treatment (M0) and post-treatment (M1)

Biomaker_names	Logarithm value	Groups	LDA_value	P_value
d__Bacteria.p__Firmicutes.c__Negativicutes.o__Selenomonadales.f__Acidaminococcaceae	2.243864489			-
d__Bacteria.p__Proteobacteria.c__Deltaproteobacteria	1.297395711			-
d__Bacteria.p__Firmicutes.c__Clostridia.o__Clostridiales.f__Lachnospiraceae.g__Blautia	3.195484523			-
d__Bacteria.p__Proteobacteria.c__Gammaproteobacteria.o__Enterobacteriales	5.879302651	M0	5.417114674	0.01040562
d__Bacteria.p__Firmicutes.c__Bacilli.o__Bacillales	4.470736261			-
d__Bacteria.p__Proteobacteria.c__Gammaproteobacteria.o__Oceanospirillales.f__Halomonadaceae.g__Halomonas	4.253434935			-
d__Bacteria.p__Bacteroidetes	5.278018107			-
d__Bacteria.p__Proteobacteria.c__Alphaproteobacteria.o__Sphingomonadales.f__Sphingomonadaceae	2.89799343	M0	3.11417114	0.00739713
d__Bacteria.p__Bacteroidetes.c__Bacteroidia.o__Bacteroidales.f__Bacteroidaceae	5.161294615			-
d__Bacteria.p__Actinobacteria.c__Actinobacteria.o__Actinomycetales.f__Actinomycetaceae	3.255071396	M1	3.004226738	0.03604871
d__Bacteria.p__Firmicutes.c__Clostridia.o__Clostridiales.f__Clostridiaceae	3.703692521			-
d__Bacteria.p__Firmicutes.c__Clostridia.o__Clostridiales.f__Clostridiales_Family_XI.g__Anaerococcus	2.756889563			-
d__Bacteria.p__Actinobacteria.c__Actinobacteria.o__Micrococcales.f__Micrococcaceae.g__Nesterenkonia	2.668851648	M0	3.371165928	0.02222962
d__Bacteria.p__Proteobacteria.c__Betaproteobacteria.o__Rhodocyclales.f__Rhodocyclaceae	1.722908012			-
d__Bacteria.p__Proteobacteria.c__Epsilonproteobacteria.o__Campylobacterales	0.684246748			-
d__Bacteria.p__Proteobacteria.c__Betaproteobacteria.o__Burkholderiales.f__Alcaligenaceae.g__Sutterella	2.621695462			-
d__Bacteria.p__Firmicutes.c__Erysipelotrichia	3.685278842			-
d__Bacteria.p__Verrucomicrobia.c__Verrucomicrobiae	1.230448921			-
d__Bacteria.p__Proteobacteria.c__Alphaproteobacteria.o__Caulobacterales.f__Caulobacteraceae	1.67669361			-
d__Bacteria.p__Proteobacteria.c__Alphaproteobacteria.o__Rhizobiales.f__Hyphomicrobiaceae.g__Pelagibacterium	3.816727261			-
d__Bacteria.p__Firmicutes.c__Clostridia.o__Clostridiales.f__Lachnospiraceae	4.244330734			-
d__Bacteria.p__Firmicutes.c__Erysipelotrichia.o__Erysipelotrichales	3.685278842			-
d__Bacteria.p__Firmicutes.c__Negativicutes.o__Selenomonadales	4.037884457			-
d__Bacteria.p__Proteobacteria.c__Betaproteobacteria.o__Burkholderiales.f__Comamonadaceae.g__Curvibacter	0.91204483			-
d__Bacteria.p__Proteobacteria.c__Betaproteobacteria.o__Burkholderiales.f__Burkholderiaceae.g__Ralstonia	2.901094895			-
d__Bacteria.p__Actinobacteria	5.382389907	M1	5.031826563	0.01040562
d__Bacteria.p__Proteobacteria.c__Gammaproteobacteria.o__Pasteurellales.f__Pasteurellaceae	2.964181032			-
d__Bacteria.p__Proteobacteria.c__Gammaproteobacteria.o__Pasteurellales	2.964181032			-
d__Bacteria.p__Proteobacteria.c__Alphaproteobacteria.o__Rhodobacterales	1.673635185			-
d__Bacteria.p__Firmicutes.c__Clostridia.o__Clostridiales.f__Lachnospiraceae.g__Dorea	1.93026965			-
d__Bacteria.p__Firmicutes.c__Clostridia.o__Clostridiales.f__Ruminococcaceae.g__Faecalibacterium	2.497390438	M0	2.932228701	0.02776836
d__Bacteria.p__Proteobacteria.c__Gammaproteobacteria.o__Enterobacteriales.f__Enterobacteriaceae.g__Raoultella	3.152594078			-
d__Bacteria.p__Verrucomicrobia.c__Verrucomicrobiae.o__Verrucomicrobiales.f__Verrucomicrobiaceae.g__Akkermansia	1.230448921			-
d__Bacteria.p__Actinobacteria.c__Actinobacteria.o__Micrococcales.f__Micrococcaceae.g__Micrococcus	1.156347201			-
d__Bacteria.p__Proteobacteria.c__Gammaproteobacteria.o__Pasteurellales.f__Pasteurellaceae.g__Haemophilus	2.952388786			-
d__Bacteria.p__Proteobacteria.c__Alphaproteobacteria.o__Rhizobiales.f__Brucellaceae	1.243038049			-
d__Bacteria.p__Fusobacteria.c__Fusobacteriia.o__Fusobacteriales	3.758697466	M0	3.801438344	0.02222962

Table S20. Detailed data for linear discriminant analysis (LDA) of the faecal microbial OTUs between pre-treatment (M0) and post-treatment (M1) (continued)

Biomaker_names	Logarithm value	Groups	LDA_value	P_value
d__Bacteria.p__Proteobacteria.c__Alphaproteobacteria.o__Sphingomonadales.f__Sphingomonadaceae.g__Novosphingobium	2.35666312			-
d__Bacteria.p__Firmicutes.c__Bacilli.o__Bacillales.f__Staphylococcaceae	4.463422622			-
d__Bacteria.p__Firmicutes.c__Bacilli.o__Lactobacillales.f__Lactobacillales_unclassified.g__Lactobacillales_unclassified	1.51851394			-
d__Bacteria.p__Firmicutes.c__Bacilli.o__Lactobacillales.f__Streptococcaceae.g__Streptococcus	5.260765227	M1	4.831868029	0.03737299
d__Bacteria.p__Firmicutes.c__Clostridia.o__Clostridiales	4.383584997	M1	4.032429867	0.01040562
d__Bacteria.p__Proteobacteria.c__Betaproteobacteria.o__Burkholderiales.f__Comamonadaceae.g__Aquabacterium	2.709552613			-
d__Bacteria.p__Actinobacteria.c__Actinobacteria.o__Bifidobacteriales.f__Bifidobacteriaceae.g__Bifidobacterium	5.28516334			-
d__Bacteria.p__Proteobacteria.c__Deltaproteobacteria.o__Desulfovibrionales.f__Desulfovibrionaceae.g__Bilophila	1.297395711			-
d__Bacteria.p__Proteobacteria.c__Gammaproteobacteria.o__Xanthomonadales.f__Xanthomonadaceae	3.30763864			-
d__Bacteria.p__Firmicutes.c__Bacilli	5.373811863			-
d__Bacteria.p__Actinobacteria.c__Actinobacteria.o__Micrococcales.f__Micrococcaceae	4.219702383			-
d__Bacteria.p__Proteobacteria.c__Gammaproteobacteria.o__Xanthomonadales.f__Xanthomonadaceae.g__Stenotrophomonas	0.56427143			-
d__Bacteria.p__Actinobacteria.c__Actinobacteria	5.382389907	M1	5.031826563	0.01040562
d__Bacteria.p__Bacteroidetes.c__Flavobacteriia.o__Flavobacteriales.f__Flavobacteriaceae	1.140926842			-
d__Bacteria.p__Firmicutes.c__Bacilli.o__Bacillales.f__Bacillales_Family_XI.g__Gemella	2.693433804	M1	3.237410583	0.00280167
d__Bacteria.p__Proteobacteria.c__Gammaproteobacteria.o__Enterobacteriales.f__Enterobacteriaceae.g__Proteus	1.185636577			-
d__Bacteria.p__Firmicutes.c__Clostridia.o__Clostridiales.f__Ruminococcaceae.g__Ruminococcus	1.467361417			-
d__Bacteria.p__Firmicutes.c__Negativicutes.o__Selenomonadales.f__Veillonellaceae.g__Megamonas	1.669006781			-
d__Bacteria.p__Actinobacteria.c__Actinobacteria.o__Coriobacteriales.f__Coriobacteriaceae.g__Coriobacteriaceae_uncultured	2.912487761			-
d__Bacteria.p__Firmicutes.c__Negativicutes	4.037884457			-
d__Bacteria.p__Actinobacteria.c__Actinobacteria.o__Micrococcales.f__Dermabacteraceae.g__Dermabacter	0.740362689			-
d__Bacteria.p__Firmicutes.c__Bacilli.o__Lactobacillales.f__Streptococcaceae.g__Lactococcus	1.713210443			-
d__Bacteria.p__Proteobacteria.c__Betaproteobacteria.o__Burkholderiales.f__Comamonadaceae	3.159667434	M0	3.313622257	0.04951017
d__Bacteria.p__Proteobacteria.c__Alphaproteobacteria.o__Sphingomonadales.f__Sphingomonadaceae.g__Sphingomonas	2.742856002	M0	2.914086331	0.02222962
d__Bacteria.p__Proteobacteria.c__Alphaproteobacteria.o__Rhizobiales.f__Hyphomicrobiaceae	3.816727261			-
d__Bacteria.p__Cyanobacteria.c__Cyanobacteria.o__Cyanobacteria_norank.f__Cyanobacteria_norank	2.134602053			-
d__Bacteria.p__Proteobacteria.c__Alphaproteobacteria.o__Caulobacterales.f__Hyphomonadaceae	2.945550644			-
d__Bacteria.p__Proteobacteria.c__Gammaproteobacteria.o__Enterobacteriales.f__Enterobacteriaceae.g__Salmonella	4.29708539			-
d__Bacteria.p__Firmicutes.c__Clostridia.o__Clostridiales.f__Peptostreptococcaceae.g__Peptostreptococcus	0.619788758			-
d__Bacteria.p__Bacteroidetes.c__Bacteroidia.o__Bacteroidales.f__Prevotellaceae.g__Alloprevotella	1.643452676			-
d__Bacteria.p__Fusobacteria.c__Fusobacteriia.o__Fusobacteriales.f__Fusobacteriaceae.g__Fusobacterium	3.758697466	M0	3.802261723	0.02222962
d__Bacteria.p__Bacteroidetes.c__Bacteroidia.o__Bacteroidales.f__Prevotellaceae.g__Paraprevotella	0.602059991			-
d__Bacteria.p__Proteobacteria.c__Betaproteobacteria.o__Burkholderiales.f__Oxalobacteraceae	2			-
d__Bacteria.p__Proteobacteria.c__Deltaproteobacteria.o__Desulfovibrionales	1.297395711			-
d__Bacteria.p__Candidate_division_TM7.c__Candidate_division_TM7_norank.o__Candidate_division_TM7_norank.f__Candidate_division_TM7_norank.g__Candidate_division_TM7_norank	2.303196057			-
d__Bacteria.p__Firmicutes.c__Clostridia.o__Clostridiales.f__Lachnospiraceae.g__Pseudobutyrivibrio	1.760924848	M0	3.36101179	0.02222962
d__Bacteria.p__Firmicutes.c__Negativicutes.o__Selenomonadales.f__Veillonellaceae.g__Megasphaera	1.691670766			-

Table S20. Detailed data for linear discriminant analysis (LDA) of the faecal microbial OTUs between pre-treatment (M0) and post-treatment (M1) (continued)

Biomaker_names	Logarithm value	Groups	LDA_value	P_value
d__Bacteria.p__Firmicutes.c__Bacilli.o__Lactobacillales.f__Carnobacteriaceae.g__Dolosigranulum	1.091080469			-
d__Bacteria.p__Cyanobacteria	2.134602053			-
d__Bacteria.p__Firmicutes.c__Clostridia.o__Clostridiales.f__Lachnospiraceae.g__Lachnospiraceae_unclassified	1.477121255			-
d__Bacteria.p__Proteobacteria.c__Alphaproteobacteria.o__Rhizobiales.f__Rhizobiaceae	2.449992357	M0	2.984333323	0.02222962
d__Bacteria.p__Firmicutes.c__Clostridia.o__Clostridiales.f__Ruminococcaceae.g__Subdoligranulum	1.80048796			-
d__Bacteria.p__Firmicutes.c__Bacilli.o__Lactobacillales.f__Leuconostocaceae.g__Weissella	1.263241435			-
d__Bacteria.p__Proteobacteria.c__Betaproteobacteria.o__Burkholderiales.f__Comamonadaceae.g__Comamonadaceae_unclassified	2.652407519			-
d__Bacteria.p__Actinobacteria.c__Actinobacteria.o__Propionibacteriales.f__Propionibacteriaceae.g__Propionibacterium	4.039486826			-
d__Bacteria.p__Proteobacteria.c__Betaproteobacteria.o__Burkholderiales.f__Comamonadaceae.g__Pelomonas	2.676388735			-
d__Bacteria.p__Proteobacteria.c__Alphaproteobacteria	4.107933191	M0	3.831959118	0.00334562
d__Bacteria.p__Cyanobacteria.c__Cyanobacteria.o__Cyanobacteria_norank.f__Cyanobacteria_norank.g__Cyanobacteria_norank	2.134602053			-
d__Bacteria.p__Firmicutes.c__Clostridia.o__Clostridiales.f__Lachnospiraceae.g__Coprococcus	1.315270435			-
d__Bacteria.p__Proteobacteria.c__Betaproteobacteria.o__Burkholderiales.f__Alcaligenaceae.g__Parasutterella	1.474701781			-
d__Bacteria.p__Firmicutes.c__Bacilli.o__Bacillales.f__Bacillaceae.g__Anoxybacillus	1.054357662			-
d__Bacteria.p__Proteobacteria.c__Gammaproteobacteria.o__Pseudomonadales.f__Moraxellaceae.g__Acinetobacter	3.018214905			-
d__Bacteria.p__Actinobacteria.c__Actinobacteria.o__Bifidobacteriales.f__Bifidobacteriaceae.g__Gardnerella	1.091080469			-
d__Bacteria.p__Firmicutes.c__Clostridia.o__Clostridiales.f__Clostridiales_Family_XI	2.803343292			-
d__Bacteria.p__Cyanobacteria.c__Cyanobacteria	2.134602053			-
d__Bacteria.p__Firmicutes.c__Bacilli.o__Lactobacillales.f__Leuconostocaceae	1.263241435			-
d__Bacteria.p__Firmicutes.c__Negativicutes.o__Selenomonadales.f__Veillonellaceae.g__Negativicoccus	3.087958789			-
d__Bacteria.p__Cyanobacteria.c__Cyanobacteria.o__Cyanobacteria_norank	2.134602053			-
d__Bacteria.p__Proteobacteria	5.899783125	M0	5.445782821	0.01040562
d__Bacteria.p__Candidate_division_TM7	2.303196057			-
d__Bacteria.p__Bacteroidetes.c__Bacteroidia	5.277995591			-
d__Bacteria.p__Actinobacteria.c__Actinobacteria.o__Bifidobacteriales.f__Bifidobacteriaceae.g__Scardovia	1.857332496			-
d__Bacteria.p__Firmicutes.c__Negativicutes.o__Selenomonadales.f__Veillonellaceae	4.030849245			-
d__Bacteria.p__Proteobacteria.c__Alphaproteobacteria.o__Sphingomonadales.f__Sphingomonadaceae.g__Sphingopyxis	1.007178585			-
d__Bacteria.p__Proteobacteria.c__Gammaproteobacteria.o__Enterobacteriales.f__Enterobacteriaceae.g__Enterobacter	3.700545081			-
d__Bacteria.p__Proteobacteria.c__Alphaproteobacteria.o__Rhizobiales.f__Rhizobiaceae.g__Rhizobium	2.449992357	M0	2.98438572	0.02222962
d__Bacteria.p__Verrucomicrobia	1.230448921			-
d__Bacteria.p__Firmicutes.c__Bacilli.o__Bacillales.f__Bacillaceae	1.371067862			-
d__Bacteria.p__Proteobacteria.c__Deltaproteobacteria.o__Desulfovibrionales.f__Desulfovibrionaceae	1.297395711			-
d__Bacteria.p__Actinobacteria.c__Actinobacteria.o__Coriobacteriales	4.266827286	M1	3.985277976	0.01040562
d__Bacteria.p__Proteobacteria.c__Alphaproteobacteria.o__Caulobacterales.f__Caulobacteraceae.g__Brevundimonas	1.459894853			-
d__Bacteria.p__Bacteroidetes.c__Bacteroidia.o__Bacteroidales.f__Bacteroidaceae.g__Bacteroides	5.161294615			-
d__Bacteria.p__Proteobacteria.c__Gammaproteobacteria.o__Enterobacteriales.f__Enterobacteriaceae	5.879302651	M0	5.417114674	0.01040562
d__Bacteria.p__Proteobacteria.c__Alphaproteobacteria.o__Rhizobiales.f__Phyllobacteriaceae.g__Phyllobacteriaceae_unclassified	2.743117625			-

Table S20. Detailed data for linear discriminant analysis (LDA) of the faecal microbial OTUs between pre-treatment (M0) and post-treatment (M1) (continued)

Biomaker_names	Logarithm value	Groups	LDA_value	P_value
d__Bacteria.p__Proteobacteria.c__Alphaproteobacteria.o__Rhizobiales.f__Brucellaceae.g__Pseudochrobactrum	1.243038049			-
d__Bacteria.p__Proteobacteria.c__Alphaproteobacteria.o__Rhizobiales	4.043512912	M0	3.77189042	0.04951017
d__Bacteria.p__Proteobacteria.c__Gammaproteobacteria.o__Enterobacteriales.f__Enterobacteriaceae.g__Citrobacter	3.843886531			-
d__Bacteria.p__Actinobacteria.c__Actinobacteria.o__Propionibacteriales.f__Propionibacteriaceae	4.039486826			-
d__Bacteria.p__Bacteroidetes.c__Bacteroidia.o__Bacteroidales.f__S24_7	0.602059991			-
d__Bacteria.p__Candidate_division_TM7.c__Candidate_division_TM7_norank.o__Candidate_division_TM7_norank.f__Candidate_di	2.303196057			-
d__Bacteria	6			-
d__Bacteria.p__Firmicutes.c__Erysipelotrichia.o__Erysipelotrichales.f__Erysipelotrichaceae.g__Coprobacillus	2.786278077			-
d__Bacteria.p__Actinobacteria.c__Actinobacteria.o__Micrococcales.f__Brevibacteriaceae.g__Brevibacterium	1.171238756			-
d__Bacteria.p__Proteobacteria.c__Gammaproteobacteria.o__Pseudomonadales.f__Moraxellaceae.g__Psychrobacter	1.568201724			-
d__Bacteria.p__Firmicutes.c__Clostridia.o__Clostridiales.f__Peptostreptococcaceae.g__Peptostreptococcaceae_Incertae_Sedis	2.467608106			-
d__Bacteria.p__Actinobacteria.c__Actinobacteria.o__Micrococcales.f__Microbacteriaceae.g__Microbacteriaceae_unclassified	1.73239376			-
d__Bacteria.p__Firmicutes.c__Clostridia.o__Clostridiales.f__Peptostreptococcaceae	2.473730204			-
d__Bacteria.p__Firmicutes.c__Clostridia.o__Clostridiales.f__Lachnospiraceae.g__Roseburia	1.648360011	M0	3.444541869	0.02222962
d__Bacteria.p__Proteobacteria.c__Betaproteobacteria.o__Burkholderiales.f__Alcaligenaceae	2.621695462			-
d__Bacteria.p__Firmicutes.c__Clostridia.o__Clostridiales.f__Ruminococcaceae.g__Ruminococcaceae_uncultured	2.261659304			-
d__Bacteria.p__Proteobacteria.c__Epsilonproteobacteria.o__Campylobacterales.f__Campylobacteraceae	0.684246748			-
d__Bacteria.p__Actinobacteria.c__Actinobacteria.o__Corynebacteriales.f__Corynebacteriaceae.g__Corynebacterium	2.732125594			-
d__Bacteria.p__Proteobacteria.c__Gammaproteobacteria.o__Pseudomonadales	3.037891091			-
d__Bacteria.p__Firmicutes.c__Clostridia.o__Clostridiales.f__Ruminococcaceae	2.810792392			-
d__Bacteria.p__Proteobacteria.c__Alphaproteobacteria.o__Rhizobiales.f__Phyllobacteriaceae.g__Aliihoeflea	3.56153864			-
d__Bacteria.p__Firmicutes.c__Negativicutes.o__Selenomonadales.f__Veillonellaceae.g__Veillonella	3.977700747			-
d__Bacteria.p__Proteobacteria.c__Gammaproteobacteria.o__Pseudomonadales.f__Moraxellaceae	3.037891091			-
d__Bacteria.p__Firmicutes.c__Erysipelotrichia.o__Erysipelotrichales.f__Erysipelotrichaceae.g__Erysipelotrichaceae_uncultured	1.636822098			-
d__Bacteria.p__Bacteroidetes.c__Bacteroidia.o__Bacteroidales.f__Rikenellaceae.g__Alistipes	1.560305243			-
d__Bacteria.p__Firmicutes.c__Bacilli.o__Lactobacillales.f__Lactobacillaceae.g__Pediococcus	1.908485019			-
d__Bacteria.p__Actinobacteria.c__Actinobacteria.o__Bifidobacteriales.f__Bifidobacteriaceae	5.285431278			-
d__Bacteria.p__Actinobacteria.c__Actinobacteria.o__Corynebacteriales.f__Corynebacteriaceae	2.732125594			-
d__Bacteria.p__Firmicutes.c__Clostridia.o__Clostridiales.f__Lachnospiraceae.g__Anaerostipes	1.486666573			-
d__Bacteria.p__Firmicutes.c__Clostridia.o__Clostridiales.f__Clostridiales_Family_XI.g__Finegoldia	1.714609139			-
d__Bacteria.p__Actinobacteria.c__Actinobacteria.o__Pseudonocardiales	1.736396502			-
d__Bacteria.p__Actinobacteria.c__Actinobacteria.o__Micrococcales.f__Micrococcaceae.g__Kocuria	2.345700391			-
d__Bacteria.p__Bacteroidetes.c__Bacteroidia.o__Bacteroidales.f__Porphyromonadaceae.g__Porphyromonas	0.875061263			-
d__Bacteria.p__Actinobacteria.c__Actinobacteria.o__Coriobacteriales.f__Coriobacteriaceae.g__Collinsella	4.161607547			-
d__Bacteria.p__Actinobacteria.c__Actinobacteria.o__Micrococcales.f__Dermacoccaceae.g__Kytococcus	0.684246748			-
d__Bacteria.p__Bacteroidetes.c__Flavobacteriia.o__Flavobacteriales	1.140926842			-
d__Bacteria.p__Firmicutes.c__Bacilli.o__Lactobacillales.f__Streptococcaceae	5.260795007	M1	4.831791661	0.03737299

Table S20. Detailed data for linear discriminant analysis (LDA) of the faecal microbial OTUs between pre-treatment (M0) and post-treatment (M1) (continued)

Biomaker_names	Logarithm value	Groups	LDA_value	P_value
d__Bacteria.p__Actinobacteria.c__Actinobacteria.o__Propionibacteriales	4.039486826			-
d__Bacteria.p__Fusobacteria	3.758697466	M0	3.802015584	0.02222962
d__Bacteria.p__Firmicutes.c__Clostridia.o__Clostridiales.f__Lachnospiraceae.g__Lachnospiraceae_Incertae_Sedis	4.200335129			-
d__Bacteria.p__Proteobacteria.c__Epsilonproteobacteria	0.684246748			-
d__Bacteria.p__Proteobacteria.c__Betaproteobacteria.o__Burkholderiales.f__Burkholderiaceae	2.901094895			-
d__Bacteria.p__Bacteroidetes.c__Bacteroidia.o__Bacteroidales.f__Rikenellaceae	1.560305243			-
d__Bacteria.p__Firmicutes.c__Negativicutes.o__Selenomonadales.f__Acidaminococcaceae.g__Phascolarctobacterium	2.243864489			-
d__Bacteria.p__Proteobacteria.c__Gammaproteobacteria.o__Oceanospirillales.f__Halomonadaceae	4.253434935			-
d__Bacteria.p__Proteobacteria.c__Gammaproteobacteria	5.891351398	M0	5.433890673	0.01040562
d__Bacteria.p__Actinobacteria.c__Actinobacteria.o__Coriobacteriales.f__Coriobacteriaceae.g__Gordonibacter	3.223366126			-
d__Bacteria.p__Proteobacteria.c__Betaproteobacteria.o__Neisseriales	2.000723222			-
d__Bacteria.p__Candidate_division_TM7.c__Candidate_division_TM7_norank	2.303196057			-
d__Bacteria.p__Proteobacteria.c__Alphaproteobacteria.o__Caulobacterales.f__Caulobacteraceae.g__Caulobacter	1.271066772			-
d__Bacteria.p__Firmicutes.c__Erysipelotrichia.o__Erysipelotrichales.f__Erysipelotrichaceae.g__Erysipelotrichaceae_Incertae_Sedis	3.681376933			-
d__Bacteria.p__Proteobacteria.c__Betaproteobacteria	3.388101202			-
d__Bacteria.p__Candidate_division_TM7.c__Candidate_division_TM7_norank.o__Candidate_division_TM7_norank	2.303196057			-
d__Bacteria.p__Fusobacteria.c__Fusobacteriia.o__Fusobacteriales.f__Fusobacteriaceae	3.758697466	M0	3.801063479	0.02222962
d__Bacteria.p__Actinobacteria.c__Actinobacteria.o__Micrococcales.f__Dermacoccaceae	0.684246748			-
d__Bacteria.p__Firmicutes.c__Bacilli.o__Lactobacillales.f__Enterococcaceae.g__Enterococcus	4.075370531			-
d__Bacteria.p__Actinobacteria.c__Actinobacteria.o__Micrococcales.f__Brevibacteriaceae	1.171238756			-
d__Bacteria.p__Firmicutes.c__Bacilli.o__Bacillales.f__Bacillales_Family_XI	2.693433804	M1	3.231397687	0.00280167
d__Bacteria.p__Proteobacteria.c__Gammaproteobacteria.o__Pseudomonadales.f__Moraxellaceae.g__Enhydrobacter	1.054357662			-
d__Bacteria.p__Firmicutes.c__Bacilli.o__Lactobacillales.f__Lactobacillales_unclassified	1.51851394			-
d__Bacteria.p__Firmicutes.c__Bacilli.o__Bacillales.f__Bacillaceae.g__Oceanobacillus	1.08517161			-
d__Bacteria.p__Fusobacteria.c__Fusobacteriia	3.758697466	M0	3.80187822	0.02222962
d__Bacteria.p__Firmicutes.c__Clostridia.o__Clostridiales.f__Lachnospiraceae.g__Anaerosporobacter	1.190331698			-
d__Bacteria.p__Firmicutes	5.441589887	M1	4.975889394	0.02497468
d__Bacteria.p__Actinobacteria.c__Actinobacteria.o__Bifidobacteriales.f__Bifidobacteriaceae.g__Parascardovia	1.539912085			-
d__Bacteria.p__Bacteroidetes.c__Bacteroidia.o__Bacteroidales.f__Prevotellaceae.g__Prevotella	3.002741858	M0	2.817174971	0.0131951
d__Bacteria.p__Actinobacteria.c__Actinobacteria.o__Actinomycetales	3.255071396	M1	2.990409845	0.03604871
d__Bacteria.p__Firmicutes.c__Clostridia.o__Clostridiales.f__Ruminococcaceae.g__Ruminococcaceae_Incertae_Sedis	0.985276743			-
d__Bacteria.p__Proteobacteria.c__Gammaproteobacteria.o__Oceanospirillales	4.253434935			-
d__Bacteria.p__Proteobacteria.c__Gammaproteobacteria.o__Xanthomonadales.f__Xanthomonadaceae.g__Xanthomonadaceae_uncultu	3.306853749			-
d__Bacteria.p__Actinobacteria.c__Actinobacteria.o__Micrococcales	4.219702383			-
d__Bacteria.p__Proteobacteria.c__Alphaproteobacteria.o__Rhodobacterales.f__Rhodobacteraceae.g__Rhodobacter	1.673635185			-
d__Bacteria.p__Bacteroidetes.c__Bacteroidia.o__Bacteroidales.f__Porphyromonadaceae.g__Parabacteroides	4.649365693			-
d__Bacteria.p__Firmicutes.c__Bacilli.o__Lactobacillales.f__Lactobacillaceae.g__Lactobacillus	3.193541989			-

Table S20. Detailed data for linear discriminant analysis (LDA) of the faecal microbial OTUs between pre-treatment (M0) and post-treatment (M1) (continued)

Biomaker_names	Logarithm value	Groups	LDA_value	P_value
d__Bacteria.p__Bacteroidetes.c__Bacteroidia.o__Bacteroidales.f__Prevotellaceae	3.022977937	M0	2.8391146	0.0131951
d__Bacteria.p__Firmicutes.c__Negativicutes.o__Selenomonadales.f__Veillonellaceae.g__Dialister	1.274927193			-
d__Bacteria.p__Actinobacteria.c__Actinobacteria.o__Coriobacteriales.f__Coriobacteriaceae.g__Eggerthella	2.89799343			-
d__Bacteria.p__Actinobacteria.c__Actinobacteria.o__Bifidobacteriales	5.285431278			-
d__Bacteria.p__Firmicutes.c__Bacilli.o__Lactobacillales.f__Aerococcaceae.g__Aerococcus	1.392110465			-
d__Bacteria.p__Firmicutes.c__Clostridia.o__Clostridiales.f__Clostridiales_Family_XI.g__Peptoniphilus	1.102662342			-
d__Bacteria.p__Bacteroidetes.c__Bacteroidia.o__Bacteroidales.f__S24_7.g__S24_7_norank	0.602059991			-
d__Bacteria.p__Actinobacteria.c__Actinobacteria.o__Micrococcales.f__Micrococcaceae.g__Rothia	4.219471005			-
d__Bacteria.p__Proteobacteria.c__Gammaproteobacteria.o__Enterobacteriales.f__Enterobacteriaceae.g__Morganella	1.199572355			-
d__Bacteria.p__Firmicutes.c__Clostridia.o__Clostridiales.f__Lachnospiraceae.g__Lachnoanaerobaculum	0.823908741			-
d__Bacteria.p__Proteobacteria.c__Gammaproteobacteria.o__Enterobacteriales.f__Enterobacteriaceae.g__Escherichia_Shigella	5.765239014			-
d__Bacteria.p__Proteobacteria.c__Betaproteobacteria.o__Burkholderiales	3.377640102			-
d__Bacteria.p__Firmicutes.c__Bacilli.o__Lactobacillales.f__Carnobacteriaceae	4.047450108			-
d__Bacteria.p__Proteobacteria.c__Betaproteobacteria.o__Burkholderiales.f__Oxalobacteraceae.g__Herbaspirillum	2			-
d__Bacteria.p__Actinobacteria.c__Actinobacteria.o__Corynebacteriales	2.732125594			-
d__Bacteria.p__Proteobacteria.c__Alphaproteobacteria.o__Caulobacterales.f__Hyphomonadaceae.g__Hyphomonadaceae_norank	2.945550644			-
d__Bacteria.p__Firmicutes.c__Erysipelotrichia.o__Erysipelotrichales.f__Erysipelotrichaceae	3.685278842			-
d__Bacteria.p__Bacteroidetes.c__Flavobacteriia	1.140926842			-
d__Bacteria.p__Firmicutes.c__Clostridia.o__Clostridiales.f__Ruminococcaceae.g__Flavonifractor	2.598790507			-
d__Bacteria.p__Verrucomicrobia.c__Verrucomicrobiae.o__Verrucomicrobiales	1.230448921			-
d__Bacteria.p__Proteobacteria.c__Gammaproteobacteria.o__Xanthomonadales	3.30763864			-
d__Bacteria.p__Firmicutes.c__Clostridia	4.383584997	M1	4.032429867	0.01040562
d__Bacteria.p__Actinobacteria.c__Actinobacteria.o__Pseudonocardiales.f__Pseudonocardiaceae	1.736396502			-
d__Bacteria.p__Actinobacteria.c__Actinobacteria.o__Pseudonocardiales.f__Pseudonocardiaceae.g__Saccharopolyspora	1.736396502			-
d__Bacteria.p__Bacteroidetes.c__Bacteroidia.o__Bacteroidales.f__Porphyromonadaceae	4.649677161			-
d__Bacteria.p__Proteobacteria.c__Betaproteobacteria.o__Rhodocyclales.f__Rhodocyclaceae.g__Methyloversatilis	1.06694679			-
d__Bacteria.p__Proteobacteria.c__Gammaproteobacteria.o__Pasteurellales.f__Pasteurellaceae.g__Actinobacillus	1.833572058			-
d__Bacteria.p__Bacteroidetes.c__Flavobacteriia.o__Flavobacteriales.f__Flavobacteriaceae.g__Chryseobacterium	1.140926842			-
d__Bacteria.p__Actinobacteria.c__Actinobacteria.o__Micrococcales.f__Microbacteriaceae	1.73239376			-
d__Bacteria.p__Proteobacteria.c__Gammaproteobacteria.o__Pasteurellales.f__Pasteurellaceae.g__Aggregatibacter	0.985276743			-
d__Bacteria.p__Proteobacteria.c__Gammaproteobacteria.o__Enterobacteriales.f__Enterobacteriaceae.g__Enterobacteriaceae_unclassifi	4.149891082			-
d__Bacteria.p__Firmicutes.c__Bacilli.o__Lactobacillales.f__Aerococcaceae	1.507406059	M0	3.908915329	0.04951017
d__Bacteria.p__Firmicutes.c__Bacilli.o__Lactobacillales.f__Enterococcaceae	4.075370531			-
d__Bacteria.p__Actinobacteria.c__Actinobacteria.o__Coriobacteriales.f__Coriobacteriaceae	4.266827286	M1	3.985277976	0.01040562
d__Bacteria.p__Proteobacteria.c__Gammaproteobacteria.o__Enterobacteriales.f__Enterobacteriaceae.g__Klebsiella	5.104501906			-
d__Bacteria.p__Actinobacteria.c__Actinobacteria.o__Micrococcales.f__Micrococcaceae.g__Arthrobacter	2.907590488	M0	3.114687168	0.00476268
d__Bacteria.p__Proteobacteria.c__Alphaproteobacteria.o__Rhodobacterales.f__Rhodobacteraceae	1.673635185			-

Table S20. Detailed data for linear discriminant analysis (LDA) of the faecal microbial OTUs between pre-treatment (M0) and post-treatment (M1) (continued)

Biomaker_names	Logarithm value	Groups	LDA_value	P_value
d__Bacteria.p__Proteobacteria.c__Alphaproteobacteria.o__Caulobacterales	2.96832726			-
d__Bacteria.p__Bacteroidetes.c__Bacteroidia.o__Bacteroidales.f__Porphyromonadaceae.g__Dysgonomonas	1.505149978			-
d__Bacteria.p__Proteobacteria.c__Betaproteobacteria.o__Rhodocyclales	1.722908012			-
d__Bacteria.p__Proteobacteria.c__Betaproteobacteria.o__Rhodocyclales.f__Rhodocyclaceae.g__Zoogloea	1.614545703			-
d__Bacteria.p__Firmicutes.c__Clostridia.o__Clostridiales.f__Clostridiaceae.g__Clostridium_sensu_stricto	3.703692521			-
d__Bacteria.p__Proteobacteria.c__Betaproteobacteria.o__Neisseriales.f__Neisseriaceae.g__Neisseria	2.000723222			-
d__Bacteria.p__Proteobacteria.c__Gammaproteobacteria.o__Enterobacteriales.f__Enterobacteriaceae.g__Serratia	2.573838205			-
d__Bacteria.p__Actinobacteria.c__Actinobacteria.o__Micrococcales.f__Dermabacteraceae	0.740362689			-
d__Bacteria.p__Actinobacteria.c__Actinobacteria.o__Coriobacteriales.f__Coriobacteriaceae.g__Atopobium	2.843025031			-
d__Bacteria.p__Proteobacteria.c__Betaproteobacteria.o__Neisseriales.f__Neisseriaceae	2.000723222			-
d__Bacteria.p__Firmicutes.c__Bacilli.o__Lactobacillales.f__Carnobacteriaceae.g__Granulicatella	4.04696965			-
d__Bacteria.p__Proteobacteria.c__Alphaproteobacteria.o__Sphingomonadales	2.89799343	M0	3.114157279	0.00739713
d__Bacteria.p__Firmicutes.c__Clostridia.o__Clostridiales.f__Lachnospiraceae.g__Lachnospira	1.936178509			-
d__Bacteria.p__Firmicutes.c__Bacilli.o__Bacillales.f__Staphylococcaceae.g__Staphylococcus	4.463422622			-
d__Bacteria.p__Verrucomicrobia.c__Verrucomicrobiae.o__Verrucomicrobiales.f__Verrucomicrobiaceae	1.230448921			-
d__Bacteria.p__Proteobacteria.c__Alphaproteobacteria.o__Rhizobiales.f__Phyllobacteriaceae	3.622956215			-
d__Bacteria.p__Actinobacteria.c__Actinobacteria.o__Actinomycetales.f__Actinomycetaceae.g__Actinomyces	3.255071396	M1	2.993731895	0.03604871
d__Bacteria.p__Proteobacteria.c__Epsilonproteobacteria.o__Campylobacterales.f__Campylobacteraceae.g__Campylobacter	0.684246748			-
d__Bacteria.p__Bacteroidetes.c__Bacteroidia.o__Bacteroidales	5.277995591			-
d__Bacteria.p__Firmicutes.c__Bacilli.o__Lactobacillales.f__Aerococcaceae.g__Abiotrophia	0.875061263			-
d__Bacteria.p__Firmicutes.c__Bacilli.o__Lactobacillales	5.315817861	M1	4.859333411	0.03737299
d__Bacteria.p__Firmicutes.c__Bacilli.o__Lactobacillales.f__Lactobacillaceae	3.193541989			-

Table S21. KEGG orthology functional terms identified by PICRUSt as different in pre-treatment (M0) and post-treatment (M1)

Biomaker_names	Logarithm value	Groups	LDA_value	P_value
L1_Metabolism.L2_Amino_Acid_Metabolism.L3_Valine__leucine_and_isoleucine_degradation	3.57571022			-
L1_Unclassified.L2_Cellular_Processes_and_Signaling.L3_Germination	1.53540825			0.0249747
L1_Unclassified.L2_Metabolism.L3_Others	4.02537543			-
L1_Human_Diseases.L2_Infectious_Diseases.L3_Tuberculosis	3.00410515			-
L1_Metabolism.L2_Amino_Acid_Metabolism.L3_Arginine_and_proline_metabolism	4.02565886			-
L1_Cellular_Processes.L2_Cell_Growth_and_Death.L3_Cell_cycle___Caulobacter	3.54023487			-
L1_Genetic_Information_Processing.L2_Translation.L3_Aminoacyl_tRNA_biosynthesis	3.9345699			-
L1_Metabolism.L2_Glycan_Biosynthesis_and_Metabolism.L3_Glycosphingolipid_biosynthesis___globo_series	2.89837302			-
L1_Metabolism.L2_Metabolism_of_Other_Amino_Acids.L3_D_Arginine_and_D_ornithine_metabolism	1.14968618			-
L1_Metabolism.L2_Enzyme_Families.L3_Protein_kinases	3.69475103			-
L1_Genetic_Information_Processing.L2_Transcription.L3_Basal_transcription_factors	0			-
L1_Metabolism.L2_Xenobiotics_Biodegradation_and_Metabolism.L3_Drug_metabolism___other_enzymes	3.42623017			-
L1_Metabolism.L2_Xenobiotics_Biodegradation_and_Metabolism.L3_Naphthalene_degradation	3.14211179			-
L1_Human_Diseases.L2_Metabolic_Diseases	2.97735512			-
L1_Metabolism.L2_Energy_Metabolism.L3_Sulfur_metabolism	3.51959927			-
L1_Environmental_Information_Processing.L2_Membrane_Transport.L3_Phosphotransferase_system__PTS_	4.06827967			-
L1_Metabolism.L2_Lipid_Metabolism.L3_Biosynthesis_of_unsaturated_fatty_acids	3.44144079			-
L1_Metabolism.L2_Amino_Acid_Metabolism.L3_Tyrosine_metabolism	3.62454914			-
L1_Cellular_Processes.L2_Cell_Growth_and_Death.L3_Apoptosis	1.71383213			-
L1_Cellular_Processes.L2_Cell_Motility.L3_Cytoskeleton_proteins	3.21638902			-
L1_Metabolism.L2_Biosynthesis_of_Other_Secondary_Metabolites	3.92145294			-
L1_Metabolism.L2_Metabolism_of_Terpenoids_and_Polyketides.L3_Biosynthesis_of_type_II_polyketide_products	0			-
L1_Organismal_Systems.L2_Environmental_Adaptation.L3_Plant_pathogen_interaction	3.03621103			-
L1_Metabolism.L2_Metabolism_of_Terpenoids_and_Polyketides.L3_Polyketide_sugar_unit_biosynthesis	3.12773606			-
L1_Metabolism.L2_Energy_Metabolism.L3_Nitrogen_metabolism	3.91920426			-
L1_Cellular_Processes	4.49356473			-
L1_Metabolism.L2_Glycan_Biosynthesis_and_Metabolism.L3_Lipopolysaccharide_biosynthesis_proteins	3.92914714	M0	3.14338008	0.0163092
L1_Metabolism.L2_Metabolism_of_Terpenoids_and_Polyketides.L3_Zeatin_biosynthesis	2.54594231			-
L1_Metabolism.L2_Metabolism_of_Other_Amino_Acids.L3_Taurine_and_hypotaurine_metabolism	3.08739485			-
L1_Genetic_Information_Processing.L2_Replication_and_Repair.L3_DNA_replication	3.71264864			-
L1_Human_Diseases.L2_Neurodegenerative_Diseases.L3_Parkinsons_disease	1.61711983			0.0161224
L1_Human_Diseases.L2_Neurodegenerative_Diseases.L3_Alzheimers_disease	2.72003195			-
L1_Metabolism.L2_Biosynthesis_of_Other_Secondary_Metabolites.L3_Penicillin_and_cephalosporin_biosynthesis	2.70899686			-
L1_Metabolism	5.66363255			-
L1_Human_Diseases.L2_Infectious_Diseases	3.78343813	M0	2.69431027	0.0249747
L1_Environmental_Information_Processing	5.27177272			-
L1_Metabolism.L2_Amino_Acid_Metabolism.L3_Lysine_degradation	3.48119436			-

Table S21. KEGG orthology functional terms identified by PICRUSt as different in pre-treatment (M0) and post-treatment (M1) (continued)

Biomaker_names	Logarithm value	Groups	LDA_value	P_value
L1_Metabolism.L2_Metabolism_of_Terpenoids_and_Polyketides	4.17879581			-
L1_Unclassified.L2_Poorly_Characterized.L3_Function_unknown	4.34090125			-
L1_Metabolism.L2_Lipid_Metabolism.L3_Steroid_hormone_biosynthesis	2.21920258			-
L1_Human_Diseases.L2_Cancers.L3_Colorectal_cancer	1.17447605			0.0091059
L1_Cellular_Processes.L2_Cell_Motility	4.40488356	M0	3.83753866	0.037373
L1_Metabolism.L2_Xenobiotics_Biodegradation_and_Metabolism.L3_Polycyclic_aromatic_hydrocarbon_degradation	2.87541918	M1	2.31692737	0.0163092
L1_Metabolism.L2_Enzyme_Families.L3_Cytochrome_P450	0			-
L1_Environmental_Information_Processing.L2_Signal_Transduction.L3_Calcium_signaling_pathway	0			-
L1_Human_Diseases.L2_Infectious_Diseases.L3_Chagas_disease__American_trypanosomiasis_	2.38185025			-
L1_Organismal_Systems.L2_Endocrine_System.L3_Melanogenesis	0	M0	2.15613943	0.0222296
L1_Human_Diseases.L2_Neurodegenerative_Diseases.L3_Prion_diseases	2.33520069			-
L1_Organismal_Systems.L2_Environmental_Adaptation.L3_Circadian_rhythm___plant	0			0.0463198
L1_Organismal_Systems.L2_Digestive_System.L3_Protein_digestion_and_absorption	2.2045919			-
L1_Organismal_Systems.L2_Immune_System.L3_RIG_I_like_receptor_signaling_pathway	1.7204487			-
L1_Metabolism.L2_Xenobiotics_Biodegradation_and_Metabolism	4.31804008			-
L1_Human_Diseases.L2_Infectious_Diseases.L3_Vibrio_cholerae_infection	0			-
L1_Metabolism.L2_Biosynthesis_of_Other_Secondary_Metabolites.L3_Tropane__piperidine_and_pyridine_alkaloid_biosynthesis	3.08817873			-
L1_Organismal_Systems.L2_Excretory_System	2.53326356			-
L1_Organismal_Systems.L2_Digestive_System.L3_Carbohydrate_digestion_and_absorption	2.42751008			-
L1_Human_Diseases.L2_Neurodegenerative_Diseases.L3_Amyotrophic_lateral_sclerosis__ALS_	2.69863529			-
L1_Environmental_Information_Processing.L2_Signaling_Molecules_and_Interaction.L3_Cellular_antigens	2.73499992			-
L1_Unclassified.L2_Cellular_Processes_and_Signaling.L3_Other_transporters	3.52422094			-
L1_Metabolism.L2_Enzyme_Families	4.32332225			-
L1_Human_Diseases.L2_Infectious_Diseases.L3_Staphylococcus_aureus_infection	2.51985184			-
L1_Metabolism.L2_Energy_Metabolism.L3_Photosynthesis_proteins	3.41420974			-
L1_Organismal_Systems.L2_Endocrine_System.L3_PPAR_signaling_pathway	2.92861582			-
L1_Metabolism.L2_Metabolism_of_Cofactors_and_Vitamins.L3_Thiamine_metabolism	3.61978305			-
L1_Human_Diseases.L2_Cancers.L3_Renal_cell_carcinoma	2.36551432			0.0163092
L1_Organismal_Systems.L2_Endocrine_System.L3_Insulin_signaling_pathway	2.8096776			-
L1_Human_Diseases.L2_Cancers.L3_Bladder_cancer	2.34535843			-
L1_Metabolism.L2_Energy_Metabolism.L3_Oxidative_phosphorylation	4.01496195			-
L1_Metabolism.L2_Metabolism_of_Terpenoids_and_Polyketides.L3_Limonene_and_pinene_degradation	3.26304016	M0	2.32881864	0.037373
L1_Organismal_Systems.L2_Endocrine_System	3.3485261			-
L1_Unclassified.L2_Genetic_Information_Processing	4.41432079			-
L1_Metabolism.L2_Carbohydrate_Metabolism.L3_Pentose_phosphate_pathway	3.91833641			-
L1_Metabolism.L2_Carbohydrate_Metabolism.L3_Ascorbate_and_aldarate_metabolism	3.50130572			-
L1_Genetic_Information_Processing.L2_Translation.L3_Ribosome_biogenesis_in_eukaryotes	2.71193405			-

Table S21. KEGG orthology functional terms identified by PICRUSt as different in pre-treatment (M0) and post-treatment (M1) (continued)

Biomaker_names	Logarithm value	Groups	LDA_value	P_value
L1_Metabolism.L2_Biosynthesis_of_Other_Secondary_Metabolites.L3_beta_Lactam_resistance	2.59896716			-
L1_Organismal_Systems.L2_Circulatory_System	1.42270596			0.0161224
L1_Metabolism.L2_Lipid_Metabolism.L3_Glycerophospholipid_metabolism	3.75987866	M0	2.55552162	0.037373
L1_Metabolism.L2_Amino_Acid_Metabolism	4.95029103			-
L1_Unclassified.L2_Poorly_Characterized.L3_General_function_prediction_only	4.53374476			-
L1_Organismal_Systems.L2_Excretory_System.L3_Proximal_tubule_bicarbonate_reclamation	2.53305472			-
L1_Metabolism.L2_Amino_Acid_Metabolism.L3_Cysteine_and_methionine_metabolism	3.97951073	M1	2.6596954	0.037373
L1_Environmental_Information_Processing.L2_Membrane_Transport.L3_Transporters	4.92238561			-
L1_Metabolism.L2_Biosynthesis_of_Other_Secondary_Metabolites.L3_Stilbenoid__diarylheptanoid_and_gingerol_biosynthesis	0.93695756			-
L1_Organismal_Systems	3.71548216			-
L1_Unclassified.L2_Metabolism.L3_Energy_metabolism	3.94931789			-
L1_Human_Diseases.L2_Metabolic_Diseases.L3_Type_II_diabetes_mellitus	2.69305521			-
L1_Metabolism.L2_Energy_Metabolism.L3_Photosynthesis___antenna_proteins	0			-
L1_Cellular_Processes.L2_Cell_Growth_and_Death.L3_p53_signaling_pathway	1.17508722			0.0091059
L1_Metabolism.L2_Carbohydrate_Metabolism.L3_Propanoate_metabolism	3.81867576			-
L1_Metabolism.L2_Metabolism_of_Cofactors_and_Vitamins.L3_Retinol_metabolism	2.78789348			-
L1_Unclassified.L2_Cellular_Processes_and_Signaling.L3_Inorganic_ion_transport_and_metabolism	3.6920569			-
L1_Metabolism.L2_Metabolism_of_Cofactors_and_Vitamins.L3_Biotin_metabolism	3.21113226			-
L1_Unclassified.L2_Cellular_Processes_and_Signaling.L3_Cell_division	2.92110969			-
L1_Unclassified.L2_Poorly_Characterized	4.74896995	M0	3.24466864	0.0249747
L1_Genetic_Information_Processing.L2_Folding__Sorting_and_Degradation.L3_RNA_degradation	3.59569704			-
L1_Metabolism.L2_Lipid_Metabolism.L3_Ether_lipid_metabolism	1.39003151			-
L1_Metabolism.L2_Amino_Acid_Metabolism.L3_Histidine_metabolism	3.65876364			-
L1_Unclassified.L2_Genetic_Information_Processing.L3_Translation_proteins	3.9354381			-
L1_Unclassified.L2_Genetic_Information_Processing.L3_Transcription_related_proteins	2.54877675			-
L1_Metabolism.L2_Metabolism_of_Terpenoids_and_Polyketides.L3_Carotenoid_biosynthesis	1.68604519			-
L1_Metabolism.L2_Energy_Metabolism.L3_Carbon_fixation_in_photosynthetic_organisms	3.76821869			-
L1_Genetic_Information_Processing.L2_Replication_and_Repair.L3_Non_homologous_end_joining	1.29933438			-
L1_Human_Diseases.L2_Cancers.L3_Pathways_in_cancer	2.69113675			-
L1_Genetic_Information_Processing.L2_Replication_and_Repair.L3_Mismatch_repair	3.77511011			-
L1_Metabolism.L2_Metabolism_of_Other_Amino_Acids.L3_D_Alanine_metabolism	3.00013201			-
L1_Metabolism.L2_Metabolism_of_Cofactors_and_Vitamins.L3_Nicotinate_and_nicotinamide_metabolism	3.66497103			-
L1_Metabolism.L2_Glycan_Biosynthesis_and_Metabolism.L3_Lipopolysaccharide_biosynthesis	3.72784407	M0	3.05308065	0.037373
L1_Cellular_Processes.L2_Transport_and_Catabolism.L3_Endocytosis	0			-
L1_Metabolism.L2_Metabolism_of_Terpenoids_and_Polyketides.L3_Tetracycline_biosynthesis	3.09948831			-
L1_Environmental_Information_Processing.L2_Membrane_Transport.L3_ABC_transporters	4.63640061			-
L1_Human_Diseases.L2_Immune_System_Diseases.L3_Systemic_lupus_erythematosus	0.21216819			-

Table S21. KEGG orthology functional terms identified by PICRUSt as different in pre-treatment (M0) and post-treatment (M1) (continued)

Biomaker_names	Logarithm value	Groups	LDA_value	P_value
L1_Organismal_Systems.L2_Endocrine_System.L3_Progesterone_mediated_oocyte_maturation	2.38757506			-
L1_Metabolism.L2_Xenobiotics_Biodegradation_and_Metabolism.L3_Benzoate_degradation	3.51259702			-
L1_Metabolism.L2_Lipid_Metabolism.L3_Glycerolipid_metabolism	3.58490114			-
L1_Metabolism.L2_Carbohydrate_Metabolism.L3_Fructose_and_mannose_metabolism	3.98109936			-
L1_Metabolism.L2_Glycan_Biosynthesis_and_Metabolism.L3_Glycosphingolipid_biosynthesis___lacto_and_neolacto_series	0			-
L1_Metabolism.L2_Amino_Acid_Metabolism.L3_Lysine_biosynthesis	3.7915464			-
L1_Metabolism.L2_Xenobiotics_Biodegradation_and_Metabolism.L3_Chloroalkane_and_chloroalkene_degradation	3.23795389			-
L1_Genetic_Information_Processing.L2_Folding__Sorting_and_Degradation.L3_Sulfur_relay_system	3.63700463			-
L1_Cellular_Processes.L2_Cell_Growth_and_Death	3.55284409			-
L1_Metabolism.L2_Metabolism_of_Cofactors_and_Vitamins.L3_Porphyrin_and_chlorophyll_metabolism	3.84002072			-
L1_Metabolism.L2_Energy_Metabolism.L3_Photosynthesis	3.39144309			-
L1_Metabolism.L2_Metabolism_of_Cofactors_and_Vitamins.L3_One_carbon_pool_by_folate	3.66270128			-
L1_Metabolism.L2_Xenobiotics_Biodegradation_and_Metabolism.L3_Aminobenzoate_degradation	3.27080248			-
L1_Human_Diseases.L2_Infectious_Diseases.L3_Influenza_A	1.17447605			0.0091059
L1_Metabolism.L2_Xenobiotics_Biodegradation_and_Metabolism.L3_Ethylbenzene_degradation	2.60912997			-
L1_Human_Diseases.L2_Infectious_Diseases.L3_Bacterial_invasion_of_epithelial_cells	2.17304153			-
L1_Metabolism.L2_Carbohydrate_Metabolism.L3_Pentose_and_glucuronate_interconversions	3.80084279			-
L1_Environmental_Information_Processing.L2_Signal_Transduction.L3_Two_component_system	4.37296824			-
L1_Metabolism.L2_Lipid_Metabolism.L3_Sphingolipid_metabolism	3.18482696			-
L1_Metabolism.L2_Xenobiotics_Biodegradation_and_Metabolism.L3_Drug_metabolism___cytochrome_P450	3.04431261			-
L1_Genetic_Information_Processing.L2_Translation.L3_Ribosome_Biogenesis	4.12120368			-
L1_Metabolism.L2_Metabolism_of_Terpenoids_and_Polyketides.L3_Biosynthesis_of_siderophore_group_nonribosomal_peptides	3.14011504			-
L1_Organismal_Systems.L2_Endocrine_System.L3_Adipocytokine_signaling_pathway	2.73784528			-
L1_Metabolism.L2_Lipid_Metabolism	4.46270271			-
L1_Metabolism.L2_Nucleotide_Metabolism.L3_Purine_metabolism	4.30684578			-
L1_Human_Diseases.L2_Metabolic_Diseases.L3_Type_I_diabetes_mellitus	2.65892465			-
L1_Human_Diseases.L2_Infectious_Diseases.L3_Epithelial_cell_signaling_in_Helicobacter_pylori_infection	2.83851818			-
L1_Metabolism.L2_Metabolism_of_Other_Amino_Acids	4.23556214			-
L1_Metabolism.L2_Xenobiotics_Biodegradation_and_Metabolism.L3_Fluorobenzoate_degradation	2.38945467			-
L1_Organismal_Systems.L2_Immune_System.L3_Antigen_processing_and_presentation	2.38757506			-
L1_Genetic_Information_Processing.L2_Folding__Sorting_and_Degradation.L3_Protein_export	3.67070658			-
L1_Genetic_Information_Processing.L2_Transcription	4.47726504			-
L1_Environmental_Information_Processing.L2_Signal_Transduction.L3_Phosphatidylinositol_signaling_system	3.01072575			-
L1_Genetic_Information_Processing.L2_Translation.L3_RNA_transport	3.06762792			-
L1_Metabolism.L2_Biosynthesis_of_Other_Secondary_Metabolites.L3_Streptomycin_biosynthesis	3.41082811			-
L1_Metabolism.L2_Lipid_Metabolism.L3_Secondary_bile_acid_biosynthesis	2.37880525			-
L1_Cellular_Processes.L2_Cell_Motility.L3_Bacterial_motility_proteins	4.13200211	M0	3.58482443	0.037373

Table S21. KEGG orthology functional terms identified by PICRUSt as different in pre-treatment (M0) and post-treatment (M1) (continued)

Biomaker_names	Logarithm value	Groups	LDA_value	P_value
L1_Organismal_Systems.L2_Circulatory_System.L3_Cardiac_muscle_contraction	1.42270596			0.0161224
L1_Human_Diseases.L2_Infectious_Diseases.L3_Toxoplasmosis	1.17447605			0.0091059
L1_Organismal_Systems.L2_Endocrine_System.L3_GnRH_signaling_pathway	0			-
L1_Human_Diseases.L2_Infectious_Diseases.L3_Pathogenic_Escherichia_coli_infection	0.28261795			0.0415959
L1_Metabolism.L2_Biosynthesis_of_Other_Secondary_Metabolites.L3_Butirosin_and_neomycin_biosynthesis	2.57309525			-
L1_Organismal_Systems.L2_Digestive_System.L3_Bile_secretion	1.42987302			-
L1_Environmental_Information_Processing.L2_Signaling_Molecules_and_Interaction.L3_Bacterial_toxins	2.93704128	M1	2.44450309	0.037373
L1_Genetic_Information_Processing	5.22190228			-
L1_Metabolism.L2_Amino_Acid_Metabolism.L3_Amino_acid_related_enzymes	4.08482213			-
L1_Cellular_Processes.L2_Transport_and_Catabolism	3.40431259			-
L1_Metabolism.L2_Biosynthesis_of_Other_Secondary_Metabolites.L3_Betalain_biosynthesis	0.67533536			0.0222296
L1_Metabolism.L2_Metabolism_of_Cofactors_and_Vitamins.L3_Ubiquinone_and_other_terpenoid_quinone_biosynthesis	3.66567076	M0	2.76445372	0.0163092
L1_Metabolism.L2_Amino_Acid_Metabolism.L3_Alanine__aspartate_and_glutamate_metabolism	3.93254866			-
L1_Cellular_Processes.L2_Transport_and_Catabolism.L3_Peroxisome	3.29075939			-
L1_Human_Diseases.L2_Immune_System_Diseases.L3_Primary_immunodeficiency	2.72802678			-
L1_Metabolism.L2_Lipid_Metabolism.L3_Steroid_biosynthesis	0			-
L1_Genetic_Information_Processing.L2_Translation.L3_Translation_factors	3.60391976			-
L1_Genetic_Information_Processing.L2_Translation	4.64034959			-
L1_Metabolism.L2_Biosynthesis_of_Other_Secondary_Metabolites.L3_Flavonoid_biosynthesis	0.79120399			-
L1_Metabolism.L2_Metabolism_of_Terpenoids_and_Polyketides.L3_Biosynthesis_of_vancomycin_group_antibiotics	2.61514572			-
L1_Metabolism.L2_Glycan_Biosynthesis_and_Metabolism.L3_Peptidoglycan_biosynthesis	3.83343601			-
L1_Unclassified.L2_Cellular_Processes_and_Signaling.L3_Signal_transduction_mechanisms	3.74398577			-
L1_Metabolism.L2_Xenobiotics_Biodegradation_and_Metabolism.L3_Chlorocyclohexane_and_chlorobenzene_degradation	2.34337362			-
L1_Environmental_Information_Processing.L2_Membrane_Transport.L3_Bacterial_secretion_system	3.97328603			-
L1_Genetic_Information_Processing.L2_Folding__Sorting_and_Degradation	4.38054431			-
L1_Genetic_Information_Processing.L2_Replication_and_Repair.L3_DNA_replication_proteins	3.97530622			-
L1_Metabolism.L2_Carbohydrate_Metabolism.L3_Starch_and_sucrose_metabolism	3.99909755	M1	3.20875789	0.0249747
L1_Genetic_Information_Processing.L2_Replication_and_Repair.L3_Base_excision_repair	3.5601192			-
L1_Metabolism.L2_Xenobiotics_Biodegradation_and_Metabolism.L3_Metabolism_of_xenobiotics_by_cytochrome_P450	3.04985601			-
L1_Metabolism.L2_Carbohydrate_Metabolism.L3_Amino_sugar_and_nucleotide_sugar_metabolism	4.13777525			-
L1_Metabolism.L2_Lipid_Metabolism.L3_Lipid_biosynthesis_proteins	3.75415955			-
L1_Cellular_Processes.L2_Cell_Growth_and_Death.L3_Meiosis___yeast	1.7027242			-
L1_Metabolism.L2_Glycan_Biosynthesis_and_Metabolism.L3_Various_types_of_N_glycan_biosynthesis	0			-
L1_Unclassified.L2_Cellular_Processes_and_Signaling.L3_Cell_motility_and_secretion	3.41713965	M0	2.60201634	0.0249747
L1_Metabolism.L2_Amino_Acid_Metabolism.L3_Tryptophan_metabolism	3.57122762	M0	2.56535397	0.037373
L1_Human_Diseases.L2_Immune_System_Diseases	2.72805199			-
L1_Unclassified.L2_Cellular_Processes_and_Signaling.L3_Other_ion_coupled_transporters	4.22546292			-

Table S21. KEGG orthology functional terms identified by PICRUSt as different in pre-treatment (M0) and post-treatment (M1) (continued)

Biomaker_names	Logarithm value	Groups	LDA_value	P_value
L1_Environmental_Information_Processing.L2_Membrane_Transport	5.21633578			-
L1_Metabolism.L2_Lipid_Metabolism.L3_alpha_Linolenic_acid_metabolism	2.72154996			0.037373
L1_Genetic_Information_Processing.L2_Replication_and_Repair.L3_DNA_repair_and_recombination_proteins	4.37261397			-
L1_Genetic_Information_Processing.L2_Folding__Sorting_and_Degradation.L3_Proteasome	2.51123464			-
L1_Unclassified.L2_Cellular_Processes_and_Signaling	4.71497121	M0	3.49131607	0.037373
L1_Human_Diseases.L2_Infectious_Diseases.L3_African_trypanosomiasis	2.38251562			-
L1_Unclassified.L2_Cellular_Processes_and_Signaling.L3_Pores_ion_channels	3.84495954			-
L1_Metabolism.L2_Carbohydrate_Metabolism.L3_Inositol_phosphate_metabolism	3.28966239			-
L1_Metabolism.L2_Lipid_Metabolism.L3_Fatty_acid_metabolism	3.64960496			-
L1_Metabolism.L2_Metabolism_of_Cofactors_and_Vitamins.L3_Vitamin_B6_metabolism	3.34285698			-
L1_Organismal_Systems.L2_Digestive_System.L3_Mineral_absorption	1.37138465			-
L1_Human_Diseases.L2_Neurodegenerative_Diseases.L3_Huntingtons_disease	2.85847599	M0	2.09123858	0.037373
L1_Metabolism.L2_Carbohydrate_Metabolism.L3_Butanoate_metabolism	3.93011333			-
L1_Metabolism.L2_Glycan_Biosynthesis_and_Metabolism.L3_N_Glycan_biosynthesis	2.02011114			-
L1_Genetic_Information_Processing.L2_Replication_and_Repair	4.85076886			-
L1_Metabolism.L2_Amino_Acid_Metabolism.L3_Phenylalanine_metabolism	3.40054159			-
L1_Unclassified.L2_Metabolism.L3_Biosynthesis_and_biodegradation_of_secondary_metabolites	3.18237417			-
L1_Organismal_Systems.L2_Endocrine_System.L3_Renin_angiotensin_system	0	M0	2.02109968	0.0326303
L1_Unclassified.L2_Metabolism.L3_Glycan_biosynthesis_and_metabolism	3.14759523			-
L1_Metabolism.L2_Xenobiotics_Biodegradation_and_Metabolism.L3_Atrazine_degradation	2.62167932			-
L1_Unclassified.L2_Metabolism.L3_Nucleotide_metabolism	3.14285391			-
L1_Organismal_Systems.L2_Immune_System.L3_NOD_like_receptor_signaling_pathway	2.40918645			-
L1_Human_Diseases.L2_Cancers	3.08758595			-
L1_Metabolism.L2_Carbohydrate_Metabolism	5.05338941			-
L1_Metabolism.L2_Lipid_Metabolism.L3_Synthesis_and_degradation_of_ketone_bodies	2.68509998			-
L1_Metabolism.L2_Nucleotide_Metabolism.L3_Pyrimidine_metabolism	4.16482663			-
L1_Metabolism.L2_Metabolism_of_Cofactors_and_Vitamins.L3_Pantothenate_and_CoA_biosynthesis	3.73107232			-
L1_Unclassified.L2_Cellular_Processes_and_Signaling.L3_Electron_transfer_carriers	3.05639535			-
L1_Metabolism.L2_Metabolism_of_Terpenoids_and_Polyketides.L3_Geraniol_degradation	3.30068319	M0	2.50732393	0.0249747
L1_Unclassified.L2_Cellular_Processes_and_Signaling.L3_Sporulation	2.87852674	M1	2.47890951	0.0163092
L1_Metabolism.L2_Energy_Metabolism	4.70727002			-
L1_Metabolism.L2_Carbohydrate_Metabolism.L3_Glyoxylate_and_dicarboxylate_metabolism	3.81842681	M0	2.5780753	0.037373
L1_Genetic_Information_Processing.L2_Folding__Sorting_and_Degradation.L3_Chaperones_and_folding_catalysts	4.01312141			-
L1_Metabolism.L2_Carbohydrate_Metabolism.L3_Citrate_cycle__TCA_cycle_	3.8695854	M0	2.57889913	0.037373
L1_Genetic_Information_Processing.L2_Transcription.L3_RNA_polymerase	3.10816267			-
L1_Organismal_Systems.L2_Immune_System	2.72225593			-
L1_Metabolism.L2_Energy_Metabolism.L3_Carbon_fixation_pathways_in_prokaryotes	3.98880759			-

Table S21. KEGG orthology functional terms identified by PICRUSt as different in pre-treatment (M0) and post-treatment (M1) (continued)

Biomaker_names	Logarithm value	Groups	LDA_value	P_value
L1_Metabolism.L2_Metabolism_of_Terpenoids_and_Polyketides.L3_Prenyltransferases	3.37698452			-
L1_Human_Diseases.L2_Infectious_Diseases.L3_Shigellosis	0	M0	2.14357613	0.0247217
L1_Environmental_Information_Processing.L2_Signaling_Molecules_and_Interaction	3.26649081			-
L1_Metabolism.L2_Biosynthesis_of_Other_Secondary_Metabolites.L3_Caffeine_metabolism	0.48084445			0.0131951
L1_Metabolism.L2_Lipid_Metabolism.L3_Linoleic_acid_metabolism	2.32132986			-
L1_Human_Diseases.L2_Neurodegenerative_Diseases	3.28611479			-
L1_Environmental_Information_Processing.L2_Signal_Transduction.L3_MAPK_signaling_pathway___yeast	2.65193599			-
L1_Environmental_Information_Processing.L2_Signal_Transduction	4.3980066			-
L1_Metabolism.L2_Glycan_Biosynthesis_and_Metabolism.L3_Glycosphingolipid_biosynthesis___ganglio_series	2.49801068			-
L1_Genetic_Information_Processing.L2_Transcription.L3_Transcription_machinery	3.75057297			-
L1_Metabolism.L2_Carbohydrate_Metabolism.L3_Galactose_metabolism	3.8969998	M1	3.1218467	0.037373
L1_Human_Diseases.L2_Cancers.L3_Small_cell_lung_cancer	1.17447605			0.0091059
L1_Metabolism.L2_Glycan_Biosynthesis_and_Metabolism.L3_Glycosyltransferases	3.68468295			-
L1_Genetic_Information_Processing.L2_Replication_and_Repair.L3_Chromosome	4.14759604			-
L1_Genetic_Information_Processing.L2_Folding__Sorting_and_Degradation.L3_Protein_processing_in_endoplasmic_reticulum	2.51533362			-
L1_Metabolism.L2_Xenobiotics_Biodegradation_and_Metabolism.L3_Bisphenol_degradation	2.50150318			-
L1_Environmental_Information_Processing.L2_Signaling_Molecules_and_Interaction.L3_Ion_channels	2.64226796			-
L1_Human_Diseases.L2_Infectious_Diseases.L3_Pertussis	3.39734194			-
L1_Unclassified.L2_Cellular_Processes_and_Signaling.L3_Membrane_and_intracellular_structural_molecules	3.979683	M0	2.96429792	0.037373
L1_Genetic_Information_Processing.L2_Transcription.L3_Transcription_factors	4.36352703			-
L1_Environmental_Information_Processing.L2_Membrane_Transport.L3_Secretion_system	4.34714085	M0	3.38648608	0.037373
L1_Metabolism.L2_Biosynthesis_of_Other_Secondary_Metabolites.L3_Flavone_and_flavonol_biosynthesis	1.75646277			-
L1_Organismal_Systems.L2_Environmental_Adaptation	3.0364643			-
L1_Genetic_Information_Processing.L2_Replication_and_Repair.L3_Nucleotide_excision_repair	3.42868533			-
L1_Metabolism.L2_Nucleotide_Metabolism	4.54264575			-
L1_Unclassified.L2_Metabolism.L3_Metabolism_of_cofactors_and_vitamins	3.34001504	M0	2.32328807	0.037373
L1_Metabolism.L2_Metabolism_of_Other_Amino_Acids.L3_Selenocompound_metabolism	3.60276084			-
L1_Metabolism.L2_Metabolism_of_Cofactors_and_Vitamins	4.62152096			-
L1_Unclassified.L2_Genetic_Information_Processing.L3_Replication__recombination_and_repair_proteins	3.91216848			-
L1_Metabolism.L2_Metabolism_of_Other_Amino_Acids.L3_Phosphonate_and_phosphinate_metabolism	2.8904965			-
L1_Metabolism.L2_Lipid_Metabolism.L3_Primary_bile_acid_biosynthesis	2.3862239			-
L1_Metabolism.L2_Biosynthesis_of_Other_Secondary_Metabolites.L3_Indole_alkaloid_biosynthesis	0.64919594			-
L1_Metabolism.L2_Metabolism_of_Terpenoids_and_Polyketides.L3_Biosynthesis_of_ansamycins	3.06593651			-
L1_Metabolism.L2_Metabolism_of_Other_Amino_Acids.L3_D_Glutamine_and_D_glutamate_metabolism	3.10337401			-
L1_Human_Diseases.L2_Infectious_Diseases.L3_Vibrio_cholerae_pathogenic_cycle	3.12174242	M0	2.35023026	0.0163092
L1_Metabolism.L2_Xenobiotics_Biodegradation_and_Metabolism.L3_Toluene_degradation	3.31986306	M0	2.38544219	0.037373
L1_Human_Diseases.L2_Cardiovascular_Diseases.L3_Hypertrophic_cardiomyopathy__HCM	0			-

Table S21. KEGG orthology functional terms identified by PICRUSt as different in pre-treatment (M0) and post-treatment (M1) (continued)

Biomaker_names	Logarithm value	Groups	LDA_value	P_value
L1_Metabolism.L2_Metabolism_of_Cofactors_and_Vitamins.L3_Lipoic_acid_metabolism	2.87151522			-
L1_Metabolism.L2_Glycan_Biosynthesis_and_Metabolism.L3_Other_glycan_degradation	3.31485476			-
L1_Metabolism.L2_Glycan_Biosynthesis_and_Metabolism.L3_Glycosaminoglycan_degradation	2.66551721			-
L1_Genetic_Information_Processing.L2_Folding__Sorting_and_Degradation.L3_Ubiquitin_system	2.48589975			-
L1_Metabolism.L2_Lipid_Metabolism.L3_Fatty_acid_elongation_in_mitochondria	0			-
L1_Metabolism.L2_Energy_Metabolism.L3_Methane_metabolism	3.99353525			-
L1_Organismal_Systems.L2_Nervous_System.L3_Glutamatergic_synapse	2.86627231			-
L1_Human_Diseases.L2_Cardiovascular_Diseases.L3_Viral_myocarditis	1.17447605			0.0091059
L1_Cellular_Processes.L2_Transport_and_Catabolism.L3_Lysosome	2.85302189			-
L1_Human_Diseases.L2_Cancers.L3_Prostate_cancer	2.39618514			-
L1_Metabolism.L2_Xenobiotics_Biodegradation_and_Metabolism.L3_Dioxin_degradation	3.05898444			-
L1_Metabolism.L2_Carbohydrate_Metabolism.L3_Glycolysis___Gluconeogenesis	4.06799611	M1	2.92424484	0.0249747
L1_Metabolism.L2_Metabolism_of_Cofactors_and_Vitamins.L3_Riboflavin_metabolism	3.45888088			-
L1_Organismal_Systems.L2_Excretory_System.L3_Vasopressin_regulated_water_reabsorption	0			-
L1_Metabolism.L2_Xenobiotics_Biodegradation_and_Metabolism.L3_Xylene_degradation	2.92308343			-
L1_Metabolism.L2_Carbohydrate_Metabolism.L3_C5_Branched_dibasic_acid_metabolism	3.50826097			-
L1_Unclassified.L2_Metabolism.L3_Carbohydrate_metabolism	3.34737516			-
L1_Metabolism.L2_Carbohydrate_Metabolism.L3_Pyruvate_metabolism	4.04117896			-
L1_Genetic_Information_Processing.L2_Translation.L3_Ribosome	4.23194255			-
L1_Metabolism.L2_Lipid_Metabolism.L3_Arachidonic_acid_metabolism	2.73351624			-
L1_Metabolism.L2_Amino_Acid_Metabolism.L3_Glycine__serine_and_threonine_metabolism	3.9360798			-
L1_Metabolism.L2_Amino_Acid_Metabolism.L3_Phenylalanine__tyrosine_and_tryptophan_biosynthesis	3.83786711			-
L1_Organismal_Systems.L2_Nervous_System	2.86627231			-
L1_Metabolism.L2_Lipid_Metabolism.L3_Fatty_acid_biosynthesis	3.63787994			-
L1_Metabolism.L2_Metabolism_of_Cofactors_and_Vitamins.L3_Folate_biosynthesis	3.63026216			-
L1_Metabolism.L2_Biosynthesis_of_Other_Secondary_Metabolites.L3_Phenylpropanoid_biosynthesis	3.19573525	M1	2.63571263	0.0163092
L1_Metabolism.L2_Xenobiotics_Biodegradation_and_Metabolism.L3_Nitrotoluene_degradation	2.99602293			-
L1_Metabolism.L2_Biosynthesis_of_Other_Secondary_Metabolites.L3_Isoquinoline_alkaloid_biosynthesis	2.80219431			-
L1_Metabolism.L2_Xenobiotics_Biodegradation_and_Metabolism.L3_Styrene_degradation	2.60889624			-
L1_Metabolism.L2_Glycan_Biosynthesis_and_Metabolism	4.42502044			-
L1_Human_Diseases	4.02969425	M0	2.98932616	0.0163092
L1_Metabolism.L2_Xenobiotics_Biodegradation_and_Metabolism.L3_1_1_1_Trichloro_2_2_bis_4_chlorophenyl_ethane__DDT__degradation	0			-
L1_Organismal_Systems.L2_Digestive_System	2.6796217			-
L1_Unclassified.L2_Metabolism	4.48447123			-
L1_Genetic_Information_Processing.L2_Replication_and_Repair.L3_Homologous_recombination	3.86411038			-
L1_Organismal_Systems.L2_Immune_System.L3_Fc_gamma_R_mediated_phagocytosis	0			-
L1_Cellular_Processes.L2_Cell_Motility.L3_Bacterial_chemotaxis	3.59869975	M0	2.92041044	0.037373

Table S21. KEGG orthology functional terms identified by PICRUSt as different in pre-treatment (M0) and post-treatment (M1) (continued)

Biomaker_names	Logarithm value	Groups	LDA_value	P_value
L1_Metabolism.L2_Metabolism_of_Other_Amino_Acids.L3_beta_Alanine_metabolism	3.49660234			-
L1_Unclassified.L2_Genetic_Information_Processing.L3_Protein_folding_and_associated_processing	3.88643792			-
L1_Metabolism.L2_Amino_Acid_Metabolism.L3_Valine__leucine_and_isoleucine_biosynthesis	3.82521129			-
L1_Metabolism.L2_Metabolism_of_Other_Amino_Acids.L3_Glutathione_metabolism	3.63038352			-
L1_Metabolism.L2_Biosynthesis_of_Other_Secondary_Metabolites.L3_Novobiocin_biosynthesis	3.12139571			-
L1_Metabolism.L2_Enzyme_Families.L3_Peptidases	4.22154381			-
L1_Unclassified.L2_Genetic_Information_Processing.L3_Restriction_enzyme	3.14058164			-
L1_Unclassified.L2_Metabolism.L3_Lipid_metabolism	3.20702564			-
L1_Cellular_Processes.L2_Cell_Motility.L3_Flagellar_assembly	3.81482544	M0	3.37158246	0.0249747
L1_Unclassified	5.21436551			-
L1_Metabolism.L2_Xenobiotics_Biodegradation_and_Metabolism.L3_Caprolactam_degradation	3.21156744	M0	2.44201414	0.037373
L1_Human_Diseases.L2_Cardiovascular_Diseases	1.17577901			0.0144833
L1_Metabolism.L2_Metabolism_of_Other_Amino_Acids.L3_Cyanoamino_acid_metabolism	3.43322003	M1	2.62913627	0.0249747
L1_Metabolism.L2_Metabolism_of_Terpenoids_and_Polyketides.L3_Terpenoid_backbone_biosynthesis	3.60499931			-
L1_Unclassified.L2_Metabolism.L3_Amino_acid_metabolism	3.43581379			-
L1_Human_Diseases.L2_Infectious_Diseases.L3_Amoebiasis	1.71057621			-
L1_Metabolism.L2_Biosynthesis_of_Other_Secondary_Metabolites.L3_Isoflavonoid_biosynthesis	0			-

Table S22. Weighted UniFrac distance of each sample between recovered NJI (neonatal jaundice infants) and non-NJI at 0, 1, 3, 6 and 12 months

sample ID	Standard deviation	Proportion of Variance	Cumulative Proportion
PC1	1.358309784	0.4864	0.4864
PC2	1.038377251	0.28425	0.77065
PC3	0.660680416	0.11507	0.88573
PC4	0.504214533	0.06702	0.95275
PC5	0.186741928	0.00919	0.96194
PC6	0.169008829	0.00753	0.96947
PC7	0.122794785	0.00398	0.97345
PC8	0.110458681	0.00322	0.97666
PC9	0.100477413	0.00266	0.97933
PC10	0.095959157	0.00243	0.98175
PC11	0.093198147	0.00229	0.98404
PC12	0.08215332	0.00178	0.98582
PC13	0.076729405	0.00155	0.98738
PC14	0.072920898	0.0014	0.98878
PC15	0.067207843	0.00119	0.98997
PC16	0.060905015	0.00098	0.99095
PC17	0.056202819	0.00083	0.99178
PC18	0.053695531	0.00076	0.99254
PC19	0.051096938	0.00069	0.99323
PC20	0.048715409	0.00063	0.99385
PC21	0.041927075	0.00046	0.99432
PC22	0.040022206	0.00042	0.99474
PC23	0.038222779	0.00039	0.99512
PC24	0.035590716	0.00033	0.99546
PC25	0.033540121	3.00E-04	0.99575
PC26	0.032112065	0.00027	0.99603
PC27	0.031487229	0.00026	0.99629
PC28	0.029418492	0.00023	0.99652
PC29	0.028030896	0.00021	0.99672
PC30	0.027335556	2.00E-04	0.99692
PC31	0.026408138	0.00018	0.9971
PC32	0.025981214	0.00018	0.99728
PC33	0.024631661	0.00016	0.99744
PC34	0.023621589	0.00015	0.99759
PC35	0.022237987	0.00013	0.99772
PC36	0.021249736	0.00012	0.99784
PC37	0.020718558	0.00011	0.99795
PC38	0.02040899	0.00011	0.99806
PC39	0.019580838	1.00E-04	0.99816
PC40	0.019351041	1.00E-04	0.99826
PC41	0.018792138	9.00E-05	0.99835
PC42	0.017688848	8.00E-05	0.99844
PC43	0.017370997	8.00E-05	0.99852
PC44	0.016989788	8.00E-05	0.99859
PC45	0.016525082	7.00E-05	0.99866
PC46	0.015972242	7.00E-05	0.99873
PC47	0.015689983	6.00E-05	0.9988
PC48	0.015174854	6.00E-05	0.99886
PC49	0.01459474	6.00E-05	0.99891
PC50	0.014440298	5.00E-05	0.99897
PC51	0.014245349	5.00E-05	0.99902
PC52	0.013783766	5.00E-05	0.99907
PC53	0.013731107	5.00E-05	0.99912
PC54	0.013262928	5.00E-05	0.99917
PC55	0.012963702	4.00E-05	0.99921
PC56	0.012454967	4.00E-05	0.99925
PC57	0.012317892	4.00E-05	0.99929
PC58	0.012102691	4.00E-05	0.99933
PC59	0.011584044	4.00E-05	0.99937

Table S22. Weighted UniFrac distance of each sample between recovered NJI (neonatal jaundice infants) and non-NJI at 0, 1, 3, 6 and 12 months (continued)

sample ID	Standard deviation	Proportion of Variance	Cumulative Proportion
PC60	0.011374192	3.00E-05	0.9994
PC61	0.011338297	3.00E-05	0.99943
PC62	0.010687759	3.00E-05	0.99946
PC63	0.010329878	3.00E-05	0.99949
PC64	0.009975763	3.00E-05	0.99952
PC65	0.009800469	3.00E-05	0.99954
PC66	0.009629886	2.00E-05	0.99957
PC67	0.009432389	2.00E-05	0.99959
PC68	0.009305907	2.00E-05	0.99962
PC69	0.009087447	2.00E-05	0.99964
PC70	0.008793826	2.00E-05	0.99966
PC71	0.008610134	2.00E-05	0.99968
PC72	0.008547925	2.00E-05	0.9997
PC73	0.008242653	2.00E-05	0.99971
PC74	0.00811506	2.00E-05	0.99973
PC75	0.007768974	2.00E-05	0.99975
PC76	0.00767749	2.00E-05	0.99976
PC77	0.007426317	1.00E-05	0.99978
PC78	0.007354451	1.00E-05	0.99979
PC79	0.007261485	1.00E-05	0.99981
PC80	0.00705771	1.00E-05	0.99982
PC81	0.006827832	1.00E-05	0.99983
PC82	0.006670665	1.00E-05	0.99984
PC83	0.006432695	1.00E-05	0.99985
PC84	0.00622907	1.00E-05	0.99986
PC85	0.00610566	1.00E-05	0.99987
PC86	0.005940944	1.00E-05	0.99988
PC87	0.005845498	1.00E-05	0.99989
PC88	0.005747212	1.00E-05	0.9999
PC89	0.005652645	1.00E-05	0.99991
PC90	0.00547475	1.00E-05	0.99992
PC91	0.005285811	1.00E-05	0.99992
PC92	0.005029531	1.00E-05	0.99993
PC93	0.004952246	1.00E-05	0.99994
PC94	0.004862534	1.00E-05	0.99994
PC95	0.004634178	1.00E-05	0.99995
PC96	0.00455159	1.00E-05	0.99995
PC97	0.00430706	0	0.99996
PC98	0.004292614	0	0.99996
PC99	0.004075283	0	0.99997
PC100	0.003971458	0	0.99997
PC101	0.003944643	0	0.99998
PC102	0.003849909	0	0.99998
PC103	0.003530261	0	0.99998
PC104	0.00336977	0	0.99999
PC105	0.00307883	0	0.99999
PC106	0.002967768	0	0.99999
PC107	0.002778007	0	0.99999
PC108	0.002504845	0	1
PC109	0.002336946	0	1
PC110	0.002131515	0	1
PC111	0.00176672	0	1
PC112	0.001368393	0	1
PC113	0.000883248	0	1
PC114	6.26E-17	0	1

Table S23. The identified key OTUs of a heatmap between recovered NJI (neonatal jaundice infants) and non-NJI at 1 month

OTU	AI023_2	AI019_2	BI006_4	BI008_2	BI013_3	BI021_2	CI008_2	CI011_3	CI015_2	CI016_2	CI021_2	DI018_2	DI024_2	AI020_3	BI020_2	CI006_4	CI009_2	CI013_3	CI017_2
OTU300 (Megasphaera)	7.55E-05	0.00015	0.0013	5.85E-05	0	0.00011	0	0.00225	0	6.52E-05	0	0	0.00931	0	0	0	0	0	0
OTU178 (Klebsiella)	0.90523	0.00586	0.44193	0.60241	0.71889	0.86787	0.30359	0.98103	0.0071	0.1456	0.02142	0.86542	0.92379	0.74754	0.92826	0.87938	0.69556	0.62216	0.79939
OTU331 (Faecalibacterium)	0.00057	0.00019	0	0	0.00081	0.00011	0.0001	0.00056	0.00133	0	9.80E-05	0.00024	0.00019	0	0	0.00027	0	0.00016	0.00032
OTU342 (Lachnospiraceae_Incertae_Sedis)	0	3.94E-05	0.00358	0	0	0	0	0	0	6.91E-05	0	0	0.00021	0.0241	0.003	0	0.28101	0.07667	0
OTU65 (Corynebacterium)	9.44E-05	6.06E-05	0	6.05E-05	0	0	0	0	0	4.94E-05	9.50E-05	0.00013	0.00019	0.00117	0.00019	0.00065	0	0.00037	0
OTU325 (Candidate_division_TM7_norank)	0	0	0	0	0.00038	0	0	8.88E-05	0	0.00078	0	0	0	0.01262	0.00024	0.00071	0	0.00034	0
OTU396 (Serratia)	0.00035	6.62E-05	0.06438	0.00038	0.00255	0.00038	0.00061	0.00016	0	0.00068	0.00055	0.00044	0.00062	0.00239	0.004	0.00384	0	0.00129	0
OTU153 (Veillonella)	0.01034	0.19503	0.08825	0.10208	0.19736	0.08794	0.04863	0.00678	0.50688	0.61165	0.63107	0.12408	0.00397	0.02947	0.01824	0.00597	0.02183	0.29673	0.00187
OTU53 (Prevotella)	0.00018	0.00043	0	5.85E-05	0.00033	5.03E-05	7.68E-05	0	0.0151	0	0.0002	0	0	0	0	0	0	0	0
OTU164 (Scardovia)	0.00053	0.00561	0.00478	0.00022	0	3.62E-05	0.00033	0.00017	0.00365	0	0.01414	0	0	0.00532	0	0	0	0	0
OTU279 (Streptococcus)	0.02372	0.01072	0.28955	0.08587	0.07244	0.03388	0.61878	0.0087	0.46594	0.02146	0.078	0.00368	0.06021	0.13332	0.03656	0.10866	0.0016	0.00213	0.19682
OTU185 (Haemophilus)	0.05838	0.78176	0.09913	0.20841	0.0043	0.00942	0.02787	0.00017	0	0.21931	0.25399	0.00541	0.0013	0.04353	0.00928	0.00052	0	0.00016	0.00103
OTU198 (Actinomyces)	0.00051	9.58E-05	0.0071	0.00046	0.00294	0.0002	0	8.88E-05	0	0.00033	0.00045	0.0006	0.00022	0.00054	0.00023	0	0	0	0.00056

Table S24. Faecal bacterial composition in each sample at the phylum level between recovered NJI (neonatal jaundice infants) and non-NJI at 1 month

phylum	AI023_2	AI019_2	BI006_4	BI008_2	BI013_3	BI021_2	CI008_2	CI011_3	CI015_2	CI016_2	CI021_2	DI018_2	DI024_2	AI020_3	BI020_2	CI006_4	CI009_2	CI013_3	CI017_2
Actinobacteria	0.02051	0.04243	0.64126	0.05475	0.02531	0.03101	0.18708	0.03439	0.11063	0.00998	0.28903	0.12613	0.20581	0.24912	0.52608	0.29672	0.21893	0.05942	0.09698
Bacteroidetes	0.00264	0.00268	0.00312	0.00417	0.01352	0.03351	0.22051	0.00856	0.00095	7.90E-05	0.00088	0.37822	0.02906	0.53125	0.0264	0.00058	0.01676	0.56281	0.00026
Candidate_division_TM7	0	0	0	0	2.30E-05	0	0	2.70E-05	0	0.0004	0	0	0	0.00102	3.70E-05	8.90E-05	0	5.60E-05	0
Cyanobacteria	0	0	0	0	0	0	0	0	0	0.00034	8.60E-05	0	0	0	0	0	0	3.20E-05	0
Firmicutes	0.67577	0.38726	0.28707	0.45335	0.69903	0.28711	0.39864	0.59433	0.76951	0.60586	0.34215	0.07783	0.12189	0.11469	0.12635	0.46288	0.73987	0.11353	0.10127
Fusobacteria	0	0	0	2.60E-05	0	0	0	2.00E-05	0	0	0.00024	0	0	0	0	0	0	0	0
Proteobacteria	0.30108	0.56764	0.06855	0.48771	0.26194	0.64836	0.19377	0.36268	0.11891	0.38326	0.36762	0.41782	0.63196	0.10392	0.32113	0.2397	0.02444	0.26414	0.8015
Verrucomicrobia	0	0	0	0	0.00018	0	0	0	0	7.20E-05	0	0	0.01128	0	0	3.20E-05	0	0	0

Table S25. Faecal bacterial composition in each sample at the genus level between recovered NJI (neonatal jaundice infants) and non-NJI at 1 month

genus	AI023_2	AI019_2	BI006_4	BI008_2	BI013_3	BI021_2	CI008_2	CI011_3	CI015_2	CI016_2	CI021_2	DI018_2	DI024_2	AI020_3	BI020_2	CI006_4	CI009_2	CI013_3	CI017_2
Abiotrophia	0	0	0	0	0	1.90E-05	2.30E-05	3.00E-05	0	0	0	4.30E-05	0	0	0	0	0	2.70E-05	0
Acinetobacter	0	2.50E-05	0	0	0.00036	0.00011	0.00011	0.00217	0	7.10E-05	3.40E-05	0	0.00076	0	4.30E-05	0.00488	0	2.90E-05	9.90E-05
Actinobacillus	0	0	0	0	0	0	0	0	0	0	0	3.40E-05	0	0	0	9.00E-05	0	0	0
Actinomyces	0.00062	0.00075	0.00035	0.00121	0.00236	0.00495	8.50E-05	0.00016	0	0.00036	0.02616	0.00114	0.00147	0.00063	0.00057	0.0093	2.00E-05	5.10E-05	0.00022
Aggregatibacter	3.20E-05	0	0	0	0	0	0	0	0	0	0	0	0	0	0	0	0	0	5.80E-05
Akkermansia	0	0	0	0	0.00018	0	0	0	0	7.20E-05	0	0	0.01128	0	0	3.20E-05	0	0	0
Alistipes	0	0	6.30E-05	0.00015	0	2.40E-05	1.80E-05	0	0	0	0	0.04543	4.00E-05	0.00022	0	0	0	0	0
Alloprevotella	0	0	0	0	0	0	0	0	4.50E-05	0	0	0	0	0	0	0	0	0	0
Anaerococcus	0.53306	0.00089	2.30E-05	3.70E-05	5.70E-05	0	0.00017	2.60E-05	0	0	0	3.20E-05	0	0.00327	0.00013	3.20E-05	0	0	0
Anaerosporobacter	0	0	0	0	0	0	0	0	0	0	0	0	0	0	0	3.10E-05	0	0	0
Anaerostipes	1.80E-05	2.50E-05	0	0	0	1.70E-05	0	0.0002	0	0	6.60E-05	3.40E-05	0	0	0.00016	0	0	0	0
Aquabacterium	0	0	0	0	0	2.60E-05	0	0	0	0	0	0	0	0	0	0	0	0	0
Arthrobacter	0	0	0	3.00E-05	0	3.60E-05	0	0.00015	0	0	3.80E-05	3.60E-05	0	0	0	5.30E-05	0	0	0
Atopobium	0.00024	0.0023	0.0004	0.00064	9.50E-05	0.00567	0.02822	0	0.00943	3.70E-05	3.00E-05	0.00628	0.00013	0.00093	0.00011	0	0	0.0029	0.00024
Bacteroides	0.00192	0.0017	0.00186	0.00246	0.01343	0.03338	0.01859	0.00658	0.00024	7.90E-05	0.00078	0.12388	0.02625	0.29468	0.02002	0.00012	0.01526	0.53952	0.00026
Barnesiella	0	0	3.20E-05	0	0	0	0	0	0	0	0	0	0	0	0	0	0	0	0
Bifidobacterium	0.01754	0.02976	0.63737	0.05047	0.02241	0.01967	0.05764	0.0117	0.09213	0.00712	0.24321	0.11792	0.13832	0.19272	0.51999	0.20845	0.21278	0.01869	0.00432
Bilophila	0	0	0	0	0	0	0	2.30E-05	0	0	0	4.00E-05	0.00073	0	0	0	0	0.00012	0
Blautia	0.00021	5.50E-05	0.00018	0.00016	0	2.70E-05	0	0.00039	0.00016	0	0.00041	0.00017	0.01148	0.00033	3.70E-05	0.00905	0	0	0
Brevundimonas	0	0	0	0	0	0	0	0	0	0	0	0	0.00066	4.50E-05	0	2.40E-05	0	0	0
Campylobacter	0	0	0	0.0001	0	0	0	0	4.00E-05	0	0.00013	0	0	0	0	0	0	2.90E-05	0
Candidate_division_TM7_norank	0	0	0	0	2.30E-05	0	0	2.70E-05	0	0.0004	0	0	0	0.00102	3.70E-05	8.90E-05	0	5.60E-05	0
Chryseobacterium	0	0	0	0	0	0	0	0	0	0	0	0	0	0	0	5.90E-05	0	0	0
Citrobacter	0.00092	0.00072	0.00154	0.0122	0.05862	0.00151	0.0319	0.00993	0	0.00086	0.00064	0.03073	0.01952	0.00588	0.00054	0.02137	2.50E-05	0.00058	0.00658
Clostridium_sensu_stricto	0.04226	0.00032	0.00018	0.0024	0.10123	0.0534	0.00187	0.2869	6.50E-05	0.00285	0.00405	0.0014	0.00032	0.00064	0.00212	0.01053	0	0.00128	0.01576
Collinsella	0.00062	0.00043	0.00174	0.00095	0	0.00015	0	0.0004	4.20E-05	0	0.00013	8.10E-05	0.04518	0.05079	0.00029	0	5.40E-05	0.03592	0
Comamonadaceae_unclassified	0	0	0	0	0	0	0	2.50E-05	0	0	0	0	0	0	0	0	0	0	0
Coprococcus	7.10E-05	0	0	0	0	0	0	0.00035	3.70E-05	0	0	0	0	0	3.50E-05	0	0	0	0
Coriobacteriaceae_uncultured	6.40E-05	6.80E-05	0	0	0	0	0	6.20E-05	0	0	0.0002	0.00025	0.01926	0.00019	0	0	0.00472	0	0
Corynebacterium	5.80E-05	4.30E-05	0.00052	1.00E-04	0	0	0.00011	0	0.0001	5.00E-05	0.0001	0.00013	8.70E-05	9.50E-05	0.00021	0.00094	0.00133	8.90E-05	0
Cryptobacterium	0	5.50E-05	0	0	6.60E-05	0	0	0	0	0	0	0	0	0	0	0	0	0	0
Curvibacter	0	0	0	0	0	3.20E-05	0	5.30E-05	0	0	0	4.70E-05	0	0	0	0	0	2.60E-05	0
Cyanobacteria_norank	0	0	0	0	0	0	0	0	0	0.00034	8.60E-05	0	0	0	0	0	0	3.20E-05	0
Defluviitaleaceae_Incertae_Sedis	0	2.60E-05	0	0	0.00014	0.00031	0	0	0	6.40E-05	0.01012	0	0	0	0	0	0	0	0
Dialister	0	0	0	0	0	0	0	0	3.70E-05	0	2.50E-05	0	0.01041	0	0	0	0	0	0
Dolosigranulum	0	0	0	0	0	0	0	0	0.00013	0	0	0	0	4.10E-05	0	0	0	3.30E-05	0
Dorea	4.60E-05	2.70E-05	0	0	5.20E-05	2.50E-05	0	0.00016	7.50E-05	0	0	0	0	0.00047	0	0	0	0	3.80E-05

Table S25. Faecal bacterial composition in each sample at the genus level between recovered NJI (neonatal jaundice infants) and non-NJI at 1 month (continued)

genus	AI023_2	AI019_2	BI006_4	BI008_2	BI013_3	BI021_2	CI008_2	CI011_3	CI015_2	CI016_2	CI021_2	DI018_2	DI024_2	AI020_3	BI020_2	CI006_4	CI009_2	CI013_3	CI017_2
Dysgonomonas	8.00E-05	3.40E-05	0	6.90E-05	0	0	0	0	0	0	0	0	0	0.00019	0	0	0	0	0
Eggerthella	8.30E-05	0	0.00011	0	3.50E-05	0.00016	0	4.80E-05	0	0	0	7.90E-05	0.00109	0	0.004	2.40E-05	2.90E-05	0.00069	0
Enhydrobacter	0	0	0	0	0	0	7.30E-05	0	0	0	0	0	0	0	0	0	0	0	0
Enterobacter	0.00965	0.00042	0.00063	0.02024	0.0134	0.01237	0.01008	0.02399	0	0.00457	0.00039	0.02376	0.00904	0.00425	0.00735	0.00779	0.00117	0.00676	0.00279
Enterobacteriaceae_unclassified	0.00967	0.00045	0.00073	0.02216	0.01695	0.00837	0.02163	0.02267	0	0.00263	0.00046	0.012	0.01598	0.00293	0.0058	0.0193	0.00102	0.00878	0.00387
Enterococcus	0.01659	0.00067	0.00052	0.00225	0.0025	0.00025	0.00013	0.17099	0.00066	0.0002	0	0.00017	9.40E-05	0.00206	0.00449	0.01738	0	2.40E-05	0.04741
Erysipelotrichaceae_Incertae_Sedis	0.00022	0.00018	2.40E-05	9.10E-05	0.00037	0	0	0.0005	0	0.10684	0.00016	0.00012	0.00866	0.00092	0	6.90E-05	0	0.02779	2.80E-05
Erysipelotrichaceae_uncultured	0	2.20E-05	0	0	0	0	0	0	0	0	3.50E-05	0	0	0.00023	0	2.90E-05	0	0	0
Escherichia-Shigella	0.02504	0.00605	0.05229	0.02695	0.08221	0.18914	0.00605	0.0042	0.11853	0.16919	0.27125	0.05406	0.30709	0.02127	0.16057	0.0427	7.80E-05	0.14375	0.66567
Faecalibacterium	0.00015	0.00014	0	0	4.90E-05	5.70E-05	2.30E-05	0.00017	4.10E-05	0	3.30E-05	8.20E-05	4.30E-05	0	0	3.40E-05	0	2.70E-05	3.70E-05
Finegoldia	0.00649	0	0	0	5.80E-05	0	2.50E-05	0	0	6.50E-05	0	0	9.20E-05	0	0	0.00031	0	0	0
Flavonifractor	1.90E-05	2.10E-05	5.70E-05	0	0	0	0	5.60E-05	0	2.10E-05	0	7.70E-05	0	4.90E-05	0	0	0	0.00233	0
Fusobacterium	0	0	0	2.60E-05	0	0	0	2.00E-05	0	0	0.00024	0	0	0	0	0	0	0	0
Gardnerella	2.10E-05	0	0.00019	0	0	0	0	0	0	0	0	0	0	0	3.90E-05	0	0	3.50E-05	0
Gemella	0.00042	0.00164	0.00465	0.01093	5.60E-05	0.00131	0.00047	0	4.40E-05	0.00164	0.01295	0	0.00023	0.00056	0.0006	0.00137	0.00016	0.00011	0.00017
Gordonibacter	0	0	0	0	0	0	0	0	0	0	0	0	0	0	0	0.01004	0	0	0
Granulicatella	8.90E-05	0	0	0.00036	2.60E-05	0.00012	0	2.70E-05	0	0	0	7.70E-05	0	0	0.00012	0.0667	0	3.40E-05	0
Haemophilus	0.01546	0.55511	0.00209	0.10369	0.00026	0.00481	0.00617	5.10E-05	0	0.13124	0.08559	0.00189	0.00039	0.00373	0.00144	6.50E-05	0	2.60E-05	0.00012
Halomonas	0	0	0	0	3.00E-05	0	0	0	4.50E-05	0	0	0	0	0	0	0	0	0	0
Howardella	0	0	0	0	0	0	0	0	0	0	0	4.20E-05	0	0	0	0	0	0	0
Klebsiella	0.23966	0.00416	0.00914	0.29873	0.04335	0.43102	0.06721	0.29828	0.00022	0.07373	0.00722	0.29378	0.20991	0.06068	0.14408	0.10987	0.02211	0.1035	0.09129
Kocuria	0	0	0	0	0	0	0	0	0	0	2.10E-05	0	0	0	0	0	0	0	0
Lachnoanaerobaculum	2.70E-05	0	0	2.50E-05	0	5.90E-05	0	0	0	0	0.00055	0	0	0	0	0	0	0	0
Lachnospira	2.00E-05	0.00011	0	0	0	0	2.20E-05	0	8.00E-05	3.40E-05	0	0	0	0	0	0	0	0	0
Lachnospiraceae_Incertae_Sedis	0.00229	0.00092	0.00014	0.00145	2.40E-05	0.00035	9.20E-05	0.0004	0.00056	0.00024	0.00014	0.00074	0.00108	0.06666	0.00112	8.60E-05	0.00893	0.01826	0.00011
Lachnospiraceae_unclassified	0	0	0	0	0	0	0	4.00E-05	0	0	0	0	0	0	0	0	0	0	0
Lachnospiraceae_uncultured	0	0	0	0	0	0	0	0	0	8.00E-05	0	0	0	0	0	0	0	0	0
Lactobacillales_unclassified	0	3.40E-05	2.60E-05	0	0	0	0	0	0	0	0.00013	0	0	0	4.90E-05	0	0	0	0
Lactobacillus	0.01514	0.01862	0.07418	0.22973	0.00149	0.01447	0.00039	0.03658	0.30484	0.00095	0.00074	0.00052	0.0386	0.00672	0.00024	0.00215	0	6.80E-05	0.00019
Lactococcus	0	0	0	0	0	0	0	0	0	0	0.00015	0	4.40E-05	0	7.50E-05	0	0	0	0
Megamonas	0	3.40E-05	0	3.70E-05	0	0	0	0	8.90E-05	0	2.30E-05	0.00134	0.00041	0	0	0	0	2.40E-05	0
Megasphaera	2.00E-05	0.0001	2.70E-05	2.90E-05	2.30E-05	7.50E-05	2.10E-05	0.00068	0	3.30E-05	0	0	0.00212	0	4.90E-05	0	0	0	0
Morganella	4.90E-05	1.90E-05	0	3.40E-05	0	0	0	2.50E-05	0	0	0	0	0	9.50E-05	0	0	0	0	0
Negativicoccus	2.90E-05	0	0	0	0	0	0	0.00122	0	0	3.20E-05	0.00442	0.00072	0	0	0.00732	0	3.00E-05	0
Neisseria	0.00011	0	0	0	0	0	0	0	0	0	0	4.80E-05	0	0.00044	0	2.30E-05	0	0.00014	0
Novosphingobium	0	0	0	0	0	0	0	5.20E-05	0	0	0	0	0	0	0	0	0	0	0
Odoribacter	0	0	0	0	0	0	0	0	8.00E-05	0	0	0	0	0	0	0	0	0	0

Table S25. Faecal bacterial composition in each sample at the genus level between recovered NJI (neonatal jaundice infants) and non-NJI at 1 month (continued)

genus	AI023_2	AI019_2	BI006_4	BI008_2	BI013_3	BI021_2	CI008_2	CI011_3	CI015_2	CI016_2	CI021_2	DI018_2	DI024_2	AI020_3	BI020_2	CI006_4	CI009_2	CI013_3	CI017_2
Oribacterium	0	0	0	0	0	0	0	0	0	0	2.80E-05	0	0	0	0	0	0	0	0
Parabacteroides	0.0006	0.00039	0.0011	0.00146	5.10E-05	8.30E-05	0.20188	0.00195	8.10E-05	0	3.10E-05	0.20883	0.00259	0.23616	0.00625	0.00041	0.0015	0.0233	0
Paracoccus	0	0	0	0	0	0	0	0	0	3.30E-05	0	0	0	0	0	0	0	0	0
Paraprevotella	0	0	0	0	0	0	0	0	0	0	0	0	0.00013	0	0	0	0	0	0
Parascardovia	0	0	0	0	0	0	0	0	0.00015	0	0	0	0	0	0	0	0	0.00021	0
Parasutterella	0	0	0	0	0	0	0	0	0	0	0	0	0.00116	0	0	0	0	0	0
Pelagibacterium	0	0	0	0	0	0	0	0	3.70E-05	0	0	0	0	0	0	0	0	0	0
Peptoniphilus	5.00E-05	0	0	0	0	0	2.10E-05	0.00066	0	0	3.60E-05	0	0	4.10E-05	3.50E-05	0	0	0	0
Peptostreptococcaceae_Incertae_Sed	0.00016	0.00024	0.00012	3.20E-05	0	2.90E-05	6.80E-05	0.01865	0	0.00013	0.00015	0.00013	0	0.00064	4.20E-05	0.00017	0	0	0.00091
Peptostreptococcus	0	0	0	0	0	0	0	0	0	0	0	0	0	0	0	0	0	0	2.50E-05
Phascolarctobacterium	3.20E-05	0	0	9.30E-05	0	0	0	0	4.30E-05	0	0	0	0	4.80E-05	0	0	0	0.001	0
Prevotella	4.80E-05	0.00055	6.90E-05	2.90E-05	3.80E-05	2.50E-05	1.70E-05	3.10E-05	0.00051	0	6.60E-05	8.50E-05	4.50E-05	0	0.00013	0	0	0	0
Propionibacterium	0.00033	0	0	0	2.40E-05	0	4.50E-05	0	0	2.50E-05	0	0	0	0	0.00023	0.06548	0	0	0
Proteus	2.90E-05	4.30E-05	0	0	0	0	0	0	0	0	6.60E-05	0	0	9.20E-05	0	0	0	0	0
Pseudobutyrivibrio	0	4.30E-05	0	0	0	0	0	0.0001	8.10E-05	0	0	0	0	0	0	0	0	0	0
Pseudochrobactrum	0	0	0	0	0	0	0	0	0	0	0	0	4.50E-05	0	0	2.20E-05	0	0	0
Ralstonia	0	1.90E-05	1.50E-05	3.70E-05	0	7.60E-05	0	0.00031	3.80E-05	0	0	4.60E-05	0	0	0	5.70E-05	2.90E-05	0	0
Raoultella	6.80E-05	0	8.00E-05	0.00057	0.00047	0.0003	0.02202	0.00031	0	0.00017	0.00023	0.00014	0.00336	0.00074	4.30E-05	0.00473	0	2.50E-05	0.00101
Rhizobium	0	0	0	0	0	0	0	7.00E-05	0	0	0	0	0	0	0	0	0	0	0
Roseburia	0	2.90E-05	0	0	0	0	0	0	8.40E-05	0	0	0	5.10E-05	0	0	0	0	0	0
Rothia	0.00079	0.00505	0.00049	0.00125	0.00032	0.00037	0.10091	0.02182	0.00866	0.00239	0.01439	0.00021	0.00027	0.00334	0.00065	0.00243	0	0.00085	0.09219
Ruminococcaceae_Incertae_Sedis	0	0	1.50E-05	0	0	0	0	9.10E-05	0	0	0	0	0	5.80E-05	0	0	0	0	0
Ruminococcaceae_uncultured	0	0	7.40E-05	5.40E-05	0	2.10E-05	0	0.00024	3.80E-05	3.40E-05	0	0	0	4.80E-05	0	0	0	0.00105	0
Ruminococcus	8.30E-05	9.10E-05	0	0	0	0.00012	0	0	0.00028	0	3.80E-05	0	0	0	0.00014	0	0	0	2.90E-05
Salmonella	0.00032	0.00051	0.00068	0.0027	0.04615	0.00027	0.02835	0.00045	0	0.00036	0.00142	0.00107	0.0629	0.00108	0.00065	0.02829	0	0.00016	0.03001
Scardovia	0.00014	0.00398	9.90E-05	0.00011	0	1.80E-05	7.40E-05	5.20E-05	0.00011	0	0.00476	0	0	0.00043	0	0	0	0	0
Serratia	9.30E-05	7.40E-05	0.00133	0.00019	0.00015	0.00021	0.00014	7.90E-05	0	0.00035	0.00019	0.00015	0.00014	0.00019	0.00062	0.00048	0	0.00022	0
Solobacterium	0	0	0	0.00017	0	0	0	0	0	0	0	0	0	0	0	0	0	0	0
Sphingomonas	0	0	0	0	0	2.60E-05	2.50E-05	0	0	0	0	3.50E-05	9.30E-05	0	0	0	0	0	0
Staphylococcus	0.01418	0.0011	0.0084	0.00379	0.01171	0.03555	0.03028	0.00999	0.00234	0.00472	0.00017	0.00102	0.00495	0.00069	0.00222	0.02341	0.14533	0.00058	0.00219
Stenotrophomonas	0	0	0	0	0	7.80E-05	0	0	0	0	0	0	0	0	0	0	0	0	0
Stomatobaculum	0	0	0	0	0	0	0	0	0	0	0.00024	0	0	0	0	0	0	0	0
Streptococcus	0.04131	0.22339	0.09673	0.14933	0.56935	0.13694	0.35406	0.0637	0.42375	0.17614	0.0992	0.02517	0.04167	0.02872	0.11144	0.32328	0.58472	0.01148	0.03411
Subdoligranulum	0	2.20E-05	0	5.40E-05	0	2.00E-05	0	0	3.60E-05	0	7.40E-05	0	0	0	4.40E-05	0	0	0	3.00E-05
Sutterella	0	0	2.20E-05	9.50E-05	0	1.30E-05	0	0	0	0	0	0	0.00018	0.00251	0	0	0	0	0
Turicibacter	0	0	0	0	0	0	0	0	0	0	3.20E-05	0	0	0	0	0	0	0	0
Veillonella	0.00279	0.1385	0.10171	0.05234	0.0119	0.04395	0.01097	0.00218	0.03605	0.31184	0.21258	0.04225	0.0009	0.00248	0.00321	0.00096	0.00074	0.0494	0.00021
Zoogloea	0	2.80E-05	0	0	0	0	2.40E-05	0	0	0	0	0	0	0	0	0	0	0	0
mitochondria_norank	0	0	0	0	0	0	0	0	0	6.40E-05	0	0	0	0	0	0	0	0	0

Table S26. Microbial composition and comparison at the phylum level between recovered NJI (neonatal jaundice infants) and non-NJI at 1 month

ID	non-NJI-median	non-NJI-mean	non-NJI-se	recovered NJI-median	recovered NJI-mean	recovered NJI-se	p-value	z-score	Sig_mark	q-value
Firmicutes	0.3986(0.2871,0.6059)	0.438445846	0.060760599	0.1205(0.1138,0.3788)	0.276433	0.108849379	0.244066047	-1.164883761		0.450891936
Proteobacteria	0.3676(0.2619,0.4877)	0.370099231	0.050823311	0.2519(0.1379,0.3069)	0.292469833	0.111191589	0.28180746	-1.076267901		0.450891936
Actinobacteria	0.0548(0.031,0.1871)	0.136794538	0.048377212	0.234(0.1275,0.2848)	0.241207	0.067980368	0.071502285	-1.80227414		0.28600914
Bacteroidetes	0.0042(0.0026,0.0291)	0.053684615	0.031670961	0.0216(0.0046,0.405)	0.1896785	0.113151975	0.578873655	-0.555030711		0.762207656
Verrucomicrobia	<0.0001(<0.0001,<0.0001)	0.000886846	0.000866376	<0.0001(<0.0001,<0.0001)	5.33333E-06	5.33333E-06	0.666931699	-0.430362872		0.762207656
Candidate_division_TM7	<0.0001(<0.0001,<0.0001)	3.43846E-05	3.03291E-05	<0.0001(<0.0001,<0.0001)	0.000201	0.000165186	0.048155042	-1.975997658	*	0.28600914
Cyanobacteria	<0.0001(<0.0001,<0.0001)	3.29231E-05	2.65867E-05	<0.0001(<0.0001,<0.0001)	5.33333E-06	5.33333E-06	1	0		1
Fusobacteria	<0.0001(<0.0001,<0.0001)	2.16154E-05	1.79435E-05	<0.0001(<0.0001,<0.0001)	0	0	0.240554568	-1.17360178		0.450891936

Table S27. Difference of fecal microbial communities at the genus level between recovered NJI (neonatal jaundice infants) and non-NJI at 1 month

ID	non-NJI-median	non-NJI-mean	non-NJI-se	recovered NJI-median	recovered NJI-mean	recovered NJI-se	p-value	z-score	Sig_mark	q-value
Streptococcus	0.1369(0.0637,0.2234)	0.184673462	0.046343766	0.0728(0.0301,0.2703)	0.182291	0.093374538	0.521376972	-0.64122429		0.992145396
Bifidobacterium	0.0505(0.0197,0.1179)	0.111172615	0.047661761	0.2006(0.0622,0.2117)	0.192825	0.076055427	0.467049978	-0.7272873		0.992145396
Klebsiella	0.0737(0.0091,0.2938)	0.152030769	0.041083209	0.0974(0.0683,0.1083)	0.0885875	0.017281572	0.8982751	-0.12784063		1
Escherichia-Shigella	0.0541(0.025,0.1692)	0.100926846	0.028618484	0.0932(0.0266,0.1564)	0.1723395	0.10224706	1	0		1
Bacteroides	0.0025(0.0017,0.0186)	0.017781077	0.009345875	0.0176(0.004,0.226)	0.1449755	0.091731523	0.467049978	-0.7272873		0.992145396
Veillonella	0.0422(0.011,0.1017)	0.074456846	0.026301751	0.0017(0.0008,0.003)	0.0094995	0.007992678	0.0285272	-2.18995914	*	0.499226006
Haemophilus	0.0048(0.0004,0.0856)	0.069749769	0.042404861	<0.0001(<0.0001,0.0011)	0.000896167	0.000610113	0.043578892	-2.01811878	*	0.508420411
Lactobacillus	0.0151(0.0009,0.0386)	0.056634462	0.0269288	0.0002(<0.0001,0.0017)	0.0015615	0.0010846	0.009214212	-2.60400237	**	0.32249742
Parabacteroides	0.0006(<0.0001,0.0019)	0.032233692	0.021314538	0.0039(0.0007,0.019)	0.044603167	0.038479893	0.334451036	-0.96518724		0.989719775
Anaerococcus	<0.0001(<0.0001,<0.0001	0.041099154	0.040996375	<0.0001(<0.0001,0.0001)	0.000571333	0.000539519	0.891223816	-0.13675581		1
Clostridium_sensu_stricto	0.0024(0.0003,0.0423)	0.038249308	0.022390994	0.0017(0.0008,0.0084)	0.005054667	0.0026619	0.578873655	-0.55503071		0.992145396
Staphylococcus	0.005(0.0023,0.0117)	0.009862154	0.003094561	0.0022(0.0011,0.0181)	0.0290685	0.023528612	0.578873655	-0.55503071		0.992145396
Enterococcus	0.0005(0.0002,0.0022)	0.015001615	0.013058093	0.0033(0.0005,0.0142)	0.011895167	0.007584201	0.59856463	-0.52646577		0.992145396
Rothia	0.0012(0.0004,0.0087)	0.012070385	0.007629629	0.0016(0.0007,0.0031)	0.016575667	0.01513188	1	0		1
Salmonella	0.0007(0.0004,0.0027)	0.011167154	0.005818086	0.0009(0.0003,0.0215)	0.010033833	0.006051745	0.860695736	-0.17548859		1
Citrobacter	0.0015(0.0009,0.0195)	0.013006231	0.004957576	0.0032(0.0005,0.0064)	0.005829333	0.003323605	0.322865989	-0.98858549		0.989719775
Enterobacteriaceae_unclassified	0.0097(0.0007,0.017)	0.010284538	0.002475474	0.0048(0.0032,0.008)	0.006949167	0.002694848	0.765369306	-0.29843746		0.992145396
Enterobacter	0.0096(0.0006,0.0134)	0.009887846	0.002414305	0.0055(0.0032,0.0072)	0.005018167	0.001102758	0.322865989	-0.98858549		0.989719775
Erysipelotrichaceae_Incertae_Sedis	0.0002(<0.0001,0.0004)	0.009012538	0.008178269	<0.0001(<0.0001,0.0007)	0.0048015	0.004599659	0.690494257	-0.39818441		0.992145396
Collinsella	0.0001(<0.0001,0.0006)	0.003825	0.003449364	0.0002(<0.0001,0.027)	0.014508	0.009321564	1	0		1
Lachnospiraceae_Incertae_Sedis	0.0004(0.0001,0.0009)	0.000648692	0.000181611	0.005(0.0004,0.0159)	0.015861167	0.010564558	0.244066047	-1.16488376		0.989719775
Granulicatella	<0.0001(<0.0001,<0.0001	0.000053	2.75015E-05	<0.0001(<0.0001,<0.0001)	0.011142167	0.011111782	0.635367495	-0.47418569		0.992145396
Propionibacterium	<0.0001(<0.0001,<0.0001	3.24615E-05	2.49501E-05	<0.0001(<0.0001,0.0002)	0.010951833	0.010906097	0.709845019	-0.37206424		0.992145396
Atopobium	0.0004(<0.0001,0.0057)	0.004113846	0.002182443	0.0002(<0.0001,0.0008)	0.000696667	0.000462151	0.333600768	-0.96688652		0.989719775
Actinomyces	0.0007(0.0003,0.0015)	0.003047154	0.00196095	0.0004(<0.0001,0.0006)	0.001799167	0.001504803	0.367610202	-0.90095884		0.989719775
Alistipes	<0.0001(<0.0001,<0.0001	0.003517	0.003492686	<0.0001(<0.0001,<0.0001)	3.63333E-05	3.63333E-05	0.361769287	-0.91199892		0.989719775
Gemella	0.0005(<0.0001,0.0016)	0.002640308	0.001202812	0.0004(0.0002,0.0006)	0.000493667	0.000195246	0.692954089	-0.39484933		0.992145396
Raoultella	0.0002(<0.0001,0.0005)	0.002130692	0.001675251	0.0004(<0.0001,0.0009)	0.001090333	0.000749011	1	0		1
Coriobacteriaceae_uncultured	<0.0001(<0.0001,<0.0001	0.001530154	0.001477405	<0.0001(<0.0001,0.0001)	0.0008175	0.000781084	0.807171191	-0.24407699		1
Blautia	0.0002(<0.0001,0.0002)	0.001019615	0.000872935	<0.0001(<0.0001,0.0003)	0.0015685	0.001496417	0.47584914	-0.71299453		0.992145396
Peptostreptococcaceae_Incertae_Sed	0.0001(<0.0001,0.0002)	0.001516	0.001427652	0.0001(<0.0001,0.0005)	0.0002935	0.000158707	0.756789874	-0.30969899		0.992145396
Akkermansia	<0.0001(<0.0001,<0.0001	0.000886846	0.000866376	<0.0001(<0.0001,<0.0001)	5.33333E-06	5.33333E-06	0.666931699	-0.43036287		0.992145396
Defluviitaleaceae_Incertae_Sedis	<0.0001(<0.0001,<0.0001	0.000820692	0.000775679	<0.0001(<0.0001,<0.0001)	0	0	0.10087988	-1.64060287		0.882698946
Dialister	<0.0001(<0.0001,<0.0001	0.000805462	0.000800302	<0.0001(<0.0001,<0.0001)	0	0	0.240554568	-1.17360178		0.989719775
Gordonibacter	<0.0001(<0.0001,<0.0001	0	0	<0.0001(<0.0001,<0.0001)	0.0016725	0.0016725	0.174231388	-1.35873244		0.989719775

Table S28. Detailed data for linear discriminant analysis (LDA) of the faecal microbial OTUs between recovered NJI (neonatal jaundice infants) and non-NJI at 1 month

Biomaker_names	Logarithm v	Groups	LDA_value	P_value
d_Bacteria.p_Firmicutes.c_Negativicutes.o_Selenomonadales.f_Acidaminococcaceae	2.2438645			-
d_Bacteria.p_Proteobacteria.c_Deltaproteobacteria	1.7836837			-
d_Bacteria.p_Firmicutes.c_Clostridia.o_Clostridiales.f_Lachnospiraceae.g_Blautia	3.1954845			-
d_Bacteria.p_Proteobacteria.c_Gammaproteobacteria.o_Enterobacteriales	5.4766754			-
d_Bacteria.p_Firmicutes.c_Bacilli.o_Bacillales	4.4707363			-
d_Bacteria.p_Proteobacteria.c_Gammaproteobacteria.o_Oceanospirillales.f_Halomonadaceae.g_Halomonas	0.7611179			-
d_Bacteria.p_Bacteroidetes	5.2780181			-
d_Bacteria.p_Proteobacteria.c_Alphaproteobacteria.o_Sphingomonadales.f_Sphingomonadaceae	1.2496686			-
d_Bacteria.p_Bacteroidetes.c_Bacteroidia.o_Bacteroidales.f_Bacteroidaceae	5.1612946			-
d_Bacteria.p_Actinobacteria.c_Actinobacteria.o_Actinomycetales.f_Actinomycetaceae	3.4838944			-
d_Bacteria.p_Firmicutes.c_Clostridia.o_Clostridiales.f_Lachnospiraceae.g_Coprococcus	1.5469221			-
d_Bacteria.p_Firmicutes.c_Clostridia.o_Clostridiales.f_Clostridiales_Family_XI.g_Anaerococcus	4.6138329			-
d_Bacteria.p_Firmicutes.c_Clostridia.o_Clostridiales.f_Lachnospiraceae.g_Stomatobaculum	1.2571245			-
d_Bacteria.p_Proteobacteria.c_Betaproteobacteria.o_Rhodocyclales.f_Rhodocyclaceae	0.60206			-
d_Bacteria.p_Proteobacteria.c_Epsilonproteobacteria.o_Campylobacterales	1.3238072			-
d_Bacteria.p_Proteobacteria.c_Betaproteobacteria.o_Burkholderiales.f_Alcaligenaceae.g_Sutterella	2.6216955			-
d_Bacteria.p_Firmicutes.c_Erysipelotrichia	3.9558172			-
d_Bacteria.p_Actinobacteria.c_Actinobacteria.o_Micrococcales.f_Micrococcaceae.g_Rothia	4.219471			-
d_Bacteria.p_Verrucomicrobia.c_Verrucomicrobiae	2.9478483			-
d_Bacteria.p_Proteobacteria.c_Alphaproteobacteria.o_Caulobacterales.f_Caulobacteraceae	1.7062581			-
d_Bacteria.p_Proteobacteria.c_Alphaproteobacteria.o_Rhizobiales.f_Hyphomicrobiaceae.g_Pelagibacterium	0.4542584			-
d_Bacteria.p_Firmicutes.c_Clostridia.o_Clostridiales.f_Lachnospiraceae	4.2443307			-
d_Bacteria.p_Firmicutes.c_Erysipelotrichia.o_Erysipelotrichales	3.9558172			-
d_Bacteria.p_Firmicutes.c_Negativicutes.o_Selenomonadales	4.8817173	non-NJI	4.515771	0.014059
d_Bacteria.p_Proteobacteria.c_Betaproteobacteria.o_Burkholderiales.f_Comamonadaceae.g_Curvibacter	1.0066306			-
d_Bacteria.p_Proteobacteria.c_Betaproteobacteria.o_Burkholderiales.f_Burkholderiaceae.g_Ralstonia	1.6168389			-
d_Bacteria.p_Actinobacteria	5.3823899			-
d_Bacteria.p_Proteobacteria.c_Gammaproteobacteria.o_Pasteurellales.f_Pasteurellaceae	4.8435744	non-NJI	4.478763	0.039209
d_Bacteria.p_Proteobacteria.c_Gammaproteobacteria.o_Pasteurellales	4.8435744	non-NJI	4.478763	0.039209
d_Bacteria.p_Proteobacteria.c_Alphaproteobacteria.o_Rhodobacterales	0.4045706			-
d_Bacteria.p_Firmicutes.c_Clostridia.o_Clostridiales.f_Lachnospiraceae.g_Dorea	1.9302696			-
d_Bacteria.p_Firmicutes.c_Clostridia.o_Clostridiales.f_Ruminococcaceae.g_Faecalibacterium	1.7814792			-
d_Bacteria.p_Proteobacteria.c_Gammaproteobacteria.o_Enterobacteriales.f_Enterobacteriaceae.g_Raoultella	3.3285207			-
d_Bacteria.p_Verrucomicrobia.c_Verrucomicrobiae.o_Verrucomicrobiales.f_Verrucomicrobiaceae.g_Akkermansia	2.9478483			-
d_Bacteria.p_Proteobacteria.c_Gammaproteobacteria.o_Pasteurellales.f_Pasteurellaceae.g_Haemophilus	4.8435428	non-NJI	4.479923	0.039209
d_Bacteria.p_Proteobacteria.c_Alphaproteobacteria.o_Rhizobiales.f_Brucellaceae	0.5642714			-
d_Bacteria.p_Fusobacteria.c_Fusobacteriia.o_Fusobacteriales	1.334763			-
d_Bacteria.p_Proteobacteria.c_Alphaproteobacteria.o_Sphingomonadales.f_Sphingomonadaceae.g_Novosphingobium	0.60206			-
d_Bacteria.p_Firmicutes.c_Bacilli.o_Bacillales.f_Staphylococcaceae	4.4634226			-
d_Bacteria.p_Firmicutes.c_Bacilli.o_Lactobacillales.f_Lactobacillales_unclassified.g_Lactobacillales_unclassified	1.1648102			-
d_Bacteria.p_Firmicutes.c_Bacilli.o_Lactobacillales.f_Streptococcaceae.g_Streptococcus	5.2664045			-
d_Bacteria.p_Firmicutes.c_Clostridia.o_Clostridiales	4.9260447			-
d_Bacteria.p_Actinobacteria.c_Actinobacteria.o_Bifidobacteriales.f_Bifidobacteriaceae.g_Bifidobacterium	5.2851633			-
d_Bacteria.p_Proteobacteria.c_Deltaproteobacteria.o_Desulfovibrionales.f_Desulfovibrionaceae.g_Bilophila	1.7836837			-

Table S28. Detailed data for linear discriminant analysis (LDA) of the faecal microbial OTUs between recovered NJI (neonatal jaundice infants) and non-NJI at 1 month (continued)

Biomaker names	Logarithm v	Groups	LDA value	P value
d Bacteria.p Proteobacteria.c Gammaproteobacteria.o Xanthomonadales.f Xanthomonadaceae	0.7781513			-
d Bacteria.p Firmicutes.c Bacilli	5.4296113			-
d Bacteria.p Actinobacteria.c Actinobacteria.o Micrococcales.f Micrococcaceae	4.2197024			-
d Bacteria.p Proteobacteria.c Gammaproteobacteria.o Xanthomonadales.f Xanthomonadaceae.g Stenotrophomonas	0.7781513			-
d Bacteria.p Actinobacteria.c Actinobacteria	5.3823899			-
d Bacteria.p Bacteroidetes.c Flavobacteriia.o Flavobacteriales.f Flavobacteriaceae	0.9927008			-
d Bacteria.p Firmicutes.c Bacilli.o Bacillales.f Bacillales Family XI.g Gemella	3.4216545			-
d Bacteria.p Proteobacteria.c Gammaproteobacteria.o Enterobacteriales.f Enterobacteriaceae.g Proteus	1.1856366			-
d Bacteria.p Firmicutes.c Erysipelotrichia.o Erysipelotrichales.f Erysipelotrichaceae.g Turicibacter	0.3912066			-
d Bacteria.p Firmicutes.c Negativicutes.o Selenomonadales.f Veillonellaceae.g Megamonas	2.171614			-
d Bacteria.p Actinobacteria.c Actinobacteria.o Coriobacteriales.f Coriobacteriaceae.g Coriobacteriaceae uncultured	3.1847351			-
d Bacteria.p Firmicutes.c Negativicutes	4.8817173	non-NJI	4.515771	0.014059
d Bacteria.p Firmicutes.c Bacilli.o Lactobacillales.f Streptococcaceae.g Lactococcus	1.1648102			-
d Bacteria.p Proteobacteria.c Betaproteobacteria.o Burkholderiales.f Comamonadaceae	1.1485077			-
d Bacteria.p Proteobacteria.c Alphaproteobacteria.o Rickettsiales.f mitochondria.g mitochondria norank	0.6922366			-
d Bacteria.p Proteobacteria.c Alphaproteobacteria.o Sphingomonadales.f Sphingomonadaceae.g Sphingomonas	1.1389097			-
d Bacteria.p Proteobacteria.c Alphaproteobacteria.o Rhizobiales.f Hyphomicrobiaceae	0.4542584			-
d Bacteria.p Cyanobacteria.c Cyanobacteria.o Cyanobacteria norank.f Cyanobacteria norank	1.5175004			-
d Bacteria.p Proteobacteria.c Gammaproteobacteria.o Enterobacteriales.f Enterobacteriaceae.g Salmonella	4.0479425			-
d Bacteria.p Firmicutes.c Clostridia.o Clostridiales.f Peptostreptococcaceae.g Peptostreptococcus	0.6197888			-
d Bacteria.p Bacteroidetes.c Bacteroidia.o Bacteroidales.f Prevotellaceae.g Alloprevotella	0.5392692			-
d Bacteria.p Fusobacteria.c Fusobacteriia.o Fusobacteriales.f Fusobacteriaceae.g Fusobacterium	1.334763			-
d Bacteria.p Bacteroidetes.c Bacteroidia.o Bacteroidales.f Prevotellaceae.g Paraprevotella	1.0099083			-
d Bacteria.p Firmicutes.c Clostridia.o Clostridiales.f Defluviitaleaceae	2.9141804			-
d Bacteria.p Firmicutes.c Clostridia.o Clostridiales.f Clostridiaceae	4.5826236			-
d__Bacteria.p__Candidate_division_TM7.c__Candidate_division_TM7_norank.o__Candidate_division_TM7_norank.f__Candidate_division_TM7_norank.g__Candidate_division_TM7_norank	2.3031961	recovered NJI	3.442284	0.042697
d Bacteria.p Firmicutes.c Clostridia.o Clostridiales.f Lachnospiraceae.g Pseudobutyrivibrio	1.2363047			-
d Bacteria.p Firmicutes.c Bacilli.o Lactobacillales.f Carnobacteriaceae.g Dolosigranulum	1.0910805			-
d Bacteria.p Cyanobacteria	1.5175004			-
d Bacteria.p Firmicutes.c Clostridia.o Clostridiales.f Lachnospiraceae.g Lachnospiraceae unclassified	0.4881166			-
d Bacteria.p Proteobacteria.c Alphaproteobacteria.o Rhizobiales.f Rhizobiaceae	0.7311547			-
d Bacteria.p Firmicutes.c Clostridia.o Clostridiales.f Ruminococcaceae.g Subdoligranulum	1.1999239			-
d Bacteria.p Actinobacteria.c Actinobacteria.o Coriobacteriales.f Coriobacteriaceae.g Cryptobacterium	0.968842			-
d Bacteria.p Proteobacteria.c Betaproteobacteria.o Burkholderiales.f Comamonadaceae.g Comamonadaceae unclassified	0.2839967			-
d Bacteria.p Actinobacteria.c Actinobacteria.o Propionibacteriales.f Propionibacteriaceae.g Propionibacterium	4.0394868			-
d Bacteria.p Proteobacteria.c Alphaproteobacteria	1.9433423			-
d Bacteria.p Proteobacteria	5.5683182			-
d Bacteria.p Proteobacteria.c Betaproteobacteria.o Burkholderiales.f Alcaligenaceae.g Parasutterella	1.9516364			-
d Bacteria.p Proteobacteria.c Gammaproteobacteria.o Pseudomonadales.f Moraxellaceae.g Acinetobacter	2.9248821			-
d Bacteria.p Actinobacteria.c Actinobacteria.o Bifidobacteriales.f Bifidobacteriaceae.g Gardnerella	1.2103391			-
d Bacteria.p Firmicutes.c Clostridia.o Clostridiales.f Clostridiales Family XI	4.6198836			-
d Bacteria.p Cyanobacteria.c Cyanobacteria	1.5175004			-
d Bacteria.p Firmicutes.c Negativicutes.o Selenomonadales.f Veillonellaceae.g Negativicoccus	3.0879588			-
d Bacteria.p Cyanobacteria.c Cyanobacteria.o Cyanobacteria norank	1.5175004			-

Table S28. Detailed data for linear discriminant analysis (LDA) of the faecal microbial OTUs between recovered NJI (neonatal jaundice infants) and non-NJI at 1 month (continued)

Biomaker names	Logarithm v	Groups	LDA value	P value
d Bacteria.p Cyanobacteria.c Cyanobacteria.o Cyanobacteria norank.f Cyanobacteria norank.g Cyanobacteria norank	1.5175004			-
d Bacteria.p Candidate division TM7	2.3031961	recovered NJI	3.508628	0.042697
d Bacteria.p Bacteroidetes.c Bacteroidia	5.2779956			-
d Bacteria.p Actinobacteria.c Actinobacteria.o Bifidobacteriales.f Bifidobacteriaceae.g Scardovia	2.8568218			-
d Bacteria.p Firmicutes.c Negativicutes.o Selenomonadales.f Veillonellaceae	4.8816436	non-NJI	4.51647	0.014059
d Bacteria.p Proteobacteria.c Gammaproteobacteria.o Enterobacteriales.f Enterobacteriaceae.g Enterobacter	3.9951017			-
d Bacteria.p Firmicutes.c Clostridia.o Clostridiales.f Ruminococcaceae.g Ruminococcus	1.6706739			-
d Bacteria.p Proteobacteria.c Deltaproteobacteria.o Desulfovibrionales.f Desulfovibrionaceae	1.7836837			-
d Bacteria.p Actinobacteria.c Actinobacteria.o Coriobacteriales	4.2668273			-
d Bacteria.p Proteobacteria.c Alphaproteobacteria.o Caulobacterales.f Caulobacteraceae.g Brevundimonas	1.7062581			-
d Bacteria.p Bacteroidetes.c Bacteroidia.o Bacteroidales.f Bacteroidaceae.g Bacteroides	5.1612946			-
d Bacteria.p Proteobacteria.c Gammaproteobacteria.o Enterobacteriales.f Enterobacteriaceae	5.4766754			-
d Bacteria.p Proteobacteria.c Alphaproteobacteria.o Rickettsiales	0.6922366			-
d Bacteria.p Proteobacteria.c Alphaproteobacteria.o Rhizobiales.f Brucellaceae.g Pseudochrobactrum	0.5642714			-
d Bacteria.p Proteobacteria.c Alphaproteobacteria.o Rhizobiales	1.0679002			-
d Bacteria.p Proteobacteria.c Gammaproteobacteria.o Enterobacteriales.f Enterobacteriaceae.g Citrobacter	4.1141515			-
d Bacteria.p Actinobacteria.c Actinobacteria.o Propionibacteriales.f Propionibacteriaceae	4.0394868			-
d Bacteria.p Candidate division TM7.c Candidate division TM7 norank.o Candidate division TM7 norank.f Candidate division TM7 norank	2.3031961	recovered NJI	3.383138	0.042697
d Bacteria	6			-
d Bacteria.p Proteobacteria.c Deltaproteobacteria.o Desulfovibrionales	1.7836837			-
d Bacteria.p Firmicutes.c Clostridia.o Clostridiales.f Peptostreptococcaceae.g Peptostreptococcaceae Incertae Sedis	3.1806992			-
d Bacteria.p Verrucomicrobia	2.9478483			-
d Bacteria.p Firmicutes.c Clostridia.o Clostridiales.f Peptostreptococcaceae	3.1806992			-
d Bacteria.p Firmicutes.c Clostridia.o Clostridiales.f Lachnospiraceae.g Roseburia	1.1009005			-
d Bacteria.p Proteobacteria.c Alphaproteobacteria.o Rhizobiales.f Rhizobiaceae.g Rhizobium	0.7311547			-
d Bacteria.p Proteobacteria.c Betaproteobacteria.o Burkholderiales.f Alcaligenaceae	2.6216955			-
d Bacteria.p Firmicutes.c Clostridia.o Clostridiales.f Ruminococcaceae.g Ruminococcaceae uncultured	2.2616593			-
d Bacteria.p Proteobacteria.c Epsilonproteobacteria.o Campylobacterales.f Campylobacteraceae	1.3238072			-
d Bacteria.p Actinobacteria.c Actinobacteria.o Corynebacteriales.f Corynebacteriaceae.g Corynebacterium	2.645586			-
d Bacteria.p Proteobacteria.c Gammaproteobacteria.o Pseudomonadales	2.9248821			-
d Bacteria.p Firmicutes.c Clostridia.o Clostridiales.f Ruminococcaceae	2.8107924			-
d Bacteria.p Firmicutes.c Negativicutes.o Selenomonadales.f Veillonellaceae.g Veillonella	4.8719046	non-NJI	4.516867	0.028333
d Bacteria.p Proteobacteria.c Gammaproteobacteria.o Pseudomonadales.f Moraxellaceae	2.9248821			-
d Bacteria.p Firmicutes.c Erysipelotrichia.o Erysipelotrichales.f Erysipelotrichaceae.g Erysipelotrichaceae uncultured	1.6368221			-
d Bacteria.p Actinobacteria.c Actinobacteria.o Bifidobacteriales.f Bifidobacteriaceae	5.2854313			-
d Bacteria.p Firmicutes.c Clostridia.o Clostridiales.f Defluviitaleaceae.g Defluviitaleaceae Incertae Sedis	2.9141804			-
d Bacteria.p Actinobacteria.c Actinobacteria.o Corynebacteriales.f Corynebacteriaceae	2.645586			-
d Bacteria.p Firmicutes.c Clostridia.o Clostridiales.f Lachnospiraceae.g Anaerostipes	1.436285			-
d Bacteria.p Firmicutes.c Clostridia.o Clostridiales.f Lachnospiraceae.g Howardella	0.5093059			-
d Bacteria.p Bacteroidetes.c Bacteroidia.o Bacteroidales.f Rikenellaceae.g Alistipes	3.5461724			-
d Bacteria.p Actinobacteria.c Actinobacteria.o Micrococcales.f Micrococcaceae.g Kocuria	0.2082759			-
d Bacteria.p Bacteroidetes.c Flavobacteriia.o Flavobacteriales	0.9927008			-
d Bacteria.p Actinobacteria.c Actinobacteria.o Coriobacteriales.f Coriobacteriaceae.g Collinsella	4.1616075			-
d Bacteria.p Firmicutes.c Bacilli.o Lactobacillales.f Streptococcaceae	5.2664389			-

Table S28. Detailed data for linear discriminant analysis (LDA) of the faecal microbial OTUs between recovered NJI (neonatal jaundice infants) and non-NJI at 1 month (continued)

Biomaker names	Logarithm v	Groups	LDA value	P value
d Bacteria.p Actinobacteria.c Actinobacteria.o Propionibacteriales	4.0394868			-
d Bacteria.p Fusobacteria	1.334763			-
d Bacteria.p Firmicutes.c Clostridia.o Clostridiales.f Lachnospiraceae.g Lachnospiraceae Incertae Sedis	4.2003351			-
d Bacteria.p Proteobacteria.c Epsilonproteobacteria	1.3238072			-
d Bacteria.p Proteobacteria.c Betaproteobacteria.o Burkholderiales.f Burkholderiaceae	1.6168389			-
d Bacteria.p Bacteroidetes.c Bacteroidia.o Bacteroidales.f Rikenellaceae	3.5461724			-
d Bacteria.p Firmicutes.c Negativicutes.o Selenomonadales.f Acidaminococcaceae.g Phascolarctobacterium	2.2438645			-
d Bacteria.p Proteobacteria.c Gammaproteobacteria.o Oceanospirillales.f Halomonadaceae	0.7611179			-
d Bacteria.p Actinobacteria.c Actinobacteria.o Coriobacteriales.f Coriobacteriaceae.g Gordonibacter	3.2233661			-
d Bacteria.p Proteobacteria.c Betaproteobacteria.o Neisseriales	2.0007232			-
d Bacteria.p Candidate division TM7.c Candidate division TM7 norank	2.3031961	recovered NJI	3.515763	0.042697
d Bacteria.p Firmicutes.c Erysipelotrichia.o Erysipelotrichales.f Erysipelotrichaceae.g Erysipelotrichaceae Incertae Sedis	3.9548471			-
d Bacteria.p Proteobacteria.c Betaproteobacteria	2.7302438			-
d Bacteria.p Bacteroidetes.c Bacteroidia.o Bacteroidales.f Porphyromonadaceae.g Odoribacter	0.7891466			-
d Bacteria.p Candidate division TM7.c Candidate division TM7 norank.o Candidate division TM7 norank	2.3031961	recovered NJI	3.596465	0.042697
d Bacteria.p Fusobacteria.c Fusobacteriia.o Fusobacteriales.f Fusobacteriaceae	1.334763			-
d Bacteria.p Firmicutes.c Bacilli.o Lactobacillales.f Enterococcaceae.g Enterococcus	4.176138			-
d Bacteria.p Firmicutes.c Bacilli.o Bacillales.f Bacillales Family XI	3.4216545			-
d Bacteria.p Proteobacteria.c Gammaproteobacteria.o Pseudomonadales.f Moraxellaceae.g Enhydrobacter	0.7493795			-
d Bacteria.p Firmicutes.c Bacilli.o Lactobacillales.f Lactobacillales unclassified	1.1648102			-
d Bacteria.p Firmicutes.c Negativicutes.o Selenomonadales.f Veillonellaceae.g Megasphaera	2.3817397	non-NJI	3.435179	0.040329
d Bacteria.p Proteobacteria.c Alphaproteobacteria.o Rhodobacterales.f Rhodobacteraceae.g Paracoccus	0.4045706			-
d Bacteria.p Fusobacteria.c Fusobacteriia	1.334763			-
d Bacteria.p Firmicutes.c Clostridia.o Clostridiales.f Lachnospiraceae.g Anaerosporobacter	0.7132104			-
d Bacteria.p Firmicutes	5.641916			-
d Bacteria.p Actinobacteria.c Actinobacteria.o Bifidobacteriales.f Bifidobacteriaceae.g Parascardovia	1.5399121			-
d Bacteria.p Bacteroidetes.c Bacteroidia.o Bacteroidales.f Prevotellaceae.g Prevotella	2.0661825	non-NJI	3.960371	0.023108
d Bacteria.p Actinobacteria.c Actinobacteria.o Actinomycetales	3.4838944			-
d Bacteria.p Proteobacteria.c Gammaproteobacteria.o Oceanospirillales	0.7611179			-
d Bacteria.p Proteobacteria.c Betaproteobacteria.o Burkholderiales.f Comamonadaceae.g Aquabacterium	0.30103			-
d Bacteria.p Firmicutes.c Clostridia.o Clostridiales.f Lachnospiraceae.g Oribacterium	0.3332147			-
d Bacteria.p Actinobacteria.c Actinobacteria.o Micrococcales	4.2197024			-
d Bacteria.p Bacteroidetes.c Bacteroidia.o Bacteroidales.f Porphyromonadaceae.g Parabacteroides	4.6493657			-
d Bacteria.p Firmicutes.c Bacilli.o Lactobacillales.f Lactobacillaceae.g Lactobacillus	4.7530808	non-NJI	4.508127	0.010976
d Bacteria.p Firmicutes.c Clostridia.o Clostridiales.f Lachnospiraceae.g Lachnospiraceae uncultured	0.7891466			-
d Bacteria.p Bacteroidetes.c Bacteroidia.o Bacteroidales.f Prevotellaceae	2.114457	non-NJI	3.801494	0.018239
d Bacteria.p Firmicutes.c Negativicutes.o Selenomonadales.f Veillonellaceae.g Dialister	2.9060448			-
d Bacteria.p Proteobacteria.c Alphaproteobacteria.o Rickettsiales.f mitochondria	0.6922366			-
d Bacteria.p Actinobacteria.c Actinobacteria.o Coriobacteriales.f Coriobacteriaceae.g Eggerthella	2.8979934			-
d Bacteria.p Actinobacteria.c Actinobacteria.o Bifidobacteriales	5.2854313			-
d Bacteria.p Firmicutes.c Clostridia.o Clostridiales.f Ruminococcaceae.g Ruminococcaceae Incertae Sedis	0.9852767			-
d Bacteria.p Firmicutes.c Clostridia.o Clostridiales.f Clostridiales Family XI.g Peptoniphilus	1.7702854			-
d Bacteria.p Bacteroidetes.c Bacteroidia.o Bacteroidales.f Porphyromonadaceae.g Dysgonomonas	1.50515			-
d Bacteria.p Proteobacteria.c Gammaproteobacteria.o Enterobacteriales.f Enterobacteriaceae.g Morganella	1.1995724			-

Table S28. Detailed data for linear discriminant analysis (LDA) of the faecal microbial OTUs between recovered NJI (neonatal jaundice infants) and non-NJI at 1 month (continued)

Biomaker names	Logarithm v	Groups	LDA value	P value
d Bacteria.p Bacteroidetes.c Bacteroidia.o Bacteroidales.f Porphyromonadaceae.g Barnesiella	0.3912066			-
d Bacteria.p Firmicutes.c Clostridia.o Clostridiales.f Lachnospiraceae.g Lachnoanaerobaculum	1.7062581			-
d Bacteria.p Proteobacteria.c Gammaproteobacteria.o Enterobacteriales.f Enterobacteriaceae.g Escherichia Shigella	5.2363848			-
d Bacteria.p Proteobacteria.c Betaproteobacteria.o Burkholderiales	2.640647			-
d Bacteria.p Firmicutes.c Bacilli.o Lactobacillales.f Carnobacteriaceae	4.0474501			-
d Bacteria.p Actinobacteria.c Actinobacteria.o Corynebacteriales	2.645586			-
d Bacteria.p Firmicutes.c Erysipelotrichia.o Erysipelotrichales.f Erysipelotrichaceae	3.9558172			-
d Bacteria.p Bacteroidetes.c Flavobacteriia	0.9927008			-
d Bacteria.p Firmicutes.c Clostridia.o Clostridiales.f Ruminococcaceae.g Flavonifractor	2.5987905			-
d Bacteria.p Verrucomicrobia.c Verrucomicrobiae.o Verrucomicrobiales	2.9478483			-
d Bacteria.p Proteobacteria.c Gammaproteobacteria.o Xanthomonadales	0.7781513			-
d Bacteria.p Firmicutes.c Clostridia	4.9260447			-
d Bacteria.p Firmicutes.c Clostridia.o Clostridiales.f Clostridiales Family XI.g Finegoldia	2.7140717			-
d Bacteria.p Bacteroidetes.c Bacteroidia.o Bacteroidales.f Porphyromonadaceae	4.6496772			-
d Bacteria.p Proteobacteria.c Gammaproteobacteria.o Pasteurellales.f Pasteurellaceae.g Actinobacillus	1.1760913			-
d Bacteria.p Bacteroidetes.c Flavobacteriia.o Flavobacteriales.f Flavobacteriaceae.g Chryseobacterium	0.9927008			-
d Bacteria.p Proteobacteria.c Gammaproteobacteria.o Pasteurellales.f Pasteurellaceae.g Aggregatibacter	0.9852767			-
d Bacteria.p Proteobacteria.c Gammaproteobacteria.o Enterobacteriales.f Enterobacteriaceae.g Enterobacteriaceae unclassified	4.0121848			-
d Bacteria.p Firmicutes.c Erysipelotrichia.o Erysipelotrichales.f Erysipelotrichaceae.g Solobacterium	1.1241028			-
d Bacteria.p Firmicutes.c Bacilli.o Lactobacillales.f Enterococcaceae	4.176138			-
d Bacteria.p Actinobacteria.c Actinobacteria.o Coriobacteriales.f Coriobacteriaceae	4.2668273			-
d Bacteria.p Proteobacteria.c Gammaproteobacteria.o Enterobacteriales.f Enterobacteriaceae.g Klebsiella	5.1819315			-
d Bacteria.p Actinobacteria.c Actinobacteria.o Micrococcales.f Micrococcaceae.g Arthrobacter	1.3469545			-
d Bacteria.p Proteobacteria.c Alphaproteobacteria.o Rhodobacterales.f Rhodobacteraceae	0.4045706			-
d Bacteria.p Proteobacteria.c Alphaproteobacteria.o Caulobacterales	1.7062581			-
d Bacteria.p Proteobacteria.c Betaproteobacteria.o Rhodocyclales	0.60206			-
d Bacteria.p Proteobacteria.c Betaproteobacteria.o Rhodocyclales.f Rhodocyclaceae.g Zoogloea	0.60206			-
d Bacteria.p Firmicutes.c Clostridia.o Clostridiales.f Clostridiaceae.g Clostridium sensu stricto	4.5826236			-
d Bacteria.p Proteobacteria.c Betaproteobacteria.o Neisseriales.f Neisseriaceae.g Neisseria	2.0007232			-
d Bacteria.p Proteobacteria.c Gammaproteobacteria.o Enterobacteriales.f Enterobacteriaceae.g Serratia	2.4008257			-
d Bacteria.p Proteobacteria.c Gammaproteobacteria	5.567902			-
d Bacteria.p Actinobacteria.c Actinobacteria.o Coriobacteriales.f Coriobacteriaceae.g Atopobium	3.614248			-
d Bacteria.p Proteobacteria.c Betaproteobacteria.o Neisseriales.f Neisseriaceae	2.0007232			-
d Bacteria.p Firmicutes.c Bacilli.o Lactobacillales.f Carnobacteriaceae.g Granulicatella	4.0469697			-
d Bacteria.p Proteobacteria.c Alphaproteobacteria.o Sphingomonadales	1.2496686			-
d Bacteria.p Firmicutes.c Clostridia.o Clostridiales.f Lachnospiraceae.g Lachnospira	1.3174204			-
d Bacteria.p Firmicutes.c Bacilli.o Bacillales.f Staphylococcaceae.g Staphylococcus	4.4634226			-
d Bacteria.p Verrucomicrobia.c Verrucomicrobiae.o Verrucomicrobiales.f Verrucomicrobiaceae	2.9478483			-
d Bacteria.p Firmicutes.c Bacilli.o Lactobacillales.f Aerococcaceae	0.9467545			-
d Bacteria.p Actinobacteria.c Actinobacteria.o Actinomycetales.f Actinomycetaceae.g Actinomyces	3.4838944			-
d Bacteria.p Proteobacteria.c Epsilonproteobacteria.o Campylobacterales.f Campylobacteraceae.g Campylobacter	1.3238072			-
d Bacteria.p Bacteroidetes.c Bacteroidia.o Bacteroidales	5.2779956			-
d Bacteria.p Firmicutes.c Bacilli.o Lactobacillales.f Aerococcaceae.g Abiotrophia	0.9467545			-
d Bacteria.p Firmicutes.c Bacilli.o Lactobacillales	5.4089353			-
d Bacteria.p Firmicutes.c Bacilli.o Lactobacillales.f Lactobacillaceae	4.7530808	non-NJI	4.508127	0.010976

Table S29. KEGG orthology functional terms identified by PICRUSt as different in recovered NJI (neonatal jaundice infants) and non-NJI at 1 month

Biomaker names	Logarithm value	Groups	LDA value	P value
L1 Metabolism.L2 Amino Acid Metabolism.L3 Valine leucine and isoleucine degradation	3.532424906			-
L1 Unclassified.L2 Cellular Processes and Signaling.L3 Germination	1.535408249			-
L1 Unclassified.L2 Metabolism.L3 Others	4.025375426			-
L1 Human Diseases.L2 Infectious Diseases.L3 Tuberculosis	3.004105152			-
L1 Metabolism.L2 Amino Acid Metabolism.L3 Arginine and proline metabolism	4.025658856			-
L1 Cellular Processes.L2 Cell Growth and Death.L3 Cell cycle Caulobacter	3.549245044			-
L1 Genetic Information Processing.L2 Translation.L3 Aminoacyl tRNA biosynthesis	3.963345206			-
L1 Metabolism.L2 Glycan Biosynthesis and Metabolism.L3 Glycosphingolipid biosynthesis globo series	2.898373016			-
L1 Metabolism.L2 Metabolism of Other Amino Acids.L3 D Arginine and D ornithine metabolism	1.467155923			-
L1 Metabolism.L2 Enzyme Families.L3 Protein kinases	3.643297912			-
L1 Genetic Information Processing.L2 Transcription.L3 Basal transcription factors	0			-
L1 Metabolism.L2 Xenobiotics Biodegradation and Metabolism.L3 Drug metabolism other enzymes	3.432768633			-
L1 Metabolism.L2 Xenobiotics Biodegradation and Metabolism.L3 Naphthalene degradation	3.142111793			-
L1 Human Diseases.L2 Metabolic Diseases	2.930822998			-
L1 Metabolism.L2 Energy Metabolism.L3 Sulfur metabolism	3.477978467			-
L1 Environmental Information Processing.L2 Membrane Transport.L3 Phosphotransferase system PTS	4.100698453			-
L1 Metabolism.L2 Lipid Metabolism.L3 Biosynthesis of unsaturated fatty acids	3.335554461			-
L1 Metabolism.L2 Amino Acid Metabolism.L3 Tyrosine metabolism	3.624549142			-
L1 Cellular Processes.L2 Cell Growth and Death.L3 Apoptosis	1.790624522			-
L1 Cellular Processes.L2 Cell Motility.L3 Cytoskeleton proteins	3.216389022			-
L1 Metabolism.L2 Biosynthesis of Other Secondary Metabolites	3.921452939			-
L1 Organismal Systems.L2 Environmental Adaptation.L3 Plant pathogen interaction	2.988214999			-
L1 Metabolism.L2 Metabolism of Terpenoids and Polyketides.L3 Polyketide sugar unit biosynthesis	3.12773606			-
L1 Metabolism.L2 Energy Metabolism.L3 Nitrogen metabolism	3.912495422			-
L1 Cellular Processes	4.264293803			-
L1 Metabolism.L2 Glycan Biosynthesis and Metabolism.L3 Lipopolysaccharide biosynthesis proteins	3.782430349			-
L1 Metabolism.L2 Metabolism of Terpenoids and Polyketides.L3 Zeatin biosynthesis	2.54594231			-
L1 Metabolism.L2 Metabolism of Other Amino Acids.L3 Taurine and hypotaurine metabolism	3.087394849			-
L1 Genetic Information Processing.L2 Replication and Repair.L3 DNA replication	3.725626181			-
L1 Human Diseases.L2 Neurodegenerative Diseases.L3 Parkinsons disease	0			-
L1 Human Diseases.L2 Neurodegenerative Diseases.L3 Alzheimers disease	2.722082361			-
L1 Metabolism.L2 Biosynthesis of Other Secondary Metabolites.L3 Penicillin and cephalosporin biosynthesis	2.744683953			-
L1 Metabolism	5.663632551			-
L1 Human Diseases.L2 Infectious Diseases	3.708845308			-
L1 Environmental Information Processing	5.287993698			-
L1 Metabolism.L2 Amino Acid Metabolism.L3 Lysine degradation	3.364640329			-
L1 Metabolism.L2 Metabolism of Terpenoids and Polyketides	4.178795812			-
L1 Unclassified.L2 Poorly Characterized.L3 Function unknown	4.298236691			-
L1 Metabolism.L2 Lipid Metabolism.L3 Steroid hormone biosynthesis	2.219202581			-
L1 Human Diseases.L2 Cancers.L3 Colorectal cancer	0			-
L1 Cellular Processes.L2 Cell Motility	4.088823747			-
L1 Metabolism.L2 Xenobiotics Biodegradation and Metabolism.L3 Polycyclic aromatic hydrocarbon degradation	2.882166022			-
L1 Metabolism.L2 Enzyme Families.L3 Cytochrome P450	0			-
L1 Human Diseases.L2 Infectious Diseases.L3 Chagas disease American trypanosomiasis	2.272597064			-

Table S29. KEGG orthology functional terms identified by PICRUSt as different in recovered NJI (neonatal jaundice infants) and non-NJI at 1 month (continued)

Biomaker names	Logarithm value	Groups	LDA value	P value
L1 Organismal Systems.L2 Endocrine System.L3 Melanogenesis	0			-
L1 Human Diseases.L2 Neurodegenerative Diseases.L3 Prion diseases	2.146479988			-
L1 Organismal Systems.L2 Environmental Adaptation.L3 Circadian rhythm plant	0			-
L1 Organismal Systems.L2 Digestive System.L3 Protein digestion and absorption	2.204591896			-
L1 Organismal Systems.L2 Immune System.L3 RIG I like receptor signaling pathway	1.720448699			-
L1 Metabolism.L2 Xenobiotics Biodegradation and Metabolism	4.320252583			-
L1 Human Diseases.L2 Infectious Diseases.L3 Vibrio cholerae infection	0			-
L1 Metabolism.L2 Biosynthesis of Other Secondary Metabolites.L3 Tropane piperidine and pyridine alkaloid biosynthesis	3.043467354			-
L1 Organismal Systems.L2 Excretory System	2.529526657			-
L1 Organismal Systems.L2 Digestive System.L3 Carbohydrate digestion and absorption	2.440479743			-
L1 Human Diseases.L2 Neurodegenerative Diseases.L3 Amyotrophic lateral sclerosis ALS	2.568525426			-
L1 Environmental Information Processing.L2 Signaling Molecules and Interaction.L3 Cellular antigens	2.734999915			-
L1 Unclassified.L2 Cellular Processes and Signaling.L3 Other transporters	3.425282819			-
L1 Metabolism.L2 Enzyme Families	4.323322252			-
L1 Human Diseases.L2 Infectious Diseases.L3 Staphylococcus aureus infection	2.603788631			-
L1 Metabolism.L2 Energy Metabolism.L3 Photosynthesis proteins	3.425368436			-
L1 Organismal Systems.L2 Endocrine System.L3 PPAR signaling pathway	2.928615818			-
L1 Metabolism.L2 Metabolism of Cofactors and Vitamins.L3 Thiamine metabolism	3.626229361			-
L1 Human Diseases.L2 Cancers.L3 Renal cell carcinoma	2.29523751			-
L1 Organismal Systems.L2 Endocrine System.L3 Insulin signaling pathway	2.8096776			-
L1 Human Diseases.L2 Cancers.L3 Bladder cancer	2.084610539			-
L1 Metabolism.L2 Energy Metabolism.L3 Oxidative phosphorylation	3.951972187			-
L1 Metabolism.L2 Metabolism of Terpenoids and Polyketides.L3 Limonene and pinene degradation	3.151695572			-
L1 Organismal Systems.L2 Endocrine System	3.348526098			-
L1 Unclassified.L2 Genetic Information Processing	4.388507872			-
L1 Metabolism.L2 Carbohydrate Metabolism.L3 Pentose phosphate pathway	3.918336411			-
L1 Metabolism.L2 Carbohydrate Metabolism.L3 Ascorbate and aldarate metabolism	3.516936575			-
L1 Genetic Information Processing.L2 Translation.L3 Ribosome biogenesis in eukaryotes	2.735690263			-
L1 Metabolism.L2 Biosynthesis of Other Secondary Metabolites.L3 beta Lactam resistance	2.607293803			-
L1 Organismal Systems.L2 Circulatory System	0			-
L1 Metabolism.L2 Lipid Metabolism.L3 Glycerophospholipid metabolism	3.725714437			-
L1 Metabolism.L2 Amino Acid Metabolism	4.950291027			-
L1 Unclassified.L2 Poorly Characterized.L3 General function prediction only	4.5273767			-
L1 Organismal Systems.L2 Excretory System.L3 Proximal tubule bicarbonate reclamation	2.529526657			-
L1 Metabolism.L2 Amino Acid Metabolism.L3 Cysteine and methionine metabolism	3.983207871			-
L1 Environmental Information Processing.L2 Membrane Transport.L3 Transporters	4.93647303			-
L1 Metabolism.L2 Biosynthesis of Other Secondary Metabolites.L3 Stilbenoid diarylheptanoid and gingerol biosynthesis	1.105702346			-
L1 Organismal Systems	3.715482164			-
L1 Unclassified.L2 Metabolism.L3 Energy metabolism	3.892554868			-
L1 Human Diseases.L2 Metabolic Diseases.L3 Type II diabetes mellitus	2.658976346			-
L1 Metabolism.L2 Energy Metabolism.L3 Photosynthesis antenna proteins	0			-
L1 Cellular Processes.L2 Cell Growth and Death.L3 p53 signaling pathway	0			-
L1 Metabolism.L2 Carbohydrate Metabolism.L3 Propanoate metabolism	3.779373418			-
L1 Metabolism.L2 Metabolism of Cofactors and Vitamins.L3 Retinol metabolism	2.787893479			-

Table S29. KEGG orthology functional terms identified by PICRUSt as different in recovered NJI (neonatal jaundice infants) and non-NJI at 1 month (continued)

Biomaker names	Logarithm value	Groups	LDA value	P value
L1 Unclassified.L2 Cellular Processes and Signaling.L3 Inorganic ion transport and metabolism	3.603387739			-
L1 Metabolism.L2 Metabolism of Cofactors and Vitamins.L3 Biotin metabolism	3.067900529			-
L1 Unclassified.L2 Cellular Processes and Signaling.L3 Cell division	2.8362957			-
L1 Unclassified.L2 Poorly Characterized	4.72206072			-
L1 Genetic Information Processing.L2 Folding Sorting and Degradation.L3 RNA degradation	3.57409272			-
L1 Metabolism.L2 Lipid Metabolism.L3 Ether lipid metabolism	1.419432288			-
L1 Metabolism.L2 Amino Acid Metabolism.L3 Histidine metabolism	3.658763637			-
L1 Unclassified.L2 Genetic Information Processing.L3 Translation proteins	3.920485692			-
L1 Unclassified.L2 Genetic Information Processing.L3 Transcription related proteins	2.54808405			-
L1 Metabolism.L2 Metabolism of Terpenoids and Polyketides.L3 Carotenoid biosynthesis	1.686045192			-
L1 Metabolism.L2 Energy Metabolism.L3 Carbon fixation in photosynthetic organisms	3.768218691			-
L1 Genetic Information Processing.L2 Replication and Repair.L3 Non homologous end joining	1.370652636			-
L1 Human Diseases.L2 Cancers.L3 Pathways in cancer	2.528487249			-
L1 Genetic Information Processing.L2 Replication and Repair.L3 Mismatch repair	3.791122691			-
L1 Metabolism.L2 Metabolism of Other Amino Acids.L3 D Alanine metabolism	3.033904468			-
L1 Metabolism.L2 Metabolism of Cofactors and Vitamins.L3 Nicotinate and nicotinamide metabolism	3.629466124			-
L1 Metabolism.L2 Glycan Biosynthesis and Metabolism.L3 Lipopolysaccharide biosynthesis	3.540284357			-
L1 Cellular Processes.L2 Transport and Catabolism.L3 Endocytosis	0			-
L1 Metabolism.L2 Metabolism of Terpenoids and Polyketides.L3 Tetracycline biosynthesis	3.075585777			-
L1 Environmental Information Processing.L2 Membrane Transport.L3 ABC transporters	4.660607723			-
L1 Human Diseases.L2 Immune System Diseases.L3 Systemic lupus erythematosus	0			-
L1 Organismal Systems.L2 Endocrine System.L3 Progesterone mediated oocyte maturation	2.280088883			-
L1 Metabolism.L2 Xenobiotics Biodegradation and Metabolism.L3 Benzoate degradation	3.555744795			-
L1 Metabolism.L2 Lipid Metabolism.L3 Glycerolipid metabolism	3.599416638			-
L1 Metabolism.L2 Carbohydrate Metabolism.L3 Fructose and mannose metabolism	3.981099356			-
L1 Metabolism.L2 Glycan Biosynthesis and Metabolism.L3 Glycosphingolipid biosynthesis lacto and neolacto series	0			-
L1 Metabolism.L2 Amino Acid Metabolism.L3 Lysine biosynthesis	3.791546398			-
L1 Metabolism.L2 Xenobiotics Biodegradation and Metabolism.L3 Chloroalkane and chloroalkene degradation	3.23795389			-
L1 Genetic Information Processing.L2 Folding Sorting and Degradation.L3 Sulfur relay system	3.550484763			-
L1 Cellular Processes.L2 Cell Growth and Death	3.560759135			-
L1 Metabolism.L2 Metabolism of Cofactors and Vitamins.L3 Porphyrin and chlorophyll metabolism	3.834477085			-
L1 Metabolism.L2 Energy Metabolism.L3 Photosynthesis	3.402149161			-
L1 Metabolism.L2 Metabolism of Cofactors and Vitamins.L3 One carbon pool by folate	3.663082762			-
L1 Metabolism.L2 Xenobiotics Biodegradation and Metabolism.L3 Aminobenzoate degradation	3.25519249			-
L1 Human Diseases.L2 Infectious Diseases.L3 Influenza A	0			-
L1 Metabolism.L2 Xenobiotics Biodegradation and Metabolism.L3 Ethylbenzene degradation	2.544897031			-
L1 Human Diseases.L2 Infectious Diseases.L3 Bacterial invasion of epithelial cells	1.410022515			-
L1 Metabolism.L2 Carbohydrate Metabolism.L3 Pentose and glucuronate interconversions	3.800842785			-
L1 Environmental Information Processing.L2 Signal Transduction.L3 Two component system	4.282503978			-
L1 Metabolism.L2 Lipid Metabolism.L3 Sphingolipid metabolism	3.184826957			-
L1 Metabolism.L2 Xenobiotics Biodegradation and Metabolism.L3 Drug metabolism cytochrome P450	2.984033813			-
L1 Genetic Information Processing.L2 Translation.L3 Ribosome Biogenesis	4.125811196			-
L1 Metabolism.L2 Metabolism of Terpenoids and Polyketides.L3 Biosynthesis of siderophore group nonribosomal peptides	3.172661377			-
L1 Organismal Systems.L2 Endocrine System.L3 Adipocytokine signaling pathway	2.737845282			-

Table S29. KEGG orthology functional terms identified by PICRUSt as different in recovered NJI (neonatal jaundice infants) and non-NJI at 1 month (continued)

Biomaker names	Logarithm value	Groups	LDA value	P value
L1 Metabolism.L2 Lipid Metabolism	4.454972635	recovered NJI	2.84175765	0.04367116
L1 Metabolism.L2 Nucleotide Metabolism.L3 Purine metabolism	4.316961664			-
L1 Human Diseases.L2 Metabolic Diseases.L3 Type I diabetes mellitus	2.608127976			-
L1 Human Diseases.L2 Infectious Diseases.L3 Epithelial cell signaling in Helicobacter pylori infection	2.838518175			-
L1 Metabolism.L2 Metabolism of Other Amino Acids	4.235562135			-
L1 Metabolism.L2 Xenobiotics Biodegradation and Metabolism.L3 Fluorobenzoate degradation	2.445117525			-
L1 Organismal Systems.L2 Immune System.L3 Antigen processing and presentation	2.280088883			-
L1 Genetic Information Processing.L2 Folding Sorting and Degradation.L3 Protein export	3.685764176			-
L1 Genetic Information Processing.L2 Transcription	4.477265042			-
L1 Environmental Information Processing.L2 Signal Transduction.L3 Phosphatidylinositol signaling system	2.977125649			-
L1 Genetic Information Processing.L2 Translation.L3 RNA transport	3.027245698			-
L1 Metabolism.L2 Biosynthesis of Other Secondary Metabolites.L3 Streptomycin biosynthesis	3.410828109			-
L1 Metabolism.L2 Lipid Metabolism.L3 Secondary bile acid biosynthesis	2.375810577			-
L1 Cellular Processes.L2 Cell Motility.L3 Bacterial motility proteins	3.792682141			-
L1 Organismal Systems.L2 Circulatory System.L3 Cardiac muscle contraction	0			-
L1 Human Diseases.L2 Infectious Diseases.L3 Toxoplasmosis	0			-
L1 Organismal Systems.L2 Endocrine System.L3 GnRH signaling pathway	0			-
L1 Human Diseases.L2 Infectious Diseases.L3 Pathogenic Escherichia coli infection	0			-
L1 Metabolism.L2 Biosynthesis of Other Secondary Metabolites.L3 Butirosin and neomycin biosynthesis	2.573095255			-
L1 Organismal Systems.L2 Digestive System.L3 Bile secretion	1.42987302			-
L1 Environmental Information Processing.L2 Signaling Molecules and Interaction.L3 Bacterial toxins	2.937041277			-
L1 Genetic Information Processing	5.233658659			-
L1 Metabolism.L2 Amino Acid Metabolism.L3 Amino acid related enzymes	4.090245976			-
L1 Cellular Processes.L2 Transport and Catabolism	3.404312594			-
L1 Metabolism.L2 Biosynthesis of Other Secondary Metabolites.L3 Betalain biosynthesis	0			-
L1 Metabolism.L2 Metabolism of Cofactors and Vitamins.L3 Ubiquinone and other terpenoid quinone biosynthesis	3.547887156			-
L1 Metabolism.L2 Amino Acid Metabolism.L3 Alanine aspartate and glutamate metabolism	3.932548659			-
L1 Cellular Processes.L2 Transport and Catabolism.L3 Peroxisome	3.261021632			-
L1 Human Diseases.L2 Immune System Diseases.L3 Primary immunodeficiency	2.728026779			-
L1 Metabolism.L2 Lipid Metabolism.L3 Steroid biosynthesis	0			-
L1 Genetic Information Processing.L2 Translation.L3 Translation factors	3.628380977			-
L1 Genetic Information Processing.L2 Translation	4.666763149			-
L1 Metabolism.L2 Biosynthesis of Other Secondary Metabolites.L3 Flavonoid biosynthesis	0.858021784			-
L1 Metabolism.L2 Metabolism of Terpenoids and Polyketides.L3 Biosynthesis of vancomycin group antibiotics	2.615145717			-
L1 Metabolism.L2 Glycan Biosynthesis and Metabolism.L3 Peptidoglycan biosynthesis	3.850054697			-
L1 Unclassified.L2 Cellular Processes and Signaling.L3 Signal transduction mechanisms	3.708290901			-
L1 Metabolism.L2 Xenobiotics Biodegradation and Metabolism.L3 Chlorocyclohexane and chlorobenzene degradation	2.343373623			-
L1 Environmental Information Processing.L2 Membrane Transport.L3 Bacterial secretion system	3.953997959			-
L1 Genetic Information Processing.L2 Folding Sorting and Degradation	4.348746269			-
L1 Genetic Information Processing.L2 Replication and Repair.L3 DNA replication proteins	3.980162539			-
L1 Metabolism.L2 Carbohydrate Metabolism.L3 Starch and sucrose metabolism	3.999097549	recovered NJI	2.73473634	0.04367116
L1 Genetic Information Processing.L2 Replication and Repair.L3 Base excision repair	3.571834397			-
L1 Metabolism.L2 Xenobiotics Biodegradation and Metabolism.L3 Metabolism of xenobiotics by cytochrome P450	2.994523337			-
L1 Metabolism.L2 Carbohydrate Metabolism.L3 Amino sugar and nucleotide sugar metabolism	4.13777525			-

Table S29. KEGG orthology functional terms identified by PICRUSt as different in recovered NJI (neonatal jaundice infants) and non-NJI at 1 month (continued)

Biomaker names	Logarithm value	Groups	LDA value	P value
L1 Metabolism.L2 Lipid Metabolism.L3 Lipid biosynthesis proteins	3.739414828			-
L1 Cellular Processes.L2 Cell Growth and Death.L3 Meiosis yeast	1.702724197			-
L1 Metabolism.L2 Glycan Biosynthesis and Metabolism.L3 Various types of N glycan biosynthesis	0			-
L1 Unclassified.L2 Cellular Processes and Signaling.L3 Cell motility and secretion	3.3159207			-
L1 Metabolism.L2 Amino Acid Metabolism.L3 Tryptophan metabolism	3.489711253			-
L1 Human Diseases.L2 Immune System Diseases	2.728051991			-
L1 Unclassified.L2 Cellular Processes and Signaling.L3 Other ion coupled transporters	4.21670326			-
L1 Environmental Information Processing.L2 Membrane Transport	5.236182038			-
L1 Metabolism.L2 Lipid Metabolism.L3 alpha Linolenic acid metabolism	2.545120195			-
L1 Genetic Information Processing.L2 Replication and Repair.L3 DNA repair and recombination proteins	4.383506844			-
L1 Genetic Information Processing.L2 Folding Sorting and Degradation.L3 Proteasome	2.511234637	recovered NJI	2.12854566	0.02258689
L1 Unclassified.L2 Cellular Processes and Signaling	4.664150534			-
L1 Human Diseases.L2 Infectious Diseases.L3 African trypanosomiasis	2.279651912			-
L1 Unclassified.L2 Cellular Processes and Signaling.L3 Pores ion channels	3.770035116			-
L1 Metabolism.L2 Carbohydrate Metabolism.L3 Inositol phosphate metabolism	3.334836003			-
L1 Metabolism.L2 Lipid Metabolism.L3 Fatty acid metabolism	3.584652202			-
L1 Metabolism.L2 Metabolism of Cofactors and Vitamins.L3 Vitamin B6 metabolism	3.318575092			-
L1 Organismal Systems.L2 Digestive System.L3 Mineral absorption	1.503456735			-
L1 Human Diseases.L2 Neurodegenerative Diseases.L3 Huntingtons disease	2.698429894			-
L1 Metabolism.L2 Carbohydrate Metabolism.L3 Butanoate metabolism	3.889248915			-
L1 Metabolism.L2 Glycan Biosynthesis and Metabolism.L3 N Glycan biosynthesis	2.020111138			-
L1 Genetic Information Processing.L2 Replication and Repair	4.861553412			-
L1 Metabolism.L2 Amino Acid Metabolism.L3 Phenylalanine metabolism	3.400541587			-
L1 Unclassified.L2 Metabolism.L3 Biosynthesis and biodegradation of secondary metabolites	3.171249196			-
L1 Organismal Systems.L2 Endocrine System.L3 Renin angiotensin system	0			-
L1 Unclassified.L2 Metabolism.L3 Glycan biosynthesis and metabolism	2.968350556			-
L1 Metabolism.L2 Xenobiotics Biodegradation and Metabolism.L3 Atrazine degradation	2.621679321			-
L1 Unclassified.L2 Metabolism.L3 Nucleotide metabolism	3.164499912			-
L1 Organismal Systems.L2 Immune System.L3 NOD like receptor signaling pathway	2.330129573			-
L1 Human Diseases.L2 Cancers	2.905805695			-
L1 Metabolism.L2 Carbohydrate Metabolism	5.053389414			-
L1 Metabolism.L2 Lipid Metabolism.L3 Synthesis and degradation of ketone bodies	2.677975393			-
L1 Metabolism.L2 Nucleotide Metabolism.L3 Pyrimidine metabolism	4.174772041			-
L1 Metabolism.L2 Metabolism of Cofactors and Vitamins.L3 Pantothenate and CoA biosynthesis	3.731072322			-
L1 Unclassified.L2 Cellular Processes and Signaling.L3 Electron transfer carriers	2.988823652			-
L1 Metabolism.L2 Metabolism of Terpenoids and Polyketides.L3 Geraniol degradation	3.138996052			-
L1 Unclassified.L2 Cellular Processes and Signaling.L3 Sporulation	2.878526741			-
L1 Metabolism.L2 Energy Metabolism	4.696629925			-
L1 Metabolism.L2 Carbohydrate Metabolism.L3 Glyoxylate and dicarboxylate metabolism	3.767124875			-
L1 Genetic Information Processing.L2 Folding Sorting and Degradation.L3 Chaperones and folding catalysts	3.975479023			-
L1 Metabolism.L2 Carbohydrate Metabolism.L3 Citrate cycle TCA cycle	3.824203084			-
L1 Genetic Information Processing.L2 Transcription.L3 RNA polymerase	3.143591965			-
L1 Organismal Systems.L2 Immune System	2.659971855			-
L1 Metabolism.L2 Energy Metabolism.L3 Carbon fixation pathways in prokaryotes	3.951790537			-

Table S29. KEGG orthology functional terms identified by PICRUSt as different in recovered NJI (neonatal jaundice infants) and non-NJI at 1 month (continued)

Biomaker names	Logarithm value	Groups	LDA value	P value
L1 Metabolism.L2 Metabolism of Terpenoids and Polyketides.L3 Prenyltransferases	3.386537939			-
L1 Human Diseases.L2 Infectious Diseases.L3 Shigellosis	0			-
L1 Environmental Information Processing.L2 Signaling Molecules and Interaction	3.266490811			-
L1 Metabolism.L2 Biosynthesis of Other Secondary Metabolites.L3 Caffeine metabolism	0			-
L1 Metabolism.L2 Lipid Metabolism.L3 Linoleic acid metabolism	2.321329856			-
L1 Human Diseases.L2 Neurodegenerative Diseases	3.186787586			-
L1 Environmental Information Processing.L2 Signal Transduction.L3 MAPK signaling pathway yeast	2.651935989			-
L1 Environmental Information Processing.L2 Signal Transduction	4.312999498			-
L1 Metabolism.L2 Glycan Biosynthesis and Metabolism.L3 Glycosphingolipid biosynthesis ganglio series	2.498010684			-
L1 Genetic Information Processing.L2 Transcription.L3 Transcription machinery	3.750572967			-
L1 Metabolism.L2 Carbohydrate Metabolism.L3 Galactose metabolism	3.896999801			-
L1 Human Diseases.L2 Cancers.L3 Small cell lung cancer	0			-
L1 Metabolism.L2 Glycan Biosynthesis and Metabolism.L3 Glycosyltransferases	3.63410176			-
L1 Genetic Information Processing.L2 Replication and Repair.L3 Chromosome	4.125207537			-
L1 Genetic Information Processing.L2 Folding Sorting and Degradation.L3 Protein processing in endoplasmic reticulum	2.495268796			-
L1 Metabolism.L2 Xenobiotics Biodegradation and Metabolism.L3 Bisphenol degradation	2.446538491			-
L1 Environmental Information Processing.L2 Signaling Molecules and Interaction.L3 Ion channels	2.690575921			-
L1 Human Diseases.L2 Infectious Diseases.L3 Pertussis	3.237597525			-
L1 Unclassified.L2 Cellular Processes and Signaling.L3 Membrane and intracellular structural molecules	3.892886176			-
L1 Genetic Information Processing.L2 Transcription.L3 Transcription factors	4.369544485			-
L1 Environmental Information Processing.L2 Membrane Transport.L3 Secretion system	4.266933829			-
L1 Metabolism.L2 Biosynthesis of Other Secondary Metabolites.L3 Flavone and flavonol biosynthesis	1.756462772			-
L1 Organismal Systems.L2 Environmental Adaptation	2.988330313			-
L1 Genetic Information Processing.L2 Replication and Repair.L3 Nucleotide excision repair	3.437972562			-
L1 Metabolism.L2 Nucleotide Metabolism	4.552690218			-
L1 Unclassified.L2 Metabolism.L3 Metabolism of cofactors and vitamins	3.296467835			-
L1 Metabolism.L2 Metabolism of Other Amino Acids.L3 Selenocompound metabolism	3.612170963			-
L1 Metabolism.L2 Metabolism of Cofactors and Vitamins	4.59654028			-
L1 Unclassified.L2 Genetic Information Processing.L3 Replication recombination and repair proteins	3.835739159			-
L1 Metabolism.L2 Metabolism of Other Amino Acids.L3 Phosphonate and phosphinate metabolism	2.897035875			-
L1 Metabolism.L2 Lipid Metabolism.L3 Primary bile acid biosynthesis	2.376054037			-
L1 Metabolism.L2 Metabolism of Terpenoids and Polyketides.L3 Biosynthesis of ansamycins	3.073732035			-
L1 Metabolism.L2 Metabolism of Other Amino Acids.L3 D Glutamine and D glutamate metabolism	3.11986706			-
L1 Human Diseases.L2 Infectious Diseases.L3 Vibrio cholerae pathogenic cycle	2.955017635			-
L1 Metabolism.L2 Xenobiotics Biodegradation and Metabolism.L3 Toluene degradation	3.210527702			-
L1 Human Diseases.L2 Cardiovascular Diseases.L3 Hypertrophic cardiomyopathy HCM	0			-
L1 Metabolism.L2 Metabolism of Cofactors and Vitamins.L3 Lipoic acid metabolism	2.786430936			-
L1 Metabolism.L2 Glycan Biosynthesis and Metabolism.L3 Other glycan degradation	3.314854764			-
L1 Metabolism.L2 Glycan Biosynthesis and Metabolism.L3 Glycosaminoglycan degradation	2.665517206			-
L1 Genetic Information Processing.L2 Folding Sorting and Degradation.L3 Ubiquitin system	2.485899754			-
L1 Metabolism.L2 Lipid Metabolism.L3 Fatty acid elongation in mitochondria	0			-
L1 Metabolism.L2 Energy Metabolism.L3 Methane metabolism	3.993535254			-
L1 Organismal Systems.L2 Nervous System.L3 Glutamatergic synapse	2.866272307			-
L1 Human Diseases.L2 Cardiovascular Diseases.L3 Viral myocarditis	0			-

Table S29. KEGG orthology functional terms identified by PICRUSt as different in recovered NJI (neonatal jaundice infants) and non-NJI at 1 month (continued)

Biomaker names	Logarithm value	Groups	LDA value	P value
L1 Cellular Processes.L2 Transport and Catabolism.L3 Lysosome	2.85302189			-
L1 Human Diseases.L2 Cancers.L3 Prostate cancer	2.298273858			-
L1 Metabolism.L2 Xenobiotics Biodegradation and Metabolism.L3 Dioxin degradation	3.104538231			-
L1 Metabolism.L2 Carbohydrate Metabolism.L3 Glycolysis Gluconeogenesis	4.070076912			-
L1 Metabolism.L2 Metabolism of Cofactors and Vitamins.L3 Riboflavin metabolism	3.397754195			-
L1 Metabolism.L2 Xenobiotics Biodegradation and Metabolism.L3 Xylene degradation	2.941412986			-
L1 Metabolism.L2 Carbohydrate Metabolism.L3 C5 Branched dibasic acid metabolism	3.508260974			-
L1 Unclassified.L2 Metabolism.L3 Carbohydrate metabolism	3.347375159	recovered NJI	2.36011636	0.04367116
L1 Metabolism.L2 Carbohydrate Metabolism.L3 Pyruvate metabolism	4.044824805			-
L1 Genetic Information Processing.L2 Translation.L3 Ribosome	4.255673926			-
L1 Metabolism.L2 Lipid Metabolism.L3 Arachidonic acid metabolism	2.733516241			-
L1 Metabolism.L2 Amino Acid Metabolism.L3 Glycine serine and threonine metabolism	3.930205611			-
L1 Metabolism.L2 Amino Acid Metabolism.L3 Phenylalanine tyrosine and tryptophan biosynthesis	3.837867111			-
L1 Organismal Systems.L2 Nervous System	2.866272307			-
L1 Metabolism.L2 Lipid Metabolism.L3 Fatty acid biosynthesis	3.637879935			-
L1 Metabolism.L2 Metabolism of Cofactors and Vitamins.L3 Folate biosynthesis	3.612202792			-
L1 Metabolism.L2 Biosynthesis of Other Secondary Metabolites.L3 Phenylpropanoid biosynthesis	3.195735249			-
L1 Metabolism.L2 Xenobiotics Biodegradation and Metabolism.L3 Nitrotoluene degradation	2.866828593			-
L1 Metabolism.L2 Biosynthesis of Other Secondary Metabolites.L3 Isoquinoline alkaloid biosynthesis	2.760437349			-
L1 Metabolism.L2 Xenobiotics Biodegradation and Metabolism.L3 Styrene degradation	2.671313177			-
L1 Metabolism.L2 Glycan Biosynthesis and Metabolism	4.375301377			-
L1 Human Diseases	3.945029262			-
L1_Metabolism.L2_Xenobiotics_Biodegradation_and_Metabolism.L3_1_1_1_Trichloro_2_2_bis_4_chlorophenyl_ethane__DDT__degradation	0			-
L1 Organismal Systems.L2 Digestive System	2.679621698	recovered NJI	2.11141813	0.04367116
L1 Unclassified.L2 Metabolism	4.484471233			-
L1 Genetic Information Processing.L2 Replication and Repair.L3 Homologous recombination	3.882576082			-
L1 Organismal Systems.L2 Immune System.L3 Fc gamma R mediated phagocytosis	0			-
L1 Cellular Processes.L2 Cell Motility.L3 Bacterial chemotaxis	3.384165726			-
L1 Metabolism.L2 Metabolism of Other Amino Acids.L3 beta Alanine metabolism	3.408797557			-
L1 Unclassified.L2 Genetic Information Processing.L3 Protein folding and associated processing	3.87650241			-
L1 Metabolism.L2 Amino Acid Metabolism.L3 Valine leucine and isoleucine biosynthesis	3.829001799			-
L1 Metabolism.L2 Metabolism of Other Amino Acids.L3 Glutathione metabolism	3.560720701			-
L1 Metabolism.L2 Biosynthesis of Other Secondary Metabolites.L3 Novobiocin biosynthesis	3.074016204			-
L1 Metabolism.L2 Enzyme Families.L3 Peptidases	4.223167612			-
L1 Unclassified.L2 Genetic Information Processing.L3 Restriction enzyme	3.148295408			-
L1 Unclassified.L2 Metabolism.L3 Lipid metabolism	3.207025636			-
L1 Cellular Processes.L2 Cell Motility.L3 Flagellar assembly	3.300479311			-
L1 Unclassified	5.184783288			-
L1 Metabolism.L2 Xenobiotics Biodegradation and Metabolism.L3 Caprolactam degradation	3.042538969			-
L1 Human Diseases.L2 Cardiovascular Diseases	0			-
L1 Metabolism.L2 Metabolism of Other Amino Acids.L3 Cyanoamino acid metabolism	3.43322003			-
L1 Metabolism.L2 Metabolism of Terpenoids and Polyketides.L3 Terpenoid backbone biosynthesis	3.619008043			-
L1 Unclassified.L2 Metabolism.L3 Amino acid metabolism	3.458827531			-
L1 Human Diseases.L2 Infectious Diseases.L3 Amoebiasis	1.710576212			-
L1 Metabolism.L2 Biosynthesis of Other Secondary Metabolites.L3 Isoflavonoid biosynthesis	0			-
